# Next-Generation Sequencing Reveals Recent Horizontal Transfer of a DNA Transposon between Divergent Mosquitoes

**DOI:** 10.1371/journal.pone.0016743

**Published:** 2011-02-10

**Authors:** Yupu Diao, Yumin Qi, Yajun Ma, Ai Xia, Igor Sharakhov, Xiaoguang Chen, Jim Biedler, Erjun Ling, Zhijian Jake Tu

**Affiliations:** 1 Institute of Plant Physiology and Ecology, Shanghai Institute for Biological Sciences, Chinese Academy of Sciences, Shanghai, China; 2 Department of Biochemistry, Virginia Tech, Blacksburg, Virginia, United States of America; 3 Department of Etiologic Biology, Second Military Medical University, Shanghai, China; 4 Department of Entomology, Virginia Tech, Blacksburg, Virginia, United States of America; 5 Department of Parasitology, Southern Medical University, Guangzhou, China; The Salk Institute, United States of America

## Abstract

Horizontal transfer of genetic material between complex organisms often involves transposable elements (TEs). For example, a DNA transposon *mariner* has been shown to undergo horizontal transfer between different orders of insects and between different phyla of animals. Here we report the discovery and characterization of an *ITmD37D* transposon, *MJ1*, in *Anopheles sinensis*. We show that some *MJ1* elements in *Aedes aegypti* and *An. sinensis* contain intact open reading frames and share nearly 99% nucleotide identity over the entire transposon, which is unexpectedly high given that these two genera had diverged 145–200 million years ago. Chromosomal hybridization and TE-display showed that *MJ1* copy number is low in *An. sinensis*. Among 24 mosquito species surveyed, *MJ1* is only found in *Ae. aegypti* and the hyrcanus group of anopheline mosquitoes to which *An. sinensis* belongs. Phylogenetic analysis is consistent with horizontal transfer and provides the basis for inference of its timing and direction. Although report of horizontal transfer of DNA transposons between higher eukaryotes is accumulating, our analysis is one of a small number of cases in which horizontal transfer of nearly identical TEs among highly divergent species has been thoroughly investigated and strongly supported. Horizontal transfer involving mosquitoes is of particular interest because there are ongoing investigations of the possibility of spreading pathogen-resistant genes into mosquito populations to control malaria and other infectious diseases. The initial indication of horizontal transfer of *MJ1* came from comparisons between a 0.4x coverage *An. sinensis* 454 sequence database and available TEs in mosquito genomes. Therefore we have shown that it is feasible to use low coverage sequencing to systematically uncover horizontal transfer events. Expanding such efforts across a wide range of species will generate novel insights into the relative frequency of horizontal transfer of different TEs and provide the evolutionary context of these lateral transfer events.

## Introduction

Horizontal transfer is the transfer of genetic material between reproductively isolated species, which is common among prokaryotes [1]. Horizontal transfer between complex organisms is generally less frequent and often involves transposable elements (TEs) [2], [3]. *Mariner*, a DNA transposon originally discovered in *Drosophila mauritiana* [4], has been shown to undergo horizontal transfer across different orders of insects and even across different phyla of animals [5], [6]. More recently, examples of horizontal transfer of DNA transposons have been found in plants [7] and mammals [8]. DNA transposons are Class II TEs. They usually contain 10–200 bp terminal inverted-repeats (TIRs) which flank one or more open reading frames that encode a transposase. Members of the *IS630-Tc1-mariner* (*ITm*) superfamily share a transposase that contains a conserved D(Asp)DE(Glu) or DDD catalytic triad [9], [10]. The *IS630-Tc1-mariner* superfamily can be organized in several families including *Tc1*, *mariner*, *ITmD37E* and *ITmD37D*, which are characterized by unique catalytic motifs of DD34E, DD34D, DD37E, and DD37D, respectively [10]. The numbering, which is conserved within each family, refers to the distance between the second D and the third D or E residues of the catalytic triad.

There are generally three lines of evidence indicating the occurrence of horizontal transfer: high sequence identity between TEs from divergent taxa, incongruence between TE phylogeny and host phylogeny, and patchy distribution of TEs among related host species [11], [12]. Two types of approaches have been employed to systematically uncover evidence of TE horizontal transfer. PCR survey of diverse organisms followed by sequence and evolutionary analysis has been a productive approach to investigate horizontal transfer of a particular TE of interest [5], [6], [13]. More recently, comparative analysis of *Drosophila* genomes uncovered evidence of potentially new horizontal transfer events and revealed that various groups of TEs showed different propensity to undergo horizontal transfer [14], [15]. Such whole-genome analysis, when expanded to diverse taxa beyond model organisms, will likely generate novel insights into the relative frequency of horizontal transfer of different TEs and provide the evolutionary context of these lateral transfer events.

Here we report the discovery and characterization of an *ITmD37D* transposon, *MJ1*, in an important malaria vector in Asia, *Anopheles sinensis*. *MJ1* elements in *Aedes aegypti* and *An. sinensis* share 97% to nearly 99% nucleotide identity over the entire transposon, which is unexpectedly high given that these genera diverged 145–200 million years ago [16]. Phylogenetic analysis of all *MJ1* sequences obtained from a survey of 24 mosquito species is consistent with horizontal transfer and leads to hypotheses on the timing and direction of horizontal transfer, which may be tested in the future by expanding the survey of *MJ1* sequences. Our analysis is one of a small number of cases in which horizontal transfer of nearly identical TEs among highly divergent species has been thoroughly investigated and strongly supported. We discuss the implications of our finding in light of the ongoing investigations of the possibility of spreading pathogen-resistant genes into mosquito populations to control malaria and other infectious diseases. The initial indication of horizontal transfer of *MJ1* came from systematic comparisons between a 0.4x coverage *An. sinensis* 454 sequence database and available TEs in mosquito genomes. Therefore our success indicate that it is feasible to use low coverage sequencing to move beyond model organisms and systematically uncover new horizontal transfer events. We expect this type of analysis will quickly expand into a diverse range of organisms as sequencing technologies rapidly improve.

## Results

### Search of a 0.4x coverage *An. sinensis* sequence database revealed fragments that are nearly identical to an *Ae. aegypti MJ1* transposon

BLAST searches were performed on a 0.4x coverage 454 shotgun sequence database of *An. sinensis*, using a list of 1090 annotated TEs from *Ae. aegypti* as query ([17] and tefam.biochem.vt.edu). *Aae*_*MJ1* (TF000904, tefam.biochem.vt.edu) matched eight of the *An. sinensis* 454 shotgun sequences with 97–99% identity. Considering that *Aedes* and *Anopheles* mosquitoes diverged 145–200 million years ago [16], this level of identity offers a clue for possible horizontal transfer. *MJ1* is an *ITmD37D* DNA transposon and *Aae_MJ1* refers to the *MJ1* that was first found in the yellow fever mosquito *Ae. aegypti* [17]. It contains an intact open reading frame with a DD37D catalytic triad [10], where D stands for aspartic acid. *Aae_MJ1* (Aae refers to the genus and species) consists of 9 full-length copies in the genome, three of which share >99% nucleotide identity. The average length of the *An. sinensis* 454 shotgun sequences is 230 bp and the matches to *Aae_MJ1* were 100–300 bp in length. Two of these hits were near the termini of *MJ1* and had flanking sequences that were specific to *An. sinensis*. Of the 1090 elements analyzed during the BLAST searches, *Aae_MJ1* is the only element that showed such a high similarity to *An. sinensis* sequences. The next best match was a *Tc1* element (TF000536, 86% identity over a 260-bp fragment), which may result from a more ancient horizontal transfer event and is beyond the scope of the current investigation.

### Full-length *MJ1* elements in *An. sinensis* and *Ae. aegypti* share up to 99% nucleotide identity

**Figure 1:**
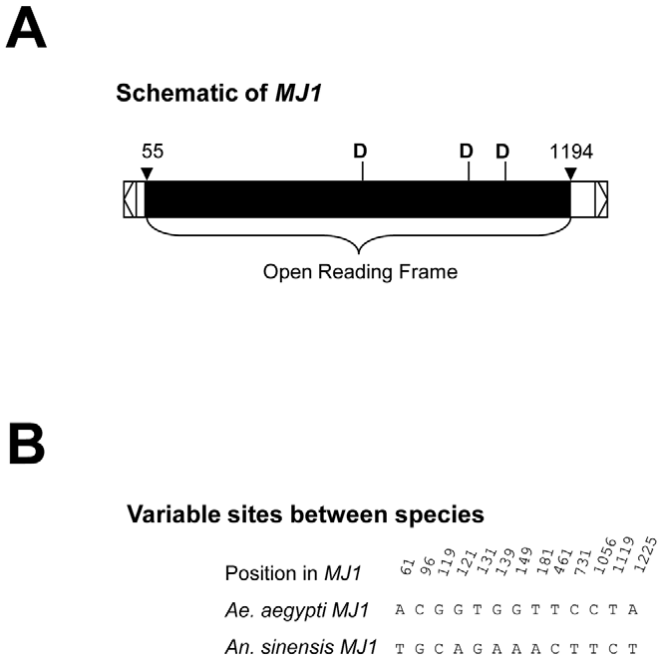
*MJ1* schematic and sequence comparison. A) *MJ1* schematic drawn according to the *MJ1* consensus from *Ae. aegypti*. The schematic shows terminal inverted repeats (open arrows at the termini), an open reading frame (black bar with the start and end positions marked), and the relative positions of the catalytic triad, which is comprised of three aspartic acid (D) residues. B) Comparison of *MJ1* sequences from *Ae. aegypti* and *An. sinensis*. Only variable sites are shown between the *Aae_MJ1* consensus and *Asi_MJ1_Clone1*, a representative of *Asi_MJ1* from *An. sinensis*. A one-base insertion between positions 1246 and 1247 in *An. sinensis* is not shown. The entire nucleotide sequences are shown in Supplemental Files S1 and S2.

Full-length *MJ1* sequences were independently obtained in two laboratories from two *An. sinensis* sources by PCR using the terminal inverted repeat as the primer, which anneal to both ends of *MJ1*. Nine clones were sequenced and all were confirmed to be *An. sinensis MJ1* (*Asi_MJ1*). These nine clones were nearly identical to each other with some having a 19 nucleotide insertion. As shown in Figure 1, one *Asi_MJ1* clone was 99% identical to the *Aae_MJ1* consensus over the entire 1.3 kb element. The open reading frames of the two sequences encoded 379 amino acids, which showed >97% identity. Sequences of the *Aae_MJ1* consensus and all nine genomic copies in *Ae. aegypti* are shown in Supplemental File S1 and sequences of the nine *Asi_MJ1* clones are included in Supplemental File S2. Deduced peptide sequences of the *Aae_MJ1* consensus and all individual *MJ1* copies/clones that had intact open reading frames are included in Supplemental File S3. When individual *MJ1* copies in *Ae. aegypti* were compared to individual *MJ1* clones in *An. sinensis* at the nucleotide level, high sequence identities were observed, ranging from 97% to nearly 99%. Similar high identities were also observed at the amino acid level. For example, the *Aae_MJ1* in CONTIG_13910, the only *Ae. aegypti MJ1* copy that has an intact open reading frame, shares 97% amino acid identity with *Asi_MJ1_Clone1*. The fact that full-length *MJ1* elements from *Aedes* and *Anopheles* mosquitoes are highly similar at both nucleotide and the amino acid levels indicates the possibility of horizontal transfer, considering that the two genera had diverged 145–200 million years ago [16]. In comparison, the range of amino acid identities between randomly selected orthologous gene products in *Ae. aegypti* and *An. gambiae* was from 28 to 96% with an average of 43% [18].

### Further confirmation of the presence of *MJ1* transposons in *An. sinensis*

**Figure 2:**
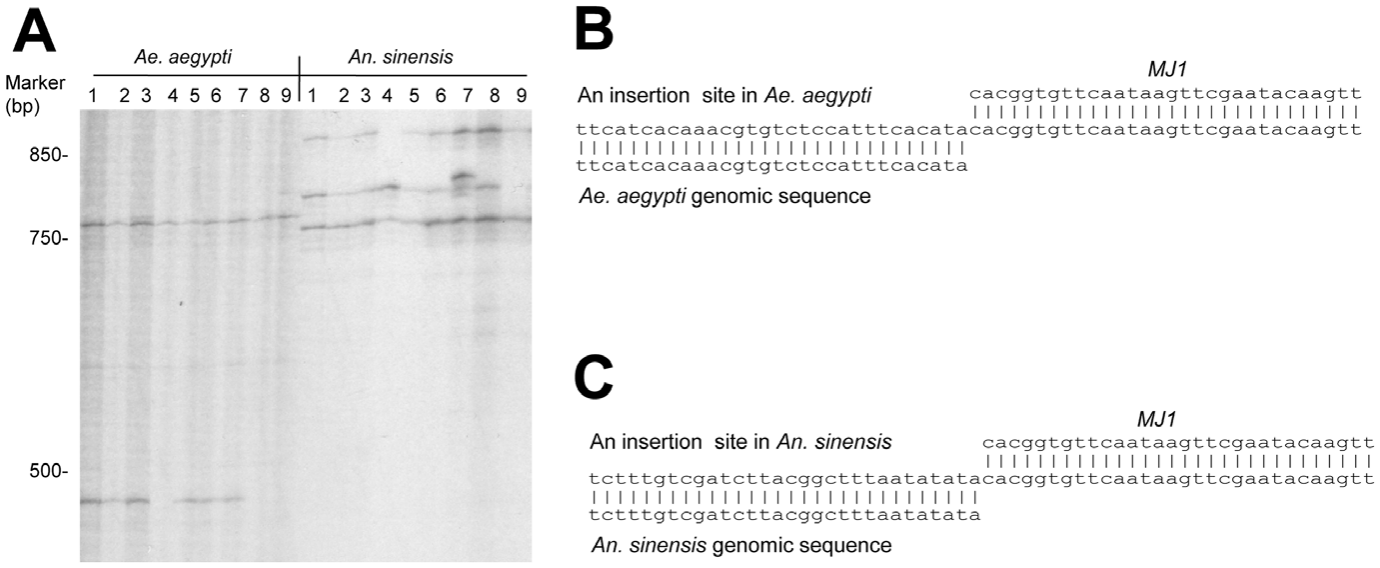
*MJ1* display. A) TE-display showing *MJ1* insertion sites in individual *Ae. aegypti* and *An. sinensis* mosquitoes. Results from nine individuals of each species are shown. Only part of the TE-display gel is shown. B) and C). Specific *MJ1* insertion sites from *Ae. aegypti* and *An. sinensis*, respectively. The sequence in the middle is the insertion site sequence recovered from TE-display, which consists of both the *MJ1* sequence and the flanking genomic sequence. Sequences flanking *MJ1* in *Ae. aegypti* and *An. sinensis* only match their respective genomes. Only parts of the sequences are shown.

**Figure 3:**
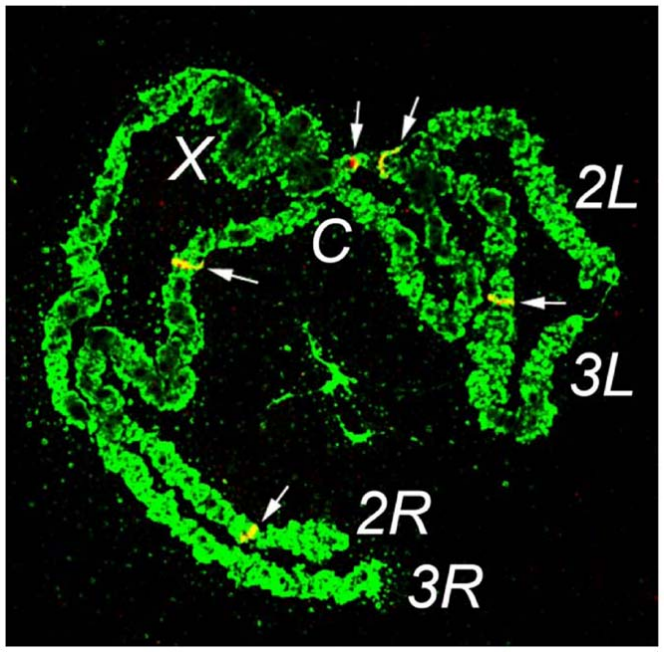
Fluorescence *in situ* hybridization confirmed the presence of *MJ1* in *An. sinensis*. Arrows point to signals on *An. sinensis* polytene chromosomes, resulting from hybridization with an *Asi_MJ1* probe.

We performed TE-display [19] to compare and isolate *MJ1* insertion sites in individual *Ae. aegypti* and *An. sinensis* mosquitoes. There were no shared bands (or shared insertion sites) between *Ae. aegypti* and *An. sinensis* while there were multiple shared bands among individuals within each species (Figure 2). We cloned and sequenced a few of these bands recovered from TE-display gels, which further confirmed the presence of *MJ1* in both species. More importantly, the recovered insertion site sequences were specific to each species (Figure 2). In other words, sequence flanking the *MJ1* insertion site that was recovered from *Ae. aegypti* matched *Ae. aegypti* genomic sequence alone. Sequence flanking the *MJ1* insertion recovered from *An. sinensis* matched *An. sinensis* genomic sequence alone. Using *Asi_MJ1* as a probe, we performed *in situ* hybridization on the polytene chromosomes of *An. sinensis*. A representative image is shown in Figure 3 and five distinct bands are apparent. These results further confirmed the presence of *Asi_MJ1* in *An. sinensis*. Although it is difficult to determine the exact copy number of *MJ1* on the basis of *in situ* and TE-display results, both experiments suggest that the copy number of *MJ1* in *An. sinensis* is low, most likely less than 10 copies per genome.

### Patchy distribution and *d*_N_/*d*_S_ results are consistent with horizontal transfer of *MJ1*

**Figure 4:**
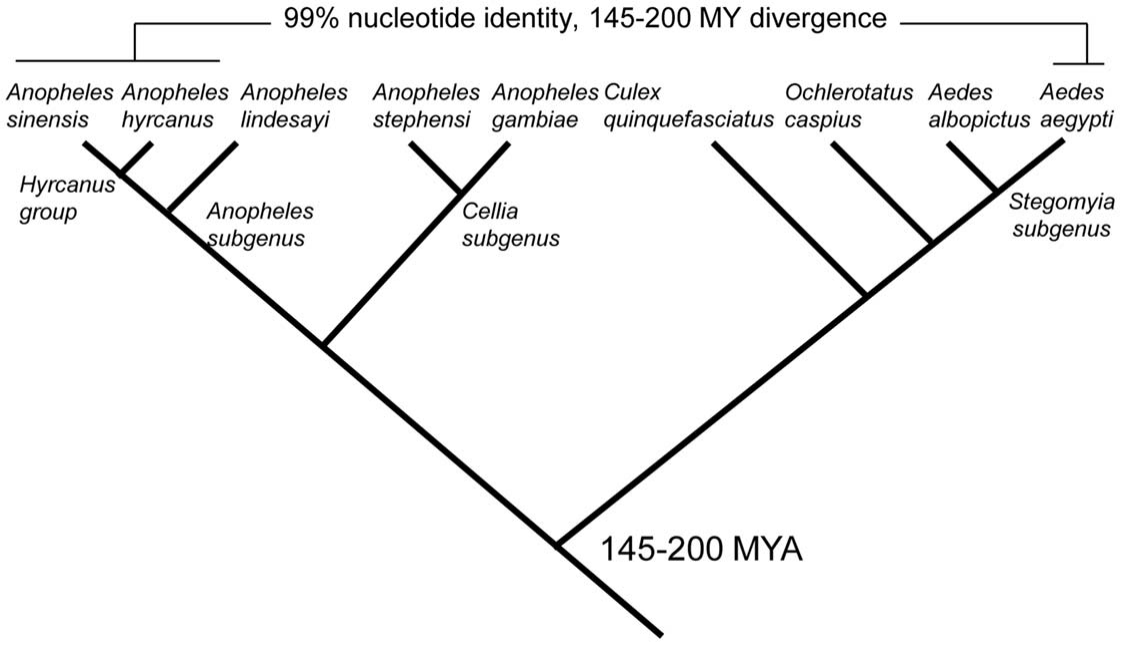
Distribution of *MJ1* in representatives of the mosquito species surveyed. Details and a full species list are provided in Table 1. The three species (*Anopheles sinensis*, *Anopheles hyrcanus*, and *Aedes aegypti*) that have *MJ1* are highlighted by the horizontal lines. All other species do not have *MJ1*. The *Anopheles* and *Aedes* genera were estimated to have diverged 145–200 million years ago [16].

A broad survey of *MJ1* in 24 species within 5 genera is shown in Table 1. Presence or absence of *MJ1* was determined by genomic PCR followed by sequencing. In the case of *An. gambiae*, *An. stephensi*, and *Cx. quinquefasciatus*, the absence of *MJ1* was also confirmed by analysis of the genome assembly as well as trace files. *MJ1* is restricted to *Ae. aegypti* and the hyrcanus group of *Anopheles* mosquitoes, to which *An. sinensis* belongs. As shown in Table 1, 10 of the 11 species within the hyrcanus group have *MJ1* sequences. *MJ1* was not found in eight *Anopheles* species outside the hyrcanus group, including *An. lindesayi*, a species that belongs to the same subgenus as the hyrcanus group. *MJ1* was also not detected in four Culicinae mosquitoes, including *Ae. albopictus*, a species that is within the same subgenus as *Ae. aegypti*. All *MJ1* copies that were obtained by PCR were confirmed by sequencing and special attention was paid to minimize false positive and false negative results as described in Methods and in Table 1. All *MJ1* sequences, the nine genomic copies from *Ae. aegypti* and the 55 PCR clones from different *Anopheles* species within the hyrcanus group, are shown in Supplemental Files S1 and S2, respectively. An abbreviated schematic summary of the survey results is also shown in Figure 4, highlighting the fact that *MJ1* is restricted to *Ae. aegypti* and the hyrcanus group of *Anopheles* mosquitoes. Overall, the pattern of patchy species distribution described in this section coupled with up to 99% sequence identity between *MJ1* elements in *Aedes* and *Anopheles* mosquito species strongly suggests a recent horizontal transfer event.

**Table 1:** Distribution of *MJ1* is consistent with horizontal transfer.

Genus	Subgenus	Species (Group)	*MJ1* 1
*Anopheles*	*Anopheles*	*sinensis (hyrcanus)*	+ (9/9)
*Anopheles*	*Anopheles*	*lesteri (hyrcanus)*	+ (13/13)
*Anopheles*	*Anopheles*	*kleini (hyrcanus)*	+ (3/3)
*Anopheles*	*Anopheles*	*hyrcanus (hyrcanus)*	+ (3/3)
*Anopheles*	*Anopheles*	*balenrae (hyrcanus)*	+ (3/3)
*Anopheles*	*Anopheles*	*yatsushiroensis (hyrcanus)*	+ (3/3)
*Anopheles*	*Anopheles*	*crawfordi (hyrcanus)*	+ (3/3)
*Anopheles*	*Anopheles*	*kweiyangensis (hyrcanus)*	+ (3/3)
*Anopheles*	*Anopheles*	*junlianensis (hyrcanus)*	+ (8/8)
*Anopheles*	*Anopheles*	*peditaeniatus (hycanus)*	+ (7/7)
*Anopheles*	*Anopheles*	*kunmingensis (hyrcanus)*	−
*Anopheles*	*Anopheles*	*lindesayi*	−
*Anopheles*	*Cellia*	*stephensi*	− 2
*Anopheles*	*Cellia*	*gambiae*	− 2
*Anopheles*	*Cellia*	*dravidicus*	−
*Anopheles*	*Cellia*	*jeyporiensis*	−
*Anopheles*	*Cellia*	*philippinensis*	−
*Anopheles*	*Cellia*	*dirus*	− 3
*Anopheles*	*Cellia*	*minimus*	−
*Mansonia*	not identified	not identified	−
*Culex*		*quinquefasciatus*	− 2
*Ochlerotatus*		*caspius*	− 3
*Aedes*	*Stegomyia*	*albopictus*	− 3
*Aedes*	*Stegomyia*	*aegypti*	+

Notes

1. Presence (+) or absence (−) of *MJ1* as determined by genome analysis and/or PCR with subsequent sequencing. The numbers in brackets show the number of *MJ1* clones among the number of clones sequenced. For species that produced no PCR product with the *MJ1* primer, a positive control with ITS primers was performed, which confirmed genomic DNA integrity.

2. The absence of *MJ1* is confirmed by searching the genome assembly as well as raw trace sequences.

3. In these species, we obtained PCR products when amplifying genomic DNA using the *MJ1* primer. However, these products were of incorrect size and were shown to be artifacts as none matched *MJ1* when we sequenced 6 (for *Oc. caspius*), 10 (for *Ae. albopictus*), and 11 (for *An. dirus*) clones from these PCR products.

It is possible, although not likely, that selection pressure could contribute to the observed 99% conservation of *MJ1* sequences between *Ae aegypti* and *An. sinensis*. However, analysis of *MJ1* copies from *Ae. aegypti* and the *Anopheles* species showed *d*_N_/*d*_S_ values ranging from 0.66 to 0.78, with no evidence of strong selection pressure. We have previously calculated *d*_N_/*d*_S_ values for Vg-C, a mosquito gene known to be relatively rapidly evolving [12]. Comparisons of Vg-C genes among *Aedes* and *Anopheles* mosquitoes showed *d*_N_/*d*_S_ values ranging from 0.065 to 0.073 [12]. Therefore, the *d*_N_/*d*_S_ values from the *MJ1* comparisons suggest that the high sequence identity between the *MJ1* elements in *Ae. aegypti* and *An. sinensis* does not result from high selection pressure. Taken together, recent horizontal transfer is the only reasonable explanation of the high identity between *MJ1* in these highly divergent mosquito species.

### Phylogenetic analysis of *MJ1* sequences in *Ae. aegypti* and the hyrcanus group of *Anopheles* mosquitoes

**Figure 5:**
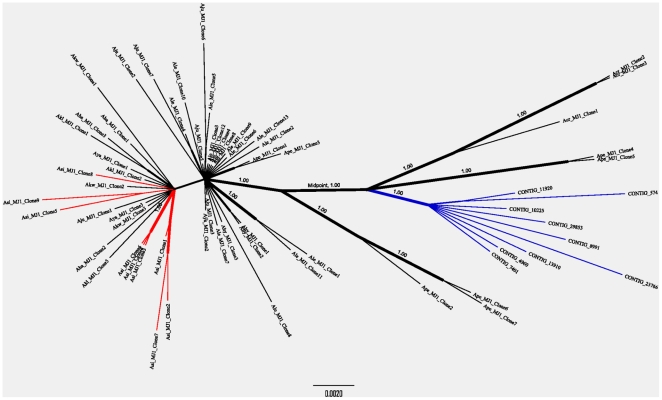
Phylogeny of the 64 *MJ1* sequences from *Ae. aegypti* and the hyrcanus group of *Anopheles* mosquitoes. The unrooted phylogeny was inferred from nucleotide sequence alignment of all 64 *MJ1* sequences using MrBayes version 3.1.2 [20]. The evolutionary model used during the Bayesian analysis was selected using JModeltest [29] and 2.5 million generations of analyses were performed to produce the phylogeny and clade credibility scores. Sequence alignment and parameters for phylogenetic analysis are provided in Supplemental File S4 which is an executable Nexus file. *Ae. aegypti MJ1* sequences are indicated by their contig names. All other *MJ1* sequences are named according to the following convention: The first letter “A” refers to genus *Anopheles* and the 2^nd^ and 3^rd^ letters are the first two letters of the species name. For example, the first clone of the *Anopheles sinensis MJ1* is Asi_MJ1_Clone1. Full species names are shown in Table 1. *Ae. aegypti MJ1* and *An. sinensis MJ1* are highlighted in blue and red, respectively. Only clades with >0.70 credibility scores are shown as resolved clades. The thickness of the corresponding branches is proportional to the credibility score. Clades with the highest possible credibility value, 1.00, are indicated. Although the tree in this figure is unrooted, the position of midpoint root is indicated.

Phylogenetic relationships of the 64 *MJ1* sequences were inferred using a Bayesian program named MrBayes [20]. Shown in Figure 5 is an unrooted phylogeny based on nucleotide sequence alignments (see Supplemental File S4 for the entire alignment and the model and parameters used for phylogenetic reconstruction). The scale bar of the tree is at 0.002 substitutions per site and the variable but overall short branch length of each *MJ1* relative to the scale bar reflects the fact that the identity levels among vast majority of these *MJ1* sequences are above 97%. All nine copies of the *Ae. aegypti MJ1* form a well supported clade (credibility score 1.00) distinct from *An. sinensis* and other *MJ1* sequences, which further argues against contamination being the explanation of the high sequence identity between *Ae. aegypti MJ1* and *Anopheles MJ1*. If midpoint rooting is applied, the nine *Ae. aegypti MJ1* sequences form a broader and well supported clade (credibility score 1.00) with two *An. peditaeniatus MJ1* (Ape_MJ1_Clone4 and Clone 5) and three *An. crawfordi MJ1* (Acr_MJ1_Clone1, Clone 2, and Clone 3). This clade, which is to the right of the midpoint in Figure 5, consists of sequences that appear to be more evolutionary divergent compared to most of the sequences that belong to the clade to the left of the midpoint. For example, the branch length of a *MJ1* sequence in the clade to the right of the midpoint is on average longer than the branch length of a *MJ1* sequence in the left clade. Moreover, while only one *MJ1* (*Ae. aegypti* Contig_13910) out of the 14 *MJ1* in the right clade contains an intact open reading frame for the transposase, 30 of the 50 *MJ1* in the left clade contain intact open reading frames (Supplemental Files S3 ). The relationship between most of the *MJ1* sequences in the left clade is not well resolved, which is expected given their short branch lengths or high sequence similarities. There are a few cases in which *MJ1* from different *Anopheles* species form a well supported clade (e.g., Ale_MJ1_Clone5 and Aju_MJ1_Clone6; Ale_MJ1_Clone4 and Aju_MJ1_Clone7; Aba_MJ1_Clone3 and Akl_MJ1_Clone1). Such relationships may reflect horizontal transfer or introgression [21] between these *Anopheles* species. Note that *An. peditaeniatus* and *An. crawfordi*, the two species that contain *MJ1* most closely-related to *Ae. aegypti MJ1*, are the basal lineages within the hyrcanus group [22], [23]. *An. peditaeniatus* also contains *MJ1* sequences that belong to the clade to the left of the midpoint. It is important to note that all *Ae. aegypti MJ1* sequences were obtained from the genome assembly while *Anopheles MJ1* were obtained by PCR, which was designed to sample *MJ1* sequences with full terminal inverted repeats. The lack of *MJ1* in *An. kumingensis* (Table 1) may reflect a loss of full-length *MJ1* because *An. kumingensis* is among the more derived lineages [22], [23] and all other hyrcanus mosquitoes including its close relative *An. kweiyangensis* harbor *MJ1*.

## Discussion

We uncovered *MJ1* transposons in *Anopheles* mosquitoes and they share up to 99% nucleotide identity with *Ae. aegypti MJ1*, even though the *Aedes* and *Anopheles* genera had diverged 145–200 million years ago. Further analyses of *MJ1* insertion sites, species distribution, and selection pressure clearly point to recent horizontal transfer as the only reasonable explanation for such a high identity between *MJ1* from these divergent species. It is not reliable to determine the divergence time of these *MJ1* sequences on the basis of substitution rates because the number of synonymous substitutions is very low between these highly similar sequences. However, a few observations can be made regarding the evolution of *MJ1* on the basis of our phylogenetic analysis. As shown in Figure 5, many *Anopheles MJ1* elements may have been recently transposed given the high sequence similarities between clones within and among different species. A few well supported clades consist of *MJ1* from different *Anopheles* species, which may either reflect horizontal transfer or introgression [21] between these species within the hyrcanus group. Our survey of *MJ1* in *Anopheles* species cannot detect copies that have truncations at any one of the termini and our survey of *MJ1* in *Aedes* has been limited to *Ae. aegypti* and *Ae. albopictus*. Nonetheless, here we discuss the timing and direction of the main horizontal transfer event between *Aedes* and *Anopheles* mosquitoes, given the current available data and with the understanding that expanded surveys in the future may support different evolutionary scenarios. It is apparent that *MJ1* from *Ae. aegypti* are more closely-related to some of the *MJ1* from the two basal species of the hyrcanus group, *An. crawfordi* and *An. peditaeniatus* [22], [23], than to other *Anopheles MJ1* sequences (Figure 5). If we accept midpoint rooting of the unrooted tree shown in Figure 5, *Ae. aegypti MJ1* form a well supported clade with some of the *MJ1* sequences from the two basal species mentioned above. Thus the most parsimonious interpretation is that *MJ1* existed in the common ancestor of the hyrcanus group and the main horizontal transfer event between *Aedes* and *Anopheles* may have occurred after the divergence between the basal lineage and the more derived species within the hyrcanus group [22], [23]. The direction of the horizontal transfer may be from the basal lineage of the hyrcanus group (the clade or subgroup that contains *An. crawfordi* and *An. peditaeniatus*) to *Aedes*. One of the alternative hypotheses, namely transfer of *MJ1* from *Aedes* to the common ancestor of the hyrcanus group, cannot explain the well-supported relationship between *Aedes MJ1* and some of the *MJ1* elements in *An. crawfordi* and *An. peditaeniatus* (Figure 5). The other alternative hypothesis, namely transfer of *MJ1* from *Aedes* to the subgroup that contains *An. crawfordi* and *An. peditaeniatus*, cannot explain the existence of *MJ1* in the more derived species such as *An. sinensis* and *An. lesteri* in the hyrcanus group. To have a better understanding of the timing and direction of the main horizontal transfer event of *MJ1* between *Aedes* and *Anopheles* mosquitoes, it is important to survey additional mosquito species, especially species that are closely related to *Ae. aegypti* and to maximize coverage of young and old *MJ1* copies in any given species. A better understanding of the phylogenetic relationship and divergence time of species within the *Anopheles* and *Aedes* genera will also be helpful to determining the timing and direction of the horizontal transfer of *MJ1* between the two genera.

The utility of low-coverage next-generation sequencing has been limited in the absence of a reference genome. However, as shown here, such an approach can readily uncover transposons that exist in multiple copies and identify transposons that may be the subject of very recent horizontal transfer events. In our case, 0.4x coverage was sufficient to identify *MJ1*, which is a low-copy element in *An. sinensis* (Figures 2 and 3). We used an earlier version of 454 GS FLX to generate the low-coverage sequences totaling 117 Mbp with an average read length of 230 bp. Currently a single illumina run can provide 1300 Mbp of sequences with 80 bp read length at a cost of $1000 or less. With the implementation of multiplexing and the rapid progress in high-throughput sequencing technology and the continuing reduction of sequencing cost, a broad survey of many species by low-coverage genomic sequencing is within reach and will allow systematic discovery of novel horizontal transfer events. For example, one could obtain low-coverage sequences of a large number of species with ecological overlap to identify repetitive sequences that show unexpectedly high identity between species. Such analysis will lead to candidates of very recent horizontal transfer events, which will likely offer opportunities to investigate the mechanisms and circumstances of horizontal transfer because the factors required for such lateral transfer may still be accessible for examination [3]. Broad low-coverage genomic surveys will also facilitate systematic investigations of the condition and frequency of horizontal transfer events, which has been difficult to study. It is important to note that the role of low-coverage sequencing here is to lead to the discovery of horizontal transfer events, which need to be confirmed by further analysis as shown in this study.

Our discovery also has important practical implications. The existence of *MJ1* copies with intact open reading frames in most species and the presence of highly similar copies within and between species suggest that *MJ1* may still be active, or an active copy may be constructed. Transformation of mosquitoes has been achieved using exogenous transposons. However, relatively low efficiency and lack of remoblization of these transposons in the mosquito germline hinders genetic manipulations for basic research and for exploring new disease control strategies [24]. *MJ1* is a candidate for a new transformation tool that may overcome some of these limitations.

Horizontal transfer has been a long-standing concern associated with a novel strategy to combat mosquito-borne infectious diseases by spreading transgenes that confer resistance to pathogens into mosquito populations [25]. The risk of transfer of an introduced transposon and/or associated transgene to unintended organisms has been difficult to evaluate. The discovery of recent horizontal transfer between mosquito species offers a starting point to investigate the conditions under which horizontal transfer occurs. Future applications of low-coverage next-generation sequencing to a wide range of mosquito species will allow for estimation of the frequency of horizontal transfer events and provide a quantitative basis for risk assessment.

## Materials and Methods

### 454 sequencing of *An. sinensis* and initial sequence analysis

The Shanghai strain of *An. sinensis* (National Institute for Parasitic Diseases, Chinese Center for Disease Control and Prevention, Shanghai, China) was used. Genomic DNA was extracted from approximately 500 adult mosquitoes. Subsequent sequencing steps involved in 454 GS FLX sequencing were all performed at the Virginia Bioinformatics Institute at Virginia Tech. Briefly, approximately 10 µg of genomic DNA were fractionated into 300 to 800 bp fragments, to which short adaptors specific for both the 3′ and 5′ ends were added by ligation. The adaptors enable individual genomic DNA fragment to bind a unique bead and get amplified by PCR. The clonally amplified beads, each representing a unique genomic DNA fragment, are used as templates for sequencing. Slightly more than 500,000 sequencing reads passed quality filtering and the average and range of sequence lengths are 230 bp, and 100–300 bp, respectively. A total 117 Mbp (0.4x genome coverage) shotgun sequences were obtained. This shotgun database was formatted for BLASTn analysis on a 2x quad-core Linux server with 32 GB of RAM using all known *Ae. aegypti* TEs [17] as query with an e-value cut-off of 1e-5.

### Amplification, cloning, and sequencing of MJ1

For confirmation of *MJ1* in *An. sinensis*, we used *An. sinensis* mosquitoes from two independent sources and carried out subsequent analysis at two different institutions. The first was the Shanghai strain, which was analyzed at the Chinese Academy of Sciences in Shanghai and the second was the Guangdong strain, which was analyzed at Virginia Tech. Adult mosquitoes were homogenized and DNA was extracted by ethanol precipitation and resuspended in 50 µl double-distilled water. Full length *MJ1* was obtained using polymerase chain reaction (PCR). PCR was carried out with 1 µl genomic DNA as the template and the *Aae_MJ1* terminal inverted repeat as the sole primer (5′-TACACGGTGTTCAATAAGTTC-3′). Either 1 unit of *Taq* plus or *Pfu* enzyme was used in a 20 µl reaction. PCR amplification was performed for 30 cycles (50 s denaturation at 94°C, 30 s of annealing at 53°C, and 1.5 min extension at 72°C). PCR products were gel purified, cloned, and multiple clones were sequenced. The same method was used for analysis of other species. As a common practice during all PCR analysis, negative controls with no genomic templates were included and were negative. Additional measures were taken to minimize contamination or false positive results, which included the use of aerosol filter tips during PCR setup, the use of fresh electrophoresis buffer every time when a gel was run, and the use of new cutters for cutting bands every time. All *MJ1* copies were confirmed by sequencing and special attention was also paid to minimize false negative results during the species survey. PCR with ITS_2_ primers was performed as positive controls to confirm genomic DNA integrity. The PCR condition for *MJ1* amplification, as described above, allows for amplification of sequences with mismatches to the *MJ1* terminal inverted repeat primer. Indeed PCR products from other *IS630-Tc1-mariner* transposons were sometimes obtained but subsequently determined not to be *MJ1* by sequencing.

### Fluorescence *in situ* hybridization

The fourth instars of *An. sinensis* (Shanghai strain) were preserved in Carnoy's solution (Methanol: Glacial Acetic acid  = 3∶1). Polytene chromosomes were prepared from salivary glands. PCR products of an *Asi_MJ1* transposon with confirmed sequence were labeled with Cy3 and Cy5-AP3-dUTP (GE Healthcare UK Ltd, Buckinghamshire, England) by Random Primers DNA Labeling System (Invitrogen Corporation, Carlsbad, CA, USA). The DNA probes were hybridized to the chromosomes at 39°C overnight in 2x hybridization buffer (Invitrogen Corporation, Carlsbad, CA, USA). The chromosome preparations were washed in 0.2XSSC, counterstained with YOYO-1, and mounted in DABCO. Under these conditions, the probe would need to be >85% identical to the target to produce a signal. Fluorescent signals were detected and recorded using a Zeiss LSM 510 Laser Scanning Microscope (Carl Zeiss MicroImaging, Inc., Thornwood, NY, USA). Localization of signal was accomplished using a cytogenetic map for *An. sinensis* [26].

### TE-display and sequencing of DNA recovered from TE-display

TE-display was performed as previously described [19]. Briefly, genomic DNA from individual mosquitoes was digested using BfaI. The digested DNA fragments were ligated to an adapter. Two rounds of PCR were used to amplify the fragments between specific *MJ1* sequences and the adapter sequence. A γ-^33^P labeled nested primer was used in the second round of PCR. The amplified fragments were separated on a sequencing gel. The sequences for the BfaI-adapter were 5′-GACGATGAGTCCTGAG-3′ and 5′-TACTCAGGACTCAT-3′. We used two sets of *MJ1*-specific primers which gave similar results. The first set of the *MJ1*-specific primers are: 5′-ACAAACTCCTGACCAGCGTG-3′ and 5′-GATTGAGCGGTTCTTTTTGC-3′. The second set of *MJ1*-specific primers are: 5′-GATTGAGCGGTTCTTTTTGC-3′ and 5′-CATTGGTCGAGGACGTCTCC-3′. Bands from the TE-display gel were purified, amplified by PCR, cloned, and sequenced.

### Computational and phylogenetic analysis

BLAST analysis between different *MJ1* sequences was carried out locally on a 2x quad-core Linux server. Multiple sequence alignment was done using Clustalw (http://www.ebi.ac.uk/Tools/clustalw2/, gap opening penalty = 10 and gap extension penalty = 0.05). Consensus was made using CONSENSUS (http://www.hiv.lanl.gov/content/sequence/CONSENSUS/consensus.html). *d*_N_/*d*_s_ analysis was performed using SNAP (www.hiv.lanl.gov) [27], [28]. Phylogeny of the 64 *MJ1* sequences from *Ae. aegypti* and the hyrcanus group of *Anopheles* mosquitoes was inferred using MrBayes version 3.1.2 [20] on the nucleotide sequence alignment. Based on the JModeltest [29] analysis of the alignment, Kimura unequal base frequency model [30] with rate variation among sites (gamma shape = 2.9370) was selected for MrBayes analysis. Two and a half million generations were run to generate phylogeny and clade credibility scores. Visualization and presentation of the tree is carried out using Figtree (http://tree.bio.ed.ac.uk/software/figtree/). Sequence alignment and parameters for phylogenetic analysis are provided in Supplemental File S4 which is an executable Nexus file.

### Sequence Deposition

All 55 *Anopheles MJ1* sequences described in this manuscript are submitted to GenBank (accession numbers HQ334205-HQ334259) and are shown in Supplemental File S2.

## Supporting Information

File S1 Nucleotide Sequences of the consensus and the nine copies of *MJ1* in *Aedes aegypti*.
(DOCX)

File S2 *MJ1* sequences from the hyrcanus group of *Anopheles* mosquitoes.
(DOCX)

File S3 Peptide sequences of the transposase encoded by Aae_MJ1_CONCENSUS and all individual *MJ1* copies that had intact open reading frames.
(DOCX)

File S4 Alignment of 64 *MJ1* sequences and the parameters/model used for phylogenetic analysis.
(DOCX)
