# Supplementary material for: Next-Generation Sequencing Reveals Recent Horizontal Transfer of a DNA Transposon between Divergent Mosquitoes
Source: PLoS One. 2011 Feb 10;6(2):e16743. doi: 10.1371/journal.pone.0016743 (PMC3037385; doi:10.1371/journal.pone.0016743)
Supplement: File S1 — Nucleotide Sequences of the consensus and the nine copies of MJ1 in Aedes aegypti. (DOCX) [file pone.0016743.s001.docx]

**Supplemental File 1**

**Nucleotide Sequences of the consensus and the nine copies of *MJ1* in *Aedes aegypti***

1A. Consensus nucleotide sequence derived from the 9 copies of MJ1 found in the *Aedes aegypti* genome assembly.

>Aae_MJ1_CONCENSUS

CACGGTGTTCAATAAGTTCGAATACAAGTTTTCATCATTGcGTAGGTATGCGCCATGTACATATTCTGCATTGGTATTGGTGTCAGCTTTAGCTTCATTCATACGCTACcGAATGTGCgCGGTGTTGACATTcTGTTAGTTGTTGTTCGTTTGTTACGcGCGATGAAAGAGTATCGGgACTTcGTAATTAAGCGTTTTTTGAACGGTGAGCGACCCGGCGATATATTcCGGCTGCTGAAATCGCATGGGGTCAAGCGGAaCTTTGTCTaCACGACCATCAGGCgATACcGGGAGACGTCCTCGACCAATGACCGTGCGAGATCCGGTCGGCCGCGTTCAGCGAGGACGCCACGGGTCAtCAAgaTcgTGAGGGAGCGAaTTCGGCGCAAAAaGAACCgCTCAATCcggaaaacggctgcagatctcaacgtttccattggaaccgctcacaccatactcatcaaggaccttggtttcaggccttacaaaaaacgtaaggtccatggcgtttcGGAGGCTACCAGCAAAAAGCGGTTGgATCGAGCTAAGAGGATCCTCTCTCGGCACGCTGgTCAGGAGTTTGTTTTTTCGGACGAGAAACTGTTCGTGCTGCAGCAGCCGCACAATGTgCAAAATGACCGGGTGTGGGCGCCATCGAGGGACAGCATTCCTGAATCCAATATAAACATCCCTcGGTTCCAAAGTGCCGCGTCgGTGATGGTTTGGGGGGCAGTATGCAAACGTGGTAAGCTACCCTTGGTGTTTATTGAAAAAAaCGTCAAAATCAACGcGGCGTACtACAAAACTgAGGTTTTGGAAAAGGTTGTTGcCcCCAGTCTCCGaagcctctacggcgatgagcactacgtgttccagcaggacggtgcaccagcccatacgGCAAATGTgGTTcAAGCCTGGTGTCGGGAcAATTTAACCgAcTTTCTGGACAAAACTTTGTGGCCTCCCAGCTCCCCGGACTTGAATCCTCTCGAcTTTTTTGTTTGGTCCTATATGATGgCGAAGCTGAACGAATACAAgGTCAGCACTTTGGACCATTTCAAGACgGTAATTCTCAAAATCTgggaCgAAatgcccatgcagtccgtgcgtgccgcttgcgacgcgTTCGAGAAACGTTTGAAGCTCGTTAAGGAGTACAAAGGgGGGGTCATtCCAAGAgAAATGTTGTaAACGTTCCTTGTAaacatagctttcaataacataaatccaaaaaataaaaaaacatgttttcatttttttaacaaattttgaaagtgtatccgaacttattgaacaccgtg

1B. Individual Sequence of the 9 MJ1 copies found in *Aedes aegypti*. The two numbers after the contig name are the start and end positions of the MJ1 copy. Note that the TA target duplications were removed.

>CONTIG_10225_23341_24669

CACGGTGTTCAATAAGTTCGAATACAAGTTTTCATCATTGCGTAGGTATGCGCCATGTACATATTCTGCATTGGTATTGGTGTCAGCTTTAGCTTCATTCATACGCTACCGAATGTGCGCGGTGTTGACATTCTGTTAGTTGTTGTTCGTTTGTTACGCGCGATGAAAGAGTATCGGGACTTCGTAATTAAGCGTTTTTTGAACGGTGAGCGACCCGGCGATATATTCCGGCTGCTGAAATCGCATGGGGTCAAGCGGAACTTTGTCTACACGACCATCAGGCGATACCGGGAGACGTCCTCGACCAATGACCGTGCGAGATCCGGTCGGCCGCGTTCAGCGAGGACGCCACGGGTCATCAAGATCTTGAGGGAGCGAATTCGGCGCAAAAAGAACCGCTCAATCCGGAAAACGGCTGCAGATCTCAACGTTTCCATTGGAACCGCTCACACCATACTCATCAAGGACCTTGGTTTCAGGCCTTACAAAAAACGTAAGGTCCATGGCGTTTCGGAGGCTACCAGCAAAAAGCGGTTGGATCGAGCTAAGAGGATCCTCTCTCGGCACGCTGGTCAGGAGTTTGTTTTTTCGGACGAGAAACTGTTCGTGCTGCAGCAGCCGCACAATGTGCAAAATGACCGGGTGTGGGCGCCATCGAGGGACAGCATTCCTGAATCCAATATAAACATCCCTTGGTTCCAAAGTGCCGCGTCGGTGATGGTTTGGGGGGCAGTATGCAAACGTGGTAAGCTACCCTTGGTGTTTATTGAAAAAAAACGTCAAAATCAACGCGGCGTACAACAAAACTGAGGTTTTGGAAAAGGTTGTTGCCCCCAGTCTCCGAAGCCTCTACGGCGATGAGCACTACGTGTTCCAGCAGGACGGTGCACCAGCCCATACGGCAAATGTGGTTTAAGCCTGGTGTCGGGACAATTTAACCGACTTTCTGGACAAAACTTTGTGGCCTCCCAGCTCCCCGGACTTGAATCCTCTCGACTTTTTTGTTTGGTCCTATATGATGGCGAAGCTGAACGAATACAAGGTCAGCACTTTGGACCATTTCAAGACGGTAATTCTCAAAATCTGGGACGAAATGCCCATGCAGTCCGTGCGTGCCGCTTGCGACGCGTTCGAGAAACGTTTGAAGCTCGTTAAGGAGTACAAAGGGGGGGTCATTCCAAGAGAAATGTTGTAAACGTTCCTTGTAAACATAGCTTTCAATAACATAAATCCAAAAAATAAAAAAACATGTTTTCATTTTTTTAACAAATTTTGAAAGTGTATCCGAACTTATTGAACACCGTG

>CONTIG_11920_201140_2023

CACGGTGTTCAATAAGTTCGAATACAAGTTTTCATCATTGCGTAGGTATGCGCCATGTACATATTCTGCATTGGTATTGGTGTCAGCTTTAGCTTCATTCATACGCTACAGAATGTGCGCGGTGTTGACATTCTGTTAGTTGTTGTTCGTTTGTTACGCGCGATGAAAGAGTATCGGGACTTCGTAATTAAGCGTTTTTTGAACGGTGAGCGACCCGGCGATATATTCCGGCTGCTGAAATCGCATGGGGTCAAGCGGAACTTTGTCTACACGACCATCAGGCGATACCGGGAGACGTCCTCGACCAATGACCGTGCGAGATCCGGTCGGCCGCGTTCAGCGAGGACGCCACGGGTCATCAAGATCGTGAGGGAGCGAATTCGGCGCAAAAAGAACCGCTCAATCTGGAAAACGGCTGCAGATCTCAACGTTTCCATTGGAACCGCTCACACCATACTCATCAAGGACCTTGGTTTCAGGCCTTACAAAAAACGTAAGGTCCATGGCGTTTCGGAGGCTACCAGCAAAAAGCGGTTGGATCGAGCTAAGAGGATCCTCTCTCGGCACGCTGGTCAGGAGTTTGTTTTTTTCGGACGAGAAACTGTTCGTGCTGCAGCAGCCGCACAATGTGCAAAATGACCGGGTGTGGGCGCCATCGAGGGACAGCATTCCTGAATCCAATATAAACATCCCTCGGTTCCAAAGTGCCGCGTCGGTGATGGTTTGGGGGGCAGTATGCAAACGTGGTAAGCTACCCTTGGTGTTTATTGAAAAAACGTCAAAATCAACGCGGCGTACTACAAAACTGAGGTTTTGGAAAAGGTTGTTGCCCCCAGTCTCCGAAGCCTCTACGGCGATGAGCACTACGTGTTCCAGCAGGACGGTGCACCAGCCCATACGGCAAATGTGGTTCAAGCCTGGTGTCGGGACAATTTAACCGATTTTCTGGACAAAACTTTGTGGCCTCCCAGCTCCCCGGACTTGAATCCTCTCGACTTTTTTGTTTGGTCCTATATGATGGCGAAGCTGAACGAATACAAGGTCAGCACTTTGGACCATTTCAAGACGGTAATTCTCAAAATCTGGGACGAAATGCCCATGCAGTCCGTGCGTGCCGCTTGCGACGCGTTCGAGAAACGTTTGAAGCTCGTTAAGGAGTACAAAGGGGGGGTCATTCCAAGAGAAATGTTGTAAACGTTCCTTG

>CONTIG_13910_402130_403457

CACGGTGTTCAATAAGTTCGAATACAAGTTTTCATCATTGCGTAGGTATGCGCCATGTACATATTCTGCATTGGTATTGGTGTCAGCTTTAGCTTCATTCATACGCTACCGAATGTGCGCGGTGTTGACATTTTGTTAGTTGTTGTTCGTTTGTTACGCGCGATGAAAGAGTATCGGGACTTCGTAATTAAGCGTTTTTTGAACGGTGAGCGACCCGGCGATATATTCCGGCTGCTGAAATCGCATGGGGTCAAGCGGAACTTTGTCTGCACGACCATCAGGCGATACCGGGAGACGTCCTCGACCAATGACCGTGCGAGATCCGGTCGGCCGCGTTCAGCGAGGACGCCACGGGTCATCAAGATCGTGAGGGAGCGAATTCGGCGCAAAAAGAACCGCTCAATCCGGAAAACGGCTGCAGATCTCAACGTTTCCATTGGAACCGCTCACACCATACTCATCAAGGACCTTGGTTTCAGGCCTTACAAAAAACGTAAGGTCCATGGCGTTTCGGAGGCTACCAGCAAAAAGCGGTTGGATCGAGCTAAGAGGATCCTCTCTCGGCACGCTGGTCAGGAGTTTGTTTTTTCGGACGAGAAACTGTTCGTGCTGCAGCAGCCGCACAATGTGCAAAATGACCGGGTGTGGGCGCCATCGAGGGACAGCATTCCTGAATCCAATATAAACATCCCTCGGTTCCAAAGTGCCGCGTCGGTGATGGTTTGGGGGGCAGTATGCAAACGTGGTAAGCTACCCTTGGTGTTTATTGAAAAAAACGTCAAAATCAACGCGGCGTACTACAAAACTGAGGTTTTGGAAAAGGTTGTTGCCTCCAGTCTCCGAAGCCTCTACGGCGATGAGCACTACGTGTTCCAGCAGGACGGTGCACCAGCCCATACGGCAAATGTGGTTCAAGCCTGGTGTCGGGACAATTTAACCGACTTTCTGGACAAAACTTTGTGGCCTCCCAGCTCCCCGGACTTGAATCCTCTCGACTTTTTTGTTTGGTCCTATATGATGGCGAAGCTGAACGAATACAATGTCAGCACTTTGGACCATTTCAAGACAGTAATTCTCAAAATCTGGGACGAAATGCCCATGCAGTCCGTGCGTGCCGCTTGCGACGCGTTCGAGAAACGTTTGAAGCTCGTTAAGGAGTACAAAGGAGGGGTCATTCCAAGAGAAATGTTGTAAACGTTCCTTGTAAACATAGCTTTCAATAACATAAATCCAAAAAATAAAAAAACATGTTTTCATTTTTTTAACAAATTTTGAAAGTGTATCCGAACTTATTGAACACCGTG

>CONTIG_23766_524_1855

CACGGTGTTCAATAAGTTCGAATACAAGTTTTCATCATTGCGTAGGTATGCGCCATGTACATATTCTGCATTGGTATTGGTGTCAGCTTTAGCTTCATTCATACGCTACCGAATGTGCGCGGTGTTGACATTCTGTTAGTTGTTGTTCGTTTGTTACGCGCGATGAAAGAGTATCGGGACTTCGTAATTAAGCGTTTTTTGAACGGTGAGCGACCCGGCGATATATTCCGGCTGCTGAAATCGCATGGGGTCAAGCGGAACTTTGTCTACACGACCATCAGGCGATACCGGGAGACGTCCTCGACCAATGACCGTGCGAGATCCGGTCGGCCGCGTTCAGCGAGGACGCCACGGGTCACCAAAGTTGTGAGGGAGCGATTCGGACGCAAAAGAACCGCTCAATCCGGAAAACGGCTGCAGATCTCAACGTTTCCATAGGAACCGCTCACACCATACTCATCAAGGACCTTGGTTTCAGGCCTTACAAAAAACGTAAGGTCCATGGCGTTTCGGAGGCTACCAGCAAAAAGCGGTTGGATCGAGCTAAGAGGATCCTCTCTCGGCACGCTGGTCAGGAGTTTGTTTTTTCGGACGAGAAACTGTTCGTGCTGCAGCAGCCGCACAATGTGCAAAATGACCGGGTGTGGGCGCCATCGAGGGACAGCATTCCTGAATCCAATATAAACATCCCTCGGTTCCAAAGTGCCGCGTCGGTGATGGTTTGGGGGGCAGTATGCAAACGTGGTAAGCTACCCTTGGTGTTTATTGAAAAAAAAACGTCAAAATCAACGCGGCGTACTACAAAACTGAGGTTTTGGAAAAGGTTGTTGCCCCCAGTCTCCGAAGCCTCTACGGCGATGAGCACTACGTGTTCCAGCAGGACGGTGCACCAGCCCATACGGCAAATGTGGTTCAAGCCTGGTGTCGGGACAATTTAACCAACTTTCTGGACAAAACTTTGTGGCCTCCCAGCTCCCCGGACTTGAATCCTCTCGACTTTTTTGTTTGGTCCTATATGATGGCGAAGCTGAACGAATACAAGGTCAGCACTTTGGACCATTTCAAGACGGTAATTCTCAAAATCTGGGACGAAATGCCCATGCAGTCCGTGCGTGCCGCTTGCGACGCGTTCGAGAAACGTTTGAAGCTCGTTAAGGAGTACAAAGGGGGGGGTCATTCCAAGAGAAATGTTGTAAACGTTCCTTGTAAACATAGCTTTCAATAACATAATTCCAAAAAATAAAAAAAACATGTTTTCATTTTTTTTAACAAATTTTGAAAGTGTATCCGAACTTACTGAACACCGTG

>CONTIG_29853_42851_44071

CACGGTGTTCAATAAGTTCGAATACAAGTTTTCATCATTGGGTAGGTATGCGCCATGTACATATTCTGCATTGGTATTGGTGTCAGCTTTAGCTTCATTCATACGCTACCGAATGTGCGCGGTGTTGACATTCTGTTAGTTGTTGTTCGTTTGTTACGTGCGATGAAAGAGTATCGGGACTTGGTAATTAAGCGTTTTTTGAACGGTGAGCGACCCGGCGATATATTCCGGCTGCTGAAATCGCATGGGGTCAAGCGGAACTTTGTCTACACGACCATCAGGCGATACCGGGAGACGTCCTCGACCAATGACCGTGCGAGATCCGGTCGGCCGCGTTCAGCGAGGACGCCACGGGTCATCAAGATCGTGAGGGAGCGAATTCGGCGCAAAAAGAACCCCTCAATCGGAGGCTACCAGCAAAAAGCGGTTGGATCGAGCTAAGAGGATCCTCTCTCGGCACGCTGGTCAGGAGTTTGTTTTTTCGGACGAGAAACTGTTCGTGCTGCAGCAGCCGCACAATGTGCAAAATGACCGGGTGTGGGCGCCATCGAGGGACAGCATTCCTGAATCCAATATAAACATCCCTCGGTTCCAAAGTGCCGCGTCGGTGATGGTTTGGGGGGCAGTATGCAAACGTGGTAAGCTACCCTTGGTGTTTATTGAAAAAAACGTCAAAATCAACGTGGCGTACTACAAAACTGAGGTTTTGGAAAAGGTTGTTGCCCCCAGTCTCCGAAGCCTCTACGGCGATGAGCACTACGTGTTCCAGCAGGACGGTGCACCAGCCCATACGGCAAATGTGGTTCAAGCCTGGTGTCGGGACAATTTAACCGACTTTCTGGACAAAACTTTGTGGCCTCCCAGCTCCCCGGACTTGAATCCTCTCGACTTTTTTGTTTGGTCCTATATGATGACGAAGCTGAACGAATACAAGGTCAGCACTTTGGACCATTTCAAGACGGTAATTCTCAAAATCTGGGACGAAATGCCCATGCAGTCCGTGCGTGCCGCTTGCGACGCGTTCGAGAAACGTTTGAAGCTCGTTAAGGAGTACAAAGGGGGGGTCATTCCAAGAGAAATGTTGTAAACGTTCCTTGTAAACATAGCTTTCAATAACATAAATCCAAAAAATAAAAAAACATGTTTTCATTTTTTTAACAAATTTTGAAAGTGTATCCGAACTTATTGAACACCGTG

>CONTIG_4960_22771_24044

CACGGTGTTCAATAAGTTCGAATACAAGTTTTCATCATTGCGTAGGTATGCGCCATGTACATATTCTGCATTGGTATTGGTGTCAGCTTTAGCTTCATTCATACGCTACCGAATGTGCGCGGTGTTGACATTCTGTTAGTTGTTGTTCGTTTGTTACGCGCGATGAAAGAGTATCGGGACTTCGTAATTAAGCGTTTTTTGAACGGTGAGCGACCCGGCGATATATTCCGGCTGCTGAAATCGCATGGGGTCAAGCGGATCTTTGTCTACACGACCATCAGGCAATACCGGGAGACGTCCTCGACCAATGACCGTGCGAGATCCGGTCGGCCGCGTTCAGCGAGGACGCCACGGGTCATCAAGATCGTGAGGGAGCGAATTCGGCGCAAAAAGAACCGCTCAATCCGGAAAACGGCTGCAGATCTCAACGTTTCCATTGGAACCGCTCACACCATACTCATCAAGGACCTTGGTTTCAGGCCTTACAAAAAACGTAAGGTCCATGGCGTTTCGGAGGCTACCAGCAAAAAGCGGTTGGATCGAGCTAAGAGGATCCTCTCTCGGCACGCTGGTCAGGAGTTTGTTTTTTCGGACGAGAAACTGTTCGTGCTGCAGCAGCCGCACAATGTGCAAAATGACCGGGTGTGGGCGCCATCGAGGGACAGCATTCCTGAATCCAATATAAACATCCCTCGGTTCCAAAGTGCCGCGTCGGTGATGGTTTGGGGGGCAGTATGCAAACGTGGTAAGCTACCCTTGGTGTTTATTGAAAAAAACGTCAAAATCAACGCGGCGTACTACAAAACTGAGGTTTTGGAAAAGGTTGTTGCCCCCAGTCTCCGGCAAATGTAGTTCAAGCCTGGTGTCGGGACAATTTAACCGACTTTCTGGACAAAACTTTGTGGCCTCCCAGCTCCCCGGACTTGAATCCTCTCGACTTTTTTGTTTGGTCCTATATGATGGCGAAGCTGAACGAATACAAGGTCAGCACTTTGGACCATTTCAAGACGGTAATTCTCAAAATCTGGGACGAAATGCCCATGCAGTCCGTGCGTGCCGCTTGCGACGCGTTCGAGAAACGTTTGAAGCTCGTTAAGGAGTACAAAGGGGGGGGGGGTCATTCCAAGAGAAATGTTGTGAACGTTCCTTGTAAACATAGCTTTCAATAACATAAATCCAAAAAATAAAAAAACATGTTTTCATTTTTTTAACAAATTTTGAAAGTGTATCCGAACTTATTGAACACCGTG

>CONTIG_574_74511_75800

CACGGTGTTCAATAAGTTCGAATACAAGTTTTCATCATTGCGTAGGTATGCGCCATGTACATATTCTGCATTGGTATTGGTGTCAGCTTTAGCTTCATTCATACGCTACCGAATGTGCGCGGTGTTGACATTCTGTTAGTTGTTGTTCGTTTGTTACGCGCGATGAAAGAGTATCGGAACTTCGTAATTAAGCGTTTTTTGAACGGTGAGCGACCCGGCGATATATTCCGGCTGCTGAAATCGCATGGGGTCAAGCGGAACTTTGTCTACACGACCATCAGGCGATACTGGGAGACGTCCTCGACCAATGACCGTGCGAGATCCGGTCGGCCGCGTTCAGCGAGGACGCCACGGGTCATCAAGATCGTGAGGGAGCGAATTCGGCGCAAAAAGAACCGCTCAATCTGGAAAACGGCTGCAGATCTCAACGTTTCCATTGGAACCGCTCACACCATACTCATCAAGGACCTTGGTTTCAGGCCTTACAAAAAACGTAAGATCCATGGCGTTTCGGAGGCTACCAGCAAAAAGCGGTTGGATCGAGCTAAGAGGATCCTCTCTCGGCACGCTGATCAGGAGTTTGTTTTTTCGGACGAGAAACTGTTCGTGCTGCAGCAGCCGCACAATGTACAAAATGACCGGGTGTGGGCGCCATCGAGGGACAGCATTCCTGAATCCAATATAAACATCCCTCGGTTCCAAAGTGCCGCGTCAGTGATGGTTTGGGGGGCAGTATGCAAACGTGGTAAGCTACCCTTGGTGTTTATTGAAAAAAACGTCAAAATCAACGCGGCGTACTACAAAACTGAGGTTTTGGAAAAGGTTGTTGCCCCCAGTCTCCGAAGCCTCTACGGCGATGAGCACTGCGTGTTCCAGCAGGACGGTGCACCAGCCCATACGGCAAATGTGGTTCAAGCCTGGTGTCGGGACAATTTAACCGACTTTCTGGACAAAACTTTGTGGCCTCCCAGCTCCCCGGACTTGAATCCTCTCGACTTTTTTGTTTGGTCCTATATGATGGCGAAGCTGAACGAATACAAGGTCAGCACTTTGGACCATTTCAAGACGGTAATTCTCAAAATCTCAAATTCGAGAAACGTTTGAAGCTCGTTAAGGAGTACAAAGGGGGGGGTCATTCCAAGAGAAATGTTGTAAACGTTCCTTGTAAACATAGCTTTTAATAACATAAATCCAAAAAATAAAAAAAACATGTTTTCATTTTTTTAACAAATTTTGAAAGTGTATCCGAACTTATTGAACACCGTG

>CONTIG_7401_80269_81963

CACGGTGTTCAATAAGTTCGAATACAAGTTTTCATCATTGCGTAGGTATGCGCCATGTACATATTCTGCATTGGTATTGGTGTCAGCTTTAGCTTCATTCATACGCTACCGAATGTGCACGGTGTTGACATTCTGTTAGTTGTTGTTCGTTTGTTACGCGCGATGAAAGAGTATCGGGACTTCGTAATAAGCCATTTTATAAGACGTTTCAGGTCATATGTTTTTTGTTTGTTTACCAGCTTTGAAACTTTCTGGCGGGCCCATCTATTTACGTTTCTTCTAGCGCCACATTTGGGAGGAGCTAATTCATTAATGGCATGCAACAAGCTTCTCAATCTTCCCCAGTAAAATTAAAATTAACAGGCAGGGAAGAATTATTTATTTTTGCTACTGGTCAGATATGTAACCGCGTAGAAAAATAGCCAGAAAGAGACGAAAACGTCATCATCAACAAAAATGTTACCAGCGCCATTCACATATCATGCTAGTTTTTAAAACAATAACAATGAGCGGCCCTCCAGAAAACGTCAACCGAATGTCTCGTAAAATGGCTTATTAAGCGTTTTTTGAACGGTGAGCGACCCGGCGATATATTTCGGCTGCTGAAATCGCATGGGGTCAAGCGGAACTTTGTCTACACGACCATCAGGCGATACCGGGAGACGTCCTCGACCAATGACCGTGCGAGATCCGGTCGGCCGCGTTCAGCGAGGACGCCACGGGTCATCAAGATCGTGAGGGAGCGAATTCGGCGCAAAAAGAACCGCTCAATCCGGAAAACGGCTGCAGATCTCAACGTTTCCATTGGAACCGCTCACACCATACTCATCAAGGACCTTGGTTTCAGGCCTTACAAAAAACGTAAGGTCCATGGCGTTTCGGAGGCTACCAGCAAAAAGCGGTTGGATCGAGCTAAGAGGATCCTCTCTCGGCACGCTGGTCAGGAGTTTGTTTTTTCGGACGAGAAACTGTTCGTGCTGCAGCAGCCGCACAATGTGCAAAATGACCGGGTGTGGGCGCCATCGAGGGACAGCATTCCTGAATCCAATATAAACATCCCTCGGTTCCAAAGTGCCGCGTCGGTGATGGTTTGGGGGGCAGTATGCAAACGTGGTAAGCTACCCTTGGTGTTTATTGAAAAAAACGTCAAAATCAACGCGGCGTACTACAAAACTGAGGTTTTGGAAAAGGTTGTTGTCCCCAGTCTCCGAAGCCTCTACGGCGATGAGCACTACGTGTTCCAGCAGGACGGTGCACCAGCCCATACGGCAAATGTGGTTCAAGCCTGGTGTCGGGAAAATTTAACCGACTTTCTGGACAAAACTTTGTGGCCTCCCAGCTCCCCGGACTTGAATCCTCTCGACTTTTTTGTTTGGTCCTATATGATGGCGAAGCTGAACGAATACAAGGTCAGCACTTTGGACCATTTCAAGACGGTAATTCTCAAAATCTGGGACGAAATGCCCATGCAGTCCGTGCGTGCCGCTTGCGACGCGTTCGAGAAACGTTTGAAGCTCGTTAAGGAGTACAAAGGGGGGGTCATTCCAAGAGAAATGTTGTAAACGTTCCTTGTAAACATAGCTTTCAATAACATAAATCCAAAAAATAAAAAAACATGTTTTCATTTTTTAACAAATTTTGAAAGTGTATCCGAACTTATTGAACACCGTG

>CONTIG_8991_31520_33053

CACGGTGTTCAATAAGTTCGAATACAAGTTTTCATCATTGCGTAGGTATGCGCCATGTACATATTCTGCATTGGTATTGGTGTCAGCTTTAGCTTCATTCATACGCTACCGAATGTGCGCGGTGTTGACATTCTGTTAGTTGTTGTTCGTTTGTTACGCGCGATGAAAGAGTATCGGGACTTCGTAATTAAGCGTTTTTTGAACGGTGAGCGACCCGGCGATATATTCCGGCTGCTGAAATCGCATGGGGTCAAGCGGAACTTTGTCTACACGACCATCAGGCGATACCGGGAGACGTCCTCGACCAATGACCGTGCGAGATCCGGTCGGCCGCGTTCAGCGAGGACGCCACGGGTCATCAAGATCGTGAGGGAGCGAATTCGGCGCAAAAAGAACCGCTCAATCCGGAAAACGGCTGCAGATCTCAACGTTTCCATTGGAACCGCTCACACCATACTCATCAAGGACCTTGGTTTCAGGCCTTACAAAAAACGTAAGGTCCATGGCGTTTCGGAGGCTACCAGCAAAAAGCGGTTGAATCGAGCTAAGAGGATCCTCTCTCGGCACGCTGGTCAGGAGTTTGTTTTTTCGGACGAGAAACTGTTCGTGCTGCAGCAGCCGCACAATGTGCAAAATGACCGGGTGTGGGCGCCATCGAGGGACAGCATTCCTGAATCCAATATAAACATCCCTCGGTTCCAAAGTGCCGCGTCGGTGATGGTTTGGGGGGCAGTATGCAAACGTGGTAAGCTACCCTTGGTGTTTATTGAAAAAAACGTCAAAATCAACGCGGCGTACTACAAAACTCAGGTTTTGGAAAAGGTTGTTGCCCCCAGTCTCCGAAGCATCTACGGCGATGAGCACTACGTGTTCCAGCAGGACGGTGCACCAGCCCATACGGCAAATGTGGTTCAAGCCTGGTGTCGGGACAATTTAACCGACTTTCTGGACAAAACTTTGTGGCCTCCCAGCTCCCCGGACTTGAATCCTCTCGAATTTTTTGTTTGGTCCTATATGATGGCGAAGCTGAACGAATACAAGGTCAGCACTTTGGACCATTTCAAGACGGTAATTCTCAAAATCTAGGACGAAATGCCCATGCAGTCCGTGCGTGCCGCTTGCGACGCGTTCGAGAAACGTTTGAAGCTCGTTAAGGTGATTATAGAACGAAGCCATACCTCAAATTTTGAAGAGCACAAGACTTGAGAACCAAACAGCGCTTCGTGTTGAAAATCTATCCCATTGGTCACCACCAGCAAGCAAGCAATTTGATTGGTTTTCAACGCGAACTGTTGTCAGATTCTCCAGTCTTGTGCACTTGAAAATTCCAAGTTTGGCTTCGTTTTATAATCACCTTAAGGAGTACAAAGGGGGGGGTCATACCAAGATAAATGTTGTAAACGTTCCTTGTAAACATAGCTTTCAATAACATAAATCCAAAAAATAAAAAAACATGTTTTCATTTTTTTAACAAATTTTGAAAGTGTATCCGAACTAATTGAACACCGTG
