# Supplementary material for: Next-Generation Sequencing Reveals Recent Horizontal Transfer of a DNA Transposon between Divergent Mosquitoes
Source: PLoS One. 2011 Feb 10;6(2):e16743. doi: 10.1371/journal.pone.0016743 (PMC3037385; doi:10.1371/journal.pone.0016743)
Supplement: File S2 — MJ1 sequences from the hyrcanus group of Anopheles mosquitoes. (DOCX) [file pone.0016743.s002.docx]

**Supplemental File 2.**

***MJ1* sequences from the hyrcanus group of *Anopheles* mosquitoes**

**Naming convention: The first letter “A” refers to genus *Anopheles* and the 2^nd^ and 3^rd^ letters are the first two letters of the species name. For example, *Anopheles sinensis MJ1* is Asi_MJ1. Full species names are shown in Table 1.**

**Multiple *MJ1* clones were sequenced from each *Anopheles* species. We use “CloneX” to distinguish different clones. As shown in Supplemental file 1, distinct genomic copies were determined from *Ae. aegypti* because a genome assembly is available. However, different “clones” within each *Anopheles* species may be amplified from the same *MJ1* copy. Thus, the clone number designation simply refers to sequenced clones, not necessarily different genomic copies.**

>Asi_MJ1_Clone1

CACGGTGTTCAATAAGTTCGAATACAAGTTTTCATCATTGCGTAGGTATGCGCCATGTACTTATTCTGCATTGGTATTGGTGTCAGCTTTAGCTTGATTCATACGCTACCGAATGTGCCCAGTGTTGACAGTCTGTTAATTGTTGTTCATTTGTTACGCGCGATGAAAGAGTATCGGGACATCGTAATTAAGCGTTTTTTGAACGGTGAGCGACCCGGCGATATATTCCGGCTGCTGAAATCGCATGGGGTCAAGCGGAACTTTGTCTACACGACCATCAGGCGATACCGGGAGACGTCCTCGACCAATGACCGTGCGAGATCCGGTCGGCCGCGTTCAGCGAGGACGCCACGGGTCATCAAGATCGTGAGGGAGCGAATTCGGCGCAAAAAGAACCGCTCAATCCGGAAAACGGCTGCAGATCTCAACGTTTCCATTGGAACCGCTCACACCATACTCACCAAGGACCTTGGTTTCAGGCCTTACAAAAAACGTAAGGTCCATGGCGTTTCGGAGGCTACCAGCAAAAAGCGGTTGGATCGAGCTAAGAGGATCCTCTCTCGGCACGCTGGTCAGGAGTTTGTTTTTTCGGACGAGAAACTGTTCGTGCTGCAGCAGCCGCACAATGTGCAAAATGACCGGGTGTGGGCGCCATCGAGGGACAGCATTCCTGAATCCAATATAAACATCCCTCGGTTCCAAAGTGCCGCGTCGGTGATGGTTTGGGGGGTAGTATGCAAACGTGGTAAGCTACCCTTGGTGTTTATTGAAAAAAACGTCAAAATCAACGCGGCGTACTACAAAACTGAGGTTTTGGAAAAGGTTGTTGCCCCCAGTCTCCGAAGCCTCTACGGCGATGAGCACTACGTGTTCCAGCAGGACGGTGCACCAGCCCATACGGCAAATGTGGTTCAAGCCTGGTGTCGGGACAATTTAACCGACTTTCTGGACAAAACTTTGTGGCCTCCCAGCTCCCCGGACTTGAATCCTCTCGACTTTTTTGTTTGGTCCTATATGATGGCGAAGCTGAACGAATACAAGGTCAGCACTTTGGATCATTTCAAGACGGTAATTCTCAAAATCTGGGACGAAATGCCCATGCAGTCCGTGCGTGCCGCCTGCGACGCGTTCGAGAAACGTTTGAAGCTCGTTAAGGAGTACAAAGGGGGGGTCATTCCAAGAGAAATGTTGTAAACGTTCCTTGTAAACATAGCTTTCAATAACTTAAATCCAAAAAATAAAAAAAACATGTTTTCATTTTTTTAACAAATTTTGAAAGTGTATCCGAACTTATTGAACACCGTG

>Asi_MJ1_Clone2

CACGGTGTTCAATAAGTTCGAATACAAGTTTTCATCATTGCGTAGGTATGCGCCATGTACTTATTCTGCATTGGTATTGGTGTCAGCTTTAGCTTGATTCATACGCTACCGAATGTGCCCAGTGTTGACAGTCTGTTAATTGTTGTTCATTTGTTACGCGCGATGAAAGAGTATCGGGACATCGTAATTAAGCGTTTTTTGAACGGTGAGCGACCCGGCGATATATTCCGGCTGCTGAAATCGCATGGGGTCAAGCGGAACTTTGTCTACACGACCATCAGACGATACCGGGAGACGTCCTCGACCAATGACCGTGCGAGATCCGGTCGGCCGCGTTCAGCGAGGACGCCACGGGTCATCAAGATCGTGAGGGAGCGAATTCGGCGCAAAAAGAACCGCTCAATCCGGAAAACGGCTGCAGATCTCAACGTTTCCATTGGAACCGCTCACACCATACTCACCAAGGACCTTGGTTTCAGGCCTTACAAAAAACGTAAGGTCCATGGCGTTTCGGAGGCTACCAGCAAAAAGCGGTTGGATCGAGCTAAGAGGATCCTCTCTCGGCACGCTGGTCAGGAGTTTGTTTTTTCGGACGAGAAACTGTTCGTGCTGCAGCAGCCGCACAATGTGCGAAATGACCGGGTGTGGGCGCCATCGAGGGACAGCATTCCTGAATCCAATATAAACATCCCTCGGTTCCAAAGTGCCGCGTCGGTGATGGTTTGGGGGGTAGTATGCAAACGTGGTAAGCTACCCTTGGTGTTTATTGAAAAAAACGTCAAAATCAACGCGGCGTACTACAAAACTGAGGTTTTGGAAAAGGTTGTTGCCCCCAGTCTCCGAAGCCTCTACGGCGATGAGCACTACGTGTTCCAGCAGGACGGTGCACCAGCCCATACGGCAAATGTGGTTCAAGCCTGGTGTCGGGACAATTTAACCGACTTTCTGGACAAAACTTTGTGGCCTCCCAGCTCCCCGGACTTGAATCCTCTCGACTTTTTTGTTTGGTCCTATATGATGGCGAAGCTGAACGAATACAAGGTCAGCACTTTGGATCATTTCAAGACGGTAATTCTCAAAATCTGGGACGAAATGCCCATGCAGTCCGTGCGTGCCGCCTGCGACGCGTTCGAGAAACGTTTGAAGCTCGTTAAGGAGTACAAAGGGGGGGTCATTCCAAGAGAAATGTTGTAAACGTTCCTTGTAAACATAGCTTTCAATAACTTAAATCCAAAAAATAAAAAAAACATGTTTTCATTCTTTTAACAAATTTTGAAAGTGTATCCGAACTTATTGAACACCGTG

>Asi_MJ1_Clone3

CACGGTGTTCAATAAGTTCGAATACAAGTTTTCATCATTGCGTAGGTATGCGCCATGTACTTATTCTGCATTGGTATTGGTGTCAGCTTTAGCTTGATTCATACGCTACCGAATGTGCCCAGTGTTGACAGTCTGTTAATTGTTGTTCATTTGTTACGCGCGATGAAAGAGTATCGGGACATCGTAATTAAGCGTTTTTTGAACGGTGAGCGACCCGGCGATATATTCCGGCTGCTGAAATCGCATGGGGTCAAGCGGAACTTTGTCTACACGACCATCAGGCGATACCGGGAGACGTCCTCGACCAATGACCGTGCGAGATCCGGTCGGCCGCGTTCAGCGAGGACGCCACGGGTCATCAAGATCGTGAGGGAGCGAATTCGGCGCAAAAAGAACCGCTCAATCCGGAAAACGGCTGCAGATCTCAACGTTTCCATTGGAACCGCTCACACCATACTCACCAAGGACCTTGGTTTCAGGCCTTACAAAAAACGTAAGGTCCATGGCGTTTCGGAGGCTACCAGCAAAAAGCGGTTGGATCGAGCTAAGAGGATCCTCTCTCGGCACGCTGTTTGAAAAACTCAGTTTGTTTCAGGAGTTTGTTTTTTCGGACGAGAAACTGTTCGTGCTGCAGCAGCCGCACAATGTGCAAAATGACCGGGTGTGGGCGCCATCGAGGGACAGCATTCCTGAATCCAATATAAACATCCCTCGGTTCCAAAGTGCCACGTCGGTGATGGTTTGGGGGGCAGTATGCAAACGTGGTAAGCTACCCTTGGTGTTTATTGAAAAAAACGTCAAAATCAACGCGGCGTACTACAAAACTGAGGTTTTGGAAAAGGTTGTTGCCCCCAGTCTCCGAAGCCTCTACGGCGATGAGCACTACGTGTTCCAGCAGGACGGTGCACCAGCCCATACGGCAAATGTGGTTCAAGCCTGGTGTCGGGACAATTTAACCGACTTTCTGGACAAAACTTTGTGGCCTCCCAGCTCCCCGGACTTGAATCCTCTCGACTTTTTTGTTTGGTCCTATATGATGGCGAAGCTGAACGAATACAAGGTCAGCACTTTGGATCATTTCAAGACGGTAATTCTCAAAATCTGGGACGAAATGCCCATGCAGTCCGTGCGTGCCGCCTGCGACGCGTTCGAGAAACGTTTGAAGCTCGTTAAGGAGTACAAAGGGGGGGTCATTCCAAGAGAAATGTTGTAAACGTTCCTTGTAAACATAGCTTTCAATAACTTAAATCCAAAAAATAAAAAAAACATGTTTTCATTTTTTTAACAAATTTTGAAAGTGTATCCGAACTTATTGAACACCGTG

>Asi_MJ1_Clone4

CACGGTGTTCAATAAGTTCGAATACAAGTTTTCATCATTGCGTAGGTATGCGCCATGTACTTATTCTGCATTGGTATTGGTGTCAGCTTTAGCTTGATTCATACGCTACCGAATGTGCCCAGTGTTGACAGTCTGTTAATTGTTGTTCATTTGTTACGCGCGATGAAAGAGTATCGGGACATCGTAATTAAGCGTTTTTTGAACGGTGAGCGACCCGGCGATATATTCCGGCTGCTGAAATCGCATGGGGTCAAGCGGAACTTTGTCTACACGACCATCAGGCGATACCGGGAGACGTCCTCGACCAATGACCGTGCGAGATCCGGTCGGCCGCGTTCAGCGAGGACGCCACGGGTCATCAAGATCGTGAGGGAGCGAATTCGGCGCAAAAAGAACCGCTCAATCCGGAAAACGGCTGCAGATCTCAACGTTTCCATTGGAACCGCTCACACCATACTCACCAAGGACCTTGGTTTCAGGCCTTACAAAAAACGTAAGGTCCATGGCGTTTCGGAGGCTACCAGCAAAAAGCGGTTGGATCGAGCTAAGAGGATCCTCTCTCGGCACGCTGTTTGAAAAACTCAGTTTGTTTCAGGAGTTTGTTTTTTCGGACGAGAAACTGTTCGTGCTGCAGCAGCCGCACAATGTGCAAAATGACCGGGTGTGGGCGCCATCGAGGGACAGCATTCCTGAATCCAATATAAACATCCCTCGGTTCCAAAGTGCCACGTCGGTGATGGTTTGGGGGGCAGTATGCAAACGTGGTAAGCTACCCTTGGTGTTTATTGAAAAAAACGTCAAAATCAACGCGGCGTACTACAAAACTGAGGTTTTGGAAAAGGTTGTTGCCCCCAGTCTCCGAAGCCTCTACGGCGATGAGCACTACGTGTTCCAGCAGGACGGTGCACCAGCCCATACGGCAAATGTGGTTCAAGCCTGGTGTCGGGACAATTTAACCGACTTTCTGGACAAAACTTTGTGGCCTCCCAGCTCCCCGGACTTGAATCCTCTCGACTTTTTTGTTTGGTCCTATATGATGGCGAAGCTGAACGAATACAAGGTCAGCACTTTGGATCATTTCAAGACGGTAATTCTCAAAATCTGGGACGAAATGCCCATGCAGTCCGTGCGTGCCGCCTGCGACGCGTTCGAGAAACGTTTGAAGCTCGTTAAGGAGTACAAAGGGGGGGTCATTCCAAGAGAAATGTTGTAAACGTTCCTTGTAAACATAGCTTTCAATAACTTAAATCCAAAAAATAAAAAAAACATGTTTTCATTTTTTTAACAAATTTTGAAAGTGTATCCGAACTTATTGAACACCGTG

>Asi_MJ1_Clone5

CACGGTGTTCAATAAGTTCGAATACAAGTTTTCATCATTGCGTAGGTATGCGCCATGTACTTATTCTGCATTGGTATTGGTGTCAGCTTTACCTTGATTCATACGCTACCGAATGTGCCCAGTGTTGACAGTCTGTTAATTGTTGTTCATTTGTTACGCGCGATGAAAGAGTATCGGGACATCGTAATTAAGCGTTTTTTGAACGGTGAGCGACCCGGCGATATATTCCGGCTGCTGAAATCGCATGGGGTCAAGCGGAACTTTGTCTACACGACCATCAGGCGATACCGGGAGACGTCCTCGACCAATGACCGTGCGAGATCCGGTCGGCCGCGTTCAGCGAGGACGCCACGGGTCATCAAGATCGTGAGGGAGCGAATTCGGCGCAAAAAGAACCGCTCAATCCGGAAAACGGCTGCAGATCTCAACGTTTCCATTGGAACCGCTCACACCATACTCACCAAGGACCTTGGTTTCAGGCCTTACAAAAAACGTAAGGTCCATGGCGTTTCGGAGGCTACCAGCAAAAAGCGGTTGGATCGAGCTAAGAGGATCCTCTCTCGGCACGCTGGTCAGGAGTATGTTTTTTCGGACGAGAAACTGTTCGTGCTGCAGCAGCCGCACAATGTGCAAAATGACCGGGTGTGGGCGCCATCGAGGGACAGCATTCCTGAATCCAATATAAACATCCCTCGGTTCCAAAGTGCCGCGTCGGTGATGGTTTGGGGGGCAGTATGCAAACGTGGTAAGCTACCCTTCGTGTTTATTGAAAAAAACGTCAAAATCAACGCGGCGTACTACAAAACTGAGGTTTTGGAAAAGGTTGTTGCCCCCAGTCTCCGAAGCCTCTACGGTGATGAGCACTACGTGTTCCAGCAGGACGGTGCACCAGCCCATACGGCAAATGTGGTTCAAGCCTGGTGTCGGGACAATTTAACCGACTTTCTGGACAAAACTTTGTGGCCTCCCAGCTCCCCGGACTTGAATCCTCTCGACTTTTTTGTTTGGTCCTATATGATGGCGAAGCTGAACGAATACAAGGTCAGCACTTTGGATCATTTCAAGACGGTAATTCTCAAAATCTGGGACGAAATGCCCATGCAGTCCGTGCGTGCCGCCTGCGACGCGTTCGAGAAACGTTTGAAGCTCGTTAAGGAGTACAAAGGGGGGTCATTCCAAGAGAAATGTTGTAAACGTTCCTTGTAAACATAGCTTTCAATAACTTAAATCCAAAAAATAAAAAAAACATGTTTTCATTTTTTTTACAAATTTTGAAAGTGTATCCGAACTTATTGAACACCGTG

>Asi_MJ1_Clone6

CACGGTGTTCAATAAGTTCGAATACAAGTTTTCATCATTGCGTAGGTATGCGCCATGTACTTATTCTGCATTGGTATTGGTGTCAGCTTTAGCTTGATTCATACGCTACCGAATGTGCCCAGTGTTGACAGTCTGTTAATTGTTGTTCATTTGTTACGCGCGATGAAAGAGTATCGGGACATCGTAATTAAGCGTTTTTTGAACGGTGAGCGACCCGGCGATATATTCCGGCTGCTGAAATCGCATGGGGTCAAGCGGAACTTTGTCTACACGACCATCAGGCGATACCGGGAGACGTCCTCGACCAATGACCGTGCGAGATCCGGTCGGCCGCGTTCAGCGAGGACGCCACGGGTCATCAAGATCGTGAGGGAGCGAATTCGGCGCAAAAAGAACCGCTCAATCCGGAAAACGGCTGCAGATCTCAACGTTTCCATTGGAACCGCTCACACCATACTCACCAAGGACCTTGGTTTCAGGCCTTACAAAAAACGTAAGGTCCATGGCGTTTCGGAGGCTACCAGCAAAAAGCGGTTGGATCGAGCTAAGAGGATCCTCTCTCGGCACGCTGTTTGAAAAACTCAGTTTGTTTCAGGAGTTTGTTTTTTCGGACGAGAAACTGTTCGTGCTGCAGCAGCCGCACAATGTGCAAAATGACCGGGTGTGGGCGCCATCGAGGGACAGCATTCCTGAATCCAATATAAACATCCCTCGGTTCCAAAGTGCCACGTCGGTGATGGTTTGGGGGGCAGTATGCAAACGTGGTAAGCTACCCTTGGTGTTTATTGAAAAAAACGTCAAAATCAACGCGGCGTACTACAAAACTGAGGTTTTGGAAAAGGTTGTTGCCCCCAGTCTCCGAAGCCTCTACGGCGATGAGCACTACGTGTTCCAGCAGGACGGTGCACCAGCCCATACGGCAAATGTGGTTCAAGCCTGGTGTCGGGACAATTTAACCGACTTTCTGGACAAAACTTTGTGGCCTCCCAGCTCCCCGGACTTGAATCCTCTCGACTTTTTTGTTTGGTCCTATATGATGGCGAAGCTGAACGAATACAAGGTCAGCACTTTGGATCATTTCAAGACGGTAATTCTCAAAATCTGGGACGAAATGCCCATGCAGTCCGTGCGTGCCGCCTGCGACGCGTTCGAGAAACGTTTGAAGCTCGTTAAGGAGTACAAAGGGGGGGTCATTCCAAGAGAAATGTTGTAAACGTTCCTTGTAAACATAGCTTTCAATAACTTAAATCCAAAAAATAAAAAAAACATGTTTTCATTTTTTTAACAAATTTTGAAAGTGTATCCGAACTTATTGAACACCGTG

>Asi_MJ1_Clone7

CACGGTGTTCAATAAGTTCGAATACAAGTTTTCATCATTGCGTAGGTATGCGCCATGTACTTATTCTGCATTGGTATTGGTGTCAGCTTTAGCTTGATTCATACGCTACCGAATGTGCCCAGTGTTGACAGTCTGTTAATTGTTGTTCATTTGTTACGCGCGATGAAAGAGTATCGGGACATCGTAATTAAGCGTTTTTTGAACGGTGAGCGACCCGGCGATATATTCCGGCTGCTGAAATCGCATGGGGTCAAGCGGAACTTTGTCTACACGACCATCAGACGATACCGGGAGACGTCCTCGACCAATGACCGTGCGAGATCCGGTCGGCCGCGTTCAGCGAGGACGCCACGGGTCATCAAGATCGTGAGGGAGCGAATTCGGCGCAAAAGGAACCGCTCAATCCGGAAAACGGCTGCAGATCTCAACGTTTCCATTGGAACCGCTCACACCATACTCACCAAGGACCTTGGTTTCAGGCCTTACAAAAAACGTAAGGTCCATGGCGTTTCGGAGGCTACCAGCAAAAAGCGGTTGGATCGAGCTAAGAGGATCCTCTCTCGGCACGCTGGTCAGGAGTTTGTTTTTTCGGACGAGAAACTGTTCGTGCTGCAGCAGCCGCACAATGTGCAAAATGACCGGGTGTGGGCGCCATCGAGGGACAGCATTCCTGAATCCAATATAAACATCCCTCGGTTCCAAAGTGCCGCGTCGGTGATGGTTTGGGGGGTAGTATGCAAACGTGGTAAGCTACCCTTGGTGTTTATTGAAAAAAACGTCAAAATCAACGCGGCGTACTACAAAACTGAGGTTTTGGAAAAGGTTGTTGCCCCCAGTCTCCGAAGCCTCTACGGCGATGAGCACTACGTGTTCCAGCAGGACGGTGCACCAGCCCATACGGCAAATGTGGTTCAAGCCTGGTGTCGGGACAATTTAACCGACTTTCTGGACAAAACTTTGTGGCCTCCCAGCTCCCCGGACTTGAATCCTCTCGACTTTTTTGTTTGGTCCTATATGATGGCGAAGCTGAACGAATACAAGGTCAGCACTTTGGATCATTTCAAGACGGTAATTCTCAAAATCTGGGACGAAATGCCCATGCAGTCCGTGCGGTGCCGCCTGCGACGCGTTCGAGAAACGTTTGAAGCTCGTTAAGGAGTACAAAGGGGGGGTCATTCCAAGAGAAATGTTGTAAACGTTCCTTGTAAACATGGCTTTCAATAACTTAAATCCAAAAAATAAAAAAAACATGTTTTCATTTTTTTAACAAATTTTGAAAGTGTATCCGAACTTATTGAACACCCGGG

>Asi_MJ1_Clone8

CACGGTGTTCAATAAGTTCGAATACAAGTTTTCATCATTGCGTAGGTATGCGCCATGTACTTATTCTGCATTGGTATTGGTGTCACGCTTTAGCTTGATTCCATACGCCTACCGAATGTGCCCAGTGTTGACAGTCTGTTAATTGTTGTTCATTTGTTACGCGCGATGAAAGAGTATCGGGACATCGTAATTAAGCGTTTTTTGAACGGTGAGCGACCCGGCGATATATTCCGGCTGCTGAAATCGCATGGGGTCAAGCGGAACTTTGTCTACACGACCATCAGGCGATACCGGGAGACGTCCTCGACCAATGACCGTGCGAGATCCGGTCGGCCGCGTTCAGCGAGGACGCCACGGGTCATCAAGATCGTGAGGGAGCGAATTCGGCGCAAAAAGAACCGCTCAATCCGGAAAATGGCTGCAGATCTCAACGTTTCCATTGGAACCGCTCACACCATACTCACCAAGGACCTTGGTTTCAGGCCTTACAAAAAACGTAAGGTCCATGGCGTCTCGGAGGCTACCAGCAAAAAGCGGTTGGATCGAGCTAAGAGGATCCTCTCTCGGCACGCTGGTCAGGAGTTTGTTTTGTCGGACGAGAAACTGTTCGTGCTGCAGCAGCCGCACAATGTGCAAAATGACCGGGTGTGGGCGCCATCGAGGGACAGCATTCCTGAATCCAATATAAACATCCCTCGGTTCCAAAGTGCCGCGTCGGTGATGGTTTGGGGGGCAGTATGCAAACGTGGTAAGCTACCCTTGGTGTTTATTGAAAAAAACGTCAAAATCAACGCGGCGTACTACAAAACTGAGGTTTTGGAAAAGGTTGTTGCCCCCAGTCTCCGAAGCCTCTACGGCGATGAGCACTACGTGTTCCAGCAGGACGGTGCACCAGCCCATACGGCAAATGTGGTTCAAGCCTGGTGTCGGGACAATTTAACCGACTTTCTGGACAAAACTTTGTGGCCTCCCAGCTCCCCGGACTTGAATCCTCTCGACTTTTTTGTTTGGTCCTATATGATGGCGAAGCTGAACGAATACAAGGTCAGCACTTTGGATCATTTCAAGACGGTAATTCTCAAAATCTGGGACGAAATGCCCATGCAGTTCGTGCGTGCCGCCTGCGACGCGTTCGAGAAACGTTTGAAGCTCGTTAAGGAGTACAAAGGGGGGGTCATTCCAAGAGAAATGTTGTAAACGTTCCTTGTAAACATAGCTTTCAATAACTTAAATCCAAAAAATAAAAAAAACATGTTTTCATTTTTTTAACAAATTTTGAAAGTGTATCCGAACTTATTGAACACCGTG

>Asi_MJ1_Clone9

CACGGTGTTCAATAAGTTCGAATACAAGTTTTCATCATTGCGTAGGTATGCGCCATGTACTTATTCTGCATTGGTATTGGTGTCAGCTTTAGCTTGATTCATACGCTACCGAATGTGCCCAGTGTTGACAGTCTGTTAATTGTTGTTCATTTGTTACGCGCGATGAAAGAGTATCGGGACATCGTAATTAAGCGTTTTTTGAACGGTGAGCGACCCGGCGATATATTCCGGCTGCTGAAATCGCATGGGGTCAAGCGGAACTTTGTCTACACGACCATCAGGCGATACCGGGAGACGCCCTCGACCAATGACCGTGCGAGATCCGGTCGGCCGCGTTCAGCGAGGACGCCACGGGTCATCAAGATCGTGAGGGAGCGAATTCGGCGCAAAAAGAACCGCTCAATCCGGAAAACGGCTGCAGATCTCAACGTTTCCATTGGAACCGCTCACACCATACTCACCAAGGACCTTGGTTTCAGGCCTTA

CAAAAAACGTAAGGTCCATGGCGTTTCGGAGGCTACCAGCAAAAAGCGGTTGGATCGAGCTAAGAGGATCCTCTCTCGGCACGCTGGTCAGGAGTTTGTTTTTTCGGACGAGAAACTGTTCGTGCTGCAGCAGCCGCACAATGTGCAAAATGACCGGGTGTGGGCGCCATCGAGGGACAGCATTCCTGAATCCAATATAAACATCCCTCGGTTCCAAAGTGCCGCGTCGGTGATGGTTTGGGGGGCAGTATGCAAACGTGGTAAGCTACCCTTGGTGTTTATTGAAAAAAACGTCAAAATCAACGCGGCGTACTACAAAACTGAGGTTTTGGAAAAGGTTGTTGCCCCCAGTCTCCGAAGCCTCTACGGTGATGAGCACTACGTGTTCCAGCAGGACGGTGCACCAGCCCATACAGCAATTGTGGTTCGAGCCTGGTGTCGGGACAATTTAACCGACTTTCTGGACAAAACTTTGTGGCCTCCCAGCTCCCCGGACTTGAATCCTCTCGACTTTTTTGTTTGGTCCTATATGATGGCGAAGCTGAACGAATACAAGGTCAGCACTTTGGATCATTTCAAGCCGGTAATTCTCAAAATCTGGGACGAAATGCCCATGCAGTCCGTGCGTGCCGCCTGCGACGCGTTCGAGAAACGTTTGAAGCTCGTTAAGGAGTACAAAGGGGGGGTCATTCCAAGAGAAATGTTGTAAACGTTCCTTGTAAACATAGCTTTCAATAACTTAAATCCAAAAAATAAAAAAAAACATGTTTTCATTTTTTTAACAAATTTTGAAAGTGTATCCGAACTTATTGAACACCGTG

>Ale_MJ1_Clone1

CACGGTGTTCAATAAGTTCGAATACAAGTTTTCATCATTGCGTAGGTATGCGCCATGTACTTATTCTGCATTGGTATTGGTGTCAGCTTTAGCTTGATTCATACGCTACCGAATGTGCCCAGTGTTGACATTCTGTTAATTGTTGTTCATTTGTTACGCGCGATGAAAGAGTATCGGGACATCGTAATTAAGCGTTTTTTGAACGGTGAGCGACCCGGCGATATATTCCGGCTGCTGAAATCGCATGGGGTCAAACGGAACTTTGTCTACACGACCATCAGGCGATACCGGGAGACGTCCTCGACCAATGACCGTGCGAGATCCGGTCGGCCGCGTTCAGCGAGGACGCCACGGATCATCAAGATCGTGAGGGAGCGAATTCGGCGCAAAAAGAACCGCTCAATCCGGAAAATGGCTGCAGATCTCAACGTTTCCATTGGAACCGCTCACACCATACTCACCAAGGACCTTGGTTTCAGGCCTTACAAAAAACGTAAGGTCCATGGCGTTTCGGAGGCTACCAGCAAAAAGCGGTTGGATCGAGCTAAGAGGATCCTCTCTCGGCACGCTGGTCAGGAGTTTGTTTTTTCGGACGAGAAACTGTTCGTGCTGCAGCAGCCGCACAATGTGCAAAATGACCGGGTGTGGGCGCCATCGAGGGACAGCATTCCTGAATCCAATATAAACATCCCTCGGTTCCAAAGTGCCGCGTCGGTGATGGTTTGGGGGGCAGTATGCAAACGTGGTAAGCTACCCTTGGTGTTTATTGAAAAAAACGTCAAAATCAACGCGGCGTACTACAAAACTGAGGTTTTGGAAAAGGTTGTTGCCTCCAGTCTTCGAAGCCTCTACGGCGATGAGCACTACGTGTTCCAGCAGGACGGTGCACCAGCCCATACGGCAAATGTGGTTCAAGCCTGGTGTCGGGACAATTTAACCGACTTTCTGGACAAAACTTTGTGGCCTCCCAGCTCCCCGGACTTGAATCCTCTCGACTTTTTTGTTTGGTCCTATATGATGGCGAAGCTGAACGAATACAAGGTCAGCACTTTGGATCATTTCAAGACGGTAATTCTCAAAATCTGGGACGAAATGCCCATGCAGTCCGTGCGTGCCGCCTGCGACGCGTTCGAGAAACGTTTGAAGCTCGTTAAGGAGTACAAAGGGGGGGTCATCCCAAGAGAAATGTTGTAAACGTTCCTTGTAAACATAGCTTTCAATAACTTAAATCCAAAAAATAAAAAAAAACATGTTTTCATTTTTTTAACAAATTTTGAAAGTGTATCCGAACTTATTGAACACCGTG

>Ale_MJ1_Clone2

CACGGTGTTCAATAAGTTCGAATACAAGTTTTCATCATTGCGTAGGTATGCGCCATGTACTTATTCTGCATTGGTATTGGTGTCAGCTTTAGCTTGATTCATACGCTACCGAATGTGCCCAGTGTTGACATTATGTTAATTGTTGTTCATCTGTTACGCGCGATGAAAGAGTATCGGGACATCGTAATTAAGCGTTTTTTGAACGGTGAGCGACCCGGCGATATATTCCGGCTGCTGAAATCGCATGGGGTCAAGCGGAACTTTGTCTACACGACCATCAGGCGATACCGGGAGACGTCCTCGACCAATGACCGTGCGAGATCCGGTCGGCCGCGTTCAGCGAGGACGCCACGGGTCATCAAGATCGTGAGGGAGCGAATTCGGCGCAAAAAGAACCGCTCAATCCGGAAAACAGCTGCAGATCTCAACGTTTCCATTGGAACCGCTCACACCATACTCACCAAGGACCTTGGTTTCAGGCCTTACAAAAAACGTAAGGTCCATGGCGTTTCGGAGGCTACCAGCAAAAAGCGGTTGGATCGAGCTAAGAGGATCCTCTCTCGGCACGCTGGTCAGGAGTTTGTTTTTTCGGACGAGAAACTGTTCGTGCTGCAGCAGCCGCACAATGTGCAAAATGACCGGGTGTGGGCGCCATCGAGGGACAGCATTCCTGAATCCAATATAAACATCCCTCGGTTCCAAAGTGCCGCGTCGGTGATGGTTTGGGGGGCAGTATGCAAACGTGGTAAGCTACCCTTGGTGTTTATTGAAAAAAACGTCAAAATCAACGCGGCGTACTACAAAACTGAGGTTTTGGAAAAGGTTGTTGCCCCCAGTCTCCGAAGCCTCTACGGCGATGAGCACTACGTGTTCCAGCAGGACGGTGCACCAGCCCATACGGCAAATGTGGTTCAAGCCTGGTGTCGGGACAATTTAACCGACTTTCTGGACAAAACTTTGTGGCCTCCCAGCTCCCCGGACTTGAATCCTCTCGACTTTTTTGTTTGGTCCTATATGATGGCGAAGCTGAACGAATACAAGGTCAGCACTTTGGATCATTTCAAGACGGTAATTCTCAAAATCTGGGACGAAATGCCCATGCAGTCCGTGCGTGCCGCCTGCGACGCGTTCGAGAAACGTTTGAAGCTCGTTAAGGAGTACAAAGGGGGGGTCATTCCAAGAGAAATGTTGTAAACGTTCCTTGTAAACATAGCTTTCAATAACTTAAATCCAAAAAATAAAAAAAACATGTTTTCATTTTTTTAACAAATTTTGAAAGTGTATCCGAACTTATTGAACACCGTG

>Ale_MJ1_Clone3

CACGGTGTTCAATAAGTTCGAATACAAGTTTTCATCATTGCGTAGGTATGCGCCATGTACTTATTCTGCATTGGTATTGGTGTCAGCTTTAGCTTGATTCATACGCTACCGAATGTGCCCAGTGTTGACATTCTGTTAATTGTTGTTCATTTGTTACGCGCGATGAAAGAGTATCGGGACATCGTAATTAAGCGTTTTTTGAACGGTGAGCGACCCGGCGATATATTCCGGCTGCTGAAATCGCATGGGGTCAAGCGGAACTTTGTCTACACGACCATCAGGCGATACCGGGAGACGTCCTCGACCAATGACCGTGCGAGATCCGGTCGGCCGCGTTCAGCGAGGACGCCACGGGTCATCAAGATCGTGAGGGAGCGAATTCGGCGCAAAAAGAACCGCTCAATCCGGAAAACGGCTGCAGATCTCAACGTTTCCATTGGAACCGCTCACACCATACTCACCAAGGACCTTGGTTTCAGGCCTTACAAAAAACGTAAGGTCCATGGCGTTTCGGAGGCTACCAGCAAAAAGCGGTTGGATCGAGCTAAGAGGATCCTCTCTCGGCACGCTGGTCAGGAGTTTGTTTTTTCGGACGAGAAACTGTTCGTGCTGCAGCAGCCGCACAATGTGCAAAATGACCGGGTGTGGGCGCCATCGAGGGACAGCATTCCTGAATCCAATATAAACATCCCTCGGTTCCAAAGTGCCGCGTCGGTGATGGTTTGGGGGGCAGTATGCAAACGTGGTAAGCTACCCTTGGTGTTTATTGAAAAAAACGTCAAAATCAACGCGGCGTACTACAAAACTGAGGTTTTGGAAAAGGTTGTTGCCCCCAGTCTCCGAAGCCTCTACGGCGATGAGCACTACGTGTTCCAGCAGGACGGTGCACCAGCCCATACGGCAAATGTGGTTCAAGCCTGGTGTCGGGACAATTTAACCGACTTTCTGGACAAAACTTTGTGGCCTCCCAGCTCCCCGGACTTGAATCCTCTCGACTTTTTTGTTTGGTCCTATATGATGGCGAAGCTGAACGAATACAAGGTCAGCACTTTGGATCATTTCAAGACGGTAATTCTCAAAATCTGGGACGAAATGCCCATGCAGTCCGTGCGTGCCGCCTGCGACGCGTTCGAGAAACGTTTGAAGCTCGTTAAGGAGTACAAAGGGGGGGTCATTCCAAGAGAAATGTTGTAAACGTTCCTTGTAAACATAGCTTTCAATAACTTAAATCCAAAAAATAAAAAAAACATGTTTTCATTTTTTTAACAAATTTTGAAAGTGTATCCGAACTTATTGAACACCGTG

>Ale_MJ1_Clone4

CACGGTGTTCAATAAGTTCGAATACAAGTTTTCATCATTGCGTAGGTATGCGCCATGTACTTATTCTGCATTGGTATTGGTGTCAGCTTTAGCTTGATTCATACGCTACCGAATGTGCCCAGTGTTGACACTCTGTTAATTGTTGTTCATTTGTTACGCGCGATGAAAGAGTATCGGGACATCGTAATTAAGCGTTTTTTGAACGGTGAGCGACCCGGCGATATATTCCGGCTGCTGAAATCGCATGGGGTCAAGCGGAACTTTGTCTACACGACCATCAGGCGATACCGGGAGACGTCCTCGACCAATGACCGTGCGAGATCCGGTCGGCCGCGTTCAGCGAGGACGCCACGGGTCATCAAGATCGTGAGGGAGCGAATTCGGCGCAAAAAGAACCGCTCAATCCGGAAAACGGCTGCAGATCTCAACGTTTCCATTGGAACCGCTCACACCATACTCACCAAGGACCTTGGTTTCAGGCCTTACAAAAAACGTAAGGTCCATGGCGTTTCGGAGGCTACCAGCAAAAAGCGGTTGGATCGAGCTAAGAGGATCCTCTCTCGGCACGCTGGTCAGGAGTTTGTTTTTTCGGACGAGAAACTGTTCGTGCTGCAGCAGCCGCACAATGTGCAAAATGACCGGGTGTGGGCGCCATCGAGGGACAGCATTCCTGAATCCAATATAAACATCCCTCGGTTCCAAAGTGCCGCGTCGGTGATGGTTTGGGGGGCAGTATGCAAACGTGGTAAGCTACCCTTGGTGTTTATTGAAAAAAAACGTCAAAATCAACGCGGCGTACTACAAAACTGAGGTTTTGGAAAAGGTTGTTGCCCCCAGTCTCCGAAGCCTCTACGGCGATGAGCACTACGTGTTCCAGCAGGACGGTGCACCAGCCCATACGGCAAATGTGGTTCAAGCCTGGTGTCGGGACAATTTAACCGACTTTCTGGACAAAACTTTGTGGCCTCCCAGCTCCCCGGACTTGAATCCTCTCGACTTTTTTGTTTGGTCCTATATGATGGCGAAGCTGAACGAATACAAGGTCAGCACTTTGGATCATTTCAAGACGGTAATTCTCAAAATCTGGGACGAAATGCCCATGCAGTCCGTGCGTGCCGCCTGCGACGCGTTCGAGAAACGTTTGAAGCTCGTTAAGGAGTACAAAGGGGGGGTCATTCCAAGAGAAATGTTGTAAACGTTCCTTGTAAACATAGCTTTCAATAACTTAAATCCAAAAAATAAAAAAAACATGTTTTCATTTTTTTAACAAATTTTGAAAGTGTATCCGAACTTATTGAACACCGTG

>Ale_MJ1_Clone5

CACGGTGTTCAATAAGTTCGAATACAAGTTTTCATCATTGCGTAGGTATGCGCCATGTACTTATTCTGCATTGGTATTGGTGTCAGCTTTAGCTTGATTCATACGCTACCGAATGTGCCCAGTGTTGACATTCTGTTAATTGTTGTTCATTTGTTACGCGCGATGAAAGAGTATCGGGACATCGTAATTAAGCGTTTTTTGAACGGTGAGCGACCCGGCGATATATTCCGGCTGCTGAAATCGCATGGGGTCAAGCGGAACTTTGTCTACACGACCATCAGGCGATACCGGGAGACGTCCTCGACCAATGACCGTGCGAGATCCGGTCGGCCGCGTTCAGCGAGGACGCCACGGGTCATCAAGATCGTGAGGGAGCGAATTCGGCGCAAAAAGAACCGCTCAATCCGGAAAACGGCTGCAGATCTCAACGTTTCCATTGGAACCGCTCACACCATACTCACCAAGGACCTTGGTTTCAGGCCTTACAAAAAACGTAAGGTCCATGGCGTTTCGGAGGCTACCAGCAAAAAGCGGTTGGATCGAGCTAAGAGGATCCTCTCTCGGCACGCTGGTCAGGAGTTTGTTTTTTCGGACGAGAAACTGTTCGTGCTGCAGCAGCCGCACAATGTGCAAAATGACCGGGTGTGGGCGCCATCGAGGGACAGCATTCCTGAATCCAATATAAACATCCCTCGGTTCCAAAGTGCCGCGTCGGTGATGGTTTGGGGGGCAGTATGCAAACGTGGTAAGCTACCCTTGGTGTTTATTGAAAAAAACGTCAATATCAACGCGGCGTACTACAAAACTGAGGTTTTGGAAAAGGTTGTTGCCCCCAGTCTCCGAAGCCTCTACGGCGATGAGCACTACGTGTTCCAGCAGGACGGTGCACCAGCCCATACGGCAAATGTGGTTCAAGCCTGGTGTCGGGACAATTTAACCGACTTTCTGGACAAAACTTTGTGGCCTCCCAGCTCCCCGGACTTGAATCCTCTCGACTTTTTTGTTTGGTCCTATATGATGGCGAAGCTGAACGAATACAAGGTCAGCACTTTGGATCATTTCAAGACGGTAATTCTCAAAATCTGGGACGAAATGCCCATGCAGTCCGTGCGTGCCGCCTGCGACGCGTTCGAGAAACGTTTGAAGCTCGTTAAGGAGTACAAAGGAGGGGGGGGGGGGGGGGTCATTCCAAGAGAAATGTTGTAAACGTTCCTTGTAAACATAGCTTTCAATAACTTAAATCCAAAAAATAAAAAAAACATGTTTTCATTTTTTTAACAAATTTTGAAAGTGTATCCGAACTTATTGAACACCGTG

>Ale_MJ1_Clone6

CACGGTGTTCAATAAGTTCGAATACAAGTTTTCATCATTGCGTAGGTATGCGCCATGTACTTATTCTGCATTGGTATTGGTGTCAGCTTTAGCTTGATTCATACGCTACCGAATGTGCCCAGTGTTGACATTCTGTTAATTGTTGTTCATTTGTTACGCGCGATGAAAGAGTATCGGGACATCGTAATTAAGCGTTTTTTGAACGGTGAGCGACCCGGCGATATATTCCGGCTGCTGAAATCGCATGGGGTCAAGCGGAACTTTGTCTACACGACCATCAGGCGATACCGGGAGACGTCCTCGACCAATGACCGTGCGAGATCCGGTCGGCCGCGTTCAGCGAGGACGCCACGGGTCATCAAGATCGTGAGGGAGCGAATTCGGCGCAAAAAGAACCGCTCAATCCGGAAAACGGCTGCAGATCTCAACGTTTCCATTGGAACCGCTCACACCATACTCACCAAGGACCTTGGTTTCAGGCCTTACAAAAAACGTAAGGTCCATGGCGTTTCGGAGGCTACCAGCAAAAAGCGGTTGGATCGAGCTAAGAGGATCCTCTCTCGGCACGCTGGTCAGGAGTTTGTTTTTTCGGACGAGAAACTGTTCGTGCTGCAGCAGCCGCACAATGTGCAAAATGACCGGGTGTGGGCGCCATCGAGGGACAGCATTCCTGAATCCAATATAAACATCCCTCGGTTCCAAAGTGCCGCGTCGGTGATGGTTTGGGGGGCAGTATGCAAACGTGGTAAGCTACCCTTGGTGTTTATTGAAAAAAACGTCAAAATCAACGCGGCGTACTACAAAACTGAGGTTTTGGAAAAGGTTGTTGCCCCCAGTCTCCGAAGCCTCTACGGCGATGAGCACTACGTGTTCCAGCAGGACGGTGCACCAGCCCATACGGCAAATGTGGTTCAAGCCTGGTGTCGGGACAATTTAACCGACTTTCTGGACAAAACTTTGTGGCCTCCCAGCTCCCCGGACTTGAATCCTCTCGACTTTTTTGTTTGGTCCTATATGATAGCGAAGCTGAACGAATACAAGGTCAGCACTTTGGATCATTTCAAGACGGTAATTCTCAAAATCTGGGACGAAATGCCCATGCAGTCCGTGCGTGCCGCCTGCGACGCGTTCGAGAAACGTTTGAAGCTCGTTAAGGAGTACAAAGGGGGGGTCATTCCAAGAGAAATGTTGTAAACGTTCCTTGTAAACATAGCTTTCAATAACTTAAATCCAAAAAATAAAAAAAACATGTTTTCATTTTTTTAACAAATTTTGAAAGTGTATCCGAACTTATTGAACACCGTG

>Ale_MJ1_Clone7

CACGGTGTTCAATAAGTTCGAATACAAGTTTTCATCATTGCGTAGGTATGCGCCATGTACTTATTCTGCATTGGTATTGGTGTCAGCTTTAGCTTGATTCATACGCTACCGAATGTGCCCAGTGTTGACATTCTGTTAATTGTTGTTCATTTGTTACGCGCGATGAAAGAGTATCGGGACATCGTAATTAAGCGTTTTTTGAACGGTGAGCGACCCGGCGATATATTCCGGCTGCTGAAATCGCATGGGGTCAAGCGGAACTTTGTCTACACGACCATCAGGCGATACCGGGAGACGTCCTCGACCAATGACCGTGCGAGATCCGGTCGGCCGCGTTCAGCGAGGACGCCACGGGTCATCAAGATCGTGAGGGAGCGAATTCGGCGCAAAAAGAACCGCTCAATCCGGAAAACGGCTGCAGATCTCAACGTTTCCATTGGAACCGCTCACACCATACTCACCAAGGACCTTGGTTTCAGGCCTTACAAAGAACGTAAGGTCCATGGCGTTTCGGAGGCTACCAGCAAAAAGCGGTTGGATCGAGCTAAGAGGATCCTCTCTCGGCACGCTGGTCAGGAGTTTGTTTTTTCGGACGAGAAACTGTTCGTGCTGCAGCAGCCGCACAATGTGCAAAATGACCGGGTGTGGGCGCCATCGAGGGACAGCATTCCTGAATCCAATATAAACATCCCTCGGTTCCAAAGTGCCGCGTCGGTGATGGTTTGGGGGGCAGTACGCAAACGTGGTAAGCTACCCTTGGTGTTTATTGAAAAAAACGTCAAAATCAACGCGGCGTACTACAAAACTGAGGTTTTGGAAAAGGTTGTTGCCCCCAGTCTCCGAAGCCTCTACGGCGATGAGCACTACGTGTTCCAGCAGGACGGTGCACCAGCCCATACGGCAAATGTGGTTCAAGCCTGGTGTCGGGACAATTTAACCGACTTTCTGGACAAAACTTTGTGGCCTCCCAGCTCCCCGGACTTGAATCCTCTCGACTTTTTTGTTTGGTCCTATATGATGGCGAAGCTGAACGAATACAAGGTCAGCACTTTGGATCATTTCAAGACGGTAATTCTCAAAATCTGGGACGAAATGCCCATGCAGTCCGTGCGTGCCGCCTGCGACGCGTTCGAGAAACGTTTGAAGCTCGTTAAGGAGTACAAAGGGGGGGTCATTCCAAGAGAAATGTTGTAAACGTTCCTTGTAAACATAGCTTTCAATAACTTAAATCCAAAAAATAAAAAAAACATGTTTTCATTTTTTTAACAAATTTTGAAAGTGTATCCGAACTTATTGAACACCGTG

>Ale_MJ1_Clone8

CACGGTGTTCAATAAGTTCGAATACAAGTTTTCATCATTGCGTAGGTATGCGCCATGTACTTATTCTGCATTGGTATTGGTGTCAGCTTTAGCTTGATTCATACGCTACCGAATGTGCCCAGTGTTGACATTCTGTTAATTGTTGTTCATTTGTTACGCGCGATGAAAGAGTATCGGGACATCGTAATTAAGCGTTTTTTAAACGGTGAGCGACCCGGCGATATATTCCGGCTGCTGAAATCGCATGGGGTCAAGCGGAACTTTGTCTACACGACCATCAGGCGATACCGGGAGACGTCCTCGACCAATGACCGTGCGAGATCCGGTCGGCCGCGTTCAGCGAGGACGCCACGGGTCATCAAGATCGTGAGGGAGCGAATTCGGCGCAAAAAGAACCGCTCAATCCGGAAAACGGCTGCAGATCTCAACGTTTCCATTGGAACCGCTCACACCATACTCACCAAGGACCTTGGTTTCAGGCCTTACAAAAAACGTAAGGTCCATGGCGTTTCGGAGGCTACCAGCAAAAAGCGGTTGGATCGAGCTAAGAGGATCCTCTCTCGGCACGCTGGTCAGGAGTTTGTTTTTTCGGACGAGAAACTGTTCGTGCTGCAGCAGCCGCACAATGTCCCATCGCCATCGAGGGACAGCATTCCTGAATCCAATATAAACATCCCTCGGTTCCAAAGTGCCGCGTCGGTGATGGTTTGGGGGGCAGTATGCAAACGTGGTAAGCTACCCTTGGTGTTTATTGAAAAAAACGTCAAAATCAACGCGGCGTACTACAAAACTGAGGTTTTGGAAAAGGTTGTTGCCCCCAGTCTCCGAAGCCTCTACGGCGATGAGCGCTACGTGTTCCAGCAGGACGGTGCACCAGCCCATACGGCAAATGTGGTTCAAGCCTGGTGTCCGGACAATTTAACCGACTTTCTGGACAAAACTTTGTGGCCTCCCAGCTCCCCGGACTTGAATCCTCTCGACTTTTTTGTTTGGTCCTATATGATGGCGAAGCTGAACGAATACAAGGTCAGCACTTTGGATCATTTCAAGACGGTAATTCTCAAAATCTGGGACGAAATGCCCATGCAGTCCGTGCGTGCCGCCTGCGACGCGTTCGAGAAACGTTTGAAGCTCGTTAAGGAGTACAAAGGGGGGGTCATTCCAAGAGAAATGTTGTAAACGTTCCTTGTAAACATAGATTTCAATAACTTAAATCCAAAAAATAAAAAAAACATGTTTTCATTTTTTTAACAAATTTTGAAAGTGTATCCGAACTTATTGAACACCGTG

>Ale_MJ1_Clone9

CACGGTGTTCAATAAGTTCGAATACAAGTTTTCATCATTGCGTAGGTATGCGCCATGTACTTATTCTGCATTGGTATTGGTGTCAGCTTTAGCTTGATTCATACGCTACCGAATGTGCCCAGTGTTGACATTCTGTTAATTGTTGTTCATTTGTTACGCGCGATGAAAGAGTATCGGGACATCGTAATTAAGCGTTTTTTGAACGGTGAGCGACCCGGCGATATATTCCGGCTGCTGAAATCGCATGGGGTCAAGCGGAACTTTGTCTACACGACCATCAGGCGATACCGGGAGACGTCCTCGACCAATGACCGTGCGAGATCCGGTCGGCCGCGTTCAGCGAGGACGCCACGGGTCATCAAGATCGTGAGGGAGCGAATTCGGCGCAAAAAGAACCGCTCAATCCGGAAAACGGCTGCAGATCTCAACGTTTCCATTGGAACCGCTCACACCATACTCACCAAGGACCTTGGTTTCAGGCCTTACAAAAAACGTAAGGTCCATGGCGTTTCGGAGGCTACCAGCAAAAAGCGGTTGGATCGAGCTAAGAGGATCCTCTCTCGGCACGCTGGTCAGGAGTTTGTTTTTTCGGACGAGAAACTGTTCGTGCTGCAGCAGCCGCACAATGTGCAAAATGACCGGGTGTGGGCGCCATCGAGGGACAGCATTCCTGAATCCAATATAAACATCCCTCGGTTCCAAAGTGCCGCGTCGGTGATGGTTTGGGGGGCAGTATGCAAACGTGGTAAGCTACCCTTGGTGTTTATTGAAAAAAACGTCAAAATCAACGCGGCGTACTACAAAACTGAGGTTTTGGAAAAGGTTGTTGCCCCCAGTCTCCGAAGCCTCTACGGCGATGAGCACTACGTGTTCCAGCAGGACGGTGCACCAGCCCATACGGCAAATGTGGTTCAAGCCTGGTGTCGGGACAATTTAACCGACTTTCTGGACAAAACTTTGTGGCCTCCCAGCTCTCCGGACTTGAATCCTCTCGACTTTTTTGTTTGGTCCTATATGATGGCGAAGCTGAACGAATACAAGGTCAGCACTTTGGATCATTTCAAGACGGTAATTCTCAAAATCTGGGACGAAATGCCCATGCAGTCCGTGCGTGCCGCCTGCGACGCGTTCGAGAAACGTTTGAAGCTCGTTAAGGAGTACAAAGGGGGGGTCATTCCAAGAGAAATGTTGTAAACGTTCCTTGTAAACATAGCTTTCAATAACTTAAATCCAAAAAATAAAAAAAACATGTTTTCATTTTTTTAACAAATTTTGAAAGTGTATCCGAACTTATTGAACACCGTG

>Ale_MJ1_Clone10

CACGGTGTTCAATAAGTTCGAATACAAGTTTTCATCATTGCGTAGGTATGCGCCATGTACTTATTCTGCATTGGTATTGGTGTCAGCTTTAGCTTGATTCATACGCTACCGAATGTGCCCAGTGTTGACATTCTGTTAATTGTTGTTCATTTGTTACGCGCGATGAAAGAGTATCGGGACATCGTAATTAAGCGTTTTTTGAACGGTGAGCGACCCGGCGATATATTCCGGCTGCTGAAATCGCATGGGGTCAAGCGGAACTTTGTCTACACGACCATCAGGCGATACCGGGAGACGTCCTCGACCAATGACCGTGCGAGATCCGGTCGGCCGCGTTCAGCGAGGACGCCACGGGTCATCAAGATCGTGAGGGAGCGAATTCGGCGCAAAAAGAACCGCTCAATCCGGAAAACGGCTGCAGATCTCAACGTTTCCATTGGAACCGCTCACACCATACTCACCAAGGACCTTGGTTTCAGGCCTTACAAAAAACGTAAGGTCCATGGCGTTTCGGAGGCTACCAGCAAAAAGCGGTTGGATCGAGCTAAGAGGATCCTCTCTCGGCACGCTGGTCAGGAGTTTGTTTTTTCGGACGAGAAACTGTTCGTGCTGCAGCAGCCGCACAATGTGCAAAATGACCGGGTGTGGGCGCCATCGAGGGACAGCATTCCTGAATCCAATATAAACATCCCTCGGTTCCAAAGTGCCGCGTCGGTGATGGTTTAGGGGGCAGTATGCAAACGTGGTAAGCTACCCTTGGTGTTTATTGAAAAAAACGTCAAAATCAACGCGGCGTACTACAAAACTGAGGTTTTGGAAAAGGTTGTTGCCCCCAGTCTCCGAAGCCTCTACGGCGATGAGCACTACGTGTTCCAGCAGGACGGTGCACCAGCCCATACGGCAAATGTGGTTCAAGCCTGGTGTCGGGACAATTTAACCGACTTTCTGGACAAAACTTTGTGGCCTCCCAGCTCCCCGGACTTGAATCCTCTCGACTTTTTTGTTTGGTCCTATATCATGGCGAAGCTGAACGAATACAAGGTCAGCACTTTGGATCATTTCAAGACGGTAATTCTCAAAATCTGGGACGAAATGCCCATACAGTCCGTGCGTGCCGCCTGCGACGCGTTCGAGAAACGTTTGAAGCTCGTTAAGGAGTACAAAGGGGGGGTCATTCCAAGAGAAATGTTGTAAACGTTCCTTGTAAACATAGCTTTCAATAACTTAAATTCAAAAAATAAAAAAAACATGTTTTCATTTTTTTAACAAATTTTGAAAGTGTATCCGAACTTATTGAACACCGTG

>Ale_MJ1_Clone11

CACGGTGTTCAATAAGTTCGAATACAAGTTTTCATCATTGCGTAGGTATGCGCCATGTACTTATTCTGCATTGGTATTGGTGTCAGCTTTAGCTTGATTCATACGCTACCGAATGTGCCCAGTGTTGACATTCTGTTAATTGTTGTTCATTTGTTACGCGCGATGAAAGAGTATCGGGACATCGTAATTAAGCGTTTTTTGAACGGTGAGCGACCCGGCGATATATTCCGGCTGCTGAAATCGCATGGGGTCAAACGGAACTTTGTCTACACGACCATCAGGCGATACCGGGAGACGTCCTCGACCAATGACCGTGCGAGATCCGGTCGGCCGCGTTCAGCGAGGACGCCACGGGTCATCAAGATCGTGAGGGAGCGAATTCGGCGCAAAAAGAACCGCTCAATCCGGAAAACGGCTGCAGATCTCAACGTTTCCATTGGAACCGCTCACACCATACTCACCAAGGACCTTGGTTTCAGGcCTTACAAAAAACGTAAGGTCCATGGCGTTTCGGAGGCTACCAGCAAAAAGCGGTTGGATCGAGCTAAGAGGATCCTCTCTCGGCACGCTGGTCAGGAGTTTGTTTTTTCGGACGAGAAACTGTTCGTGCTGCAGCAGCCGCACAATGTGCAAAATGACCGGGTGTGGGCGCCATCGAGGGACAGCATTCCTGAATCCAATATAAACATCCCTCGGTTCCAAAGTGCCGCGTCGGTGATGGTTTGGGGGGCAGTATGCAAACGTGGTAAGCTACCCTTGGTGTTTATTGGAAAAAACGTCAAAATCAACGCGGCGTACTACAAAACTGAGGTTTTGGAAAAGGTTGTTGCCTCCAGTCTTCGAAGCCTCTACGGCGATGAGCACTACGTGTTCCAGCAGGACGGTGCACCAGCCCATACGGCAAATGTGGTTCAAGCCTGGTGTCGGGACAATTTAACCGACTTTCTGGACAAAACTTTGTGGCCTCCCAGCTCCCCGGACTTGAATCCTCTCGACTTTTTTGTTTGGTCCTATATGATGGCGAAGCTGAACGAATACAAGGTCAGCACTTTGGATCATTTCAAGACGGTAATTCTCAAAATCTGGGACGAAATGCCCATGCAGTCCGTGCGTGCCGCCTGCGACGCGTTCGAGAAACGTTTGAAGCTCGTTAAGGAGTACAAAGGGGGGGTCATTCCAAGAGAAATGTTGTAAACGTTCCTTGTAAACATAGCTTTCAATAACTTAAATCCAAAAAATAAAAAAAAACATGTTTTCATTTTTTTAACAAATTTTGAAAGTGTACCCGAACTTATTGAACACCGTG

>Ale_MJ1_Clone12

CACGGTGTTCAATAAGTTCGAATACAAGTTTTCATCATTGCGTAGGTATGCGCCATGTACTTATTCTGCATTGGTATTGGTGTCAGCTTTAGCTTGATTCATACGCTACCGAATGTGCCCAGTGTTGACATTCTGTTAATTGTTGTTCATTTGTTACGCGCGATGAAAGAGTATCGGGACATCGTAATTAAGCGTTTTTTGAACGGTGAGCGACCCGGCGATATATTCCGGCTGCTGAAATCGCATGGGGTCAAGCGGAACTTTGTCTACACGACCATCAGGCGATACCGGGAGACGTCCTCGACCAATGACCGTGCGAGATCCGGTCGGCCGCGTTCAGCGAGGACGCCACGGGTCATCAAGATCGTGAGGGAGCGAATTCGGCGCAAAAAGAACCGCTCAATCCGGAAAACGGCTGCAGATCTCAACGTTTCCATTGGAACCGCTCACACCATACTCACCAAGGACCTTGGTTTCAGGCCTTACAAAAAACGTAAGGTCCATGGCGTTTCGGAGGCTACCAGCAAAAAGCGGTTGGATCGAGCTAAGAGGATCCTCTCTCGGCACGCTGGTCAGGAGTTTGTTTTTTCGGACGAGAAACTGTTCGTGCTGCAGCAGCCGCACAATGTGCAAAATGACCGGGTGTGGGCGCCATCGAGGGACAGCATTCCTGAATCCAATATAAACATCCCTCGGTTCCAAAGTGCCGCGTCGGTGATGGTTTGGGGGGCAGTATGCAAACGTGGTAAGCTACCCTTGGTGTTTATTGAAAAAAACGTCAAAATCAACGCGGCGTACTACAAAACTGAGGTTTTGGAAAAGGTTGTTGCCCCCAGTCTCCGAAGCCTCTACGGCGATGAGCACTACGTGTTCCAGCAGGACGGTGCACCAGCCCATACGGCAAATGTGGTTCAAGCCTGGTGTCGGGACAATTTAACCGACTTTCTGGACAAAACTTTGTGGCCTCCCAGCTCCCCGGACTTGAATCCTCTCGACTTTTTTGTTTGGTCCTATATGATGGCGAAGCTGAACGAATACAAGGTCAGCACTTTGGATCATTTCAAGACGGTAATTCTCAAAATCTGGGACGAAATGCCCATGCAGTCCGTGCGTGCCGCCTGCGACGCGTTCGAGAAACGTTTGAAGCTCGTTAAGGAGTACAAAGGGGGGGTCATTCCAAGAGAAATGTTGTAAACGTTCCTTGTAAACATAGCTTTCAATAACTTAAATCCAAAAAATAAAAAAAACATGTTTTCATTTTTTTAACAAATTTTGAAAGTGTATCCGAACTTATTGAACACCGTG

>Ale_MJ1_Clone13

CACGGTGTTCAATAAGTTCGAATACAAGTTTTCATCATTGCGTAGGTATGCGCCATGTACTTATTCTGCATTGGTATTGGTGTCAGCTTTAGCTTGATTCATACGCTACCGAATGTGCCCAGTGTTGACATTCTGTTAATTGTTGTTCATTTGTTACGCGCGATGAAAGAGTATCGGGACATCGTAATTAAGCGTTTTTTGAACGGTGAGCGACCCGGCGATATATTCCGGCTGCTGAAATCGCATGGGGTCAAGCGGAACTTTGTCTACACGACCATCAGGCGATACCGGGAGACGTCCTCGACCAATGACCGTGCGAGATCCGGTCGGCCGCGTTCAGCGAGGACGCCACGGGTCATCAAGATCGTGAGGGAGCGAATTCGGCGCAAAAAGAACCGCTCAATCCGGAAAACGGCTGCAGATCTCAACGTTTCCATTGGAACCGCCCACACCATACTCACCAAGGACCTTGGTTTCAGGCCTTACAAAAAACGTAAGGTCCATGGCGTTTCGGAGGCTACCAGCAAAAAGCGGTTGGATCGAGCTAAGAGGATCCTCTCTCGGCACGCTGGTCAGGAGTTTGTTTTTTCGGACGAGAAACTGTTCGTGCTGCAGCAGCCGCACAATGTGCAAAATGACCGGGTGTGGGCGCCATCGAGGGACAGCATTCCTGAATCCAATATAAACATCCCTCGGTTCCAAAGTGCCGCGTCGGTGATGGTTTGGGGGGCAGTATGCAAACGTGGTAAGCTACCCTTGGTGTTTATTGAAAAAAACGTCAAAATCAACGCGGCGTACTACAAAACTGAGGTTTTGGAAAAGGTTGTTGCCCCCAGTCTCCGAAGCCTCTACGGCGATGAGCACTACGTGTTCCAGCAGGACGGTGCACCAGCCCATATGGCAAATGTGGTTCAAGCCTGGTGTCGGGACAATTTAACCGACTTTCTGGACAAAACTTTGTGGCCTCCCAGCTCCCCAGACTTGAATCCTCTCGACTTTTTTGTTTGGTCCTATATGATGGCGAAGCTGAACGAATACAAGGTCAGCACTTTGGATCATTTCAAGACGGTAATTCTCAAAATCTGGGACGAAATGCCCATGCAGTCCGTGCGTGCCGCCTGCGACGCGTTCGAGAAACGTTTGAAGCTCGTTAAGGAGTACAAAGGGGGGGTCATTCCAAGAGAAATGTTGTAAACGTTCCTTGTAAACATAGCTTTCAATAACTTAAATCCAAAAAATAAAAAAAACATGTTTTCATTTTTTTAACAAATTTTGAAAGTGTATCCGAACTTATTGAACACCGTG

>Akl_MJ1_Clone1

CACGGTGTTCAATAAGTTCGAATACAAGTTTTTATCATTGCGTAGGTATGCGCCATGTACTTATTCTGCATTGGTATTGGTGTCAGCTTTAGCTTGATTCATACGCTACCGAATGTGCCCAGTGTTGACAGTCTGTTAATTGTTGTTCATTTGTTACGCGCGATGAAAGAGTATCGGGACATCGTAATTAAGCGTTTTTTGAACGGTGAGCGACCCGGCGATATATTCCGGCTGCTGAAATCGCATGGGGTCAAGCGGAACTTTGTCTACACGACCGTCAGGCGATACCGGGAGACGTCCTCGACCAATGACCGTGCGAGATCCGGTCGGCCGCGTTCAGCGAGGACGCCACGGGTCATCAAGATCGTGAGGGAGCGAATTCGGCGCAAAAAGAACCGCTCAATCCGGAAAACGGCTGCAGATCTCAACGTTTCCATTGGAACCGCTCACACCATACTCACCAAGGACCTTGGTTTCAGGCCTTACAAAAAACGTAAGGTCCATGGCGTTTCGGAGGCTACCAGCAAAAAGCGGTTGGATCGAGCTAAGAGGATCCTCTCTCGGCACGCTGGTCAGGAGTTTGTTTTTTTGGACGAGAAACTGTTCGTGCTGCAGCAGCCGCACAATGTGCAAAATGACCGGGTGTGGGCGCCATCGAGGGACAGCATTCCTGAATCCAATATAAACATCCCTCGGTTCCAAAGTGCCGCGTCGGTGATGGTTTGGGGGGGCAGTATGCAAACGTGGTAAGCTACCCTTGGTGTTTATTGAAAAAAACGTCAAAATCAACGCGGCGTACTACAAAACTGAGGTTTTGGAAAAGGTTGTTGCCCCCAGTCTCCGAAGCCTCTACGGCGATGAGCACTACGTGTTCCAGCAGGACGGTGCACCAGCCCATACGGCAAATGTGGTTCAAGCCTGGTGTCGGGACAATTTAACCGACTTTCTGGACAAAACTTTGTGGCCTCCCAGCTCCCCGGACTTGAATCCTCTCGACTTTTTTGTTTGGTCCTATATGATGGCGAAGCTGAACGAATACAAGGTCAGCACTTTGGATCATTTCAAGACGGTAATTCTCAAAATCTGGGACGAAATGCCCATGCATTTCCGTGCGTGCCGCCTGCGACGCGTTCGAGAAACGTTTGAAGCTCGTTAAGGAGTACAAAGGGGGGGTCATTCCAAGAGAAATGTTGTAAACGTTCCTTGTAAACATAGCTTTCAATAACTTAAATCCAAAAAATAAAAAAAACATGTTTTCATTTTTTTAACAAATTTTGAAAGTGTATCCGAACTTATTGAACACCGTG

>Akl_MJ1_Clone2

CACGGTGTTCAATAAGTTCGAATACAAGTTTTCATCATTGCGTAGGTATGCGCCATGTACTTATTCTGCATTGGTATTGGTGTCAGCTTTAGCTTGATTCATACGCTACCGAATGTGCCCAGTGTTGACAGTCTGTTAATTGTTGTTCATTTGTTACGCGCGATGAAAGAGTATCGGGACATCGTAATTAAGCGTTTTTTGAACGGTGAGCGACCCGGCGATATATTCCGGCTGCTGAAATCGCATGGGGTCAAGCGGAACTTTGTCTACACGACCATCAGGCGATACCGGGAGACGTCCTCGACCAATGACCGTGCGAGATCCGGTCGGCCGCGTTCAGCGAGGACGCCACGGGTCATCAAGATCGTGAGGGAGCGAATTCGGCGCAAAAAGAACCGCTCAATCCGGAAAACGGCTGCAGATCTCAACGTTTCCATTGGAACCGCTCACACCATACTCACCAAGGACCTTGGTTTCAGGCCTTACAAAAAACGTAAGGTCCATGGCGTTTCGGAGGCTACCAGCAAAAAGCGGTTGGATCGAGCTAAGAGGATCCTCTCTCGGCACGCTGGTCAGGAGTTTGTTTTTTCGGACGAGAAACTGTTCGTGCTGCAGCAGCCGCACAATGTGCAAAACCGGGTGTGGGCGCCATCGAGGGACAGCATTCCTGAATCCAATATAAACATCCCTCGGTTCCAAAGTGCCGCGTCGGTGATGGTTTGGGGGGCAGTATGCAAACGTGGTAAGCTACCCTTGGTGTTTATTGAAAAAAACGTCAAAATCAACGCGGCGTACTACAAAACTGAGGCTTTGGAAAAGGTTGTTGCCCCCAGTCTCCGAAGCCTCTACGGCGATGAGCACTACGTGTTCCAGCAGGACGGTGCACCAGCCCATACGGCAAATGTGGTTCAAGCCTGGTGTCGGGACAATTTAACCGACTTTCTGGACAAAACTTTGTGGCCTCCCAGCTCCCCGGACTTGAATCCTCTCGACTTTTTTGTTTGGTCCTATATGATGGCGAAGCTGAACGAATACAAGGTCAGCACTTTGGATCATTTCAAGACGGTAATTCTCAAAATCTGGGACGAAATGCCCATGCAGTCCGTGCGTGCCGCCTGCGACGCGTTCGAGAAACGTTTGAAGCTCGTTAAGGAGTACAAAGGGGGGGTCATTCCAAGAGAAATGTTGTAAACGTTCCTTGTAAACATAGCTTTCAATAACTTAAATCCAAAAAATAAAAAAAACATGTTTTCATTTTTTTAACAAATTTTGAAAGTGTATCCGAACTTATTGAACACCGTG

>Akl_MJ1_Clone3

CACGGTGTTCAATAAGTTCGAATACAAGTTTTCATCATTGCGTAGGTATGCGCCATGTACTTATTCTGCATTGGTATTGGTGTCAGCTTTAGCTTGATTCATACGCTACCGAATGTGCCCAGTGTTGACAGTCTGTTAATTGTTGTTCATTTGTTACGCGCGATGAAAGAGTATCGGGACATCGTAATTAAGCGTTTTTTGAACGGTGAGCGACCCGGCGATATATTCCGGCTGCTGAAATCGCATGGGGTCAAGCGGAACTTTGTCTACACGACCATCAGGCGATACCGGGAGACGTCCTCGACCAATGACCGTGCGAGATCCGGTCGGCCGCGTTCAGCGAGGACGCCACGGGTCATCAAGATCGTGAGGGAGCGAATTCGGCGCAAACAGAACCGCTCAATCCGGAAAACGGCTGCAGATCTCAACGTTTCCATTGGAACCGCTCACACCATACTCACCAAGGACCTTGGTTTCAGGCCTTACAAAAAACGTAAGGTCCATGGCGTTTCGGAGGCTACCAGCAAAAAGCGGTTGGATCGAGCTAAGAGGATCCTCTCTCGGCACGCTGGTCAGGAGTTTGTTTTTTCGGACGAGAAACTGTTCGTGCTGCAGCAGCCGCACAATGTGCAAAATGACCGGGTGTGGGCGCCATCGAGGGACAGCATTCCTGAATCCAATATAAACATCCCTCGGTTCCAAAGTGCCGCGTCGGTGATGGTTTGGGGGGGCAGTATGCAAACGTGGTAAGCTACCCTTGGTATTTATTGAAAAAAACGTCAAAATCAACGCGGCGTACTACGAAACTGAGGTTTTGGAAAAGGTTGTTGCCCCCAGTCTCCGAAGCTTCTACGGCGATGAGCACTACGTGTTCCAGCAGGACGGTGCACCAGCCCATACGGCAAATGTGGTTCAAGCCTGGTGTCGGGACAATTTAACCGACTTTCTGGACAAAACTTTGTGGCCTCCCAGCTCCCCGGACTTGAATCCTCTCGACTTTTTTGTTTGGTCCTATATGATGGCGAAGCTGAACGAATACAAGGTCAGCACTTTGGATCATTTCAAGACGGTAATTCTCAAAATCTGGGACGAAATGCCCATGCAGTCCGTGCGTGCCGCCTGCGACGCGTTCGAGAAACGTTTGAAGCTCGTTAAGGAGTACAAAGGGGGGGTCATTCCAAGAGAAATGTTGTAAACGTTCCTTGTAAACATAGCTTTCAAAAACTTAAATCCAAAAAATAAAAAAAACATGTTTTCATTTTTTTAACAAATTTTGAAAGTGTATCCGAACTTATTGAACACCGTG

>Ahy_MJ1_Clone1

CACGGTGTTCAATAAGTTCGAATACAAGTTTTCATCATTGCGTAGGTATGCGCCATGTACTTATTCTGCATTGGTATTGGTGTCAGCTTTAGCTTGATTCATACGCTACCGAATGTGCCCAGTGTTGACATTCTGTTAATTGTTGTTCATTTGTTACGCGCGATGAAAGAGTATCGGGACATCGTAATTAAGCGTTTTTTGAACGGTGAGCGACCCGGCGATATATTCCGGCTGCTGAAATCGCATGGGGTCAAGCGGAACTTTGTCTACACGACCATCAGGCGATACCGGGAGACGTCCTCGACCAATGACCGTGCGAGATCCGGTCGGCCGCGTTCAGCGAGGACGCCACGGGTCATCAAGATCGTGAGGGAGCGAATTCGGCGCAAAAAGAACCGCTCAATCCGGAAAACGGCTGCAGATCTCAACGTTTCCATTGGAACCGCTCACACCATACTCACCAAGGACCTTGGTTTCAGGCCTTACAAAAAACGTAAGGTCCATGGCGTTTCGGAGGCTACCAGCAAAAAGCGGTTGGATCGAGCTAAGAGGATCCTCTCTCGGCACGCTGGTCAGGAGTTTGTTTTTTCGGACCTCCATTCCTTTTCCATTCCTGAATCCAATATAAACATCCCTCGGTTCCAAAGTGCCGCGTCGGTGATGGTTTGGGGGGCAGTATGCAAACGTGGTAAGCTACCCTTGGTGTTTATTGAAAAAAACGTCAAAATCAACGCGGCGTACTACAAAACTGAGGTTTTGGAAAAGGTTGTTGCCCCCAGTCTCCGAAGCCTCTACGGCGATGAGCACTACGTGTTCCAGCAGGACGGTGCACCAGCCCATACGGCAAATGTGGTTCAAGCCTGGTGTCGGGACAATTTAACCGACTTTCTGGACAAAACTTTGTGGCCTCCCAGCTCCCCGGACTTGAATCCTCTCGACTTTTTTGTTTGGTCCTATATGATGGCGAAGCTGAACGAATACAAGGTCAGCACTTTGGATCATTTCAAGACGGTAATTCTCAAAATCTGGGACGAAATGCCCATGCAGTCCGTGCGTGCCGCCTGCGACGCGTTCGAGAAACGTTTGAAGCTCGTTAAGGAGTACAAAGGGGGGGTCATTCCAAGAGAAATGTTGTAAACGTTCCTTGTAAACATAGCTTTCAATAACTTAAATCCAAAAAATAAAAAAAACATGTTTTCATTTTTTTAACAAATTTTGAAAGTGTATCCGAACTTATTGAACACCGTG

>Ahy_MJ1_Clone2

CACGGTGTTCAATAAGTTCGAATACAAGTTTTCATCATTGCGTAGGTATGCGCCATGTACTTATTCTGCATTGGTATTGGTGTCAGCTTTAGCTTGATTCATACGCTACCGAATGTGCCCAGTGTTGACATTCTGTTAATTGTTGTTCATTTGTTACGCGCGATGAAAGAGTATCGGGACATCGTAATTAAGCGTTTTTTGAACGGTGAGCGACCCGGCGATATATTCCGGCTGCTGAAATCGCATGGGGTCAAGCGGAACTTTGTCTACACGACCATCAGGCGATACCGGGAGACGTCCTCGACCAATGACCGTGCGAGATCCGGTCGGCCGCGTTCAGCGAGGACGCCACGGGTCATCAAGATCGTGAGGGAGCGAATTCGGCGCAAAAAGAACCGCTCAATCCGGAAAACGGCTGCAGATCTCAACGTTTCCATTGGAACCGCTCACACCATACTCACCAAGGACCTTGGTTTCAGGCCTTACAAAAAACGTAAGGTCCATGGCGTTTCGGAGGCTACCAGCAAAAAGCGGTTGGATCGAGCTAAGAGGATCCTCTCTCGGCACGCTGGTCAGGAGTTTGTTTTTTCGGACCTCCATTCCTTTTCCATTCCTGAATCCAATATAAACATCCCTCGGTTCCAAAGTGCCGCGTCGGTGATGGTTTGGGGGGCAGTATGCAAACGTGGTAAGCTACCCTTGGTGTTTATTGAAAAAAACGTCAAAATCAACGCGGCGTACTACAAAACTGAGGTTTTGGAAAAGGTTGTTGCCCCCAGTCTCCGAAGCCTCTACGGCGATGAGCACTACGTGTTCCAGCAGGACGGTGCACCAGCCCATACGGCAAATGTGGTTCAAGCCTGGTGTCGGGACAATTTAACCGACTTTCTGGACAAAACTTTGTGGCCTCCCAGCTCCCCGGACTTGAATCCTCTCGACTTTTTTGTTTGGTCCTATATGATGGCGAAGCTGAACGAATACAAGGTCAGCACTTTGGATCATTTCAAGACGGTAATTCTCAAAATCTGGGACGAAATGCCCATGCAGTCCGTGCGTGCCGCCTGCGACGCGTTCGAGAAACGTTTGAAGCTCGTTAAGGAGTACAAAGGGGGGGTCATTCCAAGAGAAATGTTGTAAACGTTCCTTGTAAACATAGCTTTCAATAACTTAAATCCAAAAAATAAAAAAAACATGTTTTCATTTTTTTAACAAATTTTGAAAGTGTATCCGAACTTATTGAACACCGTG

>Ahy_MJ1_Clone3

CACGGTGTTCAATAAGTTCGAATACAAGTTTTCATCATTGCGTAGGTATGCGCCATGTACTTATTCTGCATTGGTATTGGTGTCAGCTTTAGCTTGATTCATACGCTACCGAATGTGCCCAGTGTTGACATTCTGTTAATTGTTGTTCATTTGTTACGCGCGATGAAAGAGTATCGGGACATCGTAATTAAGCGTTTTTTGAACGGTGAGCGACCCGGCGATATATTCCGGCTGCTGAAATCGCGTGGGGTCAAGCGGAACTTTGTCTACACGACCATCAGGCGATACCGGGAGACGTCCTCGACCAATGACCGTGCGAGATCCGGTCGGCCGCGTTCAGCGAGGACGCCACGGGTCATCAAGATCGTGAGGGAGCGAATTCGGCGCAAAAAGAACCGCTCAATCCGGAAAACGGCTGCAGATCTCAACGTTTCCATTGGAACCGCTCACACCATACTCACCAAGGACCTTGGTTTCAGGCCTTACAAAAAACGTAAGGTCCATGGCGTTTCGGAGGCTACCAGCAAAAAGCGGTTGGATCGAGCTAAGAGGATCCTCTCTCGGCACGCTGGTCAGGAGTTTGTTTTTTCGGACGAGAAACTGTTCGTGCTGCAGCAGCCGCACAATGTGCAAAATGACCGGGTGTGGGCGCCATCGAGGGACAGCATTCCTGAATCCAATATAAACATCCCTCGGTTCCAAAGTGCCGCGTCGGTGATGGTTTGGGGGGCAGTATGCAAACGTGGTAAGCTACCCTTGGTGTTTATTGAAAAAAACGTCAAAATCAACGCGGCGTACTACAAAACTGAGGTTTTGGAAAAGGTTGTTGCCCCCAGTCTCCGAAGCCTCTACGGCGATGAACACTACGTGTTCCAGCAGGACGGTGCACCAGCCCATACGGCAAATGTGGTTCAAGCCTGGTGTCGGGACAATTTAACCGACTTTCTGGACAAAACTTTGTGGCCTCCCAGCTCCCCGGACTTGAATCCTCTCGACTTTTTTGTTTGGTCCTATATGATGGCGAAGCTGAACGAATACAAGGTCAGCACTTTGGATCATTTCAAGACGGTAATTCTCAAAATCTGGGACGAAATGCCCATGCAGTCCGTGCGTGCCGCCTGCGACGCGTTCGAGAAACGTTTGAAGCTCGTTAAGGAGTACAAAGGGGGGGTCATTCCAAGAGAAATGTTGTAAACGTTCCTTGTAAACATAGCTTTCAATAACTTAAATCCAAAAAATAAAAAAAACATGTTTTCATTTTTTTAACAAATTTTGAAAGTGTATCCGAACTTATTGAACACCGTG

>Aba_MJ1_Clone1

CACGGTGTTCAATAAGTTCGAATACAAGTTTTCATCATTGCGTAGGTATGCGCCATGTACTTATTCTGCATTGGTATTGGTGTCAGCTTTAGCTTGATTCATACGCTACCGCATGTGCCCAGTGTTGACAGTCTGTTAATTGTTGTTCATTTGTTACGCGCGATGAAAGAGTATCGGGACATCGTAATTAAGCGTTTTTTGAACGGTGAGCGACCCGGCGATATATTCCGGCTGCTGAAATCGCATGGGGTCAAGCGGAACTTTGTCTACACGACCATCAGGCGATACCGGGAGACGTCTTCGACCAATGACCGTGCGAGATCCGGTCGGCCGCGTTCAGCGAGGACGCCACGGGTCATCAAGATCGTGAGGGAGCGAATTCGGCGCAAAAAGAACCGCTCAATCCGGAAAACGGCTGCAGATCTCAACGTTTCCATTGGAACCGCTCACACCATACTCACCAAGGACCTTGGTTTCAGGCCTTACAAAAAACGTAAGGTCCATGGCGTTTCGGAGGCTACCAGCAAAAAGCGGTTGGATCGAGCTAAGAGGATCCTCTCTCGGCACGCTGGTCAGGAGTTTGTTTTTTCGGACGAGAAACTGTTCGTGCTGCAGCAGCCGCACAATGTGCAAAATGACCGGGTGTGGGCGCCATCGAGGGACAGCATTCCTGAATCCAATATAAACATCCCTCGGTTCCAAAGTGCCGCGTCGGTGATGGTTTGGGGGGCAGTATGCATACGTGGTAAGCTACCCTTGGTGTTTATTGAAAAAAACGTCAAAATCAACGCGGCGTACTACAAAACTGAGGTTTTGGAAAAGGTTGTTGCCCCCAGTCTCCGAAGCCTCTACGGCGATGAGCACTACGTGTTCCAGCAGGACGGTGCACCAGCCCATACGGCAAATGTGGTTCAAGCCTGGTGTCGGGACAATTTAACCGACTTTCTGGACAAAACTTTGTGGCCTCCCAGCTCCCCGGACTTGAATCCTCTCGACTTTTTTGTTTGGTCCTATATGATGGCGAAGCTGAACGAATACAAGGTCAGCACTTTGGATCATTTCAAGACGGTAATTCTCAAAATCTGGGACGAAATGCCCATGCAGTCCGTGCGTGCCGCCTGCGACGCGTTCGAGAAACGTTTGAAGCTCGTTAAGGAGTACAAAGGGGGGGTCATTCCAAGAGAAATGTTGTAAACGTTCCTTGTAAACATAGCTTTCAATAACTTAAATCCAAAAAATAAAAAAAACATGTTTTCATTTTTTTAACAAATTTTGAAAGTGTATCCGAACTTATTGAACACCGTG

>Aba_MJ1_Clone2

CACGGTGTTCAATAAGTTCGAATACAAGTTTTCATCATTGCGTAGGTATGCGCCATGTACTTATTCTGCATTGGTATTGGTGTCAGCTTTAGCTTGATTCATACGCTACCGAATGTGCCCAGTGTTGACAGTCTGTTAATTGTTGTTCATTTGTTACGCGCGATGAAAGAGTATCGGGACATCGTAATTAAGCGTTTTTTGAACGGTGAGCGACCCGGCGATATATTCCGGCTGCTGAAATCGCATGGGGTCAAGCGGAACTTTGTCTACACGACCATCAGGCGATACCGGGAGACGTCCTCGACCAATGACCGTGCGAGATCCGGTCGGCCGCGTTCAGCGAGGACGCCACGGGTCATCAAGATCGTGAGGGAGCGAATTCGGCGCAAAAAGAACCGCTCAATCCGGAAAACGGCTGCAGATCTCAACGTTTCCATTGGAACCGCTCACACCATACTCACCAAGGACCTTGGTTTCAGGCCTTACAAAAAACGTAAGGTCCATGGCGTTTCGGAGGCTACCAGCAAAAAGCGGTTGGATCGAGCTAAGAGGATCCTCTCTCGGCACGCTGGTCAGGAGTTTGTTTTTTCGGACGAGAAACTGTTCGTGCTGCAGCAGCCGCACAATGTGCAAAATGACCGGGTGTGGGCGCCATCGAGGGACAGCATTCCTGAATCCAATATAAACATCCCTCGGTTCCAAAGTGCCGCGTCGGTGATGGTTTGGGGGGCAGTATGCAAACGTGGTAAGCTACCCTTGGTGTTTATTGAAAAAAaCGTCAAATGTGCGGCAAATGTGGTTCAAGCCTGGTGTCGGGACAATTTAACCGACTTTCTGGACAAAACTTTGTGGCCTCCCAGCTCCCCGGACTTGAATCCTCTCGACTTTTTTGTTTGGTCCTATATGATGGCGAAGCTGAACGAATACAAGGTCAGCACTTTGGATCATTTCAAGACGGTAATTCTCAAAATCTGGGACGAAATGCCCATGCAGTCCGTGCGTGCCGCCTGCGACGCGTTCGAGAAACGTTTGAAGCTCGTTAAGGAGTACAAAGGGGGGGAATCCAAGAGAAATGTTGTAAACGTTCCTTGTAAACATAGCTTTCAATAACTTAAATCCAAAAAATAAAAAAAACATGTTTTCATTTTTTTAACAAATTTTGAAAGTGTATCCGAACTTATTGAACACCGTG

>Aba_MJ1_Clone3

CACGGTGTTCAATAAGTTCGAATACAAGTTTTTATCATTGCGTAGGTATGCGCCATGTACTTATTCTGCATTGGTATTGGTGTCAGCTTTAGCTTGATTCATACGCTACCGAATGTGCCCAGTGTTGACAGTCTGTTAATTGTTGTTCATTTGTTACGCGCGATGAAAGAGTATCGGGACATCGTAATTAAGCGTTTTTTGAACGGTGAGCGACCCGGCGATATATTCCGGCTGCTGAAATCGCATGGGGTCAAGCGGAACTTTGTCTACACGACCATCAGGCGATACCGGGAGACGTCCTCGACCAATGACCGTGCGAGATCCGGTCGGCCGCGTTCAGCGAGGACGCCACGGGTCATCAAGATCGTGAGGGAGCGAATTCGGCGCAAAAAGAACCGCTCAATCCGGAAAACGGCTGCAGATCTCAACGTTTCCATTGGAACCGCTCACACCATACTCACCAAGGACCTTGGTTTCAGGCCTTACAAAAAACGTAAGGTCCATGGCGTTTCGGAGGCTACCAGCAAAAAGCGGTTGGATCGAGCTAAGAGGATCCTCTCTCGGCACGCTGGTCAGGAGTTTGTTTTTTCGGACGAGAAACTGTTCGTGCTACAGCAGCCGCACAATGTGCAAAATGACCGGGTGTGGGCGCCATCGAGGGACAGCATTCCTAAATCCAATATAAACATCCCTCGGTTCCAAAGTGCCGCGTCGGTGATGGTTTGGGGGGGCAGTATGCAAACGTGGTAAGCTACCCTTGGTGTTTATTGAAAAAAACGTCAAAATCAACGCGGCGTACTACAAAACTGAGGTTTTGGAAAAGGTTGTTGCCCCCAGTCTCCGAAGCCTCTACGGCGATGAGCACTACGTGTTCCAGCAGGACGGTGCACCAGCCCATACGGCAAATGTGGTTCAAGCCTGGTGTCGGGACAATTTAACCGACTTTCTGGACAAAACTTTGTGGCCTCCCAGCTCCCCGGACTTGAATCCTCTCGACTTTTTTGTTTGGTCCTATATGATGGCGAAGCTGAACGAATACAAGGTCAGCACTTTGGATCATTTCAAGACGGTAATTCTCAAAATCTGGGACGAAATGCCCATGCAGTCCGTGCGTGCCGCCTGCGACGCGTTCGAGAAACGTTTGAAGCTCGTTAAGGAGTACAAAGGGGGGGTCATTCCAAGAGAAATGTTGTAAACGTTCCTTGTAAACATAGCTTTCAATAACTTAAATCCAAAAAATAAAAAAAACATGTTTTCATTTTTTTAACAAATTTTGAAAGTGTATCCGAACTTATTGAACACCGTG

>Aya_MJ1_Clone1

CACGGTGTTCAATAAGTTCGAATACAAGTTTTCATCATTGCGTAGGTATGCGCCATGTACTTATTCTGCATTGGTATTGGTGTCAGCTTTAGCTTGATTCATACGCTACCGAATGTGCCCAGTGTTGACAGTCTGTTAATTGTTGTTCATTTGTTACGCGCGATGAAAGAGTATCGGGACATCGTAATTAAGCGTTTTTTGAACGGTGAGCGACCCGGCGATATATTCCGGCTGCCGAAATCGCATGGGGTCAAGCGGAACTTTGTCTACACGACCATCAGGCGATACCGGGAGACGTCCTCGACCAATGACCGTGCGAGATCCGGTCGGCCGCGTTCAGCGAGGACGCCACGGGTCATCAAGATCGTGAGGGAGCGAATTCGGCGCAAAAAGAACCGCTCAATCCGGAAAACGGCTGCAGATCTCAACGTTtCCAtTGGAACCGCTCACACCATACTCACCAAGGACCTTGGTTTCAGGCCTTACAAAAAACGTAAGGTCCATGGCGTTTCGGAGGCTACCAGCAAAAAGCGGTTGGATCGAGCTAAGAGGATCCTCTCTCGGCACGCTGGTCAGGAGTTTGTTTTTTCGGACGAGAAACTGTTCGTGCTGCAGCAGCCGCACAATGTGCAAAATGACCGGGTGTGGGCGCCATCGAGGGACAGCATTCCTGAATCCAATATAAACATCCCTCGGTTCCAAAGTGCCGCGTCGGTGATGGTTTGGGGGGCAGTATGCAAACGTGGTAAGCTACCCTTGGTGTTTATTGAAAAAAACGTCAAAATCAACGCGGCGTACTACAAAACTGAGGTTTTGGAAAAGGTTGTTGCCCCCAGTCTCCGAAGCCTCTACGGCGATGAGCACTACGTGTTCCAGCAGGACGGTGCACCAGCCCATACGGCAAATGTGGTTCAAGCCTGGTGTCGGGACAATTTAACCGACTTTCTGGACAAAACTTTGTGGCCTCCCAGCTCCCCGGACTTGAATCCTCTCGACTTTTTTGTTTGGTCCTATATGATGGCGAAGCTGAACGAATACAAGGTCAGCACTTTGGATCATTTCAAGACGGTAATTCTCAAAATCTGGGACGAAATGCCCATGCGGTCCGTGCGTGCCGCCTGCGACGCGTTCGAGAAACGTTTGAAGCTCGTTAAGGAGTACAAAGGGGGGGTCATTCCAAGAGAAATGTTGTAAACGTTCCTTGTAAACATAGCTTTCAATAACTTAAATCCAAAAAATAAAAAAAACATGTTTTCATTTTTTTAACAAATTTTGAAAGTGTATCCGAACTTATTGAACACCGTG

>Aya_MJ1_Clone2

CACGGTGTTCAATAAGTTCGAATACAAGTTTTCATCATTGCGTAGGTATGCGCCATGTACTTATTCTGCATTGGTATTGGTGTCAGCTTTAGCTTGATTCATACGCTACCGAATGTGCCCAGTGTTGACATTCTGTTAATTGTTGTTCATTTGTTACGCGCGATGAAAGAGTATCGGGACATCGTAATTAAGCGTTTTTTGAACGGTGAGCGACCCGGCGATATATTCCGGCTGCTGAAATCGCATGGGGTCAAGCGGAACTTTGTCTACACGACCATCAGGCGATACCGGGAGACGTCCTCGACCAATGACCGTGCGAGATCCGGTCGGCCGCGTTCAGCGAGGACGCCACGGGTCATCAAGATCGTGAGGGAGCGAATTCGGCGCAAAAAGAACCGCTCAATCCGGAAAACGGCTGCAGATCTCAACGTTTCCATTGGAACCGCTCACACCATACTCACCAAGGACCTTGGTTTCAGGCCTTACAAAAAACGTAAGGTCCATGGCGTTTCGGAGACTACCAGCAAAAAGCGGTTGGATCGAGCTAAGAGGATCCTCTCTCGGCACGCTGGTCAGGAGTTTGTTTTTTCGGACGAGAAACTGTTCGTGCTGCAGCAGCCGCACAATGTGCAAAATGACCGGGTGTGGGCGCCATCGAGGGACAGCATTCCTGAATCCAATATAAACATCCCTCGGTTCCAAAGTGCCGCGTCGGTGATGGTTTGGGGGGCAGTATGCAAACGTGGTAAGCTACCCTTGGTGTTTATTGAAAAAAACGTCAAAATCAACGCGGCGTACTACAAAACTGAGGTTTTGGAAAAGGTTGTTGCCCCCAGTCTCCGAAGCCTCTACGGCGATGAGCACTACGTGTTCCAGCAGGACGGTGCACCAGCCCATACGGCAAATGTGGTTCAAGCCTGGTGTCGGGACAATTTAACCGACTTTCTGGACAAAACTTTGTGGCCTCCCAGCTCCCCGGACTTGAATCCTCTCGACTTTTTTGTTTGGTCCTATATGATGGCGAAGCTGAACGAATACAAGGTCAGCACTTTGGATCATTTCAAGACGGTAATTCTCAAAATCTGGGACGAAATGCCCATGCAGTCCGTGCGTGCCGCCTGCGACGCGTTCGAGAAACGTTTGAAGCTCGTTAAGGAGTACAAAGGGGGGGTCATTCCAAGAGAAATGTTGTAAACGTTCCTTGTAAACATAGCTTTCAATAACTTAAATCCAAAAAATAAAAAAAACATGTTTTCATTTTTTTAACAAATTTTGAAAGTGTATCCGAACTTATTGAACACCGTG

>Aya_MJ1_Clone3

CACGGTGTTCAATAAGTTCGAATACAAGTTTTCATCATTGCGTAGGTATGCGCCATGTACTAATTCTGCATTGGTATTGGTGTCAGCTTTAGCTTGATTCATACGCTACCGAATGTGCCCAGTGTTGACAGTCTGTTAATTGTTGTTCATTTGTTACGCGCGATGAAAGAGTATCGGGACATCGTAATTAAGCGTTTTTTGAACGGTGAGCGACCCGGCGATATATTCCGGCTGCTGAAATCGCATGGGGTCAAGCGGAACTTTGTCTACACGACCATCAGGCGATACCGGGAGACGTCCTCGACCAATGACCGTGCGAGATCCGGTCGGCCGCGTTCAGCGAGGACGCCACGGGTCATCAAGATCGTGAGGGAGCGAATTCGGCGCAAAAAGAACCGCTCAATCCGGAAAACGGCTGCAGATCTCAACGTTTCCATTGGAACCGCTCACACCATACTCACCAAGGACCTTGGTTTCAGGCCTTACAAAAAACGTAAGGTCCATGGCGTTTCGGAGGCTACCAGCAAAAAGCGGTTGGATCGAGCTAAGAGGATCCTCTCTCGGCACGCTGGTCAGGAGTTTGTTTTTTCGGACGAGAAACTGTTCGTGCTGCAGCAGCCGCACAATGTGCAAAATGACCGGGTGTGGGCGCCATCGAGGGACAGCATTCCTGAATCCAATATAAACATCCCTCGGTTCCAAAGTGCCGCGTCGGTGATGGTTTGGGGGGCAGTATGCAAACGTGGTAAGCTACCCTTGGTGTTTATTGAAAAAAACGTCAAAATCAACGCGGCGTACTACAAAACTGAGGTTTTGGAAAAGGTTGTTGCCCCCAGTCTCCGAAGCCTCTACGGCGATGAGCACTACGTGTTCCAGCAGGACGGTGCACCAGCCCATACGGCAAATGTGGTTCAAGCCTGGTGTCGGGACAATTTAACCGACTTTCTGGACAAAACTTTGTGGCCTCCCAGCTCCCCGGACTTGAATCCTCTCGACTTTTTTGTTTGGTCCTATATGATGGCGAAGCTGAACGAATACAAGGTCAGCACTTTGGATCATTTCAAGACGGTAATTCTCAAAATCTGGGACGAAATGCCCATGCAGTCCGTGCGTGCCGCCTGCGACGCGTTCGAGAAACGTTTGAAGCTCGTTAAGGAGTACAAAGGGGGGGTCATTCCAAGAGAAATGTTGTAAACGTTCCTTGTAAACATAGCTTTCAATAACTTAAATCCAAAAAATAAAAAAAACATGTTTTCATTTTTTTAACAAATTTTGAAAGTGTATCCGAACTTATTGAACACCGTG

>Acr_MJ1_Clone1

CACGGTGTTCAATAAGTTCGAATACAAGTTTTCATCATTGCGTAGGTATGCGCCATGTACATATTCTGAATTGTTATTGGTGTCAGCTTTAGCTTCATTCATACGCTACCGAATGTGCGCGGTGTTGACATTCTGTTAGTTGTTGTTCGTTTGTTACGCGCGATGAAAGAGTATCGGGACTTCGTAATTAAGCGTTTTTTGAACGGTGAGCGACCCGGCGATATATTCCGGCTGCTGAAATCGCATGGGGTCAAGCGGAACTTTGTCTACACGACCATCAGGCGATACCGGGAGACGTCCTCGACCAATGACCGTGCGAGATCCGGTCGGACGCCTTAAGCGAGGACGCCACGGGTCATCAAGATCGTGAGGGAGCGAATTCGGCGCAAAAAGAACCGCTCAATCCGGAAAACGGCTGCAGATCTCAACGTTTCCATTGGAACCGCTCACACCATACTCACCAAGGACCTTGGTTTCAGGCCTTACAAAAAACGTAAGGTCCATGGCGTTTCGGAGGCTACCAGCAAAAAGCGGTTGGATCGAGCTAAGAGGATCCTCTCTCGGCACGCTGGTCAGGAGTTTGTTTTTTCGTACGAGAAACTGTTCGTGCTGCAGCAGCCGCACAATGTGCAAAATGACCGGGTGTGGGCCCCATCGAGGGACAGCATTCCTGAATCCAATATAAACATCCCTCGGTTCCAAAGTGCCGCGTCGGTGGTGGTTTGGGGGGCAGTATGCAAACGTGGTAAGCTACCCTTGGTGTTTATTGAAAAAAACGTCAAAATCAACGCGGCGTACTACAAAACTGAGGTTTTGGAAAAGGTTGTTGCCCCCAGTCTCCGAAGCCTCTACGGCGATGAGCACTACGTGTTCCAGCAGGACGGTGCACCAGCCCATACGGCAAATGTGGTTCAAGCCTGCTGTCGGGACAATTTAACCGACTTTCTGGACAAAACTTTGTGGCCTCCCAGCTCCCCGGACTTGAATCCTCTCGACTTTTTTGTTTGGTCCTATATGATGGCGAAGCTGAACGAATACAAGGTCAGCACTTTGGATCATTTCAAGACAGTAATTCTCGAAATCTGGGACGAAATGCCCATGCAGTCCGTGCGTGCCGCCTGCGACGCGTTCGAGAAACGTTTGAAGCTCGTTAAGGAGTACAAAGGGGGGGGGGTCATTCCAAGAGAAATGTTGTAAACGTTCCTTGTAAACATAGCTTTCAATAACATAAATCCAAAAAATAAAAAAACATGTTTTCATTTTTTTAACAAATTTTGAAAGTGTATCCGAACTTATTGAACACCGTG

>Acr_MJ1_Clone2

CACGGTGTTCAATAAGTTCGAATACAAGTTTTCATCATTGCGTAGGTATGCGCCATGTACATATTCTGCATTGTTATTGGTGTCAACTTTTAGCTTCATTCATACGCTACCGAATGTGCACGGTGTTGACATTCTGTTAGTTGTTGTTCGTTTGTTACGCGCGATGAAAGAGTATCGGGACTTCGTAATTAAGCGTTTTTTGAACGGTGAGCGACCCGGCGATATATTCCGGCTGCCGAAATCGCATGGGGTCAAGCGGAACTTTGTCTACACGACCATCAGGCGATACCGGGAGACGTCCTCGACCAATGACCGTGCGAGATCCGGTCGGCCGCCTTAAGCGAGGACGCCACGGGTCATCAAGATCGTGAGGGAACGAATTCGGCGCAAAAAGAACCGCTCAATCCGGAAAACGGCTGCAGATCTCAACGTTTCCATTGGAACCGCTCACACCATACTCACCAAGGACCTTGGTTTCAGGCCTTACAAAAAACCTAAGGTCCATGGCGTTTCGGAGGCTACCAGCAAAAAGCGGTTGGATCGAGCTAAGAGGATCCTCTCTCGGCACGCTGGTCAGGAGTTTGTTTTTTCGGACGAGAAACTGTTCGTGCTGCAGCAGCCGCACAATGTGCAAAATGACCGGGTGTGGGCGCCATCGAGGGACAGCATTCCTGAATCCAATATAAACATCCCTCGGTTCCAAAGTGCCGCGTCGGTGGTGGTTTGGGGGGCAGTATGCAAACGTGGTAAGCTACCCTTGGTGTTTATTGAAAAAAACGTCAAAATCAAAGCGGCGTACTACAAAACTGAGGTTTTGGAAAAGGTTGTTGCCCCCAGTCTCCGAAGCCTCTACGGCGATGAGCACTACGTGTTCCAGCAGGACGGTGCACCAGCCCATACGGCAAATGTGGCTCAAGCCTGGTGTCGGGACAATTTAACCGACTTTCTGGACAAAACTTTGTGGCCTCCTAGCTCCCCGGACTTGAATCCTCTCGACTTTTTTGTTTGGTCCTATATGATGGCGAAGCTGAACGAATACAAGGTCAGCACTTTGGATCATTTCAAGACGGTAATTCTCGAAATCTGGGACGAAATGCCCATGCAGTCCGTGCGTGCCGCCTGCGACGCGTTCGAGAAACATTTGAAGCTCGTTAAGGAGTACAAAGGGGGGGTCATTCCAAGAGAAATGTTGTAAACGTTCCTTGTAAACATAGCTTTCAATAACATAAATCCAAAAAATAAAAAAAACATGTTTTCATTTTTTTAACAAATTTTGAAAGTGTATCCGAACTTATTGAACACCGTG

>Acr_MJ1_Clone3

CACGGTGTTCAATAAGTTCGAATACAAGTTTTCATCATTGCGTAGGTATGCGCCATGTACATATTCTGCATTGTTATTGGTGTCAACTTTTAGCTTCATTCATACGCTACCGAATGTGCACGGTGTTGACATTCTGTTAGTTGTTGTTCGTTTGTTACGCGCGATGAAAGAGTATCGGGACTTCGTAATTAAGCGTTTTTTGAACGGTGAGCGACCCGGCGATATATTCCGGCTGCCGAAATCGCATGGGGTCAAGCGGAACTTTGTCTACACGACCATCAGGCGATACCGGGAGACGTCCTCGACCAATGACCGTGCGAGATCCGGTCGGCCGCCTTAAGCGAGGACGCCACGGGTCATCAAGATCGTGAGGGAACGAATTCGGCGCAAAAAGAACCGCTCAATCCGGAAAACGGCTGCAGATCTCAACGTTTCCATTGGAACCGCTCACACCATACTCACCAAGGACCTTGGTTTCAGGCCTTACAAAAAACCTAAGGTCCATGGCGTTTCGGAGGCTACCAGCAAAAAGCGGTTGGATCGAGCTAAGAGGATCCTCTCTCGGCACGCTGGTCAGGAGTTTGTTTTTTCGGACGAGAAACTGTTCGTGCTGCAGCAGCCGCACAATGTGCAAAATGACCGGGTGTGGGCGCCATCGAGGGACAGCATTCCTGAATCCAATATAAACATCCCTCGGTTCCAAAGTGCCGCGTCGGTGGTGGTTTGGGGGGCAGTATGCAAACGTGGTAAGCTACCCTTGGTGTTTATTGAAAAAAACGTCAAAATCAAAGCGGCGTACTACAAAACTGAGGTTTTGGAAAAGGTTGTTGCCCCCAGTCTCCGAAGCCTCTACGGCGATGAGCACTACGTGTTCCAGCAGGACGGTGCACCAGCCCATACGGCAAATGTGGCTCAAGCCTGGTGTCGGGACAATTTAACCGACTTTCTGGACAAAACTTTGTGGCCTCCTAGCTCCCCGGACTTGAATCCTCTCGACTTTTTTGTTTGGTCCTATATGATGGCGAAGCTGAACGAATACAAGGTCAGCACTTTGGATCATTTCAAGACGGTAATTCTCGAAATCTGGGACGAAATGCCCATGCAGTCCGTGCGTGCCGCCTGCGACGCGTTCGAGAAACATTTGAAGCTCGTTAAGGAGTACAAAGGGGGGGTCATTCCAAGAGAAATGTTGTAAACGTTCCTTGTAAACATAGCTTTCAATAACATAAATCCAAAAAATAAAAAAAACATGTTTTCATTTTTTTAACAAATTTTGAAAGTGTATCCGAACTTATTGAACACCGTG

>Akw_MJ1_Clone1

CACGGTGTTCAATAAGTTCGAATACAAGTTTTCATCATTGCGTAGGTATGCGCCATGTACTTATTCTGCATTGGTATCGGTGTCAGCTTTAGCTTGATTCATACGCTACCGAATGTGCCCAGTGTTGACAGTCTGTTAATTGTTGTTCATTTGTTACGCGCGATGAAAGAGTATCGGGACATCGTAATTAAGCGTTTTTTGAACGGTGAGCGACCCGGCGATATATTCCGGCTGCTGAAATCGCATGGGGTCAAGCGGAACTTTGTCTACACGACCATCAGGCGATACCGGGAGACGTCCTCGACCAATGACCGTGCGAGATCCGGTCGGCCGCGTTCGGCGAGGACGCCACGGGTCATCAAGATCGTGAGGGAGCGAATTCGGCGCAAAAAGAACCGCTCAATCCGGAAAACGGCTGCAGATCTCAACGTTTCCATTGGAACCGCTCACACCATACTCACCAAGGACCTTGGTTTCAAGCCTTACAAAAAACGTAAGGTCCATGGCGTTTCGGAGGCTACCAGCAAAAAGCGGTTGGATCGAGCTAAGAGGATCCTCTCTCGGCACGCTGGTCAGGAGTTTGTTTTTTCGGACGAGAAACTGTTCGTGCTGCAGCAGCCGCACAATGTGCAAAATGACCGGATGTGGGCGCCATCGAGGGACAGCATTCCTGAATCCAATATAAACATCCCTCGGTTCCAAAGTGCCGCGTCGGTGATGGTTTGGGGGGCAGTATGCAAACGTGGTAAGCTACCCTTGGTGTTTATTGAAAAAAACGTCAAAATCAACGCGGCGTACTACAAAACTGAGGTTTTGGAAAAGGTTGTTGCCCCCAGTCTCCGAAGCCTCTACGGCGATGAGCACTACGTGTTCCAGCAGGACGGTGCACCAGCCCATACGGCAAATGTGGTTCAAGCCTGGTGTCGGGACAATTTAACCGACTTTCTGGACAAAACTTTGGGACCTCCCAGCTTCCCGGACTTGAATCCTCTCGACTTTTTTGTTTGGTCCTATATGATGGCGAAGCTGAACGAATACAAGGTCAGCACTTTGGATCATTTCAAGACGGTAATTCTCAAAATCTGGGACGAAATGCCCATGCAGTCCGTGCGTGCCGCCTGCGACGCGTTCGAGAAACGTTTGAAGCTCGTTAAGGAGTACAAAGGGGGGGTCATTCCAAGAGAAATGTTGTAAACGTTCCTTGTAAACATAGCTTTCAATAACTTAAATCCAAAAAATAAAAAAAACATGTTTTCATTTTTTTAACAAATTTTGAAAGTGTATCCGAACTTATTGAACACCGTG

>Akw_MJ1_Clone2

CACGGTGTTCAATAAGTTCCAATACAAGTTTTCATCATTGCGTAGGTATGCGCCATGTACTTATTCTGCATTGGTATTGGTGTCAGCTTTAGCTTGATTCATACGCTACCGAATGTGCCCAGTGTTGACAGTCTGTTAATTGTTGTTCATTTGTTACGCGCGATGAAAGAGTATCGGGACATCGTAATTAAGCGTTTTTTGAACGGTGAGCGACCCGGCGATATATTCCGGCTGCTGAAATCGCATGGGGTCAAGCGGAACTTTGTCTACACGACCATCAGGCGATACCGGGAGACGTCCTCGACCAATGACCGTGCGAGATCCGGTCGGCCGCGTTCAGCGAGGACGCCACGGGTCATCAAGATCGTGAGGGAGCGAATTCGGCGCAAAAAGAACCGCTCAATCCGGAAAACGGCTGCAGATCTCAACGTTTCCATTGGAACCGCTCACACCATACTCACCAAGGACCTTGGTTTCAGGCCTTACAAGAAACGTAAGGTCCATGGCGTTTCGGAGGCTACCAGCAAAAAGCGGTTGGATCGAGCTAAGAGGATCCTCTCTCGGCACGCTGGTCAGGAGTTTGTTTTTTCGGACGAGAAACTGTTCGTGCTGCAGCAGCCGCACAATGTGCAAAATGACCGGGTGTGGGCGCCATCGAGGGACAGCATTCCTGAATCCAATATAAACATCCCTCGGTTCCAAAGTGCCGCGTCGGTGATGGTTTGGGGGGCAGTATGCAAACGTGGTAAGCTACCCTTGGTGTTTATTGAAAAAAACGTCAAAATCAACGCGGCGTACTACAAAACTGAGGTTTTGGAAAAGGTTGTTGCCCCCAGTCTCCGAAGCCTCTACGGCGATGAGCACTACGTGTTCCAGCAGGACGGTGCACCAGCCCATACGGCAAATGTGGTTCAAGCCTGGTGTCGGGACAATTTAACCGACTTTCTGGACAAAACTTTGTGGCCTCCCAGCTCCCCGGACTTGAATCCTCTCGACTTTTTTGTTTGGTCCTATATGATGGCGAAGCTGAACGAATACAAGGTCAGCACTTTGGATCATTTCAAGACGGTAATTCTCAAAATCTGGGACGAAATGCCCATGCAGTCCGTGCGTGCCGCCTGCGACGCGTTCGAGAAACGTTTGAAGCTCGTTAAGGAGTACAAAGGGGGGGTCATTCCAAGAGAAATGTTGTAAACGTTCCTTGTAAACATAGCTTTCAATAACTTAAATCCAAAAAATAAAAAAAACATGTTTTCATTTTTTTAACAAATTTTGAAAGTGTATCCGAACTTATTGAACACCGTG

>Akw_MJ1_Clone3

CACGGTGTTCAATAAGTTCGAATACAAGTTTTCATCATTGCGTAGGTATGCGCCATGTACTTATTCTGCATTGGTATTGGTGTCAGCTTTAGCTTGATTCATACGCTACCGAATGTGCCCAGTGTTGACAGTCTGTTAATTGTTGTTCATTTGTTACGCGCGATGAAAGAGTATCGGGACATCGTAATTAAGCGTTTTTTGAACGGTGAGCGACCCGGCGATATATTCCGGCTGCTGAAATCGCATGGGGTCAAGCGGAACTTTGTCTACACGACCATCAGGCGATACCGGGAGACGTCCTCGACCAATGACCGTGCGAGATCCGGTCGGCCGCGTTCAGCGAGGACGCCACGGGTCATCAAGATCGTGAGGGAGCGAATTCGGCGCAAAAAGAACCGCTCAATCCGGAAAACGGCTGCAGATCTCAACGTTTCCATTGGAACCGCTCACACCATACTCACCAAGGACCTTGGTTTCAGGCCTTACAAAAAACGTAAGGTCCATGGCGTTTCGGAGGCTACCAGCAAAAAGCGGTTGGATCGAGCTAAGAGGATCCTCTCTCGGCACGCTGGTCAGGAGTTTGTTTTTTCGGACGAGAAACTGTTCGTGCTGCAGCAGCCGCACAATGTGCAAAATGACCGGGTGTGGGCGCCATCGAGGGACAGCATTCCTGAATCCAATATAAACATCCCTCGGTTCCAAAGTGCCGCGTCGGTGATGGTTTGGGGGGCAGTATGCAAACGTGGTAAGCTACCCTTGGTGTTTATTGAAAAAAACGTCAAAATCAACGCGGCGTACTACAAAACTGAGGTTTTGGAAAAGGTTGTTGCCCCCAGTCTCCGAAGCCTCTACGGCGATGAGCACTACGTGTTCCAGCAGCACGGTGCACCAGCCCATACGGCAAATGTGGTTCAAGCCTGGTGTCGGGACAATTTAACCGACTTTCTGGACAAAACTTTGTGGCCTCCCAGCTCCCCGGACTTGAATCCTCTCGACTTTTTTGTTTGGTCCTATATGATGGCGAAGCTGAACGAATACAAGGTCAGCACTTTGGATCATTTCAAGACGGTAATTCTCAAAATCTGGGACGAAATGCCCATGCAGTCCGTGCGTGCCGCCTGCGACGCGTTCGAGAAACGTTTGAAGCTCGTTAAGGAGTACAAAGGGGGGGTCATTCCAAGAGAAATGTTGTAAACGTTCCTTGTAAACATAGCTTTCAATAACTTAAATCCAAAAAATAAAAAAAACATGTTTTCATTTTTTTAACAAATTTTGAAAGTGTATCCGAACTTATTGAACACCGTG

>Aju_MJ1_Clone1

CACGGTGTTCAATAAGTTCGAATACAAGTTTTCATCATTGCGTAGGTATGCGCCATGTACTTATTCTGCATTGGTATTGGTGTCAGCTTTAGCTTGATTCATACGCTACCGAATGTGCCCAGTGTTGACAGTCTGTTAATTGTTGTTCATTTGTTACGCGCGATGAAAGAGTATCGGGACATCGTAATTAAGCGTTTTTTGAACGGTGAGCGACCCGGCGATATATTCCGGCTGCTGAAATCGCATGGGGTCAAGCGGAACTTTGTCTACACGACCATCAGGCGATACCGGGAGACGTCCTCGACCAATGACCGTGCGAGATCCGGTCGGCCGCGTTCAGCGAGGACGCCACGGGTCATCAAGATCGTGAGGGAGCGAATTCGGCGCAAAAAGAACCGCTCAATCCGGAAAACGGCTGCAGACCTCAACGTTTCCATTGGAACCGCTCACACCATACTCACCAAGGACCTTGGTTTCAGGCCTTACAAAAAACGTAAGGTCCATGGCGTTTCGGAGGCTACCAGCAAAAAGCGGTTGGATCGAGCTAAGAGGATCCTCTCTCGGCACGCTGGTCAGGAGTTTGTTTTTTCGGACGAGAAACTGTTCGTGCTGCAGCAGCCGCACAATGTGCAAAATGACCGGGTGTGGGCGCCATCGAGGGACAGCATTCCTGAATCCAATATAAACATCCCTCGGTTCCAAAGTGCCGCGTCGGTGATGGTTTGGGGGGCAGTATGCAAACGTGGTAAGCTACCCTTGGTGTTTATTGAAAAAAACGTCAAAATCAACGCGGCGTACTACAAAACTGAGGTTTTGGAAAAGGTTGTTGCCCCCAGTCTCCGAAGCCTCTACGGCGATGAGCACTACGTGTTCCAGCAGGACGGTGCACCAGCCCATACGGCAAATGTGGTTCAAGCCTGGTGTCGGGACAATTTAACCGACTTTCTGGACAAAACTTTGTGGCCTCCCAGCTCCCCGGACTTGAATCCTCTCGACTTTTTTGTTTGGTCCTATATGATGGCGAAGCTGAACGAATACAAGGTCAGCACTTTGGATCATTTCATGACGGTAATGCTCAAAATCTGGGACGAAATGCCCATGCAGTCCGTGCGTGCCGCCTGCGACGCGTTCGAGAAACGTTTGAAGCTCGTTAAGGAGTACAAAGGGGGGGTCATTCCAAGAGAAATGTTGTAAACGTTCCTTGTAAACATAGCTTTCAATAACTTAAATCCAAAAAATAAAAAAAACATGTTTTCATTTTTTTAACAAATTTTGAAAGTGTATCCGAACTTATTGAACACCGTG

>Aju_MJ1_Clone2

CACGGTGTTCAATAAGTTCGAATACAAGTTTTCATCATTGCGTAGGTATGCGCCATGTACTTATTCTGCATTGGTATTGGTGTCAGCTTTAGCTTGATTCATACGCTACCGAATGTGCCCAGTGTTGACATTCTGTTAATTGTTGTTCATTTGTTACGCGCGATGAAAGGGTATCGGGACATCGTAATTAAGCGTTTTTTGAACGGTGAGCGACCCGGCGATATATTCCGGCTGCTGAAATCGCATGGGGTCAAGCGGAACTTTGTCTACACGACCATCAGGCGATACCGGGAGACGTCCTCGACCAATGACCGTGCGAGATCCGGTCGGCCGCGTTCAGCGAGGACGCCACGGGTCATCAAGATCGTGAGGGAGCGAATTCGGCGCAAAAAGAACCGCTCAATCCGGAAAACGGCTGCAGATCTCAACGTTTCCATTGGAACCGCTCACACCATACTCACCAAGGACCTTGGTTTCAGGCCTTACAAAAAACGTAAGGTCCATGGCGTTTCGGAGGCTACCAGCAAAAAGCGGTTGGATCGAGCTAAGAGGATCCTCTCTCGGCACGCTGGTCAGGAGTTTGTTTTTTCGGACGAGAAACTGTTCGTGCTGCAGCAGCCGCACAATGTGCAAAATGCGTCGGTGATGGTTTGGGGGGCAGTATGCAAACGTGGTAAGCTACCCTTGGTGTTTATTGAAAAAAACGTCAAAATCAACGCGGCGTACTACAAAACTGAGGTTTTGGAAAAGGTTGTTGCCCCCAGTCTCCGAAGCCTCTACGGCGATGAGCACTACGTGTTCCAGCAGGACGGTGCACCAGCCCATACGGCAAATGTGGTTCAAGCCTGGTGTCGGGACAATTTAACCGACTTTCTGGACAAAACTTTGTGGCCTCCCAGCTCCCCGGACTTGAATCCTCTCGACTTTTTTGTTTGGTCCTATATGATGGCGAAGCTGAACGAATACAAGGTCAGCACTTTGGATCATTTCAAGACGGTAATTCTCAAAATCTGGGACGAAATGCCCATGCAGTCCGTGCGTGCCGCCTGCGACGCGTTCGAGAAACGTTTGAAGCTCGTTAAGGAGTACAAAGGGGGGGTCATTCCAAGAGAAATGTTGTAAACGTTCCTTGTAAACATAGCTTTCAATAACTTAAAAAGTTATTGTGTAAAAGTGTATCCGAACTTATTGAACACCGTG

>Aju_MJ1_Clone3

CACGGTGTTCAATAAGTTCGAATACAAGTTTTCATCATTGCGTAGGTATGCGCCATGTACTTATTCTGCATTGGTATTGGTGTCAGCTTTAGCTTGATTCATACGCTACCGAATGTGCCCAGTGTTGACATTCTGTTAATTGTTGTTCATTTGTTACGCGCGATGAAAGAGTATCGGGACATCGTAATTAAGCGTTTTTTGAACGGTGAGCGACCCGGCGATATATTCCGGCTGCTGAAATCGCATGGGGTCAAGCGGAACTTTGTCTACACGACCATCAGGCGATACCGGGAGACGTCCTCGACCAATGACCGTGCGAGATCCGGTCGGCCGCGTTCAGCGAGGACGCCACGGGTCATCAAGATCGTGAGGGAGCGAATTCGGCGCAAAAAGAACCGCTCAATCCGGAAAACGGCTGCAGATCTCAACGTTTCCATTGGAACCGCTCACACCATACTCACCAAGGACCTTGGTTTCAGGCCTTACAAAAAACGTAAGGTCCATGGCGTTTCGGAGGCTACCAGCAAAAAGCGGTTGGATCGAGCTAAGAGGATCCTCTCTCGGCACGCTGGTCAGGAGTTTGTTTTTTCGGACGAGAAACTGTTCGTGCTGCAGCAGCCGCACAATGTGCAAAATGACCGGGTGTGGGCGCCATCGAGGGACAGCATTCCTGAATCCAATATAAACATCCCTCGGTTCCAAAGTGCCGCGTCGGTGATGGTTTGGGGGGCAGTATGCAAACGTGGTAAGCTACCCTTGGTGTTTATTGAAAAAAACGTCAAAATCAACGCGGCGTACTACAAAACTGAGGTTTTGGAAAAGGTTGTTGCCCCCAGTCTCCGAAGCCTCTACGGCGATGAGCACTACGTGTTCCAGCAGGACGGTGCACCAGCCCATACGGCAAATGTGGTTCAAGCCTGGTGTCGGGACAATTTAACCGACTTTCTGGACAAAACTTTGTGGCCTCCCAGCTCCCCGGACTTGAATCCTCTCGACTTTTTTGTTTGGTCCTATATGATGGCGAAGCTGAACGAATACAAGGTCAGCACTTTGGATCATTTCAAGACGGTAATTCTCAAAATCTGGGACGAAATGCCCATGCAGTCCGTGCGTGCCGCCTGCGACGCGTTCGAGAAACGTTTGAAGCTCGTTAAGGAGTACAAAGGGGGGGTCATTCCAAGAGAAATGTTGTAAACGTTCCTTGTAAACATAGCTTTCAATAACTTAAATCCAAAAAATAAAAAAAACATGTTTTCATTTTTTTAACAAATTTTGAAAGTGTATCCGAACTTATTGAACACCGTG

>Aju_MJ1_Clone4

CACGGTGTTCAATAAGTTCGAATACAAGTTTTCATCATTGCGTAGGTATGCGCCATGTACTTATTCTGCATTGGTATTGGTGTCAGCTTTAGCTTGATTCATACGCTACCGAATGTGCCCAGTGTTGACATTCTGTTAATTGTTGTTCATTTGTTACGCGCGATGAAAGAGTATCGGGACATCGTAATTAAGCGTTTTTTGAACGGTGAGCGACCCGGCGATATATTCCGGCTGCTGAAATCGCATGGGGTCAAGCGGAACTTTGTCTACACGACCATCAGGCGATACCGGGAGACGTCCTCGACCAATGACCGTGCGAGATCCGGTCGGCCGCGTTCAGCGAGGACGCCACGGGTCATCAAGATCGTGAGGGAGCGAATTCGGCGCAAAAAGAACCGCTCAATCCGGAAAACGGCTGCAGATCTCAACGTTTCCATTGGAACCGCTCACACCATACTCACCAAGGACCTTGGTTTCAGGCCTTACAAAAAACGTAAGGTCCATGGCGTTTCGGAGGCTACCAGCAAAAAGCGGTTGGATCGAGCTAAGAGGATCCTCTCTCGGCACGCTGGTCAGGAGTTTGTTTTTTCGGACGAGAAACTGTTCGTGCTGCAGCAGCCGCACAATGTGCAAAATGACCGGGTGTGGGCGCCATCGAGGGACAGCATTCCTGAATCCAATATAAACATCCCTCGGTTCCAAAGTGCCGCGTCGGTGATGGTTTGGGGGGCAGTATGCAAACGTGGTAAGCTACCCTTGGTGTTTATTGAAAAAAACGTCAAAATCAACGCGGCGTACTACAAAACTGAGGTTTTGGAAAAGGTTGTTGCCCCCAGTCTCCGAAGCCTCTACGGCGATGAGCACTACGTGTTCCAGCAGGACGGTGCACCAGCCCATACGGCAAATGTGGTTCAAGCCTGGTGTCGGGACAATTTAACCGACTTTCTGGACAAAACTTTGTGGCCTCCCAGCTCCCCGGACTTGAATCCTCTCGACTTTTTTGTTTGGTCCTATATGATGGCGAAGCTGAACGAATACAAGGTCAGCACTTTGGATCATTTCAAGACGGTAATTCTCAAAATCTGGGACGAAATGCCCATGCAGTCCGTGCGTGCCGCCTGCGACGCGTTCGAGAAACGTTTGAAGCTCGTTAAGGAGTACAAAGGGGGGGTCATTCCAAGAGAAATGTTGTAAACGTTCCTTGTAAACATAGCTTTCAATAACTTAAATCCAAAAAATAAAAAAAACATGTTTTCATTTTTTTAACAAATTTTGAAAGTGTATCCGAACTTATTGAACACCGTG

>Aju_MJ1_Clone5

CACGGTGTTCAATAAGTTCGAATACAAGTTTTCATCATTGCGTAGGTATGCGCCATGTACTTATTCTGCATTGGTATTGGTGTCAGCTTTAGCTTGATTCATACGCTACCGAATGTGCCCAGTGTTGACATTCTGTTAATTGTTGTTCATTTGTTACGCGCGATGAAAGAGTATCGGGACATCGTAATTAAGCGTTTTTTGAACGGTGAGCGACCCGGCGATATATTCCGGCTGCTGAAATCGCATGGGGTCAAGCGGAACTTTGTCTACACGACCATCAGGCGATACCGGGAGACGTCCTCGACCAATGACCGTGCGAGATCCGGTCGGCCGCGTTCAGCGAGGACGCCACGGGTCATCAAGATCGTGAGGGAGCGAATTCGGCGCAAAAAGAACCGCTCAATCCGGAAAACGGCTGCAGATCTCAACGTTTCCATTGGAACCGCTCACACCATACTCACCAAGGACCTTGGTTTCAGGCCTTACAAAAAACGTAAGGTCCATGGCGTTTCGGAGGCTACCAGCAAAAAGCGGTTGGATCGAGCTAAGAGGATCCTCTCTCGGCACGCTGGTCAGGAGTTTGTTTTTTCGGACGAGAAACTGTTCGTGCTGCAGCAGCCGCACAATGTGCAAAATGACCGGGTGTGGGCGCCATCGAGGGACAGCATTCCTGAATCCAATATAAACATCCCTCGGTTCCAAAGTGCCGCGTCGGTGATGGTTTGGGGGGCAGTATGCAAACGTGGTAAGCTACCCTTGGTGTTTATTGAAAAAAACGTCAAAATCAACGCGGCGTACTACAAAACTGAGGTTTTGGAAAAGGTTGTTGCCCCCAGTCTCCGAAGCCTCTACGGCGATGAGCACTACGTGTTCCAGCAGGACGGTGCACCAGCCCATACGGCAAATGTGGTTCAAGCCTGGTGTCGGGACAATTTAACCGACTTTCTGGACAAAACTTTGTGGCCTCCCAGCTCCCCGGACTTGAATCCTCTCGACTTTTTTGTTTGGTCCTATATGATGGCGAAGCTGAACGAATACAAGGTCAGCACTTTGGATCATTTCAAGACGGTAATTCTCAAAATCTGGGACGAAATGCCCATGCAGTCCGTGCGTGCCGCCTGCGACGCGTTCGAGAAACGTTTGAAGCTCGTTAAGGAGTACAAAGGGGGGGTCATTCCAAGAGAAATGTTGTAAACGTTCCTTGTAAACATAGCTTTCAATAACTTAAATCCAAAAAATAAAAAAAACATGTTTTCATTTTTTTAACAAATTTTGAAAGTGTATCCGAACTTATTGAACACCGTG

>Aju_MJ1_Clone6

CACGGTGTTCAATAAGTTCGAATACAAGTTTTCATCATTGCGTAGGTATGCGCCATGTACTTATTCTGCATTGGTATTGGTGTCAGCTTTAGCTTGATTCATACGCTACCGAATGCGCCCAGTGTTGACATTCTGTTAATTGTTGTTCATTTGTTACGCGCGATGAAAGAGTATCGGGACATCGTAATTAAGCGTTTTTTGAACGGTGAGCGACCCGGCGATATATTCCGGCTGCTGAAATCGCATGGGGTCAAGCGGAACTTTGTCTACACGACCATCAGGCGATACCGGGAGACGTCCTCGACCAATGACCGTGCGAGATCCGGTCGGCCGCGTTCAGCGAGGACGCCACGGGTCATCAAGATCGTGAGGGAGCGAATTCGGCGCAAAAAGAACCGCTCAATCCGGAAAACGGCTGCAGATCTCAACGTTTCCATTGGAACCGCTCACACCATACTCACCAAGGACCTTGGTTTCAGGCCTTACAAAAAACGTAAGGTCCATGGCGTTTCGGAGGCTACCAGCAAAAAGCGGTTGGATCGAGCTAAGAGGATCCTCTCTCGGCACGCTGGTCAGCAGTTTGTTTTTTCGGACGAGAAACTGTTCGTGCTGCAGCAGCCGCACAATGTGCAAAATGCGTCGGTGATGGTTTGGGGGGCAGTATGCAAACGTGGTAAGCTACCCTTGGTGTTTATTGAAAAAAACGTCAAAATCAACGCGGCGTACTACAAAACTGAGGTTTTGGAAAAGGTTGTTGCCCCCAGTCTCCGAAGCCTCTACGGCGATGAGCACTACGTGTTCCAGCAGGACGGTGCACCAGCCCATACGGCAAATGTGGCTCAAGCCTGGTGTCGGGACAATTTAACCGACTTTCTGGACAAAACTTTGTGGCCTCCCAGCTCCCCGGACTTGAATCCTCTCGACTTTTTTGTTTGGTCCTATATGATGGCGACGCTGAACGAATACAAGGTCAGCACTTTGGATCATTTCAAGACGGTAATTCTCAAAATCTGGGACGAAATGCTCATGCAGTCCGTGCGTGCCGCCTGCGACGCGTTCGAGAAACGTTTGAAGCTCGTTAAGGAGTACAAGGGGGGGGTCATTCCAAGAGAAATGTTGTAAACGTTCCTTGTAAACATAGCTTTCAATAACTTAAATCCAAAAAATAAAAAAAACATGTTTTCATTTTTTTAACAAATTTTGAAAGTGTATCCGAACTTATTGAACACCGTG

>Aju_MJ1_Clone7

CACGGTGTTCAATAAGTTCGAATACAAGTTTTCATCATTGCGTAGGTATGCGCCATGTACTTATTCTGCATTGGTATTGGTGTCAGCTTTAGCTTGATTCATACGCTACCGAATGTGCCCAGTGTTGACACTCTGTTAATTGTTGTTCATTTGTTACGCGCGATGAAAGAGTATCGGGACATCGTAATTAAGCGTTTTTTGAACGGTGAGCGACCCGGCGATATATTCCGGCTGCTGAAATCGCATGGGGTCAAGCGGAACTTTGTCTACACGACCATCAGGCGATACCGGGAGACGTCCTCGACCAATGACCGTGCGAGATCCGGTCGGCCGCGTTCAGCGAGGACGCCACGGGTCATCAAGATCGTGAGGGAGCGAATTCGGCGCAAAAAGAACCGCTCAATCCGGAAAACGGCTGCAGATCTCAACGTTTCCATTGGAACCGCTCACACCATACTCACCAAGGACCTTGGTTTCAGGCCTTACAAAAAACGTAAGGTCCATGGCGTTTTGGAGGCTACCAGCAAAAAGCGGTTGGATCGAGCTAAGAGGATCCTCTCTCGGCACGCTGGTCAGGAGTTTGTTTTTTCGGACGAGAAACTGTTCGTGCTGCAGCAGCCGCACAATGTGCAAAATGACCGGGTGTGGGCGCCATCGAGGGACAGCATTCCTGAATCCAATATAAACATCCCTCGGTTCCAAAGTGCCGCGTCGGTGATGGTTTGGGGGGCAGTATGCAAACGTGGTAAGCTACCCTTGGTGTTTATTGAAAAAAACGTCAAAATCAACGCGGCGTACTACAAAACTGAGGTTTTGGAAAAGGTTGTTGCCCCCAGTCTCCGAAGCCTCTACGGCGATGAGCACTACGTGTTCCAGCAGGACGGTGCACCAGCCCATACGGCAAATGTGGTTCAAGCCTGGTGTCGGGACAATTTAACCGACTTTCTGGACAAAACTCTGTGGCCTCCCAGCTCCCCGGACTTGAATCCTCTCGACTTTTTTGTTTGGTCCTATATGATGGCGAAGCTGAACGAATACAAGGTCAGCACTTTGGATCATTTCAAGACGGTAATTCTCAAAATCTGGGACGAAATGCCCATGCAGTCCGTGCGTGCCGCCTGCGACGCGTTCGAGAAACGTTTGAAGCTCGTTAAGGAGTACAAAGGGGGGGTCATTCCAAGAGAAATGTTGTAAACGTTCCTTGTAAACATAGCTTTCAATAACTAAAATCCAAAAAATAAAAAAACCATGTTTTCATTTTTTTAACAAATTTTGAAAGTGTATCCGAACTTATTGAACACCGTG

>Aju_MJ1_Clone8

CACGGTGTTCAATAAGTTCGAATACAAGTTTTCATCATTGCGTAGGTATGCGCCATGTACTTATTCTGCATTGGTATTGGTGTCAGCTTTAGCTTGATTCATACGCTACCGAATGTGCCCAGTGTTGACATTCTGTTAATTGTTGTTCATTTGTTACGCGCGATGAAAGAGTATCGGGACATCGTAATTAAGCGTTTTTTGAACGGTGAGCGACCCGGCGATATATTCCGGCTGCTGAAATCGCATGGGGTCAAGCGGAACTTTGTCTACACGACCATCAGGCGATACCGGGAGACGTCCTCGACCAATGACCGTGCGAGATCCGGTCGGCCGCGTTCAGCGAGGACGCCACGGGTCATCAAGATCGTGAGGGAGCGAATTCGGCGCAAAAAGAACCGCTCAATCCGGAAAACGGCTGCAGATCTCAACGTTTCCATTGGAACCGCTCACACCATACTCACCAAGGACCTTGGTTTCAGGCCTTACAAAAAACGTAAGGTCCATGGCGTTTCGGAGGCTACCAGCAAAAAGCGGTTGGATCGAGCTAAGAGGATCCTCTCTCGGCACGCTGGTCAGGAGTTTGTTTTTTCGGACGAGAAACTGTTCGTGCTGCAGCAGCCGCACAATGTGCAAAATGACCGGGTGTGGGCGCCATCGAGGGACAGCATTCCTGAATCCAATATAAACATCCCTCGGTTCCAAAGTGCCGCGTCGGTGATGGTTTGGGGGGCAGTATGCAAACGTGGTAAGCTACCCTTGGTGTTTATTGAAAAAAACGTCAAAATCAACGCGGCGTACTACAAAACTGAGGTTTTGGAAAAGGTTGTTGCCCCCAGTCTCCGAAGCCTCTACGGCGATGAGCACTACGTGTTCCAGCAGGACGGTGCACCAGCCCATACGGCAAATGTGGTTCAAGCCTGGTGTCGGGACAATTTAACCGACTTTCTGGACAAAACTTTGTGGCCTCCCAGCTCCCCGGACTTGAATCCTCTCGACTTTTTTGTTTGGTCCTATATGATGGCGAAGCTGAACGAATACAAGGTCAGCACTTTGGATCATTTCAAGACGGTAATTCTCAAAATCTGGGACGAAATGCCCATGCAGTCCGTGCGTGCCGCCTGCGACGCGTTCGAGAAACGTTTGAAGCTCGTTAAGGAGTACAAAGGGGGGGTCATTCCAAGAGAAATGTTGTAAACGTTCCTTGTAAACATAGCTTTCAATAACTTAAATCCAAAAAATAAAAAAAACATGTTTTCATTTTTTTAACAAATTTTGAAAGTGTATCCGAACTTATTGAACACCGTG

>Ape_MJ1_Clone1

CACGGTGTTCAATAAGTTCGAATACAAGTTTTCATCATTGCGTAGGTATGCGCCATGTACTTATTCTGCATTGGTATTGGTGTCAGCTTTAGCTTGATTCATACGCTACCGAATGTGCCCAGTGTTGACATTCTGTTAATTGTTGTTCATTTGTTACGCGCGATGAAAGAGTATCGGGACATCGTAATTAAGCGTTTTTTGAACGGTGAGCGACCCGGCGATATATTCCGGCTGCTGAAATCGCATGGGGTCAAGCGGAACTTTGTCTACACGACCATCAGGCGATACCGGGAGACGTCCTCGACCAATGACCGTGCGAGATCCGGTCGGCCGCGTTCAGCGAGGACGCCACGGGTCATCAAGATCGTGAGGGAGCGAATTCGGCGCAAAAAGAACCGCTCAATCCGGAAAACGGCTGCAGATCTCAACGTTTCCATTGGAACCGCTCACACCATACTCATCAAGGACCTTGGTTTCAGGCCTTACAAAAAACGTAAGGTCCATGGCGTTTCGGAGGCTACCAGCAAAAAGCGGTTGGATCGAGCTAAGAGGATCCTCTCTCGGCACGCTGGTCAGGAGTTTGTTTTTTCGGACGAGAAACTGTTCGTGCTGCAGCAGCCGCACAATGTGCAAAATGACCGGGTGTGGGCGCCATCGAGGGACAGCATTCCTGAATCCAATATAAACATCCCTCGGTTCCAAAGTGCCGCGTCGGTGATGGTTTGGGGGGCAGTATGCAAACGTGGTAAGCTACCCTTGGTGTTTATTGAAAAAAACGTCAAAATCAACGCGGCGTACTACAAAACTGAGGTTTTGGAAAAGGTTGTTGCCCCCAGTCTCCGAAGCCTCTACGGCGATGAGCACTACGTGTTCCAGCAGGACGGTGCACCAGCCCATACGGCAAATGTGGTTCAAGCCTGGTGTCGGGACAATTTAACCGACTTTCTGGACAAAACTTTGTGGCCTCCCAGCTCCCCGGACTTGAATCCTCTCGACTTTTTTGTTTGGTCCTATATGATGGCGAAGCTGAACGAATACAAGGTCAGCACTTTGGATCATTTCAAGACGGTAATTCTCAAAATCTGGGACGAAATGCCCATGCAGTCCGTGCGTGCCGCCTGCGACGCGTTCGAGAAACGTTTGAAGCTCGTTAAGGAGTACAAAGGGGGGTCATTCCAAGAGAAATGTTGTAAACGTTCCTTGTAAACATAGCTTTCAATAACTTAAATCCAAAAAATAAAAAAAACATGTTTTCATTTTTTTAACAAATTTTGAAAGTGTATCCGAACTTATTGAACACCGTG

>Ape_MJ1_Clone2

CACGGTGTTCAATAAGTTCGAATACAAGTTTTCATCATTGCGTAGGTATGCGCCATGTACATATTCTGTATTGGTATTGGTGTCAGCTTTAGCTTGATTCGTACGCTACCGAATGTGCACGGTGTTGACATTCTGTTAATTGTTGTTCATTTGTTACGCGCGATGAAAGAGTATCGGGACATCGTAATTAAGCGTTTTTTGAACGGTGAGCGACCCGGCGATATATTCCGGCTGCTGAAATCGCATGGGGTCAAGCGGAACTTTGTCTACACGACCATCAGGCGATACCGGGAGACGTCCTCGACCAATGACCGTGCGAGGTCCGGTCGGCCGCGTTCAGCGAGGACGCCACGGGTCATCAAGATCGTGAGGGAGCGAATTCGGCGCAAAAAGAACCGCTCAATCCGGAAAACGGCTGCAGATCTCAACGTTTCCATTGGAACCGCTCACACCATACTCACCAAGGACCTTGGTTTCAGGCCTTACAAAAAACGTAAGGTCCATGGCGTTTCGGAGGCTACCAGCAAAAAGCGGTTGGATCGAGCTAAGAGGATCCTCTCTCGGCACGCTGGTCAGGAGTTTGTTTTTTCGAACGAGAAACTGTTCGTGCTGCAACAGCCGCACAATGTGCAAAATGACCGGGTGTGGGCGCCATCGAGGGACAGCATTCCTGAATCCAATATAAACATCCCTCGGTTCCAAAGTGCCGCGTCGGTGATGGTTTGGGGGGCAGTATGCAAACGTGGAAAGCTACCCTTGGTGTTTATTGAAAAAAACGTCAAAATCAACGCGGCGTACTACAAAACTGAGGTTTTGGAAAAGGTTGTTGCCCCCAGTCTCCGAAGCCTCTACGGCGATGAGCACTACGTGTTCCAGCAGGACGGTGCACCAGCCCATACGGCAAATGTGGTTCAAGCCTGGTGTCGGGACAATTTAACCGACTTTCTGGACAAAACTTTGTGGCCTCCCAGCTCCCCGGACTTGAATCCTCTCGACTTTTTTGTTTGGTCCTATATGATGGCGAAGCTGAACGAATACAAGATCAGCACTTTGGATCATTTCAAGACGGTAATTCTCAAAATCTGGGACGAAATGCCCATGCAGTCCGTGCGTGCCGCCTGCGACGCGTTCGAGAAACGTTTGAAGCTCGTTAAGGAGTACAAAGGGGGGGGTCATTCCAAGAGAAATGTTGTAAACGTTCCTTGTAAACATAGCTTTCAACAACATAAATCCAAAAAATAAAAAAAACATGTTTTCATTTTTTTAACAAATTTTGAAAGTGTATCCGAACTTATTGAACACCGTG

>Ape_MJ1_Clone3

CACGGTGTTCAATAAGTTCGAATACAAGTTTTCATCATTGCGTAGGTATGCGCCATGTACTTATTCTGCATTGGTATTGGTGTCAGCTTTAGCTTGATCCATACGCTACCGAATGTGCCCAGTGTTGACATTCTGTTAATTGTTGTTCATTTGTTACGCGCGATGAAAGAGTATCGGGACATCGTAATTAAGCGTTTTTTGAACGGTGAGCGACCCGGCGATATATTCCGGCTGCTGAAATCGCATGGGGTCAAGCGGAACTTTGTCTACACGACCATCAGGCGATACCGGGAGACGTCCTCGACCAATGACCGTGCGAGATCCGGTCGGCCGCGTTCAGCGAGGACGCCACGGGTCATCAAGATCGTGAGGGAGCGAATTCGGCGCAAAAAGAACCGCTCAATCCGGAAAACGGCTGCAGATCTCAACGTTTCCATTGGAACCGCTCACACCATACTCATCAAGGACCTTGGTTTCAGGCCTTACAAAAAACGTAAGGTCCATGGCGTTTCGGAGGCTACCAGCAAAAAGCGGTTGGATCGAGCTAAGAGGATCCTCTCTCGGCACGCTGGTCAGGAGTTTGTTTTTTCGGACGAGAAACTGTTCGTGCTGCAGCAGCCGCACAATGTGCAAAATGACCGGGTGTGGGCGCCATCGAGGGACAGCATTCCTGAATCCAATATAAACATCCCTCGGTTCCAAAGTGCCGCGTCGGTGATGGTTTGGGGGGCAGTATGCAAACGTGGTAAGCTACCCTTGGTGTTTATTGAAAAAAACGTCAAAATCAACGCGGCGTACTACAAAACTGAGGTTTTGGAAAAGGTTGTTGCCCCCAGTCTCCGAAGCCTCTACGGCGATGAGCACTACGTGTTCCAGCAGGACGGTGCACCAGCCCATACGGCAAATGTGGTTCAAGCCTGGTGTCGGGACAATTTAACCGACTTTCTGGACAAAACTTTGTGGCCTCCCAGCTCCCCGGACTTGAATCCTCTCGACTTTTTTGTTTGGTCCTATATGATGGCGAAGCTGAACGAATACAAGGTCAGCACTTTGGATCATTTCAAGACGGTAATTCTCAAAATCTGGGACGAAATGCCCATGCAGTCCGTGCGTGCCGCCTGCGACGCGTTTGAGAAACGTTTGAAGCTCGTTAAGGAGTACAAAGGGGGGGTCATTCCAAGAGAAATGTTGTAAACGTTCCTTGTAAACATAGCTTTCAATAACTTAAATCCAAAAAATAAAAAAAACATGTTTTCATTTTTTTAACAAATTTTGAAAGTGTATCCGAACTTATTGAACACCGTG

>Ape_MJ1_Clone4

CACGGTGTTCAATAAGTTCGAATACAAGTTTTCATCATTGCGTAGGTATGCGCCATGTACATATCCTGCATTGGTATTGGTGTCAGCTTTAGCTTCATTCATACGCTACCGAATGTGCGCGGTGTTGACATTCTGTTAGTTGTTGTTCGTTTGTTACGCGCGATGAAAGAGTATCGGGACTTCGTAATTAAGCGTTTTTTTGAACGGTGAGCGACCCGGCGATATATTCCGGCTGCTGAAATCGCATGGGGTCAAACGGAACTTTGTCTACACGACCATCAGGCGATACCGGGAGACGTCCTCGACCAATGACCGTGCGAGATCCGGTCGGCCGCGTTCAGCGAGGACGCCACGGGTCATCAAGATCGTGAGGGAGCGAATTCGGCGCAAAAAGAACCGCTCAATCCGGAAAACGGCTGCAGATTGCTGTTCTGCAGTTTCCATTGGAACCGCTCACACCATACTCACCAAGGACCTTGGTTTCAGGCCTTACAAAAAACGTAAGGTCCATGGCGTTTCGGAGGCTACCAGCAAAAAGCGGTTGGATCGAGCTAATAGGATCTCGGCACGCTGTCAGGAGTTTGATTTTTCGGACGAGAAAGCTACCCTTGGTGTTTATTGAAAAAAAAACGCCAAAATCAACGCGGCGTACTACAAAAACTGAGGTTGTTGCCCCCAGTCTCCGAAGCCTCTACGGCGATGAGCAGCACGGTGCACCAGCCCATACGGCAAATGTGGTTCAAGCCTGGTGTCGGGACAATTTAACCGACTTTCTGGACAAAACTTTGTGGCCTCCCAGCTCCCCGGACTTGAATCCTCTCGACTTTTTTGTTTGGTCCTATATGATGGCGAAGCTGAACGAATACAAGGTCAGCACTTTGGATCATTTCAAGACGGTAATTCTCAAAATCTGGGACGAAATGCCCATGCAGTCCGTGCGTGCCGCCTGCGACGCGTTCGAGAAACGTTTGAAGCTCGTTAAGGAGTACAAAGGGGGGTCATTCCAAGAGAAATGTTGTAAACGTTCCTTGTAAACATAGCTTTCAATACCATAAATCCAAAAAATAAAAAAACATGTTTTCATTTTTTTAACAAATTTTGAAAGTGTATCCGAACTTATTGAACACCGTG

>Ape_MJ1_Clone5

CACGGTGTTCAATAAGTTCGAATACAAGTTTTCATCATTGCGTAGGTATGCGCCATGTACATATCCTGCATTGGTATTGGTGTCAGCTTTAGCTTCATTCATACGCTACCGAATGTGCGCGGTGTTGACATTCTGTTAGTTGTTGTTCGTTTGTTACGCGCGATGAAAGAGTATCGGGACTTCGTAATTAAGCGTTTTTTTGAACGGTGAGCGACCCGGCGATATATTCCGGCTGCTGAAATCGCATGGGGTCAAACGGAACTTTGTCTACACGACCATCAGGCGATACCGGGAGACGTCCTCGACCAATGACCGTGCGAGATCCGGTCGGCCGCGTTCAGCGAGGACGCCACGGGTCATCAAGATCGTGAGGGAGCGAATTCGGCGCAAAAAGAACCGCTCAATCCGGAAAACGGCTGCAGATTGCTGTTCTGCAGTTTCCATTGGAACCGCTCACACCATACTCACCAAGGACCTTGGTTTCAGGCCTTACAAAAAACGTAAGGTCCATGGCGTTTCGGAGGCTACCAGCAAAAAGCGGTTGGATCGAGCTAATAGGATCTCGGCACGCTGTCAGGAGTTTGATTTTTCGGACGAGAAAGCTACCCTTGGTGTTTATTGAAGAAAAAACGCCAAAATCAACGCGGCGTACTACAAAAACTGAGGTTGTTGCCCCCAGTCTCCGAAGCCTCTACGGCGATGAGCAGCACGGTGCACCAGCCCATACGGCAAATGTGGTTCAAGCCTGGTGTCGGGACAATTTAACCGACTTTCTGGACAAAACTTTGTGGCCTCCCAGCTCCCCGGACTTGAATCCTCTCGACTTTTTTGTTTGGTCCTATATGATGGCGAAGCTGAACGAATACAAGGTCAGCACTTTGGATCATTTCAAGACGGTAATTCTCAAAATCTGGGACGAAATGCCCATGCAGTCCGTGCGTGCCGCCTGCGACGCGTTCGAGAAACGTTTGAAGCTCGTTAAGGAGTACAAAGGGGGGTCATTCCAAGAGAAATGTTGTAAACGTTCCTTGTAAACATAGCTTTCAATACCATAAATCCAAAAAATAAAAAAACATGTTTTCATTTTTTTAACAAATTTTGAAAGTGTATCCGAACTTATTGAACACCGTG

>Ape_MJ1_Clone6

CACGGTGTTCAATAAGTTCGAATACAAGTTTTCATCATTGCGTAGGTATGCGCCATGTACATATTCTGTATTGGGATTGGTGTCAGCTTTGGCTTGATTCGTACGCTACCGAATGTGCACGGTGTTGACATTCTGTTAATTGTTGTTCATTTGTTACGCGCGATGAAAGAGTATCGGGACATCGTAATTAAGCGTTTTTTGAACGGTGAGCGACCCGGCGATATATTCCGGCTGCTGAAATCGCATGGGGTCAAGCGGAACTTTGTCTACACGACCATCAGGCGATACCGGGAGACGTCCTCGACCAATGACCGTGCGAGATCCGGTCGGCCGCGTTCAGCCAGGACGCCACGGGTCATCAAGATCGTGAGGGAGCGAATTCGGCGCAAAAAGAACCGCTCAATCCGGAAAACGGCTGCAGATCTCAACGTTTCCATTGGAACCGCTCACACCATACTCACCAAGGACCTTGGTTTCAGGCCTTACAAAAAACGTAAGGTCCATGGCGTTTCGGAGGCTACCAGCAAAAAGCGGTTGGATCGAGCTAAGAGGATCCTCTCTCGGCACGCTGGTCAGGAGTTTGTTTTTTCGGACGAGAAACTGTTCGTGCTGCAGCAGCCGCACAATGTGCAAAATGACCGGGTGTGGGCGCCATCGAGGGACAGCATTCCTGAATCCAATATAAACATCCCTCGGTTCCAAAGTGCCGCGTCGGTGATGGTTTGGGGGGCAGTATGCAAACGTGGAAAGCTACCCTTGGTGTTTATTGAAAAAAACGTCAAAATCAACGCGGCGTACTACAAAACTGAGGTTTTGGAAAAGGTTGTTGCCCCCAGTCTCCGAAGCCTCTACGGCGATGAGCACTACGTGTTCCAGCAGGACGGTGCACCAGCCCATACGGCAAATGTGGTTCAAGCCTGGTGTCGGGACAATTTAACCGACTTTCTGGACAAAACTTTGTGGCCTCCCAGCTCCCCGGACTTGAATCCTCTCGACTTTTTTGTTTGGTCCTATATGATGGCGAAGCTGAACGAATACAAGGTCAGCACTTTGGATCATTTCAAGACGGTAATTCTTAAAATCTGGGACGACATGCCCATGCAGTCCGTGCGTGCCGCCTGCGACGCGTTCGAGAAACGTTTGAAGCTCGTTAAGGAGTACAAAGGGGGGGTCATTCCAAGAGAAATGTTGTAAACGTTCCTTATAAACATAGCTTTCAACAACATAAATCCAAAAAATAAAAAAAACATGTTTTCATTTTTTTAACAAATTTTGAAAGTGTATCCGAACTTATTGAACACCGTG

>Ape_MJ1_Clone7

CACGGTGTTCAATAAGTTCGAATACAAGTTTTCATCATTGCGTAGGTATGCGCCATGTACATATTCTGTATTGGGATTGGTGTCAGCTTTGGCTTGATTCGTACGCTACCGAATGTGCACGGTGTTGACATTCTGTTAATTGTTGTTCATTTGTTACGCGCGATGAAAGAGTATCGGGACATCGTAATTAAGCGTTTTTTGAACGGTGAGCGACCCGGCGATATATTCCGGCTGCTGAAATCGCATGGGGTCAAGCGGAACTTTGTCTACACGACCATCAGGCGATACCGGGAGACGTCCTCGACCAATGACCGTGCGAGATCCGGTCGGCCGCGTTCAGCGAGGACGCCACGGGTCATCAAGATCGTGAGGGAGCGAATTCGGCGCAAAAAGAACCGCTCAATCCGGAAAACGGCTGCAGATCTCAACGTTTCCATTGGAACCGCTCACACCATACTCACCAAGGGCCTTGGTTTCAGGCCTTACAAAAAACGTAAGGTCCATGGCGTTTCGGAGGCTACCAGCAAAAAGCGGTTGGATCGAGCTAAGAGGATCCTCTCTCGGCACGCTGGTCAGGAGTTTGTTTTTTCGGACGAGAAACTGTTCGTGCTGCAGCAGCCGCACAATGTGCAAAATGACCGGGTGTGGGCGCCATCGAGGGACAGCATTCCTGAATCCAATATAAACATCCCTCGGTTCCAAAGTGCCGCGTCGGTGATGGTTTGGGGGGCAGTATGCAAACGTGGAAAGCTACCCTTGGTGTTTATTGAGAAAAACGTCAAAATCAACGCGGCGTACTACAAAACTGAGGTTTTGGAAAAGGTTGTTGCCCCCAGTCTCCGAAGCCTCTACGGCGATGAGCACTACGTGTTCCAGCAGGACGGTGCACCAGCCCATACGGCAAATGTGGTTCAAGCCTGGTGTCGGGACAATTTAACCGACTTTCTGGACAAAACTTTGTGGCCTCCCAGCTCCCCGGACTTGAATCCTCTCGACTTTTTTGTTTGGTCCTATATGATGGCGAAGCTGAACGAATACAAGGTCAGCACTTTGGATCATTTCAAGACGGTAATTCTTAAAATCTGGGACGACATGCCCATGCAGTCCGTGCGTGCCGCCTGCGACGCGTTCGAGAAACGTTTGAAGCTCGTTAAGGAGTACAAAGGGGGGGTCATTCCAAGAGAAATGTTGTAAACGTTCCTTATAAACATAGCTTTCAACAACATAAATCCAAAAAATAAAAAAAACATGTTTTCATTTTTTTAACAAATTTTGAAAGTGTATCCGAACTTATTGAACACCGTG
