# Supplementary material for: Next-Generation Sequencing Reveals Recent Horizontal Transfer of a DNA Transposon between Divergent Mosquitoes
Source: PLoS One. 2011 Feb 10;6(2):e16743. doi: 10.1371/journal.pone.0016743 (PMC3037385; doi:10.1371/journal.pone.0016743)
Supplement: File S3 — Peptide sequences of the transposase encoded by Aae_MJ1_CONCENSUS and all individual MJ1 copies that had intact open reading frames. (DOCX) [file pone.0016743.s003.docx]

**Supplemental file 3**

**Peptide sequences of the transposase encoded by Aae_MJ1_CONCENSUS and all individual *MJ1* copies that had intact open reading frames**

>Aae_MJ1_consensus

MYIFCIGIGVSFSFIHTLPNVRGVDILLVVVRLLRAMKEYRDFVIKRFLNGERPGDIFRLLKSHGVKRNFVYTTIRRYRETSSTNDRARSGRPRSARTPRVIKIVRERIRRKKNRSIRKTAADLNVSIGTAHTILIKDLGFRPYKKRKVHGVSEATSKKRLDRAKRILSRHAGQEFVFSDEKLFVLQQPHNVQNDRVWAPSRDSIPESNINIPRFQSAASVMVWGAVCKRGKLPLVFIEKNVKINAAYYKTEVLEKVVAPSLRSLYGDEHYVFQQDGAPAHTANVVQAWCRDNLTDFLDKTLWPPSSPDLNPLDFFVWSYMMAKLNEYKVSTLDHFKTVILKIWDEMPMQSVRAACDAFEKRLKLVKEYKGGVIPREML

>CONTIG_13910_402130_403457 (Aedes aegypti)

MYIFCIGIGVSFSFIHTLPNVRGVDILLVVVRLLRAMKEYRDFVIKRFLNGERPGDIFRL

LKSHGVKRNFVCTTIRRYRETSSTNDRARSGRPRSARTPRVIKIVRERIRRKKNRSIRKT

AADLNVSIGTAHTILIKDLGFRPYKKRKVHGVSEATSKKRLDRAKRILSRHAGQEFVFSD

EKLFVLQQPHNVQNDRVWAPSRDSIPESNINIPRFQSAASVMVWGAVCKRGKLPLVFIEK

NVKINAAYYKTEVLEKVVASSLRSLYGDEHYVFQQDGAPAHTANVVQAWCRDNLTDFLDK

TLWPPSSPDLNPLDFFVWSYMMAKLNEYNVSTLDHFKTVILKIWDEMPMQSVRAACDAFE

KRLKLVKEYKGGVIPREML

>Ape_MJ1_Clone3

MYLFCIGIGVSFSLIHTLPNVPSVDILLIVVHLLRAMKEYRDIVIKRFLNGERPGDIFRL

LKSHGVKRNFVYTTIRRYRETSSTNDRARSGRPRSARTPRVIKIVRERIRRKKNRSIRKT

AADLNVSIGTAHTILIKDLGFRPYKKRKVHGVSEATSKKRLDRAKRILSRHAGQEFVFSD

EKLFVLQQPHNVQNDRVWAPSRDSIPESNINIPRFQSAASVMVWGAVCKRGKLPLVFIEK

NVKINAAYYKTEVLEKVVAPSLRSLYGDEHYVFQQDGAPAHTANVVQAWCRDNLTDFLDK

TLWPPSSPDLNPLDFFVWSYMMAKLNEYKVSTLDHFKTVILKIWDEMPMQSVRAACDAFE

KRLKLVKEYKGGVIPREML

>Aju_MJ1_Clone8

MYLFCIGIGVSFSLIHTLPNVPSVDILLIVVHLLRAMKEYRDIVIKRFLNGERPGDIFRL

LKSHGVKRNFVYTTIRRYRETSSTNDRARSGRPRSARTPRVIKIVRERIRRKKNRSIRKT

AADLNVSIGTAHTILTKDLGFRPYKKRKVHGVSEATSKKRLDRAKRILSRHAGQEFVFSD

EKLFVLQQPHNVQNDRVWAPSRDSIPESNINIPRFQSAASVMVWGAVCKRGKLPLVFIEK

NVKINAAYYKTEVLEKVVAPSLRSLYGDEHYVFQQDGAPAHTANVVQAWCRDNLTDFLDK

TLWPPSSPDLNPLDFFVWSYMMAKLNEYKVSTLDHFKTVILKIWDEMPMQSVRAACDAFE

KRLKLVKEYKGGVIPREML

>Aju_MJ1_Clone5

MYLFCIGIGVSFSLIHTLPNVPSVDILLIVVHLLRAMKEYRDIVIKRFLNGERPGDIFRL

LKSHGVKRNFVYTTIRRYRETSSTNDRARSGRPRSARTPRVIKIVRERIRRKKNRSIRKT

AADLNVSIGTAHTILTKDLGFRPYKKRKVHGVSEATSKKRLDRAKRILSRHAGQEFVFSD

EKLFVLQQPHNVQNDRVWAPSRDSIPESNINIPRFQSAASVMVWGAVCKRGKLPLVFIEK

NVKINAAYYKTEVLEKVVAPSLRSLYGDEHYVFQQDGAPAHTANVVQAWCRDNLTDFLDK

TLWPPSSPDLNPLDFFVWSYMMAKLNEYKVSTLDHFKTVILKIWDEMPMQSVRAACDAFE

KRLKLVKEYKGGVIPREML

>Aju_MJ1_Clone4

MYLFCIGIGVSFSLIHTLPNVPSVDILLIVVHLLRAMKEYRDIVIKRFLNGERPGDIFRL

LKSHGVKRNFVYTTIRRYRETSSTNDRARSGRPRSARTPRVIKIVRERIRRKKNRSIRKT

AADLNVSIGTAHTILTKDLGFRPYKKRKVHGVSEATSKKRLDRAKRILSRHAGQEFVFSD

EKLFVLQQPHNVQNDRVWAPSRDSIPESNINIPRFQSAASVMVWGAVCKRGKLPLVFIEK

NVKINAAYYKTEVLEKVVAPSLRSLYGDEHYVFQQDGAPAHTANVVQAWCRDNLTDFLDK

TLWPPSSPDLNPLDFFVWSYMMAKLNEYKVSTLDHFKTVILKIWDEMPMQSVRAACDAFE

KRLKLVKEYKGGVIPREML

>Aju_MJ1_Clone3

MYLFCIGIGVSFSLIHTLPNVPSVDILLIVVHLLRAMKEYRDIVIKRFLNGERPGDIFRL

LKSHGVKRNFVYTTIRRYRETSSTNDRARSGRPRSARTPRVIKIVRERIRRKKNRSIRKT

AADLNVSIGTAHTILTKDLGFRPYKKRKVHGVSEATSKKRLDRAKRILSRHAGQEFVFSD

EKLFVLQQPHNVQNDRVWAPSRDSIPESNINIPRFQSAASVMVWGAVCKRGKLPLVFIEK

NVKINAAYYKTEVLEKVVAPSLRSLYGDEHYVFQQDGAPAHTANVVQAWCRDNLTDFLDK

TLWPPSSPDLNPLDFFVWSYMMAKLNEYKVSTLDHFKTVILKIWDEMPMQSVRAACDAFE

KRLKLVKEYKGGVIPREML

>Ale_MJ1_Clone12

MYLFCIGIGVSFSLIHTLPNVPSVDILLIVVHLLRAMKEYRDIVIKRFLNGERPGDIFRL

LKSHGVKRNFVYTTIRRYRETSSTNDRARSGRPRSARTPRVIKIVRERIRRKKNRSIRKT

AADLNVSIGTAHTILTKDLGFRPYKKRKVHGVSEATSKKRLDRAKRILSRHAGQEFVFSD

EKLFVLQQPHNVQNDRVWAPSRDSIPESNINIPRFQSAASVMVWGAVCKRGKLPLVFIEK

NVKINAAYYKTEVLEKVVAPSLRSLYGDEHYVFQQDGAPAHTANVVQAWCRDNLTDFLDK

TLWPPSSPDLNPLDFFVWSYMMAKLNEYKVSTLDHFKTVILKIWDEMPMQSVRAACDAFE

KRLKLVKEYKGGVIPREML

>Ale_MJ1_Clone9

MYLFCIGIGVSFSLIHTLPNVPSVDILLIVVHLLRAMKEYRDIVIKRFLNGERPGDIFRL

LKSHGVKRNFVYTTIRRYRETSSTNDRARSGRPRSARTPRVIKIVRERIRRKKNRSIRKT

AADLNVSIGTAHTILTKDLGFRPYKKRKVHGVSEATSKKRLDRAKRILSRHAGQEFVFSD

EKLFVLQQPHNVQNDRVWAPSRDSIPESNINIPRFQSAASVMVWGAVCKRGKLPLVFIEK

NVKINAAYYKTEVLEKVVAPSLRSLYGDEHYVFQQDGAPAHTANVVQAWCRDNLTDFLDK

TLWPPSSPDLNPLDFFVWSYMMAKLNEYKVSTLDHFKTVILKIWDEMPMQSVRAACDAFE

KRLKLVKEYKGGVIPREML

>Ale_MJ1_Clone3

MYLFCIGIGVSFSLIHTLPNVPSVDILLIVVHLLRAMKEYRDIVIKRFLNGERPGDIFRL

LKSHGVKRNFVYTTIRRYRETSSTNDRARSGRPRSARTPRVIKIVRERIRRKKNRSIRKT

AADLNVSIGTAHTILTKDLGFRPYKKRKVHGVSEATSKKRLDRAKRILSRHAGQEFVFSD

EKLFVLQQPHNVQNDRVWAPSRDSIPESNINIPRFQSAASVMVWGAVCKRGKLPLVFIEK

NVKINAAYYKTEVLEKVVAPSLRSLYGDEHYVFQQDGAPAHTANVVQAWCRDNLTDFLDK

TLWPPSSPDLNPLDFFVWSYMMAKLNEYKVSTLDHFKTVILKIWDEMPMQSVRAACDAFE

KRLKLVKEYKGGVIPREML

>Ale_MJ1_Clone2

MYLFCIGIGVSFSLIHTLPNVPSVDIMLIVVHLLRAMKEYRDIVIKRFLNGERPGDIFRL

LKSHGVKRNFVYTTIRRYRETSSTNDRARSGRPRSARTPRVIKIVRERIRRKKNRSIRKT

AADLNVSIGTAHTILTKDLGFRPYKKRKVHGVSEATSKKRLDRAKRILSRHAGQEFVFSD

EKLFVLQQPHNVQNDRVWAPSRDSIPESNINIPRFQSAASVMVWGAVCKRGKLPLVFIEK

NVKINAAYYKTEVLEKVVAPSLRSLYGDEHYVFQQDGAPAHTANVVQAWCRDNLTDFLDK

TLWPPSSPDLNPLDFFVWSYMMAKLNEYKVSTLDHFKTVILKIWDEMPMQSVRAACDAFE

KRLKLVKEYKGGVIPREML

>Aya_MJ1_Clone2

MYLFCIGIGVSFSLIHTLPNVPSVDILLIVVHLLRAMKEYRDIVIKRFLNGERPGDIFRL

LKSHGVKRNFVYTTIRRYRETSSTNDRARSGRPRSARTPRVIKIVRERIRRKKNRSIRKT

AADLNVSIGTAHTILTKDLGFRPYKKRKVHGVSETTSKKRLDRAKRILSRHAGQEFVFSD

EKLFVLQQPHNVQNDRVWAPSRDSIPESNINIPRFQSAASVMVWGAVCKRGKLPLVFIEK

NVKINAAYYKTEVLEKVVAPSLRSLYGDEHYVFQQDGAPAHTANVVQAWCRDNLTDFLDK

TLWPPSSPDLNPLDFFVWSYMMAKLNEYKVSTLDHFKTVILKIWDEMPMQSVRAACDAFE

KRLKLVKEYKGGVIPREML

>Ale_MJ1_Clone6

MYLFCIGIGVSFSLIHTLPNVPSVDILLIVVHLLRAMKEYRDIVIKRFLNGERPGDIFRL

LKSHGVKRNFVYTTIRRYRETSSTNDRARSGRPRSARTPRVIKIVRERIRRKKNRSIRKT

AADLNVSIGTAHTILTKDLGFRPYKKRKVHGVSEATSKKRLDRAKRILSRHAGQEFVFSD

EKLFVLQQPHNVQNDRVWAPSRDSIPESNINIPRFQSAASVMVWGAVCKRGKLPLVFIEK

NVKINAAYYKTEVLEKVVAPSLRSLYGDEHYVFQQDGAPAHTANVVQAWCRDNLTDFLDK

TLWPPSSPDLNPLDFFVWSYMIAKLNEYKVSTLDHFKTVILKIWDEMPMQSVRAACDAFE

KRLKLVKEYKGGVIPREML

>Ale_MJ1_Clone13

MYLFCIGIGVSFSLIHTLPNVPSVDILLIVVHLLRAMKEYRDIVIKRFLNGERPGDIFRL

LKSHGVKRNFVYTTIRRYRETSSTNDRARSGRPRSARTPRVIKIVRERIRRKKNRSIRKT

AADLNVSIGTAHTILTKDLGFRPYKKRKVHGVSEATSKKRLDRAKRILSRHAGQEFVFSD

EKLFVLQQPHNVQNDRVWAPSRDSIPESNINIPRFQSAASVMVWGAVCKRGKLPLVFIEK

NVKINAAYYKTEVLEKVVAPSLRSLYGDEHYVFQQDGAPAHMANVVQAWCRDNLTDFLDK

TLWPPSSPDLNPLDFFVWSYMMAKLNEYKVSTLDHFKTVILKIWDEMPMQSVRAACDAFE

KRLKLVKEYKGGVIPREML

>Akw_MJ1_Clone2

MYLFCIGIGVSFSLIHTLPNVPSVDSLLIVVHLLRAMKEYRDIVIKRFLNGERPGDIFRL

LKSHGVKRNFVYTTIRRYRETSSTNDRARSGRPRSARTPRVIKIVRERIRRKKNRSIRKT

AADLNVSIGTAHTILTKDLGFRPYKKRKVHGVSEATSKKRLDRAKRILSRHAGQEFVFSD

EKLFVLQQPHNVQNDRVWAPSRDSIPESNINIPRFQSAASVMVWGAVCKRGKLPLVFIEK

NVKINAAYYKTEVLEKVVAPSLRSLYGDEHYVFQQDGAPAHTANVVQAWCRDNLTDFLDK

TLWPPSSPDLNPLDFFVWSYMMAKLNEYKVSTLDHFKTVILKIWDEMPMQSVRAACDAFE

KRLKLVKEYKGGVIPREML

>Ape_MJ1_Clone6

MYIFCIGIGVSFGLIRTLPNVHGVDILLIVVHLLRAMKEYRDIVIKRFLNGERPGDIFRL

LKSHGVKRNFVYTTIRRYRETSSTNDRARSGRPRSARTPRVIKIVRERIRRKKNRSIRKT

AADLNVSIGTAHTILTKDLGFRPYKKRKVHGVSEATSKKRLDRAKRILSRHAGQEFVFSD

EKLFVLQQPHNVQNDRVWAPSRDSIPESNINIPRFQSAASVMVWGAVCKRGKLPLVFIEK

NVKINAAYYKTEVLEKVVAPSLRSLYGDEHYVFQQDGAPAHTANVVQAWCRDNLTDFLDK

TLWPPSSPDLNPLDFFVWSYMMAKLNEYKVSTLDHFKTVILKIWDDMPMQSVRAACDAFE

KRLKLVKEYKGGVIPREML

>Ahy_MJ1_Clone3

MYLFCIGIGVSFSLIHTLPNVPSVDILLIVVHLLRAMKEYRDIVIKRFLNGERPGDIFRL

LKSRGVKRNFVYTTIRRYRETSSTNDRARSGRPRSARTPRVIKIVRERIRRKKNRSIRKT

AADLNVSIGTAHTILTKDLGFRPYKKRKVHGVSEATSKKRLDRAKRILSRHAGQEFVFSD

EKLFVLQQPHNVQNDRVWAPSRDSIPESNINIPRFQSAASVMVWGAVCKRGKLPLVFIEK

NVKINAAYYKTEVLEKVVAPSLRSLYGDEHYVFQQDGAPAHTANVVQAWCRDNLTDFLDK

TLWPPSSPDLNPLDFFVWSYMMAKLNEYKVSTLDHFKTVILKIWDEMPMQSVRAACDAFE

KRLKLVKEYKGGVIPREML 1191

>Aju_MJ1_Clone7

MYLFCIGIGVSFSLIHTLPNVPSVDTLLIVVHLLRAMKEYRDIVIKRFLNGERPGDIFRL

LKSHGVKRNFVYTTIRRYRETSSTNDRARSGRPRSARTPRVIKIVRERIRRKKNRSIRKT

AADLNVSIGTAHTILTKDLGFRPYKKRKVHGVLEATSKKRLDRAKRILSRHAGQEFVFSD

EKLFVLQQPHNVQNDRVWAPSRDSIPESNINIPRFQSAASVMVWGAVCKRGKLPLVFIEK

NVKINAAYYKTEVLEKVVAPSLRSLYGDEHYVFQQDGAPAHTANVVQAWCRDNLTDFLDK

TLWPPSSPDLNPLDFFVWSYMMAKLNEYKVSTLDHFKTVILKIWDEMPMQSVRAACDAFE

KRLKLVKEYKGGVIPREML

>Asi_MJ1_Clone1

MYLFCIGIGVSFSLIHTLPNVPSVDSLLIVVHLLRAMKEYRDIVIKRFLNGERPGDIFRL

LKSHGVKRNFVYTTIRRYRETSSTNDRARSGRPRSARTPRVIKIVRERIRRKKNRSIRKT

AADLNVSIGTAHTILTKDLGFRPYKKRKVHGVSEATSKKRLDRAKRILSRHAGQEFVFSD

EKLFVLQQPHNVQNDRVWAPSRDSIPESNINIPRFQSAASVMVWGVVCKRGKLPLVFIEK

NVKINAAYYKTEVLEKVVAPSLRSLYGDEHYVFQQDGAPAHTANVVQAWCRDNLTDFLDK

TLWPPSSPDLNPLDFFVWSYMMAKLNEYKVSTLDHFKTVILKIWDEMPMQSVRAACDAFE

KRLKLVKEYKGGVIPREML

>Ale_MJ1_Clone1

MYLFCIGIGVSFSLIHTLPNVPSVDILLIVVHLLRAMKEYRDIVIKRFLNGERPGDIFRL

LKSHGVKRNFVYTTIRRYRETSSTNDRARSGRPRSARTPRIIKIVRERIRRKKNRSIRKM

AADLNVSIGTAHTILTKDLGFRPYKKRKVHGVSEATSKKRLDRAKRILSRHAGQEFVFSD

EKLFVLQQPHNVQNDRVWAPSRDSIPESNINIPRFQSAASVMVWGAVCKRGKLPLVFIEK

NVKINAAYYKTEVLEKVVASSLRSLYGDEHYVFQQDGAPAHTANVVQAWCRDNLTDFLDK

TLWPPSSPDLNPLDFFVWSYMMAKLNEYKVSTLDHFKTVILKIWDEMPMQSVRAACDAFE

KRLKLVKEYKGGVIPREML

>Akw_MJ1_Clone3

MYLFCIGIGVSFSLIHTLPNVPSVDSLLIVVHLLRAMKEYRDIVIKRFLNGERPGDIFRL

LKSHGVKRNFVYTTIRRYRETSSTNDRARSGRPRSARTPRVIKIVRERIRRKKNRSIRKT

AADLNVSIGTAHTILTKDLGFRPYKKRKVHGVSEATSKKRLDRAKRILSRHAGQEFVFSD

EKLFVLQQPHNVQNDRVWAPSRDSIPESNINIPRFQSAASVMVWGAVCKRGKLPLVFIEK

NVKINAAYYKTEVLEKVVAPSLRSLYGDEHYVFQQHGAPAHTANVVQAWCRDNLTDFLDK

TLWPPSSPDLNPLDFFVWSYMMAKLNEYKVSTLDHFKTVILKIWDEMPMQSVRAACDAFE

KRLKLVKEYKGGVIPREML

>Ape_MJ1_Clone7

MYIFCIGIGVSFGLIRTLPNVHGVDILLIVVHLLRAMKEYRDIVIKRFLNGERPGDIFRL

LKSHGVKRNFVYTTIRRYRETSSTNDRARSGRPRSARTPRVIKIVRERIRRKKNRSIRKT

AADLNVSIGTAHTILTKGLGFRPYKKRKVHGVSEATSKKRLDRAKRILSRHAGQEFVFSD

EKLFVLQQPHNVQNDRVWAPSRDSIPESNINIPRFQSAASVMVWGAVCKRGKLPLVFIEK

NVKINAAYYKTEVLEKVVAPSLRSLYGDEHYVFQQDGAPAHTANVVQAWCRDNLTDFLDK

TLWPPSSPDLNPLDFFVWSYMMAKLNEYKVSTLDHFKTVILKIWDDMPMQSVRAACDAFE

KRLKLVKEYKGGVIPREML

>Aju_MJ1_Clone1

MYLFCIGIGVSFSLIHTLPNVPSVDSLLIVVHLLRAMKEYRDIVIKRFLNGERPGDIFRL

LKSHGVKRNFVYTTIRRYRETSSTNDRARSGRPRSARTPRVIKIVRERIRRKKNRSIRKT

AADLNVSIGTAHTILTKDLGFRPYKKRKVHGVSEATSKKRLDRAKRILSRHAGQEFVFSD

EKLFVLQQPHNVQNDRVWAPSRDSIPESNINIPRFQSAASVMVWGAVCKRGKLPLVFIEK

NVKINAAYYKTEVLEKVVAPSLRSLYGDEHYVFQQDGAPAHTANVVQAWCRDNLTDFLDK

TLWPPSSPDLNPLDFFVWSYMMAKLNEYKVSTLDHFMTVMLKIWDEMPMQSVRAACDAFE

KRLKLVKEYKGGVIPREML

>Ale_MJ1_Clone11

MYLFCIGIGVSFSLIHTLPNVPSVDILLIVVHLLRAMKEYRDIVIKRFLNGERPGDIFRL

LKSHGVKRNFVYTTIRRYRETSSTNDRARSGRPRSARTPRVIKIVRERIRRKKNRSIRKT

AADLNVSIGTAHTILTKDLGFRPYKKRKVHGVSEATSKKRLDRAKRILSRHAGQEFVFSD

EKLFVLQQPHNVQNDRVWAPSRDSIPESNINIPRFQSAASVMVWGAVCKRGKLPLVFIGK

NVKINAAYYKTEVLEKVVASSLRSLYGDEHYVFQQDGAPAHTANVVQAWCRDNLTDFLDK

TLWPPSSPDLNPLDFFVWSYMMAKLNEYKVSTLDHFKTVILKIWDEMPMQSVRAACDAFE

KRLKLVKEYKGGVIPREML

>Ale_MJ1_Clone7

MYLFCIGIGVSFSLIHTLPNVPSVDILLIVVHLLRAMKEYRDIVIKRFLNGERPGDIFRL

LKSHGVKRNFVYTTIRRYRETSSTNDRARSGRPRSARTPRVIKIVRERIRRKKNRSIRKT

AADLNVSIGTAHTILTKDLGFRPYKERKVHGVSEATSKKRLDRAKRILSRHAGQEFVFSD

EKLFVLQQPHNVQNDRVWAPSRDSIPESNINIPRFQSAASVMVWGAVRKRGKLPLVFIEK

NVKINAAYYKTEVLEKVVAPSLRSLYGDEHYVFQQDGAPAHTANVVQAWCRDNLTDFLDK

TLWPPSSPDLNPLDFFVWSYMMAKLNEYKVSTLDHFKTVILKIWDEMPMQSVRAACDAFE

KRLKLVKEYKGGVIPREML

>Asi_MJ1_Clone2

MYLFCIGIGVSFSLIHTLPNVPSVDSLLIVVHLLRAMKEYRDIVIKRFLNGERPGDIFRL

LKSHGVKRNFVYTTIRRYRETSSTNDRARSGRPRSARTPRVIKIVRERIRRKKNRSIRKT

AADLNVSIGTAHTILTKDLGFRPYKKRKVHGVSEATSKKRLDRAKRILSRHAGQEFVFSD

EKLFVLQQPHNVRNDRVWAPSRDSIPESNINIPRFQSAASVMVWGVVCKRGKLPLVFIEK

NVKINAAYYKTEVLEKVVAPSLRSLYGDEHYVFQQDGAPAHTANVVQAWCRDNLTDFLDK

TLWPPSSPDLNPLDFFVWSYMMAKLNEYKVSTLDHFKTVILKIWDEMPMQSVRAACDAFE

KRLKLVKEYKGGVIPREML

>Aba_MJ1_Clone1

MYLFCIGIGVSFSLIHTLPHVPSVDSLLIVVHLLRAMKEYRDIVIKRFLNGERPGDIFRL

LKSHGVKRNFVYTTIRRYRETSSTNDRARSGRPRSARTPRVIKIVRERIRRKKNRSIRKT

AADLNVSIGTAHTILTKDLGFRPYKKRKVHGVSEATSKKRLDRAKRILSRHAGQEFVFSD

EKLFVLQQPHNVQNDRVWAPSRDSIPESNINIPRFQSAASVMVWGAVCIRGKLPLVFIEK

NVKINAAYYKTEVLEKVVAPSLRSLYGDEHYVFQQDGAPAHTANVVQAWCRDNLTDFLDK

TLWPPSSPDLNPLDFFVWSYMMAKLNEYKVSTLDHFKTVILKIWDEMPMQSVRAACDAFE

KRLKLVKEYKGGVIPREML

>Aya_MJ1_Clone1

MYLFCIGIGVSFSLIHTLPNVPSVDSLLIVVHLLRAMKEYRDIVIKRFLNGERPGDIFRL

PKSHGVKRNFVYTTIRRYRETSSTNDRARSGRPRSARTPRVIKIVRERIRRKKNRSIRKT

AADLNVSIGTAHTILTKDLGFRPYKKRKVHGVSEATSKKRLDRAKRILSRHAGQEFVFSD

EKLFVLQQPHNVQNDRVWAPSRDSIPESNINIPRFQSAASVMVWGAVCKRGKLPLVFIEK

NVKINAAYYKTEVLEKVVAPSLRSLYGDEHYVFQQDGAPAHTANVVQAWCRDNLTDFLDK

TLWPPSSPDLNPLDFFVWSYMMAKLNEYKVSTLDHFKTVILKIWDEMPMRSVRAACDAFE

KRLKLVKEYKGGVIPREML

>Akl_MJ1_Clone2

MYLFCIGIGVSFSLIHTLPNVPSVDSLLIVVHLLRAMKEYRDIVIKRFLNGERPGDIFRL

LKSHGVKRNFVYTTIRRYRETSSTNDRARSGRPRSARTPRVIKIVRERIRRKKNRSIRKT

AADLNVSIGTAHTILTKDLGFRPYKKRKVHGVSEATSKKRLDRAKRILSRHAGQEFVFSD

EKLFVLQQPHNVQN-RVWAPSRDSIPESNINIPRFQSAASVMVWGAVCKRGKLPLVFIEK

NVKINAAYYKTEALEKVVAPSLRSLYGDEHYVFQQDGAPAHTANVVQAWCRDNLTDFLDK

TLWPPSSPDLNPLDFFVWSYMMAKLNEYKVSTLDHFKTVILKIWDEMPMQSVRAACDAFE

KRLKLVKEYKGGVIPREML 1188

>Akw_MJ1_Clone1

MYLFCIGIGVSFSLIHTLPNVPSVDSLLIVVHLLRAMKEYRDIVIKRFLNGERPGDIFRL

LKSHGVKRNFVYTTIRRYRETSSTNDRARSGRPRSARTPRVIKIVRERIRRKKNRSIRKT

AADLNVSIGTAHTILTKDLGFKPYKKRKVHGVSEATSKKRLDRAKRILSRHAGQEFVFSD

EKLFVLQQPHNVQNDRMWAPSRDSIPESNINIPRFQSAASVMVWGAVCKRGKLPLVFIEK

NVKINAAYYKTEVLEKVVAPSLRSLYGDEHYVFQQDGAPAHTANVVQAWCRDNLTDFLDK

TLGPPSFPDLNPLDFFVWSYMMAKLNEYKVSTLDHFKTVILKIWDEMPMQSVRAACDAFE

KRLKLVKEYKGGVIPREML

>Asi_MJ1_Clone9

MYLFCIGIGVSFSLIHTLPNVPSVDSLLIVVHLLRAMKEYRDIVIKRFLNGERPGDIFRL

LKSHGVKRNFVYTTIRRYRETPSTNDRARSGRPRSARTPRVIKIVRERIRRKKNRSIRKT

AADLNVSIGTAHTILTKDLGFRPYKKRKVHGVSEATSKKRLDRAKRILSRHAGQEFVFSD

EKLFVLQQPHNVQNDRVWAPSRDSIPESNINIPRFQSAASVMVWGAVCKRGKLPLVFIEK

NVKINAAYYKTEVLEKVVAPSLRSLYGDEHYVFQQDGAPAHTAIVVRAWCRDNLTDFLDK

TLWPPSSPDLNPLDFFVWSYMMAKLNEYKVSTLDHFKPVILKIWDEMPMQSVRAACDAFE

KRLKLVKEYKGGVIPREML

>Ale_MJ1_Clone5

MYLFCIGIGVSFSLIHTLPNVPSVDILLIVVHLLRAMKEYRDIVIKRFLNGERPGDIFRL

LKSHGVKRNFVYTTIRRYRETSSTNDRARSGRPRSARTPRVIKIVRERIRRKKNRSIRKT

AADLNVSIGTAHTILTKDLGFRPYKKRKVHGVSEATSKKRLDRAKRILSRHAGQEFVFSD

EKLFVLQQPHNVQNDRVWAPSRDSIPESNINIPRFQSAASVMVWGAVCKRGKLPLVFIEK

NVNINAAYYKTEVLEKVVAPSLRSLYGDEHYVFQQDGAPAHTANVVQAWCRDNLTDFLDK

TLWPPSSPDLNPLDFFVWSYMMAKLNEYKVSTLDHFKTVILKIWDEMPMQSVRAACDAFE

KRLKLVKEYKGGGGGGVIPREML
