# Supplementary material for: Next-Generation Sequencing Reveals Recent Horizontal Transfer of a DNA Transposon between Divergent Mosquitoes
Source: PLoS One. 2011 Feb 10;6(2):e16743. doi: 10.1371/journal.pone.0016743 (PMC3037385; doi:10.1371/journal.pone.0016743)
Supplement: File S4 — Alignment of 64 MJ1 sequences and the parameters/model used for phylogenetic analysis. (DOCX) [file pone.0016743.s004.docx]

**Supplemental file 4**

**Alignment of 64 *MJ1* sequences and the parameters/model used for phylogenetic analysis**

Note: The parameters and the selected model are shown after the sequence alignment block. Below is an executable nexus file.

#NEXUS

begin mrbayes;

set autoclose=yes nowarn=yes;

BEGIN DATA;

dimensions ntax=64 nchar=2006;

format missing=?

interleave datatype=DNA gap= -;

matrix

CONTIG_10225 CACGGTGTTCAATAAGTTCGAATACAAGTTTTCATCATTGCGTAGGTATG

CONTIG_29853 CACGGTGTTCAATAAGTTCGAATACAAGTTTTCATCATTGGGTAGGTATG

CONTIG_11920 CACGGTGTTCAATAAGTTCGAATACAAGTTTTCATCATTGCGTAGGTATG

CONTIG_8991 CACGGTGTTCAATAAGTTCGAATACAAGTTTTCATCATTGCGTAGGTATG

CONTIG_23766 CACGGTGTTCAATAAGTTCGAATACAAGTTTTCATCATTGCGTAGGTATG

CONTIG_13910 CACGGTGTTCAATAAGTTCGAATACAAGTTTTCATCATTGCGTAGGTATG

CONTIG_4960 CACGGTGTTCAATAAGTTCGAATACAAGTTTTCATCATTGCGTAGGTATG

Ape_MJ1_Clone6 CACGGTGTTCAATAAGTTCGAATACAAGTTTTCATCATTGCGTAGGTATG

Ape_MJ1_Clone7 CACGGTGTTCAATAAGTTCGAATACAAGTTTTCATCATTGCGTAGGTATG

Ape_MJ1_Clone2 CACGGTGTTCAATAAGTTCGAATACAAGTTTTCATCATTGCGTAGGTATG

Ale_MJ1_Clone1 CACGGTGTTCAATAAGTTCGAATACAAGTTTTCATCATTGCGTAGGTATG

Ale_MJ1_Clone11 CACGGTGTTCAATAAGTTCGAATACAAGTTTTCATCATTGCGTAGGTATG

Ahy_MJ1_Clone1 CACGGTGTTCAATAAGTTCGAATACAAGTTTTCATCATTGCGTAGGTATG

Ahy_MJ1_Clone2 CACGGTGTTCAATAAGTTCGAATACAAGTTTTCATCATTGCGTAGGTATG

Ale_MJ1_Clone8 CACGGTGTTCAATAAGTTCGAATACAAGTTTTCATCATTGCGTAGGTATG

Acr_MJ1_Clone2 CACGGTGTTCAATAAGTTCGAATACAAGTTTTCATCATTGCGTAGGTATG

Acr_MJ1_Clone3 CACGGTGTTCAATAAGTTCGAATACAAGTTTTCATCATTGCGTAGGTATG

Acr_MJ1_Clone1 CACGGTGTTCAATAAGTTCGAATACAAGTTTTCATCATTGCGTAGGTATG

Ape_MJ1_Clone4 CACGGTGTTCAATAAGTTCGAATACAAGTTTTCATCATTGCGTAGGTATG

Ape_MJ1_Clone5 CACGGTGTTCAATAAGTTCGAATACAAGTTTTCATCATTGCGTAGGTATG

Ahy_MJ1_Clone3 CACGGTGTTCAATAAGTTCGAATACAAGTTTTCATCATTGCGTAGGTATG

Ale_MJ1_Clone7 CACGGTGTTCAATAAGTTCGAATACAAGTTTTCATCATTGCGTAGGTATG

Aju_MJ1_Clone3 CACGGTGTTCAATAAGTTCGAATACAAGTTTTCATCATTGCGTAGGTATG

Aya_MJ1_Clone2 CACGGTGTTCAATAAGTTCGAATACAAGTTTTCATCATTGCGTAGGTATG

Asi_MJ1_Clone2 CACGGTGTTCAATAAGTTCGAATACAAGTTTTCATCATTGCGTAGGTATG

Asi_MJ1_Clone7 CACGGTGTTCAATAAGTTCGAATACAAGTTTTCATCATTGCGTAGGTATG

Asi_MJ1_Clone1 CACGGTGTTCAATAAGTTCGAATACAAGTTTTCATCATTGCGTAGGTATG

Asi_MJ1_Clone3 CACGGTGTTCAATAAGTTCGAATACAAGTTTTCATCATTGCGTAGGTATG

Asi_MJ1_Clone4 CACGGTGTTCAATAAGTTCGAATACAAGTTTTCATCATTGCGTAGGTATG

Asi_MJ1_Clone6 CACGGTGTTCAATAAGTTCGAATACAAGTTTTCATCATTGCGTAGGTATG

Akl_MJ1_Clone3 CACGGTGTTCAATAAGTTCGAATACAAGTTTTCATCATTGCGTAGGTATG

Aba_MJ1_Clone2 CACGGTGTTCAATAAGTTCGAATACAAGTTTTCATCATTGCGTAGGTATG

Akw_MJ1_Clone3 CACGGTGTTCAATAAGTTCGAATACAAGTTTTCATCATTGCGTAGGTATG

Aya_MJ1_Clone3 CACGGTGTTCAATAAGTTCGAATACAAGTTTTCATCATTGCGTAGGTATG

Aju_MJ1_Clone1 CACGGTGTTCAATAAGTTCGAATACAAGTTTTCATCATTGCGTAGGTATG

Asi_MJ1_Clone5 CACGGTGTTCAATAAGTTCGAATACAAGTTTTCATCATTGCGTAGGTATG

Asi_MJ1_Clone9 CACGGTGTTCAATAAGTTCGAATACAAGTTTTCATCATTGCGTAGGTATG

Akw_MJ1_Clone2 CACGGTGTTCAATAAGTTCCAATACAAGTTTTCATCATTGCGTAGGTATG

Asi_MJ1_Clone8 CACGGTGTTCAATAAGTTCGAATACAAGTTTTCATCATTGCGTAGGTATG

Akl_MJ1_Clone2 CACGGTGTTCAATAAGTTCGAATACAAGTTTTCATCATTGCGTAGGTATG

Aya_MJ1_Clone1 CACGGTGTTCAATAAGTTCGAATACAAGTTTTCATCATTGCGTAGGTATG

Akl_MJ1_Clone1 CACGGTGTTCAATAAGTTCGAATACAAGTTTTTATCATTGCGTAGGTATG

Aba_MJ1_Clone3 CACGGTGTTCAATAAGTTCGAATACAAGTTTTTATCATTGCGTAGGTATG

Aba_MJ1_Clone1 CACGGTGTTCAATAAGTTCGAATACAAGTTTTCATCATTGCGTAGGTATG

Akw_MJ1_Clone1 CACGGTGTTCAATAAGTTCGAATACAAGTTTTCATCATTGCGTAGGTATG

Aju_MJ1_Clone2 CACGGTGTTCAATAAGTTCGAATACAAGTTTTCATCATTGCGTAGGTATG

Aju_MJ1_Clone7 CACGGTGTTCAATAAGTTCGAATACAAGTTTTCATCATTGCGTAGGTATG

Ale_MJ1_Clone4 CACGGTGTTCAATAAGTTCGAATACAAGTTTTCATCATTGCGTAGGTATG

Ale_MJ1_Clone10 CACGGTGTTCAATAAGTTCGAATACAAGTTTTCATCATTGCGTAGGTATG

Aju_MJ1_Clone5 CACGGTGTTCAATAAGTTCGAATACAAGTTTTCATCATTGCGTAGGTATG

Ale_MJ1_Clone5 CACGGTGTTCAATAAGTTCGAATACAAGTTTTCATCATTGCGTAGGTATG

Aju_MJ1_Clone6 CACGGTGTTCAATAAGTTCGAATACAAGTTTTCATCATTGCGTAGGTATG

Ale_MJ1_Clone3 CACGGTGTTCAATAAGTTCGAATACAAGTTTTCATCATTGCGTAGGTATG

Ale_MJ1_Clone12 CACGGTGTTCAATAAGTTCGAATACAAGTTTTCATCATTGCGTAGGTATG

Aju_MJ1_Clone4 CACGGTGTTCAATAAGTTCGAATACAAGTTTTCATCATTGCGTAGGTATG

Aju_MJ1_Clone8 CACGGTGTTCAATAAGTTCGAATACAAGTTTTCATCATTGCGTAGGTATG

Ale_MJ1_Clone9 CACGGTGTTCAATAAGTTCGAATACAAGTTTTCATCATTGCGTAGGTATG

Ale_MJ1_Clone6 CACGGTGTTCAATAAGTTCGAATACAAGTTTTCATCATTGCGTAGGTATG

Ale_MJ1_Clone13 CACGGTGTTCAATAAGTTCGAATACAAGTTTTCATCATTGCGTAGGTATG

Ale_MJ1_Clone2 CACGGTGTTCAATAAGTTCGAATACAAGTTTTCATCATTGCGTAGGTATG

Ape_MJ1_Clone1 CACGGTGTTCAATAAGTTCGAATACAAGTTTTCATCATTGCGTAGGTATG

Ape_MJ1_Clone3 CACGGTGTTCAATAAGTTCGAATACAAGTTTTCATCATTGCGTAGGTATG

CONTIG_574 CACGGTGTTCAATAAGTTCGAATACAAGTTTTCATCATTGCGTAGGTATG

CONTIG_7401 CACGGTGTTCAATAAGTTCGAATACAAGTTTTCATCATTGCGTAGGTATG

CONTIG_10225 CGCCATGTACATATTCTGCATTGGTATTGGTGTCA-GCTTT-AGCTTCAT

CONTIG_29853 CGCCATGTACATATTCTGCATTGGTATTGGTGTCA-GCTTT-AGCTTCAT

CONTIG_11920 CGCCATGTACATATTCTGCATTGGTATTGGTGTCA-GCTTT-AGCTTCAT

CONTIG_8991 CGCCATGTACATATTCTGCATTGGTATTGGTGTCA-GCTTT-AGCTTCAT

CONTIG_23766 CGCCATGTACATATTCTGCATTGGTATTGGTGTCA-GCTTT-AGCTTCAT

CONTIG_13910 CGCCATGTACATATTCTGCATTGGTATTGGTGTCA-GCTTT-AGCTTCAT

CONTIG_4960 CGCCATGTACATATTCTGCATTGGTATTGGTGTCA-GCTTT-AGCTTCAT

Ape_MJ1_Clone6 CGCCATGTACATATTCTGTATTGGGATTGGTGTCA-GCTTT-GGCTTGAT

Ape_MJ1_Clone7 CGCCATGTACATATTCTGTATTGGGATTGGTGTCA-GCTTT-GGCTTGAT

Ape_MJ1_Clone2 CGCCATGTACATATTCTGTATTGGTATTGGTGTCA-GCTTT-AGCTTGAT

Ale_MJ1_Clone1 CGCCATGTACTTATTCTGCATTGGTATTGGTGTCA-GCTTT-AGCTTGAT

Ale_MJ1_Clone11 CGCCATGTACTTATTCTGCATTGGTATTGGTGTCA-GCTTT-AGCTTGAT

Ahy_MJ1_Clone1 CGCCATGTACTTATTCTGCATTGGTATTGGTGTCA-GCTTT-AGCTTGAT

Ahy_MJ1_Clone2 CGCCATGTACTTATTCTGCATTGGTATTGGTGTCA-GCTTT-AGCTTGAT

Ale_MJ1_Clone8 CGCCATGTACTTATTCTGCATTGGTATTGGTGTCA-GCTTT-AGCTTGAT

Acr_MJ1_Clone2 CGCCATGTACATATTCTGCATTGTTATTGGTGTCA-ACTTTTAGCTTCAT

Acr_MJ1_Clone3 CGCCATGTACATATTCTGCATTGTTATTGGTGTCA-ACTTTTAGCTTCAT

Acr_MJ1_Clone1 CGCCATGTACATATTCTGAATTGTTATTGGTGTCA-GCTTT-AGCTTCAT

Ape_MJ1_Clone4 CGCCATGTACATATCCTGCATTGGTATTGGTGTCA-GCTTT-AGCTTCAT

Ape_MJ1_Clone5 CGCCATGTACATATCCTGCATTGGTATTGGTGTCA-GCTTT-AGCTTCAT

Ahy_MJ1_Clone3 CGCCATGTACTTATTCTGCATTGGTATTGGTGTCA-GCTTT-AGCTTGAT

Ale_MJ1_Clone7 CGCCATGTACTTATTCTGCATTGGTATTGGTGTCA-GCTTT-AGCTTGAT

Aju_MJ1_Clone3 CGCCATGTACTTATTCTGCATTGGTATTGGTGTCA-GCTTT-AGCTTGAT

Aya_MJ1_Clone2 CGCCATGTACTTATTCTGCATTGGTATTGGTGTCA-GCTTT-AGCTTGAT

Asi_MJ1_Clone2 CGCCATGTACTTATTCTGCATTGGTATTGGTGTCA-GCTTT-AGCTTGAT

Asi_MJ1_Clone7 CGCCATGTACTTATTCTGCATTGGTATTGGTGTCA-GCTTT-AGCTTGAT

Asi_MJ1_Clone1 CGCCATGTACTTATTCTGCATTGGTATTGGTGTCA-GCTTT-AGCTTGAT

Asi_MJ1_Clone3 CGCCATGTACTTATTCTGCATTGGTATTGGTGTCA-GCTTT-AGCTTGAT

Asi_MJ1_Clone4 CGCCATGTACTTATTCTGCATTGGTATTGGTGTCA-GCTTT-AGCTTGAT

Asi_MJ1_Clone6 CGCCATGTACTTATTCTGCATTGGTATTGGTGTCA-GCTTT-AGCTTGAT

Akl_MJ1_Clone3 CGCCATGTACTTATTCTGCATTGGTATTGGTGTCA-GCTTT-AGCTTGAT

Aba_MJ1_Clone2 CGCCATGTACTTATTCTGCATTGGTATTGGTGTCA-GCTTT-AGCTTGAT

Akw_MJ1_Clone3 CGCCATGTACTTATTCTGCATTGGTATTGGTGTCA-GCTTT-AGCTTGAT

Aya_MJ1_Clone3 CGCCATGTACTAATTCTGCATTGGTATTGGTGTCA-GCTTT-AGCTTGAT

Aju_MJ1_Clone1 CGCCATGTACTTATTCTGCATTGGTATTGGTGTCA-GCTTT-AGCTTGAT

Asi_MJ1_Clone5 CGCCATGTACTTATTCTGCATTGGTATTGGTGTCA-GCTTT-ACCTTGAT

Asi_MJ1_Clone9 CGCCATGTACTTATTCTGCATTGGTATTGGTGTCA-GCTTT-AGCTTGAT

Akw_MJ1_Clone2 CGCCATGTACTTATTCTGCATTGGTATTGGTGTCA-GCTTT-AGCTTGAT

Asi_MJ1_Clone8 CGCCATGTACTTATTCTGCATTGGTATTGGTGTCACGCTTT-AGCTTGAT

Akl_MJ1_Clone2 CGCCATGTACTTATTCTGCATTGGTATTGGTGTCA-GCTTT-AGCTTGAT

Aya_MJ1_Clone1 CGCCATGTACTTATTCTGCATTGGTATTGGTGTCA-GCTTT-AGCTTGAT

Akl_MJ1_Clone1 CGCCATGTACTTATTCTGCATTGGTATTGGTGTCA-GCTTT-AGCTTGAT

Aba_MJ1_Clone3 CGCCATGTACTTATTCTGCATTGGTATTGGTGTCA-GCTTT-AGCTTGAT

Aba_MJ1_Clone1 CGCCATGTACTTATTCTGCATTGGTATTGGTGTCA-GCTTT-AGCTTGAT

Akw_MJ1_Clone1 CGCCATGTACTTATTCTGCATTGGTATCGGTGTCA-GCTTT-AGCTTGAT

Aju_MJ1_Clone2 CGCCATGTACTTATTCTGCATTGGTATTGGTGTCA-GCTTT-AGCTTGAT

Aju_MJ1_Clone7 CGCCATGTACTTATTCTGCATTGGTATTGGTGTCA-GCTTT-AGCTTGAT

Ale_MJ1_Clone4 CGCCATGTACTTATTCTGCATTGGTATTGGTGTCA-GCTTT-AGCTTGAT

Ale_MJ1_Clone10 CGCCATGTACTTATTCTGCATTGGTATTGGTGTCA-GCTTT-AGCTTGAT

Aju_MJ1_Clone5 CGCCATGTACTTATTCTGCATTGGTATTGGTGTCA-GCTTT-AGCTTGAT

Ale_MJ1_Clone5 CGCCATGTACTTATTCTGCATTGGTATTGGTGTCA-GCTTT-AGCTTGAT

Aju_MJ1_Clone6 CGCCATGTACTTATTCTGCATTGGTATTGGTGTCA-GCTTT-AGCTTGAT

Ale_MJ1_Clone3 CGCCATGTACTTATTCTGCATTGGTATTGGTGTCA-GCTTT-AGCTTGAT

Ale_MJ1_Clone12 CGCCATGTACTTATTCTGCATTGGTATTGGTGTCA-GCTTT-AGCTTGAT

Aju_MJ1_Clone4 CGCCATGTACTTATTCTGCATTGGTATTGGTGTCA-GCTTT-AGCTTGAT

Aju_MJ1_Clone8 CGCCATGTACTTATTCTGCATTGGTATTGGTGTCA-GCTTT-AGCTTGAT

Ale_MJ1_Clone9 CGCCATGTACTTATTCTGCATTGGTATTGGTGTCA-GCTTT-AGCTTGAT

Ale_MJ1_Clone6 CGCCATGTACTTATTCTGCATTGGTATTGGTGTCA-GCTTT-AGCTTGAT

Ale_MJ1_Clone13 CGCCATGTACTTATTCTGCATTGGTATTGGTGTCA-GCTTT-AGCTTGAT

Ale_MJ1_Clone2 CGCCATGTACTTATTCTGCATTGGTATTGGTGTCA-GCTTT-AGCTTGAT

Ape_MJ1_Clone1 CGCCATGTACTTATTCTGCATTGGTATTGGTGTCA-GCTTT-AGCTTGAT

Ape_MJ1_Clone3 CGCCATGTACTTATTCTGCATTGGTATTGGTGTCA-GCTTT-AGCTTGAT

CONTIG_574 CGCCATGTACATATTCTGCATTGGTATTGGTGTCA-GCTTT-AGCTTCAT

CONTIG_7401 CGCCATGTACATATTCTGCATTGGTATTGGTGTCA-GCTTT-AGCTTCAT

CONTIG_10225 TC-ATACGC-TACCGAATGTGCGCGGTGTTGACATTCTGTTAGTTGTTGT

CONTIG_29853 TC-ATACGC-TACCGAATGTGCGCGGTGTTGACATTCTGTTAGTTGTTGT

CONTIG_11920 TC-ATACGC-TACAGAATGTGCGCGGTGTTGACATTCTGTTAGTTGTTGT

CONTIG_8991 TC-ATACGC-TACCGAATGTGCGCGGTGTTGACATTCTGTTAGTTGTTGT

CONTIG_23766 TC-ATACGC-TACCGAATGTGCGCGGTGTTGACATTCTGTTAGTTGTTGT

CONTIG_13910 TC-ATACGC-TACCGAATGTGCGCGGTGTTGACATTTTGTTAGTTGTTGT

CONTIG_4960 TC-ATACGC-TACCGAATGTGCGCGGTGTTGACATTCTGTTAGTTGTTGT

Ape_MJ1_Clone6 TC-GTACGC-TACCGAATGTGCACGGTGTTGACATTCTGTTAATTGTTGT

Ape_MJ1_Clone7 TC-GTACGC-TACCGAATGTGCACGGTGTTGACATTCTGTTAATTGTTGT

Ape_MJ1_Clone2 TC-GTACGC-TACCGAATGTGCACGGTGTTGACATTCTGTTAATTGTTGT

Ale_MJ1_Clone1 TC-ATACGC-TACCGAATGTGCCCAGTGTTGACATTCTGTTAATTGTTGT

Ale_MJ1_Clone11 TC-ATACGC-TACCGAATGTGCCCAGTGTTGACATTCTGTTAATTGTTGT

Ahy_MJ1_Clone1 TC-ATACGC-TACCGAATGTGCCCAGTGTTGACATTCTGTTAATTGTTGT

Ahy_MJ1_Clone2 TC-ATACGC-TACCGAATGTGCCCAGTGTTGACATTCTGTTAATTGTTGT

Ale_MJ1_Clone8 TC-ATACGC-TACCGAATGTGCCCAGTGTTGACATTCTGTTAATTGTTGT

Acr_MJ1_Clone2 TC-ATACGC-TACCGAATGTGCACGGTGTTGACATTCTGTTAGTTGTTGT

Acr_MJ1_Clone3 TC-ATACGC-TACCGAATGTGCACGGTGTTGACATTCTGTTAGTTGTTGT

Acr_MJ1_Clone1 TC-ATACGC-TACCGAATGTGCGCGGTGTTGACATTCTGTTAGTTGTTGT

Ape_MJ1_Clone4 TC-ATACGC-TACCGAATGTGCGCGGTGTTGACATTCTGTTAGTTGTTGT

Ape_MJ1_Clone5 TC-ATACGC-TACCGAATGTGCGCGGTGTTGACATTCTGTTAGTTGTTGT

Ahy_MJ1_Clone3 TC-ATACGC-TACCGAATGTGCCCAGTGTTGACATTCTGTTAATTGTTGT

Ale_MJ1_Clone7 TC-ATACGC-TACCGAATGTGCCCAGTGTTGACATTCTGTTAATTGTTGT

Aju_MJ1_Clone3 TC-ATACGC-TACCGAATGTGCCCAGTGTTGACATTCTGTTAATTGTTGT

Aya_MJ1_Clone2 TC-ATACGC-TACCGAATGTGCCCAGTGTTGACATTCTGTTAATTGTTGT

Asi_MJ1_Clone2 TC-ATACGC-TACCGAATGTGCCCAGTGTTGACAGTCTGTTAATTGTTGT

Asi_MJ1_Clone7 TC-ATACGC-TACCGAATGTGCCCAGTGTTGACAGTCTGTTAATTGTTGT

Asi_MJ1_Clone1 TC-ATACGC-TACCGAATGTGCCCAGTGTTGACAGTCTGTTAATTGTTGT

Asi_MJ1_Clone3 TC-ATACGC-TACCGAATGTGCCCAGTGTTGACAGTCTGTTAATTGTTGT

Asi_MJ1_Clone4 TC-ATACGC-TACCGAATGTGCCCAGTGTTGACAGTCTGTTAATTGTTGT

Asi_MJ1_Clone6 TC-ATACGC-TACCGAATGTGCCCAGTGTTGACAGTCTGTTAATTGTTGT

Akl_MJ1_Clone3 TC-ATACGC-TACCGAATGTGCCCAGTGTTGACAGTCTGTTAATTGTTGT

Aba_MJ1_Clone2 TC-ATACGC-TACCGAATGTGCCCAGTGTTGACAGTCTGTTAATTGTTGT

Akw_MJ1_Clone3 TC-ATACGC-TACCGAATGTGCCCAGTGTTGACAGTCTGTTAATTGTTGT

Aya_MJ1_Clone3 TC-ATACGC-TACCGAATGTGCCCAGTGTTGACAGTCTGTTAATTGTTGT

Aju_MJ1_Clone1 TC-ATACGC-TACCGAATGTGCCCAGTGTTGACAGTCTGTTAATTGTTGT

Asi_MJ1_Clone5 TC-ATACGC-TACCGAATGTGCCCAGTGTTGACAGTCTGTTAATTGTTGT

Asi_MJ1_Clone9 TC-ATACGC-TACCGAATGTGCCCAGTGTTGACAGTCTGTTAATTGTTGT

Akw_MJ1_Clone2 TC-ATACGC-TACCGAATGTGCCCAGTGTTGACAGTCTGTTAATTGTTGT

Asi_MJ1_Clone8 TCCATACGCCTACCGAATGTGCCCAGTGTTGACAGTCTGTTAATTGTTGT

Akl_MJ1_Clone2 TC-ATACGC-TACCGAATGTGCCCAGTGTTGACAGTCTGTTAATTGTTGT

Aya_MJ1_Clone1 TC-ATACGC-TACCGAATGTGCCCAGTGTTGACAGTCTGTTAATTGTTGT

Akl_MJ1_Clone1 TC-ATACGC-TACCGAATGTGCCCAGTGTTGACAGTCTGTTAATTGTTGT

Aba_MJ1_Clone3 TC-ATACGC-TACCGAATGTGCCCAGTGTTGACAGTCTGTTAATTGTTGT

Aba_MJ1_Clone1 TC-ATACGC-TACCGCATGTGCCCAGTGTTGACAGTCTGTTAATTGTTGT

Akw_MJ1_Clone1 TC-ATACGC-TACCGAATGTGCCCAGTGTTGACAGTCTGTTAATTGTTGT

Aju_MJ1_Clone2 TC-ATACGC-TACCGAATGTGCCCAGTGTTGACATTCTGTTAATTGTTGT

Aju_MJ1_Clone7 TC-ATACGC-TACCGAATGTGCCCAGTGTTGACACTCTGTTAATTGTTGT

Ale_MJ1_Clone4 TC-ATACGC-TACCGAATGTGCCCAGTGTTGACACTCTGTTAATTGTTGT

Ale_MJ1_Clone10 TC-ATACGC-TACCGAATGTGCCCAGTGTTGACATTCTGTTAATTGTTGT

Aju_MJ1_Clone5 TC-ATACGC-TACCGAATGTGCCCAGTGTTGACATTCTGTTAATTGTTGT

Ale_MJ1_Clone5 TC-ATACGC-TACCGAATGTGCCCAGTGTTGACATTCTGTTAATTGTTGT

Aju_MJ1_Clone6 TC-ATACGC-TACCGAATGCGCCCAGTGTTGACATTCTGTTAATTGTTGT

Ale_MJ1_Clone3 TC-ATACGC-TACCGAATGTGCCCAGTGTTGACATTCTGTTAATTGTTGT

Ale_MJ1_Clone12 TC-ATACGC-TACCGAATGTGCCCAGTGTTGACATTCTGTTAATTGTTGT

Aju_MJ1_Clone4 TC-ATACGC-TACCGAATGTGCCCAGTGTTGACATTCTGTTAATTGTTGT

Aju_MJ1_Clone8 TC-ATACGC-TACCGAATGTGCCCAGTGTTGACATTCTGTTAATTGTTGT

Ale_MJ1_Clone9 TC-ATACGC-TACCGAATGTGCCCAGTGTTGACATTCTGTTAATTGTTGT

Ale_MJ1_Clone6 TC-ATACGC-TACCGAATGTGCCCAGTGTTGACATTCTGTTAATTGTTGT

Ale_MJ1_Clone13 TC-ATACGC-TACCGAATGTGCCCAGTGTTGACATTCTGTTAATTGTTGT

Ale_MJ1_Clone2 TC-ATACGC-TACCGAATGTGCCCAGTGTTGACATTATGTTAATTGTTGT

Ape_MJ1_Clone1 TC-ATACGC-TACCGAATGTGCCCAGTGTTGACATTCTGTTAATTGTTGT

Ape_MJ1_Clone3 CC-ATACGC-TACCGAATGTGCCCAGTGTTGACATTCTGTTAATTGTTGT

CONTIG_574 TC-ATACGC-TACCGAATGTGCGCGGTGTTGACATTCTGTTAGTTGTTGT

CONTIG_7401 TC-ATACGC-TACCGAATGTGCACGGTGTTGACATTCTGTTAGTTGTTGT

CONTIG_10225 TCGTTTGTTACGCGCGATGAAAGAGTATCGGGACTTCGTAAT--------

CONTIG_29853 TCGTTTGTTACGTGCGATGAAAGAGTATCGGGACTTGGTAAT--------

CONTIG_11920 TCGTTTGTTACGCGCGATGAAAGAGTATCGGGACTTCGTAAT--------

CONTIG_8991 TCGTTTGTTACGCGCGATGAAAGAGTATCGGGACTTCGTAAT--------

CONTIG_23766 TCGTTTGTTACGCGCGATGAAAGAGTATCGGGACTTCGTAAT--------

CONTIG_13910 TCGTTTGTTACGCGCGATGAAAGAGTATCGGGACTTCGTAAT--------

CONTIG_4960 TCGTTTGTTACGCGCGATGAAAGAGTATCGGGACTTCGTAAT--------

Ape_MJ1_Clone6 TCATTTGTTACGCGCGATGAAAGAGTATCGGGACATCGTAAT--------

Ape_MJ1_Clone7 TCATTTGTTACGCGCGATGAAAGAGTATCGGGACATCGTAAT--------

Ape_MJ1_Clone2 TCATTTGTTACGCGCGATGAAAGAGTATCGGGACATCGTAAT--------

Ale_MJ1_Clone1 TCATTTGTTACGCGCGATGAAAGAGTATCGGGACATCGTAAT--------

Ale_MJ1_Clone11 TCATTTGTTACGCGCGATGAAAGAGTATCGGGACATCGTAAT--------

Ahy_MJ1_Clone1 TCATTTGTTACGCGCGATGAAAGAGTATCGGGACATCGTAAT--------

Ahy_MJ1_Clone2 TCATTTGTTACGCGCGATGAAAGAGTATCGGGACATCGTAAT--------

Ale_MJ1_Clone8 TCATTTGTTACGCGCGATGAAAGAGTATCGGGACATCGTAAT--------

Acr_MJ1_Clone2 TCGTTTGTTACGCGCGATGAAAGAGTATCGGGACTTCGTAAT--------

Acr_MJ1_Clone3 TCGTTTGTTACGCGCGATGAAAGAGTATCGGGACTTCGTAAT--------

Acr_MJ1_Clone1 TCGTTTGTTACGCGCGATGAAAGAGTATCGGGACTTCGTAAT--------

Ape_MJ1_Clone4 TCGTTTGTTACGCGCGATGAAAGAGTATCGGGACTTCGTAAT--------

Ape_MJ1_Clone5 TCGTTTGTTACGCGCGATGAAAGAGTATCGGGACTTCGTAAT--------

Ahy_MJ1_Clone3 TCATTTGTTACGCGCGATGAAAGAGTATCGGGACATCGTAAT--------

Ale_MJ1_Clone7 TCATTTGTTACGCGCGATGAAAGAGTATCGGGACATCGTAAT--------

Aju_MJ1_Clone3 TCATTTGTTACGCGCGATGAAAGAGTATCGGGACATCGTAAT--------

Aya_MJ1_Clone2 TCATTTGTTACGCGCGATGAAAGAGTATCGGGACATCGTAAT--------

Asi_MJ1_Clone2 TCATTTGTTACGCGCGATGAAAGAGTATCGGGACATCGTAAT--------

Asi_MJ1_Clone7 TCATTTGTTACGCGCGATGAAAGAGTATCGGGACATCGTAAT--------

Asi_MJ1_Clone1 TCATTTGTTACGCGCGATGAAAGAGTATCGGGACATCGTAAT--------

Asi_MJ1_Clone3 TCATTTGTTACGCGCGATGAAAGAGTATCGGGACATCGTAAT--------

Asi_MJ1_Clone4 TCATTTGTTACGCGCGATGAAAGAGTATCGGGACATCGTAAT--------

Asi_MJ1_Clone6 TCATTTGTTACGCGCGATGAAAGAGTATCGGGACATCGTAAT--------

Akl_MJ1_Clone3 TCATTTGTTACGCGCGATGAAAGAGTATCGGGACATCGTAAT--------

Aba_MJ1_Clone2 TCATTTGTTACGCGCGATGAAAGAGTATCGGGACATCGTAAT--------

Akw_MJ1_Clone3 TCATTTGTTACGCGCGATGAAAGAGTATCGGGACATCGTAAT--------

Aya_MJ1_Clone3 TCATTTGTTACGCGCGATGAAAGAGTATCGGGACATCGTAAT--------

Aju_MJ1_Clone1 TCATTTGTTACGCGCGATGAAAGAGTATCGGGACATCGTAAT--------

Asi_MJ1_Clone5 TCATTTGTTACGCGCGATGAAAGAGTATCGGGACATCGTAAT--------

Asi_MJ1_Clone9 TCATTTGTTACGCGCGATGAAAGAGTATCGGGACATCGTAAT--------

Akw_MJ1_Clone2 TCATTTGTTACGCGCGATGAAAGAGTATCGGGACATCGTAAT--------

Asi_MJ1_Clone8 TCATTTGTTACGCGCGATGAAAGAGTATCGGGACATCGTAAT--------

Akl_MJ1_Clone2 TCATTTGTTACGCGCGATGAAAGAGTATCGGGACATCGTAAT--------

Aya_MJ1_Clone1 TCATTTGTTACGCGCGATGAAAGAGTATCGGGACATCGTAAT--------

Akl_MJ1_Clone1 TCATTTGTTACGCGCGATGAAAGAGTATCGGGACATCGTAAT--------

Aba_MJ1_Clone3 TCATTTGTTACGCGCGATGAAAGAGTATCGGGACATCGTAAT--------

Aba_MJ1_Clone1 TCATTTGTTACGCGCGATGAAAGAGTATCGGGACATCGTAAT--------

Akw_MJ1_Clone1 TCATTTGTTACGCGCGATGAAAGAGTATCGGGACATCGTAAT--------

Aju_MJ1_Clone2 TCATTTGTTACGCGCGATGAAAGGGTATCGGGACATCGTAAT--------

Aju_MJ1_Clone7 TCATTTGTTACGCGCGATGAAAGAGTATCGGGACATCGTAAT--------

Ale_MJ1_Clone4 TCATTTGTTACGCGCGATGAAAGAGTATCGGGACATCGTAAT--------

Ale_MJ1_Clone10 TCATTTGTTACGCGCGATGAAAGAGTATCGGGACATCGTAAT--------

Aju_MJ1_Clone5 TCATTTGTTACGCGCGATGAAAGAGTATCGGGACATCGTAAT--------

Ale_MJ1_Clone5 TCATTTGTTACGCGCGATGAAAGAGTATCGGGACATCGTAAT--------

Aju_MJ1_Clone6 TCATTTGTTACGCGCGATGAAAGAGTATCGGGACATCGTAAT--------

Ale_MJ1_Clone3 TCATTTGTTACGCGCGATGAAAGAGTATCGGGACATCGTAAT--------

Ale_MJ1_Clone12 TCATTTGTTACGCGCGATGAAAGAGTATCGGGACATCGTAAT--------

Aju_MJ1_Clone4 TCATTTGTTACGCGCGATGAAAGAGTATCGGGACATCGTAAT--------

Aju_MJ1_Clone8 TCATTTGTTACGCGCGATGAAAGAGTATCGGGACATCGTAAT--------

Ale_MJ1_Clone9 TCATTTGTTACGCGCGATGAAAGAGTATCGGGACATCGTAAT--------

Ale_MJ1_Clone6 TCATTTGTTACGCGCGATGAAAGAGTATCGGGACATCGTAAT--------

Ale_MJ1_Clone13 TCATTTGTTACGCGCGATGAAAGAGTATCGGGACATCGTAAT--------

Ale_MJ1_Clone2 TCATCTGTTACGCGCGATGAAAGAGTATCGGGACATCGTAAT--------

Ape_MJ1_Clone1 TCATTTGTTACGCGCGATGAAAGAGTATCGGGACATCGTAAT--------

Ape_MJ1_Clone3 TCATTTGTTACGCGCGATGAAAGAGTATCGGGACATCGTAAT--------

CONTIG_574 TCGTTTGTTACGCGCGATGAAAGAGTATCGGAACTTCGTAAT--------

CONTIG_7401 TCGTTTGTTACGCGCGATGAAAGAGTATCGGGACTTCGTAATAAGCCATT

CONTIG_10225 --------------------------------------------------

CONTIG_29853 --------------------------------------------------

CONTIG_11920 --------------------------------------------------

CONTIG_8991 --------------------------------------------------

CONTIG_23766 --------------------------------------------------

CONTIG_13910 --------------------------------------------------

CONTIG_4960 --------------------------------------------------

Ape_MJ1_Clone6 --------------------------------------------------

Ape_MJ1_Clone7 --------------------------------------------------

Ape_MJ1_Clone2 --------------------------------------------------

Ale_MJ1_Clone1 --------------------------------------------------

Ale_MJ1_Clone11 --------------------------------------------------

Ahy_MJ1_Clone1 --------------------------------------------------

Ahy_MJ1_Clone2 --------------------------------------------------

Ale_MJ1_Clone8 --------------------------------------------------

Acr_MJ1_Clone2 --------------------------------------------------

Acr_MJ1_Clone3 --------------------------------------------------

Acr_MJ1_Clone1 --------------------------------------------------

Ape_MJ1_Clone4 --------------------------------------------------

Ape_MJ1_Clone5 --------------------------------------------------

Ahy_MJ1_Clone3 --------------------------------------------------

Ale_MJ1_Clone7 --------------------------------------------------

Aju_MJ1_Clone3 --------------------------------------------------

Aya_MJ1_Clone2 --------------------------------------------------

Asi_MJ1_Clone2 --------------------------------------------------

Asi_MJ1_Clone7 --------------------------------------------------

Asi_MJ1_Clone1 --------------------------------------------------

Asi_MJ1_Clone3 --------------------------------------------------

Asi_MJ1_Clone4 --------------------------------------------------

Asi_MJ1_Clone6 --------------------------------------------------

Akl_MJ1_Clone3 --------------------------------------------------

Aba_MJ1_Clone2 --------------------------------------------------

Akw_MJ1_Clone3 --------------------------------------------------

Aya_MJ1_Clone3 --------------------------------------------------

Aju_MJ1_Clone1 --------------------------------------------------

Asi_MJ1_Clone5 --------------------------------------------------

Asi_MJ1_Clone9 --------------------------------------------------

Akw_MJ1_Clone2 --------------------------------------------------

Asi_MJ1_Clone8 --------------------------------------------------

Akl_MJ1_Clone2 --------------------------------------------------

Aya_MJ1_Clone1 --------------------------------------------------

Akl_MJ1_Clone1 --------------------------------------------------

Aba_MJ1_Clone3 --------------------------------------------------

Aba_MJ1_Clone1 --------------------------------------------------

Akw_MJ1_Clone1 --------------------------------------------------

Aju_MJ1_Clone2 --------------------------------------------------

Aju_MJ1_Clone7 --------------------------------------------------

Ale_MJ1_Clone4 --------------------------------------------------

Ale_MJ1_Clone10 --------------------------------------------------

Aju_MJ1_Clone5 --------------------------------------------------

Ale_MJ1_Clone5 --------------------------------------------------

Aju_MJ1_Clone6 --------------------------------------------------

Ale_MJ1_Clone3 --------------------------------------------------

Ale_MJ1_Clone12 --------------------------------------------------

Aju_MJ1_Clone4 --------------------------------------------------

Aju_MJ1_Clone8 --------------------------------------------------

Ale_MJ1_Clone9 --------------------------------------------------

Ale_MJ1_Clone6 --------------------------------------------------

Ale_MJ1_Clone13 --------------------------------------------------

Ale_MJ1_Clone2 --------------------------------------------------

Ape_MJ1_Clone1 --------------------------------------------------

Ape_MJ1_Clone3 --------------------------------------------------

CONTIG_574 --------------------------------------------------

CONTIG_7401 TTATAAGACGTTTCAGGTCATATGTTTTTTGTTTGTTTACCAGCTTTGAA

CONTIG_10225 --------------------------------------------------

CONTIG_29853 --------------------------------------------------

CONTIG_11920 --------------------------------------------------

CONTIG_8991 --------------------------------------------------

CONTIG_23766 --------------------------------------------------

CONTIG_13910 --------------------------------------------------

CONTIG_4960 --------------------------------------------------

Ape_MJ1_Clone6 --------------------------------------------------

Ape_MJ1_Clone7 --------------------------------------------------

Ape_MJ1_Clone2 --------------------------------------------------

Ale_MJ1_Clone1 --------------------------------------------------

Ale_MJ1_Clone11 --------------------------------------------------

Ahy_MJ1_Clone1 --------------------------------------------------

Ahy_MJ1_Clone2 --------------------------------------------------

Ale_MJ1_Clone8 --------------------------------------------------

Acr_MJ1_Clone2 --------------------------------------------------

Acr_MJ1_Clone3 --------------------------------------------------

Acr_MJ1_Clone1 --------------------------------------------------

Ape_MJ1_Clone4 --------------------------------------------------

Ape_MJ1_Clone5 --------------------------------------------------

Ahy_MJ1_Clone3 --------------------------------------------------

Ale_MJ1_Clone7 --------------------------------------------------

Aju_MJ1_Clone3 --------------------------------------------------

Aya_MJ1_Clone2 --------------------------------------------------

Asi_MJ1_Clone2 --------------------------------------------------

Asi_MJ1_Clone7 --------------------------------------------------

Asi_MJ1_Clone1 --------------------------------------------------

Asi_MJ1_Clone3 --------------------------------------------------

Asi_MJ1_Clone4 --------------------------------------------------

Asi_MJ1_Clone6 --------------------------------------------------

Akl_MJ1_Clone3 --------------------------------------------------

Aba_MJ1_Clone2 --------------------------------------------------

Akw_MJ1_Clone3 --------------------------------------------------

Aya_MJ1_Clone3 --------------------------------------------------

Aju_MJ1_Clone1 --------------------------------------------------

Asi_MJ1_Clone5 --------------------------------------------------

Asi_MJ1_Clone9 --------------------------------------------------

Akw_MJ1_Clone2 --------------------------------------------------

Asi_MJ1_Clone8 --------------------------------------------------

Akl_MJ1_Clone2 --------------------------------------------------

Aya_MJ1_Clone1 --------------------------------------------------

Akl_MJ1_Clone1 --------------------------------------------------

Aba_MJ1_Clone3 --------------------------------------------------

Aba_MJ1_Clone1 --------------------------------------------------

Akw_MJ1_Clone1 --------------------------------------------------

Aju_MJ1_Clone2 --------------------------------------------------

Aju_MJ1_Clone7 --------------------------------------------------

Ale_MJ1_Clone4 --------------------------------------------------

Ale_MJ1_Clone10 --------------------------------------------------

Aju_MJ1_Clone5 --------------------------------------------------

Ale_MJ1_Clone5 --------------------------------------------------

Aju_MJ1_Clone6 --------------------------------------------------

Ale_MJ1_Clone3 --------------------------------------------------

Ale_MJ1_Clone12 --------------------------------------------------

Aju_MJ1_Clone4 --------------------------------------------------

Aju_MJ1_Clone8 --------------------------------------------------

Ale_MJ1_Clone9 --------------------------------------------------

Ale_MJ1_Clone6 --------------------------------------------------

Ale_MJ1_Clone13 --------------------------------------------------

Ale_MJ1_Clone2 --------------------------------------------------

Ape_MJ1_Clone1 --------------------------------------------------

Ape_MJ1_Clone3 --------------------------------------------------

CONTIG_574 --------------------------------------------------

CONTIG_7401 ACTTTCTGGCGGGCCCATCTATTTACGTTTCTTCTAGCGCCACATTTGGG

CONTIG_10225 --------------------------------------------------

CONTIG_29853 --------------------------------------------------

CONTIG_11920 --------------------------------------------------

CONTIG_8991 --------------------------------------------------

CONTIG_23766 --------------------------------------------------

CONTIG_13910 --------------------------------------------------

CONTIG_4960 --------------------------------------------------

Ape_MJ1_Clone6 --------------------------------------------------

Ape_MJ1_Clone7 --------------------------------------------------

Ape_MJ1_Clone2 --------------------------------------------------

Ale_MJ1_Clone1 --------------------------------------------------

Ale_MJ1_Clone11 --------------------------------------------------

Ahy_MJ1_Clone1 --------------------------------------------------

Ahy_MJ1_Clone2 --------------------------------------------------

Ale_MJ1_Clone8 --------------------------------------------------

Acr_MJ1_Clone2 --------------------------------------------------

Acr_MJ1_Clone3 --------------------------------------------------

Acr_MJ1_Clone1 --------------------------------------------------

Ape_MJ1_Clone4 --------------------------------------------------

Ape_MJ1_Clone5 --------------------------------------------------

Ahy_MJ1_Clone3 --------------------------------------------------

Ale_MJ1_Clone7 --------------------------------------------------

Aju_MJ1_Clone3 --------------------------------------------------

Aya_MJ1_Clone2 --------------------------------------------------

Asi_MJ1_Clone2 --------------------------------------------------

Asi_MJ1_Clone7 --------------------------------------------------

Asi_MJ1_Clone1 --------------------------------------------------

Asi_MJ1_Clone3 --------------------------------------------------

Asi_MJ1_Clone4 --------------------------------------------------

Asi_MJ1_Clone6 --------------------------------------------------

Akl_MJ1_Clone3 --------------------------------------------------

Aba_MJ1_Clone2 --------------------------------------------------

Akw_MJ1_Clone3 --------------------------------------------------

Aya_MJ1_Clone3 --------------------------------------------------

Aju_MJ1_Clone1 --------------------------------------------------

Asi_MJ1_Clone5 --------------------------------------------------

Asi_MJ1_Clone9 --------------------------------------------------

Akw_MJ1_Clone2 --------------------------------------------------

Asi_MJ1_Clone8 --------------------------------------------------

Akl_MJ1_Clone2 --------------------------------------------------

Aya_MJ1_Clone1 --------------------------------------------------

Akl_MJ1_Clone1 --------------------------------------------------

Aba_MJ1_Clone3 --------------------------------------------------

Aba_MJ1_Clone1 --------------------------------------------------

Akw_MJ1_Clone1 --------------------------------------------------

Aju_MJ1_Clone2 --------------------------------------------------

Aju_MJ1_Clone7 --------------------------------------------------

Ale_MJ1_Clone4 --------------------------------------------------

Ale_MJ1_Clone10 --------------------------------------------------

Aju_MJ1_Clone5 --------------------------------------------------

Ale_MJ1_Clone5 --------------------------------------------------

Aju_MJ1_Clone6 --------------------------------------------------

Ale_MJ1_Clone3 --------------------------------------------------

Ale_MJ1_Clone12 --------------------------------------------------

Aju_MJ1_Clone4 --------------------------------------------------

Aju_MJ1_Clone8 --------------------------------------------------

Ale_MJ1_Clone9 --------------------------------------------------

Ale_MJ1_Clone6 --------------------------------------------------

Ale_MJ1_Clone13 --------------------------------------------------

Ale_MJ1_Clone2 --------------------------------------------------

Ape_MJ1_Clone1 --------------------------------------------------

Ape_MJ1_Clone3 --------------------------------------------------

CONTIG_574 --------------------------------------------------

CONTIG_7401 AGGAGCTAATTCATTAATGGCATGCAACAAGCTTCTCAATCTTCCCCAGT

CONTIG_10225 --------------------------------------------------

CONTIG_29853 --------------------------------------------------

CONTIG_11920 --------------------------------------------------

CONTIG_8991 --------------------------------------------------

CONTIG_23766 --------------------------------------------------

CONTIG_13910 --------------------------------------------------

CONTIG_4960 --------------------------------------------------

Ape_MJ1_Clone6 --------------------------------------------------

Ape_MJ1_Clone7 --------------------------------------------------

Ape_MJ1_Clone2 --------------------------------------------------

Ale_MJ1_Clone1 --------------------------------------------------

Ale_MJ1_Clone11 --------------------------------------------------

Ahy_MJ1_Clone1 --------------------------------------------------

Ahy_MJ1_Clone2 --------------------------------------------------

Ale_MJ1_Clone8 --------------------------------------------------

Acr_MJ1_Clone2 --------------------------------------------------

Acr_MJ1_Clone3 --------------------------------------------------

Acr_MJ1_Clone1 --------------------------------------------------

Ape_MJ1_Clone4 --------------------------------------------------

Ape_MJ1_Clone5 --------------------------------------------------

Ahy_MJ1_Clone3 --------------------------------------------------

Ale_MJ1_Clone7 --------------------------------------------------

Aju_MJ1_Clone3 --------------------------------------------------

Aya_MJ1_Clone2 --------------------------------------------------

Asi_MJ1_Clone2 --------------------------------------------------

Asi_MJ1_Clone7 --------------------------------------------------

Asi_MJ1_Clone1 --------------------------------------------------

Asi_MJ1_Clone3 --------------------------------------------------

Asi_MJ1_Clone4 --------------------------------------------------

Asi_MJ1_Clone6 --------------------------------------------------

Akl_MJ1_Clone3 --------------------------------------------------

Aba_MJ1_Clone2 --------------------------------------------------

Akw_MJ1_Clone3 --------------------------------------------------

Aya_MJ1_Clone3 --------------------------------------------------

Aju_MJ1_Clone1 --------------------------------------------------

Asi_MJ1_Clone5 --------------------------------------------------

Asi_MJ1_Clone9 --------------------------------------------------

Akw_MJ1_Clone2 --------------------------------------------------

Asi_MJ1_Clone8 --------------------------------------------------

Akl_MJ1_Clone2 --------------------------------------------------

Aya_MJ1_Clone1 --------------------------------------------------

Akl_MJ1_Clone1 --------------------------------------------------

Aba_MJ1_Clone3 --------------------------------------------------

Aba_MJ1_Clone1 --------------------------------------------------

Akw_MJ1_Clone1 --------------------------------------------------

Aju_MJ1_Clone2 --------------------------------------------------

Aju_MJ1_Clone7 --------------------------------------------------

Ale_MJ1_Clone4 --------------------------------------------------

Ale_MJ1_Clone10 --------------------------------------------------

Aju_MJ1_Clone5 --------------------------------------------------

Ale_MJ1_Clone5 --------------------------------------------------

Aju_MJ1_Clone6 --------------------------------------------------

Ale_MJ1_Clone3 --------------------------------------------------

Ale_MJ1_Clone12 --------------------------------------------------

Aju_MJ1_Clone4 --------------------------------------------------

Aju_MJ1_Clone8 --------------------------------------------------

Ale_MJ1_Clone9 --------------------------------------------------

Ale_MJ1_Clone6 --------------------------------------------------

Ale_MJ1_Clone13 --------------------------------------------------

Ale_MJ1_Clone2 --------------------------------------------------

Ape_MJ1_Clone1 --------------------------------------------------

Ape_MJ1_Clone3 --------------------------------------------------

CONTIG_574 --------------------------------------------------

CONTIG_7401 AAAATTAAAATTAACAGGCAGGGAAGAATTATTTATTTTTGCTACTGGTC

CONTIG_10225 --------------------------------------------------

CONTIG_29853 --------------------------------------------------

CONTIG_11920 --------------------------------------------------

CONTIG_8991 --------------------------------------------------

CONTIG_23766 --------------------------------------------------

CONTIG_13910 --------------------------------------------------

CONTIG_4960 --------------------------------------------------

Ape_MJ1_Clone6 --------------------------------------------------

Ape_MJ1_Clone7 --------------------------------------------------

Ape_MJ1_Clone2 --------------------------------------------------

Ale_MJ1_Clone1 --------------------------------------------------

Ale_MJ1_Clone11 --------------------------------------------------

Ahy_MJ1_Clone1 --------------------------------------------------

Ahy_MJ1_Clone2 --------------------------------------------------

Ale_MJ1_Clone8 --------------------------------------------------

Acr_MJ1_Clone2 --------------------------------------------------

Acr_MJ1_Clone3 --------------------------------------------------

Acr_MJ1_Clone1 --------------------------------------------------

Ape_MJ1_Clone4 --------------------------------------------------

Ape_MJ1_Clone5 --------------------------------------------------

Ahy_MJ1_Clone3 --------------------------------------------------

Ale_MJ1_Clone7 --------------------------------------------------

Aju_MJ1_Clone3 --------------------------------------------------

Aya_MJ1_Clone2 --------------------------------------------------

Asi_MJ1_Clone2 --------------------------------------------------

Asi_MJ1_Clone7 --------------------------------------------------

Asi_MJ1_Clone1 --------------------------------------------------

Asi_MJ1_Clone3 --------------------------------------------------

Asi_MJ1_Clone4 --------------------------------------------------

Asi_MJ1_Clone6 --------------------------------------------------

Akl_MJ1_Clone3 --------------------------------------------------

Aba_MJ1_Clone2 --------------------------------------------------

Akw_MJ1_Clone3 --------------------------------------------------

Aya_MJ1_Clone3 --------------------------------------------------

Aju_MJ1_Clone1 --------------------------------------------------

Asi_MJ1_Clone5 --------------------------------------------------

Asi_MJ1_Clone9 --------------------------------------------------

Akw_MJ1_Clone2 --------------------------------------------------

Asi_MJ1_Clone8 --------------------------------------------------

Akl_MJ1_Clone2 --------------------------------------------------

Aya_MJ1_Clone1 --------------------------------------------------

Akl_MJ1_Clone1 --------------------------------------------------

Aba_MJ1_Clone3 --------------------------------------------------

Aba_MJ1_Clone1 --------------------------------------------------

Akw_MJ1_Clone1 --------------------------------------------------

Aju_MJ1_Clone2 --------------------------------------------------

Aju_MJ1_Clone7 --------------------------------------------------

Ale_MJ1_Clone4 --------------------------------------------------

Ale_MJ1_Clone10 --------------------------------------------------

Aju_MJ1_Clone5 --------------------------------------------------

Ale_MJ1_Clone5 --------------------------------------------------

Aju_MJ1_Clone6 --------------------------------------------------

Ale_MJ1_Clone3 --------------------------------------------------

Ale_MJ1_Clone12 --------------------------------------------------

Aju_MJ1_Clone4 --------------------------------------------------

Aju_MJ1_Clone8 --------------------------------------------------

Ale_MJ1_Clone9 --------------------------------------------------

Ale_MJ1_Clone6 --------------------------------------------------

Ale_MJ1_Clone13 --------------------------------------------------

Ale_MJ1_Clone2 --------------------------------------------------

Ape_MJ1_Clone1 --------------------------------------------------

Ape_MJ1_Clone3 --------------------------------------------------

CONTIG_574 --------------------------------------------------

CONTIG_7401 AGATATGTAACCGCGTAGAAAAATAGCCAGAAAGAGACGAAAACGTCATC

CONTIG_10225 --------------------------------------------------

CONTIG_29853 --------------------------------------------------

CONTIG_11920 --------------------------------------------------

CONTIG_8991 --------------------------------------------------

CONTIG_23766 --------------------------------------------------

CONTIG_13910 --------------------------------------------------

CONTIG_4960 --------------------------------------------------

Ape_MJ1_Clone6 --------------------------------------------------

Ape_MJ1_Clone7 --------------------------------------------------

Ape_MJ1_Clone2 --------------------------------------------------

Ale_MJ1_Clone1 --------------------------------------------------

Ale_MJ1_Clone11 --------------------------------------------------

Ahy_MJ1_Clone1 --------------------------------------------------

Ahy_MJ1_Clone2 --------------------------------------------------

Ale_MJ1_Clone8 --------------------------------------------------

Acr_MJ1_Clone2 --------------------------------------------------

Acr_MJ1_Clone3 --------------------------------------------------

Acr_MJ1_Clone1 --------------------------------------------------

Ape_MJ1_Clone4 --------------------------------------------------

Ape_MJ1_Clone5 --------------------------------------------------

Ahy_MJ1_Clone3 --------------------------------------------------

Ale_MJ1_Clone7 --------------------------------------------------

Aju_MJ1_Clone3 --------------------------------------------------

Aya_MJ1_Clone2 --------------------------------------------------

Asi_MJ1_Clone2 --------------------------------------------------

Asi_MJ1_Clone7 --------------------------------------------------

Asi_MJ1_Clone1 --------------------------------------------------

Asi_MJ1_Clone3 --------------------------------------------------

Asi_MJ1_Clone4 --------------------------------------------------

Asi_MJ1_Clone6 --------------------------------------------------

Akl_MJ1_Clone3 --------------------------------------------------

Aba_MJ1_Clone2 --------------------------------------------------

Akw_MJ1_Clone3 --------------------------------------------------

Aya_MJ1_Clone3 --------------------------------------------------

Aju_MJ1_Clone1 --------------------------------------------------

Asi_MJ1_Clone5 --------------------------------------------------

Asi_MJ1_Clone9 --------------------------------------------------

Akw_MJ1_Clone2 --------------------------------------------------

Asi_MJ1_Clone8 --------------------------------------------------

Akl_MJ1_Clone2 --------------------------------------------------

Aya_MJ1_Clone1 --------------------------------------------------

Akl_MJ1_Clone1 --------------------------------------------------

Aba_MJ1_Clone3 --------------------------------------------------

Aba_MJ1_Clone1 --------------------------------------------------

Akw_MJ1_Clone1 --------------------------------------------------

Aju_MJ1_Clone2 --------------------------------------------------

Aju_MJ1_Clone7 --------------------------------------------------

Ale_MJ1_Clone4 --------------------------------------------------

Ale_MJ1_Clone10 --------------------------------------------------

Aju_MJ1_Clone5 --------------------------------------------------

Ale_MJ1_Clone5 --------------------------------------------------

Aju_MJ1_Clone6 --------------------------------------------------

Ale_MJ1_Clone3 --------------------------------------------------

Ale_MJ1_Clone12 --------------------------------------------------

Aju_MJ1_Clone4 --------------------------------------------------

Aju_MJ1_Clone8 --------------------------------------------------

Ale_MJ1_Clone9 --------------------------------------------------

Ale_MJ1_Clone6 --------------------------------------------------

Ale_MJ1_Clone13 --------------------------------------------------

Ale_MJ1_Clone2 --------------------------------------------------

Ape_MJ1_Clone1 --------------------------------------------------

Ape_MJ1_Clone3 --------------------------------------------------

CONTIG_574 --------------------------------------------------

CONTIG_7401 ATCAACAAAAATGTTACCAGCGCCATTCACATATCATGCTAGTTTTTAAA

CONTIG_10225 --------------------------------------------------

CONTIG_29853 --------------------------------------------------

CONTIG_11920 --------------------------------------------------

CONTIG_8991 --------------------------------------------------

CONTIG_23766 --------------------------------------------------

CONTIG_13910 --------------------------------------------------

CONTIG_4960 --------------------------------------------------

Ape_MJ1_Clone6 --------------------------------------------------

Ape_MJ1_Clone7 --------------------------------------------------

Ape_MJ1_Clone2 --------------------------------------------------

Ale_MJ1_Clone1 --------------------------------------------------

Ale_MJ1_Clone11 --------------------------------------------------

Ahy_MJ1_Clone1 --------------------------------------------------

Ahy_MJ1_Clone2 --------------------------------------------------

Ale_MJ1_Clone8 --------------------------------------------------

Acr_MJ1_Clone2 --------------------------------------------------

Acr_MJ1_Clone3 --------------------------------------------------

Acr_MJ1_Clone1 --------------------------------------------------

Ape_MJ1_Clone4 --------------------------------------------------

Ape_MJ1_Clone5 --------------------------------------------------

Ahy_MJ1_Clone3 --------------------------------------------------

Ale_MJ1_Clone7 --------------------------------------------------

Aju_MJ1_Clone3 --------------------------------------------------

Aya_MJ1_Clone2 --------------------------------------------------

Asi_MJ1_Clone2 --------------------------------------------------

Asi_MJ1_Clone7 --------------------------------------------------

Asi_MJ1_Clone1 --------------------------------------------------

Asi_MJ1_Clone3 --------------------------------------------------

Asi_MJ1_Clone4 --------------------------------------------------

Asi_MJ1_Clone6 --------------------------------------------------

Akl_MJ1_Clone3 --------------------------------------------------

Aba_MJ1_Clone2 --------------------------------------------------

Akw_MJ1_Clone3 --------------------------------------------------

Aya_MJ1_Clone3 --------------------------------------------------

Aju_MJ1_Clone1 --------------------------------------------------

Asi_MJ1_Clone5 --------------------------------------------------

Asi_MJ1_Clone9 --------------------------------------------------

Akw_MJ1_Clone2 --------------------------------------------------

Asi_MJ1_Clone8 --------------------------------------------------

Akl_MJ1_Clone2 --------------------------------------------------

Aya_MJ1_Clone1 --------------------------------------------------

Akl_MJ1_Clone1 --------------------------------------------------

Aba_MJ1_Clone3 --------------------------------------------------

Aba_MJ1_Clone1 --------------------------------------------------

Akw_MJ1_Clone1 --------------------------------------------------

Aju_MJ1_Clone2 --------------------------------------------------

Aju_MJ1_Clone7 --------------------------------------------------

Ale_MJ1_Clone4 --------------------------------------------------

Ale_MJ1_Clone10 --------------------------------------------------

Aju_MJ1_Clone5 --------------------------------------------------

Ale_MJ1_Clone5 --------------------------------------------------

Aju_MJ1_Clone6 --------------------------------------------------

Ale_MJ1_Clone3 --------------------------------------------------

Ale_MJ1_Clone12 --------------------------------------------------

Aju_MJ1_Clone4 --------------------------------------------------

Aju_MJ1_Clone8 --------------------------------------------------

Ale_MJ1_Clone9 --------------------------------------------------

Ale_MJ1_Clone6 --------------------------------------------------

Ale_MJ1_Clone13 --------------------------------------------------

Ale_MJ1_Clone2 --------------------------------------------------

Ape_MJ1_Clone1 --------------------------------------------------

Ape_MJ1_Clone3 --------------------------------------------------

CONTIG_574 --------------------------------------------------

CONTIG_7401 ACAATAACAATGAGCGGCCCTCCAGAAAACGTCAACCGAATGTCTCGTAA

CONTIG_10225 ----------TAAGCGTTTTTT-GAACGGTGAGCGACCCGGCGATATATT

CONTIG_29853 ----------TAAGCGTTTTTT-GAACGGTGAGCGACCCGGCGATATATT

CONTIG_11920 ----------TAAGCGTTTTTT-GAACGGTGAGCGACCCGGCGATATATT

CONTIG_8991 ----------TAAGCGTTTTTT-GAACGGTGAGCGACCCGGCGATATATT

CONTIG_23766 ----------TAAGCGTTTTTT-GAACGGTGAGCGACCCGGCGATATATT

CONTIG_13910 ----------TAAGCGTTTTTT-GAACGGTGAGCGACCCGGCGATATATT

CONTIG_4960 ----------TAAGCGTTTTTT-GAACGGTGAGCGACCCGGCGATATATT

Ape_MJ1_Clone6 ----------TAAGCGTTTTTT-GAACGGTGAGCGACCCGGCGATATATT

Ape_MJ1_Clone7 ----------TAAGCGTTTTTT-GAACGGTGAGCGACCCGGCGATATATT

Ape_MJ1_Clone2 ----------TAAGCGTTTTTT-GAACGGTGAGCGACCCGGCGATATATT

Ale_MJ1_Clone1 ----------TAAGCGTTTTTT-GAACGGTGAGCGACCCGGCGATATATT

Ale_MJ1_Clone11 ----------TAAGCGTTTTTT-GAACGGTGAGCGACCCGGCGATATATT

Ahy_MJ1_Clone1 ----------TAAGCGTTTTTT-GAACGGTGAGCGACCCGGCGATATATT

Ahy_MJ1_Clone2 ----------TAAGCGTTTTTT-GAACGGTGAGCGACCCGGCGATATATT

Ale_MJ1_Clone8 ----------TAAGCGTTTTTT-AAACGGTGAGCGACCCGGCGATATATT

Acr_MJ1_Clone2 ----------TAAGCGTTTTTT-GAACGGTGAGCGACCCGGCGATATATT

Acr_MJ1_Clone3 ----------TAAGCGTTTTTT-GAACGGTGAGCGACCCGGCGATATATT

Acr_MJ1_Clone1 ----------TAAGCGTTTTTT-GAACGGTGAGCGACCCGGCGATATATT

Ape_MJ1_Clone4 ----------TAAGCGTTTTTTTGAACGGTGAGCGACCCGGCGATATATT

Ape_MJ1_Clone5 ----------TAAGCGTTTTTTTGAACGGTGAGCGACCCGGCGATATATT

Ahy_MJ1_Clone3 ----------TAAGCGTTTTTT-GAACGGTGAGCGACCCGGCGATATATT

Ale_MJ1_Clone7 ----------TAAGCGTTTTTT-GAACGGTGAGCGACCCGGCGATATATT

Aju_MJ1_Clone3 ----------TAAGCGTTTTTT-GAACGGTGAGCGACCCGGCGATATATT

Aya_MJ1_Clone2 ----------TAAGCGTTTTTT-GAACGGTGAGCGACCCGGCGATATATT

Asi_MJ1_Clone2 ----------TAAGCGTTTTTT-GAACGGTGAGCGACCCGGCGATATATT

Asi_MJ1_Clone7 ----------TAAGCGTTTTTT-GAACGGTGAGCGACCCGGCGATATATT

Asi_MJ1_Clone1 ----------TAAGCGTTTTTT-GAACGGTGAGCGACCCGGCGATATATT

Asi_MJ1_Clone3 ----------TAAGCGTTTTTT-GAACGGTGAGCGACCCGGCGATATATT

Asi_MJ1_Clone4 ----------TAAGCGTTTTTT-GAACGGTGAGCGACCCGGCGATATATT

Asi_MJ1_Clone6 ----------TAAGCGTTTTTT-GAACGGTGAGCGACCCGGCGATATATT

Akl_MJ1_Clone3 ----------TAAGCGTTTTTT-GAACGGTGAGCGACCCGGCGATATATT

Aba_MJ1_Clone2 ----------TAAGCGTTTTTT-GAACGGTGAGCGACCCGGCGATATATT

Akw_MJ1_Clone3 ----------TAAGCGTTTTTT-GAACGGTGAGCGACCCGGCGATATATT

Aya_MJ1_Clone3 ----------TAAGCGTTTTTT-GAACGGTGAGCGACCCGGCGATATATT

Aju_MJ1_Clone1 ----------TAAGCGTTTTTT-GAACGGTGAGCGACCCGGCGATATATT

Asi_MJ1_Clone5 ----------TAAGCGTTTTTT-GAACGGTGAGCGACCCGGCGATATATT

Asi_MJ1_Clone9 ----------TAAGCGTTTTTT-GAACGGTGAGCGACCCGGCGATATATT

Akw_MJ1_Clone2 ----------TAAGCGTTTTTT-GAACGGTGAGCGACCCGGCGATATATT

Asi_MJ1_Clone8 ----------TAAGCGTTTTTT-GAACGGTGAGCGACCCGGCGATATATT

Akl_MJ1_Clone2 ----------TAAGCGTTTTTT-GAACGGTGAGCGACCCGGCGATATATT

Aya_MJ1_Clone1 ----------TAAGCGTTTTTT-GAACGGTGAGCGACCCGGCGATATATT

Akl_MJ1_Clone1 ----------TAAGCGTTTTTT-GAACGGTGAGCGACCCGGCGATATATT

Aba_MJ1_Clone3 ----------TAAGCGTTTTTT-GAACGGTGAGCGACCCGGCGATATATT

Aba_MJ1_Clone1 ----------TAAGCGTTTTTT-GAACGGTGAGCGACCCGGCGATATATT

Akw_MJ1_Clone1 ----------TAAGCGTTTTTT-GAACGGTGAGCGACCCGGCGATATATT

Aju_MJ1_Clone2 ----------TAAGCGTTTTTT-GAACGGTGAGCGACCCGGCGATATATT

Aju_MJ1_Clone7 ----------TAAGCGTTTTTT-GAACGGTGAGCGACCCGGCGATATATT

Ale_MJ1_Clone4 ----------TAAGCGTTTTTT-GAACGGTGAGCGACCCGGCGATATATT

Ale_MJ1_Clone10 ----------TAAGCGTTTTTT-GAACGGTGAGCGACCCGGCGATATATT

Aju_MJ1_Clone5 ----------TAAGCGTTTTTT-GAACGGTGAGCGACCCGGCGATATATT

Ale_MJ1_Clone5 ----------TAAGCGTTTTTT-GAACGGTGAGCGACCCGGCGATATATT

Aju_MJ1_Clone6 ----------TAAGCGTTTTTT-GAACGGTGAGCGACCCGGCGATATATT

Ale_MJ1_Clone3 ----------TAAGCGTTTTTT-GAACGGTGAGCGACCCGGCGATATATT

Ale_MJ1_Clone12 ----------TAAGCGTTTTTT-GAACGGTGAGCGACCCGGCGATATATT

Aju_MJ1_Clone4 ----------TAAGCGTTTTTT-GAACGGTGAGCGACCCGGCGATATATT

Aju_MJ1_Clone8 ----------TAAGCGTTTTTT-GAACGGTGAGCGACCCGGCGATATATT

Ale_MJ1_Clone9 ----------TAAGCGTTTTTT-GAACGGTGAGCGACCCGGCGATATATT

Ale_MJ1_Clone6 ----------TAAGCGTTTTTT-GAACGGTGAGCGACCCGGCGATATATT

Ale_MJ1_Clone13 ----------TAAGCGTTTTTT-GAACGGTGAGCGACCCGGCGATATATT

Ale_MJ1_Clone2 ----------TAAGCGTTTTTT-GAACGGTGAGCGACCCGGCGATATATT

Ape_MJ1_Clone1 ----------TAAGCGTTTTTT-GAACGGTGAGCGACCCGGCGATATATT

Ape_MJ1_Clone3 ----------TAAGCGTTTTTT-GAACGGTGAGCGACCCGGCGATATATT

CONTIG_574 ----------TAAGCGTTTTTT-GAACGGTGAGCGACCCGGCGATATATT

CONTIG_7401 AATGGCTTATTAAGCGTTTTTT-GAACGGTGAGCGACCCGGCGATATATT

CONTIG_10225 CCGGCTGCTGAAATCGCATGGGGTCAAGCGGAACTTTGTCTACACGACCA

CONTIG_29853 CCGGCTGCTGAAATCGCATGGGGTCAAGCGGAACTTTGTCTACACGACCA

CONTIG_11920 CCGGCTGCTGAAATCGCATGGGGTCAAGCGGAACTTTGTCTACACGACCA

CONTIG_8991 CCGGCTGCTGAAATCGCATGGGGTCAAGCGGAACTTTGTCTACACGACCA

CONTIG_23766 CCGGCTGCTGAAATCGCATGGGGTCAAGCGGAACTTTGTCTACACGACCA

CONTIG_13910 CCGGCTGCTGAAATCGCATGGGGTCAAGCGGAACTTTGTCTGCACGACCA

CONTIG_4960 CCGGCTGCTGAAATCGCATGGGGTCAAGCGGATCTTTGTCTACACGACCA

Ape_MJ1_Clone6 CCGGCTGCTGAAATCGCATGGGGTCAAGCGGAACTTTGTCTACACGACCA

Ape_MJ1_Clone7 CCGGCTGCTGAAATCGCATGGGGTCAAGCGGAACTTTGTCTACACGACCA

Ape_MJ1_Clone2 CCGGCTGCTGAAATCGCATGGGGTCAAGCGGAACTTTGTCTACACGACCA

Ale_MJ1_Clone1 CCGGCTGCTGAAATCGCATGGGGTCAAACGGAACTTTGTCTACACGACCA

Ale_MJ1_Clone11 CCGGCTGCTGAAATCGCATGGGGTCAAACGGAACTTTGTCTACACGACCA

Ahy_MJ1_Clone1 CCGGCTGCTGAAATCGCATGGGGTCAAGCGGAACTTTGTCTACACGACCA

Ahy_MJ1_Clone2 CCGGCTGCTGAAATCGCATGGGGTCAAGCGGAACTTTGTCTACACGACCA

Ale_MJ1_Clone8 CCGGCTGCTGAAATCGCATGGGGTCAAGCGGAACTTTGTCTACACGACCA

Acr_MJ1_Clone2 CCGGCTGCCGAAATCGCATGGGGTCAAGCGGAACTTTGTCTACACGACCA

Acr_MJ1_Clone3 CCGGCTGCCGAAATCGCATGGGGTCAAGCGGAACTTTGTCTACACGACCA

Acr_MJ1_Clone1 CCGGCTGCTGAAATCGCATGGGGTCAAGCGGAACTTTGTCTACACGACCA

Ape_MJ1_Clone4 CCGGCTGCTGAAATCGCATGGGGTCAAACGGAACTTTGTCTACACGACCA

Ape_MJ1_Clone5 CCGGCTGCTGAAATCGCATGGGGTCAAACGGAACTTTGTCTACACGACCA

Ahy_MJ1_Clone3 CCGGCTGCTGAAATCGCGTGGGGTCAAGCGGAACTTTGTCTACACGACCA

Ale_MJ1_Clone7 CCGGCTGCTGAAATCGCATGGGGTCAAGCGGAACTTTGTCTACACGACCA

Aju_MJ1_Clone3 CCGGCTGCTGAAATCGCATGGGGTCAAGCGGAACTTTGTCTACACGACCA

Aya_MJ1_Clone2 CCGGCTGCTGAAATCGCATGGGGTCAAGCGGAACTTTGTCTACACGACCA

Asi_MJ1_Clone2 CCGGCTGCTGAAATCGCATGGGGTCAAGCGGAACTTTGTCTACACGACCA

Asi_MJ1_Clone7 CCGGCTGCTGAAATCGCATGGGGTCAAGCGGAACTTTGTCTACACGACCA

Asi_MJ1_Clone1 CCGGCTGCTGAAATCGCATGGGGTCAAGCGGAACTTTGTCTACACGACCA

Asi_MJ1_Clone3 CCGGCTGCTGAAATCGCATGGGGTCAAGCGGAACTTTGTCTACACGACCA

Asi_MJ1_Clone4 CCGGCTGCTGAAATCGCATGGGGTCAAGCGGAACTTTGTCTACACGACCA

Asi_MJ1_Clone6 CCGGCTGCTGAAATCGCATGGGGTCAAGCGGAACTTTGTCTACACGACCA

Akl_MJ1_Clone3 CCGGCTGCTGAAATCGCATGGGGTCAAGCGGAACTTTGTCTACACGACCA

Aba_MJ1_Clone2 CCGGCTGCTGAAATCGCATGGGGTCAAGCGGAACTTTGTCTACACGACCA

Akw_MJ1_Clone3 CCGGCTGCTGAAATCGCATGGGGTCAAGCGGAACTTTGTCTACACGACCA

Aya_MJ1_Clone3 CCGGCTGCTGAAATCGCATGGGGTCAAGCGGAACTTTGTCTACACGACCA

Aju_MJ1_Clone1 CCGGCTGCTGAAATCGCATGGGGTCAAGCGGAACTTTGTCTACACGACCA

Asi_MJ1_Clone5 CCGGCTGCTGAAATCGCATGGGGTCAAGCGGAACTTTGTCTACACGACCA

Asi_MJ1_Clone9 CCGGCTGCTGAAATCGCATGGGGTCAAGCGGAACTTTGTCTACACGACCA

Akw_MJ1_Clone2 CCGGCTGCTGAAATCGCATGGGGTCAAGCGGAACTTTGTCTACACGACCA

Asi_MJ1_Clone8 CCGGCTGCTGAAATCGCATGGGGTCAAGCGGAACTTTGTCTACACGACCA

Akl_MJ1_Clone2 CCGGCTGCTGAAATCGCATGGGGTCAAGCGGAACTTTGTCTACACGACCA

Aya_MJ1_Clone1 CCGGCTGCCGAAATCGCATGGGGTCAAGCGGAACTTTGTCTACACGACCA

Akl_MJ1_Clone1 CCGGCTGCTGAAATCGCATGGGGTCAAGCGGAACTTTGTCTACACGACCG

Aba_MJ1_Clone3 CCGGCTGCTGAAATCGCATGGGGTCAAGCGGAACTTTGTCTACACGACCA

Aba_MJ1_Clone1 CCGGCTGCTGAAATCGCATGGGGTCAAGCGGAACTTTGTCTACACGACCA

Akw_MJ1_Clone1 CCGGCTGCTGAAATCGCATGGGGTCAAGCGGAACTTTGTCTACACGACCA

Aju_MJ1_Clone2 CCGGCTGCTGAAATCGCATGGGGTCAAGCGGAACTTTGTCTACACGACCA

Aju_MJ1_Clone7 CCGGCTGCTGAAATCGCATGGGGTCAAGCGGAACTTTGTCTACACGACCA

Ale_MJ1_Clone4 CCGGCTGCTGAAATCGCATGGGGTCAAGCGGAACTTTGTCTACACGACCA

Ale_MJ1_Clone10 CCGGCTGCTGAAATCGCATGGGGTCAAGCGGAACTTTGTCTACACGACCA

Aju_MJ1_Clone5 CCGGCTGCTGAAATCGCATGGGGTCAAGCGGAACTTTGTCTACACGACCA

Ale_MJ1_Clone5 CCGGCTGCTGAAATCGCATGGGGTCAAGCGGAACTTTGTCTACACGACCA

Aju_MJ1_Clone6 CCGGCTGCTGAAATCGCATGGGGTCAAGCGGAACTTTGTCTACACGACCA

Ale_MJ1_Clone3 CCGGCTGCTGAAATCGCATGGGGTCAAGCGGAACTTTGTCTACACGACCA

Ale_MJ1_Clone12 CCGGCTGCTGAAATCGCATGGGGTCAAGCGGAACTTTGTCTACACGACCA

Aju_MJ1_Clone4 CCGGCTGCTGAAATCGCATGGGGTCAAGCGGAACTTTGTCTACACGACCA

Aju_MJ1_Clone8 CCGGCTGCTGAAATCGCATGGGGTCAAGCGGAACTTTGTCTACACGACCA

Ale_MJ1_Clone9 CCGGCTGCTGAAATCGCATGGGGTCAAGCGGAACTTTGTCTACACGACCA

Ale_MJ1_Clone6 CCGGCTGCTGAAATCGCATGGGGTCAAGCGGAACTTTGTCTACACGACCA

Ale_MJ1_Clone13 CCGGCTGCTGAAATCGCATGGGGTCAAGCGGAACTTTGTCTACACGACCA

Ale_MJ1_Clone2 CCGGCTGCTGAAATCGCATGGGGTCAAGCGGAACTTTGTCTACACGACCA

Ape_MJ1_Clone1 CCGGCTGCTGAAATCGCATGGGGTCAAGCGGAACTTTGTCTACACGACCA

Ape_MJ1_Clone3 CCGGCTGCTGAAATCGCATGGGGTCAAGCGGAACTTTGTCTACACGACCA

CONTIG_574 CCGGCTGCTGAAATCGCATGGGGTCAAGCGGAACTTTGTCTACACGACCA

CONTIG_7401 TCGGCTGCTGAAATCGCATGGGGTCAAGCGGAACTTTGTCTACACGACCA

CONTIG_10225 TCAGGCGATACCGGGAGACGTCCTCGACCAATGACCGTGCGAGATCCGGT

CONTIG_29853 TCAGGCGATACCGGGAGACGTCCTCGACCAATGACCGTGCGAGATCCGGT

CONTIG_11920 TCAGGCGATACCGGGAGACGTCCTCGACCAATGACCGTGCGAGATCCGGT

CONTIG_8991 TCAGGCGATACCGGGAGACGTCCTCGACCAATGACCGTGCGAGATCCGGT

CONTIG_23766 TCAGGCGATACCGGGAGACGTCCTCGACCAATGACCGTGCGAGATCCGGT

CONTIG_13910 TCAGGCGATACCGGGAGACGTCCTCGACCAATGACCGTGCGAGATCCGGT

CONTIG_4960 TCAGGCAATACCGGGAGACGTCCTCGACCAATGACCGTGCGAGATCCGGT

Ape_MJ1_Clone6 TCAGGCGATACCGGGAGACGTCCTCGACCAATGACCGTGCGAGATCCGGT

Ape_MJ1_Clone7 TCAGGCGATACCGGGAGACGTCCTCGACCAATGACCGTGCGAGATCCGGT

Ape_MJ1_Clone2 TCAGGCGATACCGGGAGACGTCCTCGACCAATGACCGTGCGAGGTCCGGT

Ale_MJ1_Clone1 TCAGGCGATACCGGGAGACGTCCTCGACCAATGACCGTGCGAGATCCGGT

Ale_MJ1_Clone11 TCAGGCGATACCGGGAGACGTCCTCGACCAATGACCGTGCGAGATCCGGT

Ahy_MJ1_Clone1 TCAGGCGATACCGGGAGACGTCCTCGACCAATGACCGTGCGAGATCCGGT

Ahy_MJ1_Clone2 TCAGGCGATACCGGGAGACGTCCTCGACCAATGACCGTGCGAGATCCGGT

Ale_MJ1_Clone8 TCAGGCGATACCGGGAGACGTCCTCGACCAATGACCGTGCGAGATCCGGT

Acr_MJ1_Clone2 TCAGGCGATACCGGGAGACGTCCTCGACCAATGACCGTGCGAGATCCGGT

Acr_MJ1_Clone3 TCAGGCGATACCGGGAGACGTCCTCGACCAATGACCGTGCGAGATCCGGT

Acr_MJ1_Clone1 TCAGGCGATACCGGGAGACGTCCTCGACCAATGACCGTGCGAGATCCGGT

Ape_MJ1_Clone4 TCAGGCGATACCGGGAGACGTCCTCGACCAATGACCGTGCGAGATCCGGT

Ape_MJ1_Clone5 TCAGGCGATACCGGGAGACGTCCTCGACCAATGACCGTGCGAGATCCGGT

Ahy_MJ1_Clone3 TCAGGCGATACCGGGAGACGTCCTCGACCAATGACCGTGCGAGATCCGGT

Ale_MJ1_Clone7 TCAGGCGATACCGGGAGACGTCCTCGACCAATGACCGTGCGAGATCCGGT

Aju_MJ1_Clone3 TCAGGCGATACCGGGAGACGTCCTCGACCAATGACCGTGCGAGATCCGGT

Aya_MJ1_Clone2 TCAGGCGATACCGGGAGACGTCCTCGACCAATGACCGTGCGAGATCCGGT

Asi_MJ1_Clone2 TCAGACGATACCGGGAGACGTCCTCGACCAATGACCGTGCGAGATCCGGT

Asi_MJ1_Clone7 TCAGACGATACCGGGAGACGTCCTCGACCAATGACCGTGCGAGATCCGGT

Asi_MJ1_Clone1 TCAGGCGATACCGGGAGACGTCCTCGACCAATGACCGTGCGAGATCCGGT

Asi_MJ1_Clone3 TCAGGCGATACCGGGAGACGTCCTCGACCAATGACCGTGCGAGATCCGGT

Asi_MJ1_Clone4 TCAGGCGATACCGGGAGACGTCCTCGACCAATGACCGTGCGAGATCCGGT

Asi_MJ1_Clone6 TCAGGCGATACCGGGAGACGTCCTCGACCAATGACCGTGCGAGATCCGGT

Akl_MJ1_Clone3 TCAGGCGATACCGGGAGACGTCCTCGACCAATGACCGTGCGAGATCCGGT

Aba_MJ1_Clone2 TCAGGCGATACCGGGAGACGTCCTCGACCAATGACCGTGCGAGATCCGGT

Akw_MJ1_Clone3 TCAGGCGATACCGGGAGACGTCCTCGACCAATGACCGTGCGAGATCCGGT

Aya_MJ1_Clone3 TCAGGCGATACCGGGAGACGTCCTCGACCAATGACCGTGCGAGATCCGGT

Aju_MJ1_Clone1 TCAGGCGATACCGGGAGACGTCCTCGACCAATGACCGTGCGAGATCCGGT

Asi_MJ1_Clone5 TCAGGCGATACCGGGAGACGTCCTCGACCAATGACCGTGCGAGATCCGGT

Asi_MJ1_Clone9 TCAGGCGATACCGGGAGACGCCCTCGACCAATGACCGTGCGAGATCCGGT

Akw_MJ1_Clone2 TCAGGCGATACCGGGAGACGTCCTCGACCAATGACCGTGCGAGATCCGGT

Asi_MJ1_Clone8 TCAGGCGATACCGGGAGACGTCCTCGACCAATGACCGTGCGAGATCCGGT

Akl_MJ1_Clone2 TCAGGCGATACCGGGAGACGTCCTCGACCAATGACCGTGCGAGATCCGGT

Aya_MJ1_Clone1 TCAGGCGATACCGGGAGACGTCCTCGACCAATGACCGTGCGAGATCCGGT

Akl_MJ1_Clone1 TCAGGCGATACCGGGAGACGTCCTCGACCAATGACCGTGCGAGATCCGGT

Aba_MJ1_Clone3 TCAGGCGATACCGGGAGACGTCCTCGACCAATGACCGTGCGAGATCCGGT

Aba_MJ1_Clone1 TCAGGCGATACCGGGAGACGTCTTCGACCAATGACCGTGCGAGATCCGGT

Akw_MJ1_Clone1 TCAGGCGATACCGGGAGACGTCCTCGACCAATGACCGTGCGAGATCCGGT

Aju_MJ1_Clone2 TCAGGCGATACCGGGAGACGTCCTCGACCAATGACCGTGCGAGATCCGGT

Aju_MJ1_Clone7 TCAGGCGATACCGGGAGACGTCCTCGACCAATGACCGTGCGAGATCCGGT

Ale_MJ1_Clone4 TCAGGCGATACCGGGAGACGTCCTCGACCAATGACCGTGCGAGATCCGGT

Ale_MJ1_Clone10 TCAGGCGATACCGGGAGACGTCCTCGACCAATGACCGTGCGAGATCCGGT

Aju_MJ1_Clone5 TCAGGCGATACCGGGAGACGTCCTCGACCAATGACCGTGCGAGATCCGGT

Ale_MJ1_Clone5 TCAGGCGATACCGGGAGACGTCCTCGACCAATGACCGTGCGAGATCCGGT

Aju_MJ1_Clone6 TCAGGCGATACCGGGAGACGTCCTCGACCAATGACCGTGCGAGATCCGGT

Ale_MJ1_Clone3 TCAGGCGATACCGGGAGACGTCCTCGACCAATGACCGTGCGAGATCCGGT

Ale_MJ1_Clone12 TCAGGCGATACCGGGAGACGTCCTCGACCAATGACCGTGCGAGATCCGGT

Aju_MJ1_Clone4 TCAGGCGATACCGGGAGACGTCCTCGACCAATGACCGTGCGAGATCCGGT

Aju_MJ1_Clone8 TCAGGCGATACCGGGAGACGTCCTCGACCAATGACCGTGCGAGATCCGGT

Ale_MJ1_Clone9 TCAGGCGATACCGGGAGACGTCCTCGACCAATGACCGTGCGAGATCCGGT

Ale_MJ1_Clone6 TCAGGCGATACCGGGAGACGTCCTCGACCAATGACCGTGCGAGATCCGGT

Ale_MJ1_Clone13 TCAGGCGATACCGGGAGACGTCCTCGACCAATGACCGTGCGAGATCCGGT

Ale_MJ1_Clone2 TCAGGCGATACCGGGAGACGTCCTCGACCAATGACCGTGCGAGATCCGGT

Ape_MJ1_Clone1 TCAGGCGATACCGGGAGACGTCCTCGACCAATGACCGTGCGAGATCCGGT

Ape_MJ1_Clone3 TCAGGCGATACCGGGAGACGTCCTCGACCAATGACCGTGCGAGATCCGGT

CONTIG_574 TCAGGCGATACTGGGAGACGTCCTCGACCAATGACCGTGCGAGATCCGGT

CONTIG_7401 TCAGGCGATACCGGGAGACGTCCTCGACCAATGACCGTGCGAGATCCGGT

CONTIG_10225 CGGCCGCGTTCAGCGAGGACGCCACGGGTCATCAAGATCTTGAGGGAGCG

CONTIG_29853 CGGCCGCGTTCAGCGAGGACGCCACGGGTCATCAAGATCGTGAGGGAGCG

CONTIG_11920 CGGCCGCGTTCAGCGAGGACGCCACGGGTCATCAAGATCGTGAGGGAGCG

CONTIG_8991 CGGCCGCGTTCAGCGAGGACGCCACGGGTCATCAAGATCGTGAGGGAGCG

CONTIG_23766 CGGCCGCGTTCAGCGAGGACGCCACGGGTCACCAAAGTTGTGAGGGAGCG

CONTIG_13910 CGGCCGCGTTCAGCGAGGACGCCACGGGTCATCAAGATCGTGAGGGAGCG

CONTIG_4960 CGGCCGCGTTCAGCGAGGACGCCACGGGTCATCAAGATCGTGAGGGAGCG

Ape_MJ1_Clone6 CGGCCGCGTTCAGCCAGGACGCCACGGGTCATCAAGATCGTGAGGGAGCG

Ape_MJ1_Clone7 CGGCCGCGTTCAGCGAGGACGCCACGGGTCATCAAGATCGTGAGGGAGCG

Ape_MJ1_Clone2 CGGCCGCGTTCAGCGAGGACGCCACGGGTCATCAAGATCGTGAGGGAGCG

Ale_MJ1_Clone1 CGGCCGCGTTCAGCGAGGACGCCACGGATCATCAAGATCGTGAGGGAGCG

Ale_MJ1_Clone11 CGGCCGCGTTCAGCGAGGACGCCACGGGTCATCAAGATCGTGAGGGAGCG

Ahy_MJ1_Clone1 CGGCCGCGTTCAGCGAGGACGCCACGGGTCATCAAGATCGTGAGGGAGCG

Ahy_MJ1_Clone2 CGGCCGCGTTCAGCGAGGACGCCACGGGTCATCAAGATCGTGAGGGAGCG

Ale_MJ1_Clone8 CGGCCGCGTTCAGCGAGGACGCCACGGGTCATCAAGATCGTGAGGGAGCG

Acr_MJ1_Clone2 CGGCCGCCTTAAGCGAGGACGCCACGGGTCATCAAGATCGTGAGGGAACG

Acr_MJ1_Clone3 CGGCCGCCTTAAGCGAGGACGCCACGGGTCATCAAGATCGTGAGGGAACG

Acr_MJ1_Clone1 CGGACGCCTTAAGCGAGGACGCCACGGGTCATCAAGATCGTGAGGGAGCG

Ape_MJ1_Clone4 CGGCCGCGTTCAGCGAGGACGCCACGGGTCATCAAGATCGTGAGGGAGCG

Ape_MJ1_Clone5 CGGCCGCGTTCAGCGAGGACGCCACGGGTCATCAAGATCGTGAGGGAGCG

Ahy_MJ1_Clone3 CGGCCGCGTTCAGCGAGGACGCCACGGGTCATCAAGATCGTGAGGGAGCG

Ale_MJ1_Clone7 CGGCCGCGTTCAGCGAGGACGCCACGGGTCATCAAGATCGTGAGGGAGCG

Aju_MJ1_Clone3 CGGCCGCGTTCAGCGAGGACGCCACGGGTCATCAAGATCGTGAGGGAGCG

Aya_MJ1_Clone2 CGGCCGCGTTCAGCGAGGACGCCACGGGTCATCAAGATCGTGAGGGAGCG

Asi_MJ1_Clone2 CGGCCGCGTTCAGCGAGGACGCCACGGGTCATCAAGATCGTGAGGGAGCG

Asi_MJ1_Clone7 CGGCCGCGTTCAGCGAGGACGCCACGGGTCATCAAGATCGTGAGGGAGCG

Asi_MJ1_Clone1 CGGCCGCGTTCAGCGAGGACGCCACGGGTCATCAAGATCGTGAGGGAGCG

Asi_MJ1_Clone3 CGGCCGCGTTCAGCGAGGACGCCACGGGTCATCAAGATCGTGAGGGAGCG

Asi_MJ1_Clone4 CGGCCGCGTTCAGCGAGGACGCCACGGGTCATCAAGATCGTGAGGGAGCG

Asi_MJ1_Clone6 CGGCCGCGTTCAGCGAGGACGCCACGGGTCATCAAGATCGTGAGGGAGCG

Akl_MJ1_Clone3 CGGCCGCGTTCAGCGAGGACGCCACGGGTCATCAAGATCGTGAGGGAGCG

Aba_MJ1_Clone2 CGGCCGCGTTCAGCGAGGACGCCACGGGTCATCAAGATCGTGAGGGAGCG

Akw_MJ1_Clone3 CGGCCGCGTTCAGCGAGGACGCCACGGGTCATCAAGATCGTGAGGGAGCG

Aya_MJ1_Clone3 CGGCCGCGTTCAGCGAGGACGCCACGGGTCATCAAGATCGTGAGGGAGCG

Aju_MJ1_Clone1 CGGCCGCGTTCAGCGAGGACGCCACGGGTCATCAAGATCGTGAGGGAGCG

Asi_MJ1_Clone5 CGGCCGCGTTCAGCGAGGACGCCACGGGTCATCAAGATCGTGAGGGAGCG

Asi_MJ1_Clone9 CGGCCGCGTTCAGCGAGGACGCCACGGGTCATCAAGATCGTGAGGGAGCG

Akw_MJ1_Clone2 CGGCCGCGTTCAGCGAGGACGCCACGGGTCATCAAGATCGTGAGGGAGCG

Asi_MJ1_Clone8 CGGCCGCGTTCAGCGAGGACGCCACGGGTCATCAAGATCGTGAGGGAGCG

Akl_MJ1_Clone2 CGGCCGCGTTCAGCGAGGACGCCACGGGTCATCAAGATCGTGAGGGAGCG

Aya_MJ1_Clone1 CGGCCGCGTTCAGCGAGGACGCCACGGGTCATCAAGATCGTGAGGGAGCG

Akl_MJ1_Clone1 CGGCCGCGTTCAGCGAGGACGCCACGGGTCATCAAGATCGTGAGGGAGCG

Aba_MJ1_Clone3 CGGCCGCGTTCAGCGAGGACGCCACGGGTCATCAAGATCGTGAGGGAGCG

Aba_MJ1_Clone1 CGGCCGCGTTCAGCGAGGACGCCACGGGTCATCAAGATCGTGAGGGAGCG

Akw_MJ1_Clone1 CGGCCGCGTTCGGCGAGGACGCCACGGGTCATCAAGATCGTGAGGGAGCG

Aju_MJ1_Clone2 CGGCCGCGTTCAGCGAGGACGCCACGGGTCATCAAGATCGTGAGGGAGCG

Aju_MJ1_Clone7 CGGCCGCGTTCAGCGAGGACGCCACGGGTCATCAAGATCGTGAGGGAGCG

Ale_MJ1_Clone4 CGGCCGCGTTCAGCGAGGACGCCACGGGTCATCAAGATCGTGAGGGAGCG

Ale_MJ1_Clone10 CGGCCGCGTTCAGCGAGGACGCCACGGGTCATCAAGATCGTGAGGGAGCG

Aju_MJ1_Clone5 CGGCCGCGTTCAGCGAGGACGCCACGGGTCATCAAGATCGTGAGGGAGCG

Ale_MJ1_Clone5 CGGCCGCGTTCAGCGAGGACGCCACGGGTCATCAAGATCGTGAGGGAGCG

Aju_MJ1_Clone6 CGGCCGCGTTCAGCGAGGACGCCACGGGTCATCAAGATCGTGAGGGAGCG

Ale_MJ1_Clone3 CGGCCGCGTTCAGCGAGGACGCCACGGGTCATCAAGATCGTGAGGGAGCG

Ale_MJ1_Clone12 CGGCCGCGTTCAGCGAGGACGCCACGGGTCATCAAGATCGTGAGGGAGCG

Aju_MJ1_Clone4 CGGCCGCGTTCAGCGAGGACGCCACGGGTCATCAAGATCGTGAGGGAGCG

Aju_MJ1_Clone8 CGGCCGCGTTCAGCGAGGACGCCACGGGTCATCAAGATCGTGAGGGAGCG

Ale_MJ1_Clone9 CGGCCGCGTTCAGCGAGGACGCCACGGGTCATCAAGATCGTGAGGGAGCG

Ale_MJ1_Clone6 CGGCCGCGTTCAGCGAGGACGCCACGGGTCATCAAGATCGTGAGGGAGCG

Ale_MJ1_Clone13 CGGCCGCGTTCAGCGAGGACGCCACGGGTCATCAAGATCGTGAGGGAGCG

Ale_MJ1_Clone2 CGGCCGCGTTCAGCGAGGACGCCACGGGTCATCAAGATCGTGAGGGAGCG

Ape_MJ1_Clone1 CGGCCGCGTTCAGCGAGGACGCCACGGGTCATCAAGATCGTGAGGGAGCG

Ape_MJ1_Clone3 CGGCCGCGTTCAGCGAGGACGCCACGGGTCATCAAGATCGTGAGGGAGCG

CONTIG_574 CGGCCGCGTTCAGCGAGGACGCCACGGGTCATCAAGATCGTGAGGGAGCG

CONTIG_7401 CGGCCGCGTTCAGCGAGGACGCCACGGGTCATCAAGATCGTGAGGGAGCG

CONTIG_10225 AATTCGGCGCAAAAAGAACCGCTCAATCCGGAAAACGGCTGCAGATC---

CONTIG_29853 AATTCGGCGCAAAAAGAACCCCTCAATC----------------------

CONTIG_11920 AATTCGGCGCAAAAAGAACCGCTCAATCTGGAAAACGGCTGCAGATC---

CONTIG_8991 AATTCGGCGCAAAAAGAACCGCTCAATCCGGAAAACGGCTGCAGATC---

CONTIG_23766 ATTCGGACGCAAAA-GAACCGCTCAATCCGGAAAACGGCTGCAGATC---

CONTIG_13910 AATTCGGCGCAAAAAGAACCGCTCAATCCGGAAAACGGCTGCAGATC---

CONTIG_4960 AATTCGGCGCAAAAAGAACCGCTCAATCCGGAAAACGGCTGCAGATC---

Ape_MJ1_Clone6 AATTCGGCGCAAAAAGAACCGCTCAATCCGGAAAACGGCTGCAGATC---

Ape_MJ1_Clone7 AATTCGGCGCAAAAAGAACCGCTCAATCCGGAAAACGGCTGCAGATC---

Ape_MJ1_Clone2 AATTCGGCGCAAAAAGAACCGCTCAATCCGGAAAACGGCTGCAGATC---

Ale_MJ1_Clone1 AATTCGGCGCAAAAAGAACCGCTCAATCCGGAAAATGGCTGCAGATC---

Ale_MJ1_Clone11 AATTCGGCGCAAAAAGAACCGCTCAATCCGGAAAACGGCTGCAGATC---

Ahy_MJ1_Clone1 AATTCGGCGCAAAAAGAACCGCTCAATCCGGAAAACGGCTGCAGATC---

Ahy_MJ1_Clone2 AATTCGGCGCAAAAAGAACCGCTCAATCCGGAAAACGGCTGCAGATC---

Ale_MJ1_Clone8 AATTCGGCGCAAAAAGAACCGCTCAATCCGGAAAACGGCTGCAGATC---

Acr_MJ1_Clone2 AATTCGGCGCAAAAAGAACCGCTCAATCCGGAAAACGGCTGCAGATC---

Acr_MJ1_Clone3 AATTCGGCGCAAAAAGAACCGCTCAATCCGGAAAACGGCTGCAGATC---

Acr_MJ1_Clone1 AATTCGGCGCAAAAAGAACCGCTCAATCCGGAAAACGGCTGCAGATC---

Ape_MJ1_Clone4 AATTCGGCGCAAAAAGAACCGCTCAATCCGGAAAACGGCTGCAGATTGCT

Ape_MJ1_Clone5 AATTCGGCGCAAAAAGAACCGCTCAATCCGGAAAACGGCTGCAGATTGCT

Ahy_MJ1_Clone3 AATTCGGCGCAAAAAGAACCGCTCAATCCGGAAAACGGCTGCAGATC---

Ale_MJ1_Clone7 AATTCGGCGCAAAAAGAACCGCTCAATCCGGAAAACGGCTGCAGATC---

Aju_MJ1_Clone3 AATTCGGCGCAAAAAGAACCGCTCAATCCGGAAAACGGCTGCAGATC---

Aya_MJ1_Clone2 AATTCGGCGCAAAAAGAACCGCTCAATCCGGAAAACGGCTGCAGATC---

Asi_MJ1_Clone2 AATTCGGCGCAAAAAGAACCGCTCAATCCGGAAAACGGCTGCAGATC---

Asi_MJ1_Clone7 AATTCGGCGCAAAAGGAACCGCTCAATCCGGAAAACGGCTGCAGATC---

Asi_MJ1_Clone1 AATTCGGCGCAAAAAGAACCGCTCAATCCGGAAAACGGCTGCAGATC---

Asi_MJ1_Clone3 AATTCGGCGCAAAAAGAACCGCTCAATCCGGAAAACGGCTGCAGATC---

Asi_MJ1_Clone4 AATTCGGCGCAAAAAGAACCGCTCAATCCGGAAAACGGCTGCAGATC---

Asi_MJ1_Clone6 AATTCGGCGCAAAAAGAACCGCTCAATCCGGAAAACGGCTGCAGATC---

Akl_MJ1_Clone3 AATTCGGCGCAAACAGAACCGCTCAATCCGGAAAACGGCTGCAGATC---

Aba_MJ1_Clone2 AATTCGGCGCAAAAAGAACCGCTCAATCCGGAAAACGGCTGCAGATC---

Akw_MJ1_Clone3 AATTCGGCGCAAAAAGAACCGCTCAATCCGGAAAACGGCTGCAGATC---

Aya_MJ1_Clone3 AATTCGGCGCAAAAAGAACCGCTCAATCCGGAAAACGGCTGCAGATC---

Aju_MJ1_Clone1 AATTCGGCGCAAAAAGAACCGCTCAATCCGGAAAACGGCTGCAGACC---

Asi_MJ1_Clone5 AATTCGGCGCAAAAAGAACCGCTCAATCCGGAAAACGGCTGCAGATC---

Asi_MJ1_Clone9 AATTCGGCGCAAAAAGAACCGCTCAATCCGGAAAACGGCTGCAGATC---

Akw_MJ1_Clone2 AATTCGGCGCAAAAAGAACCGCTCAATCCGGAAAACGGCTGCAGATC---

Asi_MJ1_Clone8 AATTCGGCGCAAAAAGAACCGCTCAATCCGGAAAATGGCTGCAGATC---

Akl_MJ1_Clone2 AATTCGGCGCAAAAAGAACCGCTCAATCCGGAAAACGGCTGCAGATC---

Aya_MJ1_Clone1 AATTCGGCGCAAAAAGAACCGCTCAATCCGGAAAACGGCTGCAGATC---

Akl_MJ1_Clone1 AATTCGGCGCAAAAAGAACCGCTCAATCCGGAAAACGGCTGCAGATC---

Aba_MJ1_Clone3 AATTCGGCGCAAAAAGAACCGCTCAATCCGGAAAACGGCTGCAGATC---

Aba_MJ1_Clone1 AATTCGGCGCAAAAAGAACCGCTCAATCCGGAAAACGGCTGCAGATC---

Akw_MJ1_Clone1 AATTCGGCGCAAAAAGAACCGCTCAATCCGGAAAACGGCTGCAGATC---

Aju_MJ1_Clone2 AATTCGGCGCAAAAAGAACCGCTCAATCCGGAAAACGGCTGCAGATC---

Aju_MJ1_Clone7 AATTCGGCGCAAAAAGAACCGCTCAATCCGGAAAACGGCTGCAGATC---

Ale_MJ1_Clone4 AATTCGGCGCAAAAAGAACCGCTCAATCCGGAAAACGGCTGCAGATC---

Ale_MJ1_Clone10 AATTCGGCGCAAAAAGAACCGCTCAATCCGGAAAACGGCTGCAGATC---

Aju_MJ1_Clone5 AATTCGGCGCAAAAAGAACCGCTCAATCCGGAAAACGGCTGCAGATC---

Ale_MJ1_Clone5 AATTCGGCGCAAAAAGAACCGCTCAATCCGGAAAACGGCTGCAGATC---

Aju_MJ1_Clone6 AATTCGGCGCAAAAAGAACCGCTCAATCCGGAAAACGGCTGCAGATC---

Ale_MJ1_Clone3 AATTCGGCGCAAAAAGAACCGCTCAATCCGGAAAACGGCTGCAGATC---

Ale_MJ1_Clone12 AATTCGGCGCAAAAAGAACCGCTCAATCCGGAAAACGGCTGCAGATC---

Aju_MJ1_Clone4 AATTCGGCGCAAAAAGAACCGCTCAATCCGGAAAACGGCTGCAGATC---

Aju_MJ1_Clone8 AATTCGGCGCAAAAAGAACCGCTCAATCCGGAAAACGGCTGCAGATC---

Ale_MJ1_Clone9 AATTCGGCGCAAAAAGAACCGCTCAATCCGGAAAACGGCTGCAGATC---

Ale_MJ1_Clone6 AATTCGGCGCAAAAAGAACCGCTCAATCCGGAAAACGGCTGCAGATC---

Ale_MJ1_Clone13 AATTCGGCGCAAAAAGAACCGCTCAATCCGGAAAACGGCTGCAGATC---

Ale_MJ1_Clone2 AATTCGGCGCAAAAAGAACCGCTCAATCCGGAAAACAGCTGCAGATC---

Ape_MJ1_Clone1 AATTCGGCGCAAAAAGAACCGCTCAATCCGGAAAACGGCTGCAGATC---

Ape_MJ1_Clone3 AATTCGGCGCAAAAAGAACCGCTCAATCCGGAAAACGGCTGCAGATC---

CONTIG_574 AATTCGGCGCAAAAAGAACCGCTCAATCTGGAAAACGGCTGCAGATC---

CONTIG_7401 AATTCGGCGCAAAAAGAACCGCTCAATCCGGAAAACGGCTGCAGATC---

CONTIG_10225 --TCAAC-GTTTCCATTGGAACCGCTCACACCATACTCATCAAGGACCTT

CONTIG_29853 --------------------------------------------------

CONTIG_11920 --TCAAC-GTTTCCATTGGAACCGCTCACACCATACTCATCAAGGACCTT

CONTIG_8991 --TCAAC-GTTTCCATTGGAACCGCTCACACCATACTCATCAAGGACCTT

CONTIG_23766 --TCAAC-GTTTCCATAGGAACCGCTCACACCATACTCATCAAGGACCTT

CONTIG_13910 --TCAAC-GTTTCCATTGGAACCGCTCACACCATACTCATCAAGGACCTT

CONTIG_4960 --TCAAC-GTTTCCATTGGAACCGCTCACACCATACTCATCAAGGACCTT

Ape_MJ1_Clone6 --TCAAC-GTTTCCATTGGAACCGCTCACACCATACTCACCAAGGACCTT

Ape_MJ1_Clone7 --TCAAC-GTTTCCATTGGAACCGCTCACACCATACTCACCAAGGGCCTT

Ape_MJ1_Clone2 --TCAAC-GTTTCCATTGGAACCGCTCACACCATACTCACCAAGGACCTT

Ale_MJ1_Clone1 --TCAAC-GTTTCCATTGGAACCGCTCACACCATACTCACCAAGGACCTT

Ale_MJ1_Clone11 --TCAAC-GTTTCCATTGGAACCGCTCACACCATACTCACCAAGGACCTT

Ahy_MJ1_Clone1 --TCAAC-GTTTCCATTGGAACCGCTCACACCATACTCACCAAGGACCTT

Ahy_MJ1_Clone2 --TCAAC-GTTTCCATTGGAACCGCTCACACCATACTCACCAAGGACCTT

Ale_MJ1_Clone8 --TCAAC-GTTTCCATTGGAACCGCTCACACCATACTCACCAAGGACCTT

Acr_MJ1_Clone2 --TCAAC-GTTTCCATTGGAACCGCTCACACCATACTCACCAAGGACCTT

Acr_MJ1_Clone3 --TCAAC-GTTTCCATTGGAACCGCTCACACCATACTCACCAAGGACCTT

Acr_MJ1_Clone1 --TCAAC-GTTTCCATTGGAACCGCTCACACCATACTCACCAAGGACCTT

Ape_MJ1_Clone4 GTTCTGCAGTTTCCATTGGAACCGCTCACACCATACTCACCAAGGACCTT

Ape_MJ1_Clone5 GTTCTGCAGTTTCCATTGGAACCGCTCACACCATACTCACCAAGGACCTT

Ahy_MJ1_Clone3 --TCAAC-GTTTCCATTGGAACCGCTCACACCATACTCACCAAGGACCTT

Ale_MJ1_Clone7 --TCAAC-GTTTCCATTGGAACCGCTCACACCATACTCACCAAGGACCTT

Aju_MJ1_Clone3 --TCAAC-GTTTCCATTGGAACCGCTCACACCATACTCACCAAGGACCTT

Aya_MJ1_Clone2 --TCAAC-GTTTCCATTGGAACCGCTCACACCATACTCACCAAGGACCTT

Asi_MJ1_Clone2 --TCAAC-GTTTCCATTGGAACCGCTCACACCATACTCACCAAGGACCTT

Asi_MJ1_Clone7 --TCAAC-GTTTCCATTGGAACCGCTCACACCATACTCACCAAGGACCTT

Asi_MJ1_Clone1 --TCAAC-GTTTCCATTGGAACCGCTCACACCATACTCACCAAGGACCTT

Asi_MJ1_Clone3 --TCAAC-GTTTCCATTGGAACCGCTCACACCATACTCACCAAGGACCTT

Asi_MJ1_Clone4 --TCAAC-GTTTCCATTGGAACCGCTCACACCATACTCACCAAGGACCTT

Asi_MJ1_Clone6 --TCAAC-GTTTCCATTGGAACCGCTCACACCATACTCACCAAGGACCTT

Akl_MJ1_Clone3 --TCAAC-GTTTCCATTGGAACCGCTCACACCATACTCACCAAGGACCTT

Aba_MJ1_Clone2 --TCAAC-GTTTCCATTGGAACCGCTCACACCATACTCACCAAGGACCTT

Akw_MJ1_Clone3 --TCAAC-GTTTCCATTGGAACCGCTCACACCATACTCACCAAGGACCTT

Aya_MJ1_Clone3 --TCAAC-GTTTCCATTGGAACCGCTCACACCATACTCACCAAGGACCTT

Aju_MJ1_Clone1 --TCAAC-GTTTCCATTGGAACCGCTCACACCATACTCACCAAGGACCTT

Asi_MJ1_Clone5 --TCAAC-GTTTCCATTGGAACCGCTCACACCATACTCACCAAGGACCTT

Asi_MJ1_Clone9 --TCAAC-GTTTCCATTGGAACCGCTCACACCATACTCACCAAGGACCTT

Akw_MJ1_Clone2 --TCAAC-GTTTCCATTGGAACCGCTCACACCATACTCACCAAGGACCTT

Asi_MJ1_Clone8 --TCAAC-GTTTCCATTGGAACCGCTCACACCATACTCACCAAGGACCTT

Akl_MJ1_Clone2 --TCAAC-GTTTCCATTGGAACCGCTCACACCATACTCACCAAGGACCTT

Aya_MJ1_Clone1 --TCAAC-GTTTCCATTGGAACCGCTCACACCATACTCACCAAGGACCTT

Akl_MJ1_Clone1 --TCAAC-GTTTCCATTGGAACCGCTCACACCATACTCACCAAGGACCTT

Aba_MJ1_Clone3 --TCAAC-GTTTCCATTGGAACCGCTCACACCATACTCACCAAGGACCTT

Aba_MJ1_Clone1 --TCAAC-GTTTCCATTGGAACCGCTCACACCATACTCACCAAGGACCTT

Akw_MJ1_Clone1 --TCAAC-GTTTCCATTGGAACCGCTCACACCATACTCACCAAGGACCTT

Aju_MJ1_Clone2 --TCAAC-GTTTCCATTGGAACCGCTCACACCATACTCACCAAGGACCTT

Aju_MJ1_Clone7 --TCAAC-GTTTCCATTGGAACCGCTCACACCATACTCACCAAGGACCTT

Ale_MJ1_Clone4 --TCAAC-GTTTCCATTGGAACCGCTCACACCATACTCACCAAGGACCTT

Ale_MJ1_Clone10 --TCAAC-GTTTCCATTGGAACCGCTCACACCATACTCACCAAGGACCTT

Aju_MJ1_Clone5 --TCAAC-GTTTCCATTGGAACCGCTCACACCATACTCACCAAGGACCTT

Ale_MJ1_Clone5 --TCAAC-GTTTCCATTGGAACCGCTCACACCATACTCACCAAGGACCTT

Aju_MJ1_Clone6 --TCAAC-GTTTCCATTGGAACCGCTCACACCATACTCACCAAGGACCTT

Ale_MJ1_Clone3 --TCAAC-GTTTCCATTGGAACCGCTCACACCATACTCACCAAGGACCTT

Ale_MJ1_Clone12 --TCAAC-GTTTCCATTGGAACCGCTCACACCATACTCACCAAGGACCTT

Aju_MJ1_Clone4 --TCAAC-GTTTCCATTGGAACCGCTCACACCATACTCACCAAGGACCTT

Aju_MJ1_Clone8 --TCAAC-GTTTCCATTGGAACCGCTCACACCATACTCACCAAGGACCTT

Ale_MJ1_Clone9 --TCAAC-GTTTCCATTGGAACCGCTCACACCATACTCACCAAGGACCTT

Ale_MJ1_Clone6 --TCAAC-GTTTCCATTGGAACCGCTCACACCATACTCACCAAGGACCTT

Ale_MJ1_Clone13 --TCAAC-GTTTCCATTGGAACCGCCCACACCATACTCACCAAGGACCTT

Ale_MJ1_Clone2 --TCAAC-GTTTCCATTGGAACCGCTCACACCATACTCACCAAGGACCTT

Ape_MJ1_Clone1 --TCAAC-GTTTCCATTGGAACCGCTCACACCATACTCATCAAGGACCTT

Ape_MJ1_Clone3 --TCAAC-GTTTCCATTGGAACCGCTCACACCATACTCATCAAGGACCTT

CONTIG_574 --TCAAC-GTTTCCATTGGAACCGCTCACACCATACTCATCAAGGACCTT

CONTIG_7401 --TCAAC-GTTTCCATTGGAACCGCTCACACCATACTCATCAAGGACCTT

CONTIG_10225 GGTTTCAGGCCTTACAAAAAACGTAAGGTCCATGGCGTTTCGGAGGCTAC

CONTIG_29853 -----------------------------------------GGAGGCTAC

CONTIG_11920 GGTTTCAGGCCTTACAAAAAACGTAAGGTCCATGGCGTTTCGGAGGCTAC

CONTIG_8991 GGTTTCAGGCCTTACAAAAAACGTAAGGTCCATGGCGTTTCGGAGGCTAC

CONTIG_23766 GGTTTCAGGCCTTACAAAAAACGTAAGGTCCATGGCGTTTCGGAGGCTAC

CONTIG_13910 GGTTTCAGGCCTTACAAAAAACGTAAGGTCCATGGCGTTTCGGAGGCTAC

CONTIG_4960 GGTTTCAGGCCTTACAAAAAACGTAAGGTCCATGGCGTTTCGGAGGCTAC

Ape_MJ1_Clone6 GGTTTCAGGCCTTACAAAAAACGTAAGGTCCATGGCGTTTCGGAGGCTAC

Ape_MJ1_Clone7 GGTTTCAGGCCTTACAAAAAACGTAAGGTCCATGGCGTTTCGGAGGCTAC

Ape_MJ1_Clone2 GGTTTCAGGCCTTACAAAAAACGTAAGGTCCATGGCGTTTCGGAGGCTAC

Ale_MJ1_Clone1 GGTTTCAGGCCTTACAAAAAACGTAAGGTCCATGGCGTTTCGGAGGCTAC

Ale_MJ1_Clone11 GGTTTCAGGCCTTACAAAAAACGTAAGGTCCATGGCGTTTCGGAGGCTAC

Ahy_MJ1_Clone1 GGTTTCAGGCCTTACAAAAAACGTAAGGTCCATGGCGTTTCGGAGGCTAC

Ahy_MJ1_Clone2 GGTTTCAGGCCTTACAAAAAACGTAAGGTCCATGGCGTTTCGGAGGCTAC

Ale_MJ1_Clone8 GGTTTCAGGCCTTACAAAAAACGTAAGGTCCATGGCGTTTCGGAGGCTAC

Acr_MJ1_Clone2 GGTTTCAGGCCTTACAAAAAACCTAAGGTCCATGGCGTTTCGGAGGCTAC

Acr_MJ1_Clone3 GGTTTCAGGCCTTACAAAAAACCTAAGGTCCATGGCGTTTCGGAGGCTAC

Acr_MJ1_Clone1 GGTTTCAGGCCTTACAAAAAACGTAAGGTCCATGGCGTTTCGGAGGCTAC

Ape_MJ1_Clone4 GGTTTCAGGCCTTACAAAAAACGTAAGGTCCATGGCGTTTCGGAGGCTAC

Ape_MJ1_Clone5 GGTTTCAGGCCTTACAAAAAACGTAAGGTCCATGGCGTTTCGGAGGCTAC

Ahy_MJ1_Clone3 GGTTTCAGGCCTTACAAAAAACGTAAGGTCCATGGCGTTTCGGAGGCTAC

Ale_MJ1_Clone7 GGTTTCAGGCCTTACAAAGAACGTAAGGTCCATGGCGTTTCGGAGGCTAC

Aju_MJ1_Clone3 GGTTTCAGGCCTTACAAAAAACGTAAGGTCCATGGCGTTTCGGAGGCTAC

Aya_MJ1_Clone2 GGTTTCAGGCCTTACAAAAAACGTAAGGTCCATGGCGTTTCGGAGACTAC

Asi_MJ1_Clone2 GGTTTCAGGCCTTACAAAAAACGTAAGGTCCATGGCGTTTCGGAGGCTAC

Asi_MJ1_Clone7 GGTTTCAGGCCTTACAAAAAACGTAAGGTCCATGGCGTTTCGGAGGCTAC

Asi_MJ1_Clone1 GGTTTCAGGCCTTACAAAAAACGTAAGGTCCATGGCGTTTCGGAGGCTAC

Asi_MJ1_Clone3 GGTTTCAGGCCTTACAAAAAACGTAAGGTCCATGGCGTTTCGGAGGCTAC

Asi_MJ1_Clone4 GGTTTCAGGCCTTACAAAAAACGTAAGGTCCATGGCGTTTCGGAGGCTAC

Asi_MJ1_Clone6 GGTTTCAGGCCTTACAAAAAACGTAAGGTCCATGGCGTTTCGGAGGCTAC

Akl_MJ1_Clone3 GGTTTCAGGCCTTACAAAAAACGTAAGGTCCATGGCGTTTCGGAGGCTAC

Aba_MJ1_Clone2 GGTTTCAGGCCTTACAAAAAACGTAAGGTCCATGGCGTTTCGGAGGCTAC

Akw_MJ1_Clone3 GGTTTCAGGCCTTACAAAAAACGTAAGGTCCATGGCGTTTCGGAGGCTAC

Aya_MJ1_Clone3 GGTTTCAGGCCTTACAAAAAACGTAAGGTCCATGGCGTTTCGGAGGCTAC

Aju_MJ1_Clone1 GGTTTCAGGCCTTACAAAAAACGTAAGGTCCATGGCGTTTCGGAGGCTAC

Asi_MJ1_Clone5 GGTTTCAGGCCTTACAAAAAACGTAAGGTCCATGGCGTTTCGGAGGCTAC

Asi_MJ1_Clone9 GGTTTCAGGCCTTACAAAAAACGTAAGGTCCATGGCGTTTCGGAGGCTAC

Akw_MJ1_Clone2 GGTTTCAGGCCTTACAAGAAACGTAAGGTCCATGGCGTTTCGGAGGCTAC

Asi_MJ1_Clone8 GGTTTCAGGCCTTACAAAAAACGTAAGGTCCATGGCGTCTCGGAGGCTAC

Akl_MJ1_Clone2 GGTTTCAGGCCTTACAAAAAACGTAAGGTCCATGGCGTTTCGGAGGCTAC

Aya_MJ1_Clone1 GGTTTCAGGCCTTACAAAAAACGTAAGGTCCATGGCGTTTCGGAGGCTAC

Akl_MJ1_Clone1 GGTTTCAGGCCTTACAAAAAACGTAAGGTCCATGGCGTTTCGGAGGCTAC

Aba_MJ1_Clone3 GGTTTCAGGCCTTACAAAAAACGTAAGGTCCATGGCGTTTCGGAGGCTAC

Aba_MJ1_Clone1 GGTTTCAGGCCTTACAAAAAACGTAAGGTCCATGGCGTTTCGGAGGCTAC

Akw_MJ1_Clone1 GGTTTCAAGCCTTACAAAAAACGTAAGGTCCATGGCGTTTCGGAGGCTAC

Aju_MJ1_Clone2 GGTTTCAGGCCTTACAAAAAACGTAAGGTCCATGGCGTTTCGGAGGCTAC

Aju_MJ1_Clone7 GGTTTCAGGCCTTACAAAAAACGTAAGGTCCATGGCGTTTTGGAGGCTAC

Ale_MJ1_Clone4 GGTTTCAGGCCTTACAAAAAACGTAAGGTCCATGGCGTTTCGGAGGCTAC

Ale_MJ1_Clone10 GGTTTCAGGCCTTACAAAAAACGTAAGGTCCATGGCGTTTCGGAGGCTAC

Aju_MJ1_Clone5 GGTTTCAGGCCTTACAAAAAACGTAAGGTCCATGGCGTTTCGGAGGCTAC

Ale_MJ1_Clone5 GGTTTCAGGCCTTACAAAAAACGTAAGGTCCATGGCGTTTCGGAGGCTAC

Aju_MJ1_Clone6 GGTTTCAGGCCTTACAAAAAACGTAAGGTCCATGGCGTTTCGGAGGCTAC

Ale_MJ1_Clone3 GGTTTCAGGCCTTACAAAAAACGTAAGGTCCATGGCGTTTCGGAGGCTAC

Ale_MJ1_Clone12 GGTTTCAGGCCTTACAAAAAACGTAAGGTCCATGGCGTTTCGGAGGCTAC

Aju_MJ1_Clone4 GGTTTCAGGCCTTACAAAAAACGTAAGGTCCATGGCGTTTCGGAGGCTAC

Aju_MJ1_Clone8 GGTTTCAGGCCTTACAAAAAACGTAAGGTCCATGGCGTTTCGGAGGCTAC

Ale_MJ1_Clone9 GGTTTCAGGCCTTACAAAAAACGTAAGGTCCATGGCGTTTCGGAGGCTAC

Ale_MJ1_Clone6 GGTTTCAGGCCTTACAAAAAACGTAAGGTCCATGGCGTTTCGGAGGCTAC

Ale_MJ1_Clone13 GGTTTCAGGCCTTACAAAAAACGTAAGGTCCATGGCGTTTCGGAGGCTAC

Ale_MJ1_Clone2 GGTTTCAGGCCTTACAAAAAACGTAAGGTCCATGGCGTTTCGGAGGCTAC

Ape_MJ1_Clone1 GGTTTCAGGCCTTACAAAAAACGTAAGGTCCATGGCGTTTCGGAGGCTAC

Ape_MJ1_Clone3 GGTTTCAGGCCTTACAAAAAACGTAAGGTCCATGGCGTTTCGGAGGCTAC

CONTIG_574 GGTTTCAGGCCTTACAAAAAACGTAAGATCCATGGCGTTTCGGAGGCTAC

CONTIG_7401 GGTTTCAGGCCTTACAAAAAACGTAAGGTCCATGGCGTTTCGGAGGCTAC

CONTIG_10225 CAGCAAAAAGCGGTTGGATCGAGCTAAGAGGATCCTCTCTCGGCACGCTG

CONTIG_29853 CAGCAAAAAGCGGTTGGATCGAGCTAAGAGGATCCTCTCTCGGCACGCTG

CONTIG_11920 CAGCAAAAAGCGGTTGGATCGAGCTAAGAGGATCCTCTCTCGGCACGCTG

CONTIG_8991 CAGCAAAAAGCGGTTGAATCGAGCTAAGAGGATCCTCTCTCGGCACGCTG

CONTIG_23766 CAGCAAAAAGCGGTTGGATCGAGCTAAGAGGATCCTCTCTCGGCACGCTG

CONTIG_13910 CAGCAAAAAGCGGTTGGATCGAGCTAAGAGGATCCTCTCTCGGCACGCTG

CONTIG_4960 CAGCAAAAAGCGGTTGGATCGAGCTAAGAGGATCCTCTCTCGGCACGCTG

Ape_MJ1_Clone6 CAGCAAAAAGCGGTTGGATCGAGCTAAGAGGATCCTCTCTCGGCACGCTG

Ape_MJ1_Clone7 CAGCAAAAAGCGGTTGGATCGAGCTAAGAGGATCCTCTCTCGGCACGCTG

Ape_MJ1_Clone2 CAGCAAAAAGCGGTTGGATCGAGCTAAGAGGATCCTCTCTCGGCACGCTG

Ale_MJ1_Clone1 CAGCAAAAAGCGGTTGGATCGAGCTAAGAGGATCCTCTCTCGGCACGCTG

Ale_MJ1_Clone11 CAGCAAAAAGCGGTTGGATCGAGCTAAGAGGATCCTCTCTCGGCACGCTG

Ahy_MJ1_Clone1 CAGCAAAAAGCGGTTGGATCGAGCTAAGAGGATCCTCTCTCGGCACGCTG

Ahy_MJ1_Clone2 CAGCAAAAAGCGGTTGGATCGAGCTAAGAGGATCCTCTCTCGGCACGCTG

Ale_MJ1_Clone8 CAGCAAAAAGCGGTTGGATCGAGCTAAGAGGATCCTCTCTCGGCACGCTG

Acr_MJ1_Clone2 CAGCAAAAAGCGGTTGGATCGAGCTAAGAGGATCCTCTCTCGGCACGCTG

Acr_MJ1_Clone3 CAGCAAAAAGCGGTTGGATCGAGCTAAGAGGATCCTCTCTCGGCACGCTG

Acr_MJ1_Clone1 CAGCAAAAAGCGGTTGGATCGAGCTAAGAGGATCCTCTCTCGGCACGCTG

Ape_MJ1_Clone4 CAGCAAAAAGCGGTTGGATCGAGCTAATAGGAT-----CTCGGCACGCTG

Ape_MJ1_Clone5 CAGCAAAAAGCGGTTGGATCGAGCTAATAGGAT-----CTCGGCACGCTG

Ahy_MJ1_Clone3 CAGCAAAAAGCGGTTGGATCGAGCTAAGAGGATCCTCTCTCGGCACGCTG

Ale_MJ1_Clone7 CAGCAAAAAGCGGTTGGATCGAGCTAAGAGGATCCTCTCTCGGCACGCTG

Aju_MJ1_Clone3 CAGCAAAAAGCGGTTGGATCGAGCTAAGAGGATCCTCTCTCGGCACGCTG

Aya_MJ1_Clone2 CAGCAAAAAGCGGTTGGATCGAGCTAAGAGGATCCTCTCTCGGCACGCTG

Asi_MJ1_Clone2 CAGCAAAAAGCGGTTGGATCGAGCTAAGAGGATCCTCTCTCGGCACGCTG

Asi_MJ1_Clone7 CAGCAAAAAGCGGTTGGATCGAGCTAAGAGGATCCTCTCTCGGCACGCTG

Asi_MJ1_Clone1 CAGCAAAAAGCGGTTGGATCGAGCTAAGAGGATCCTCTCTCGGCACGCTG

Asi_MJ1_Clone3 CAGCAAAAAGCGGTTGGATCGAGCTAAGAGGATCCTCTCTCGGCACGCTG

Asi_MJ1_Clone4 CAGCAAAAAGCGGTTGGATCGAGCTAAGAGGATCCTCTCTCGGCACGCTG

Asi_MJ1_Clone6 CAGCAAAAAGCGGTTGGATCGAGCTAAGAGGATCCTCTCTCGGCACGCTG

Akl_MJ1_Clone3 CAGCAAAAAGCGGTTGGATCGAGCTAAGAGGATCCTCTCTCGGCACGCTG

Aba_MJ1_Clone2 CAGCAAAAAGCGGTTGGATCGAGCTAAGAGGATCCTCTCTCGGCACGCTG

Akw_MJ1_Clone3 CAGCAAAAAGCGGTTGGATCGAGCTAAGAGGATCCTCTCTCGGCACGCTG

Aya_MJ1_Clone3 CAGCAAAAAGCGGTTGGATCGAGCTAAGAGGATCCTCTCTCGGCACGCTG

Aju_MJ1_Clone1 CAGCAAAAAGCGGTTGGATCGAGCTAAGAGGATCCTCTCTCGGCACGCTG

Asi_MJ1_Clone5 CAGCAAAAAGCGGTTGGATCGAGCTAAGAGGATCCTCTCTCGGCACGCTG

Asi_MJ1_Clone9 CAGCAAAAAGCGGTTGGATCGAGCTAAGAGGATCCTCTCTCGGCACGCTG

Akw_MJ1_Clone2 CAGCAAAAAGCGGTTGGATCGAGCTAAGAGGATCCTCTCTCGGCACGCTG

Asi_MJ1_Clone8 CAGCAAAAAGCGGTTGGATCGAGCTAAGAGGATCCTCTCTCGGCACGCTG

Akl_MJ1_Clone2 CAGCAAAAAGCGGTTGGATCGAGCTAAGAGGATCCTCTCTCGGCACGCTG

Aya_MJ1_Clone1 CAGCAAAAAGCGGTTGGATCGAGCTAAGAGGATCCTCTCTCGGCACGCTG

Akl_MJ1_Clone1 CAGCAAAAAGCGGTTGGATCGAGCTAAGAGGATCCTCTCTCGGCACGCTG

Aba_MJ1_Clone3 CAGCAAAAAGCGGTTGGATCGAGCTAAGAGGATCCTCTCTCGGCACGCTG

Aba_MJ1_Clone1 CAGCAAAAAGCGGTTGGATCGAGCTAAGAGGATCCTCTCTCGGCACGCTG

Akw_MJ1_Clone1 CAGCAAAAAGCGGTTGGATCGAGCTAAGAGGATCCTCTCTCGGCACGCTG

Aju_MJ1_Clone2 CAGCAAAAAGCGGTTGGATCGAGCTAAGAGGATCCTCTCTCGGCACGCTG

Aju_MJ1_Clone7 CAGCAAAAAGCGGTTGGATCGAGCTAAGAGGATCCTCTCTCGGCACGCTG

Ale_MJ1_Clone4 CAGCAAAAAGCGGTTGGATCGAGCTAAGAGGATCCTCTCTCGGCACGCTG

Ale_MJ1_Clone10 CAGCAAAAAGCGGTTGGATCGAGCTAAGAGGATCCTCTCTCGGCACGCTG

Aju_MJ1_Clone5 CAGCAAAAAGCGGTTGGATCGAGCTAAGAGGATCCTCTCTCGGCACGCTG

Ale_MJ1_Clone5 CAGCAAAAAGCGGTTGGATCGAGCTAAGAGGATCCTCTCTCGGCACGCTG

Aju_MJ1_Clone6 CAGCAAAAAGCGGTTGGATCGAGCTAAGAGGATCCTCTCTCGGCACGCTG

Ale_MJ1_Clone3 CAGCAAAAAGCGGTTGGATCGAGCTAAGAGGATCCTCTCTCGGCACGCTG

Ale_MJ1_Clone12 CAGCAAAAAGCGGTTGGATCGAGCTAAGAGGATCCTCTCTCGGCACGCTG

Aju_MJ1_Clone4 CAGCAAAAAGCGGTTGGATCGAGCTAAGAGGATCCTCTCTCGGCACGCTG

Aju_MJ1_Clone8 CAGCAAAAAGCGGTTGGATCGAGCTAAGAGGATCCTCTCTCGGCACGCTG

Ale_MJ1_Clone9 CAGCAAAAAGCGGTTGGATCGAGCTAAGAGGATCCTCTCTCGGCACGCTG

Ale_MJ1_Clone6 CAGCAAAAAGCGGTTGGATCGAGCTAAGAGGATCCTCTCTCGGCACGCTG

Ale_MJ1_Clone13 CAGCAAAAAGCGGTTGGATCGAGCTAAGAGGATCCTCTCTCGGCACGCTG

Ale_MJ1_Clone2 CAGCAAAAAGCGGTTGGATCGAGCTAAGAGGATCCTCTCTCGGCACGCTG

Ape_MJ1_Clone1 CAGCAAAAAGCGGTTGGATCGAGCTAAGAGGATCCTCTCTCGGCACGCTG

Ape_MJ1_Clone3 CAGCAAAAAGCGGTTGGATCGAGCTAAGAGGATCCTCTCTCGGCACGCTG

CONTIG_574 CAGCAAAAAGCGGTTGGATCGAGCTAAGAGGATCCTCTCTCGGCACGCTG

CONTIG_7401 CAGCAAAAAGCGGTTGGATCGAGCTAAGAGGATCCTCTCTCGGCACGCTG

CONTIG_10225 GT-------------------CAGGAGTTTGTTTTTT-CGGAC-------

CONTIG_29853 GT-------------------CAGGAGTTTGTTTTTT-CGGAC-------

CONTIG_11920 GT-------------------CAGGAGTTTGTTTTTTTCGGAC-------

CONTIG_8991 GT-------------------CAGGAGTTTGTTTTTT-CGGAC-------

CONTIG_23766 GT-------------------CAGGAGTTTGTTTTTT-CGGAC-------

CONTIG_13910 GT-------------------CAGGAGTTTGTTTTTT-CGGAC-------

CONTIG_4960 GT-------------------CAGGAGTTTGTTTTTT-CGGAC-------

Ape_MJ1_Clone6 GT-------------------CAGGAGTTTGTTTTTT-CGGAC-------

Ape_MJ1_Clone7 GT-------------------CAGGAGTTTGTTTTTT-CGGAC-------

Ape_MJ1_Clone2 GT-------------------CAGGAGTTTGTTTTTT-CGAAC-------

Ale_MJ1_Clone1 GT-------------------CAGGAGTTTGTTTTTT-CGGAC-------

Ale_MJ1_Clone11 GT-------------------CAGGAGTTTGTTTTTT-CGGAC-------

Ahy_MJ1_Clone1 GT-------------------CAGGAGTTTGTTTTTT-CGGAC-------

Ahy_MJ1_Clone2 GT-------------------CAGGAGTTTGTTTTTT-CGGAC-------

Ale_MJ1_Clone8 GT-------------------CAGGAGTTTGTTTTTT-CGGACGAGAAAC

Acr_MJ1_Clone2 GT-------------------CAGGAGTTTGTTTTTT-CGGAC-------

Acr_MJ1_Clone3 GT-------------------CAGGAGTTTGTTTTTT-CGGAC-------

Acr_MJ1_Clone1 GT-------------------CAGGAGTTTGTTTTTT-CGTAC-------

Ape_MJ1_Clone4 -T-------------------CAGGAGTTTGATTTTT-CGGAC-------

Ape_MJ1_Clone5 -T-------------------CAGGAGTTTGATTTTT-CGGAC-------

Ahy_MJ1_Clone3 GT-------------------CAGGAGTTTGTTTTTT-CGGAC-------

Ale_MJ1_Clone7 GT-------------------CAGGAGTTTGTTTTTT-CGGAC-------

Aju_MJ1_Clone3 GT-------------------CAGGAGTTTGTTTTTT-CGGAC-------

Aya_MJ1_Clone2 GT-------------------CAGGAGTTTGTTTTTT-CGGAC-------

Asi_MJ1_Clone2 GT-------------------CAGGAGTTTGTTTTTT-CGGAC-------

Asi_MJ1_Clone7 GT-------------------CAGGAGTTTGTTTTTT-CGGAC-------

Asi_MJ1_Clone1 GT-------------------CAGGAGTTTGTTTTTT-CGGAC-------

Asi_MJ1_Clone3 TTTGAAAAACTCAGTTTGTTTCAGGAGTTTGTTTTTT-CGGAC-------

Asi_MJ1_Clone4 TTTGAAAAACTCAGTTTGTTTCAGGAGTTTGTTTTTT-CGGAC-------

Asi_MJ1_Clone6 TTTGAAAAACTCAGTTTGTTTCAGGAGTTTGTTTTTT-CGGAC-------

Akl_MJ1_Clone3 GT-------------------CAGGAGTTTGTTTTTT-CGGAC-------

Aba_MJ1_Clone2 GT-------------------CAGGAGTTTGTTTTTT-CGGAC-------

Akw_MJ1_Clone3 GT-------------------CAGGAGTTTGTTTTTT-CGGAC-------

Aya_MJ1_Clone3 GT-------------------CAGGAGTTTGTTTTTT-CGGAC-------

Aju_MJ1_Clone1 GT-------------------CAGGAGTTTGTTTTTT-CGGAC-------

Asi_MJ1_Clone5 GT-------------------CAGGAGTATGTTTTTT-CGGAC-------

Asi_MJ1_Clone9 GT-------------------CAGGAGTTTGTTTTTT-CGGAC-------

Akw_MJ1_Clone2 GT-------------------CAGGAGTTTGTTTTTT-CGGAC-------

Asi_MJ1_Clone8 GT-------------------CAGGAGTTTGTTTTGT-CGGAC-------

Akl_MJ1_Clone2 GT-------------------CAGGAGTTTGTTTTTT-CGGAC-------

Aya_MJ1_Clone1 GT-------------------CAGGAGTTTGTTTTTT-CGGAC-------

Akl_MJ1_Clone1 GT-------------------CAGGAGTTTGTTTTTT-TGGAC-------

Aba_MJ1_Clone3 GT-------------------CAGGAGTTTGTTTTTT-CGGAC-------

Aba_MJ1_Clone1 GT-------------------CAGGAGTTTGTTTTTT-CGGAC-------

Akw_MJ1_Clone1 GT-------------------CAGGAGTTTGTTTTTT-CGGAC-------

Aju_MJ1_Clone2 GT-------------------CAGGAGTTTGTTTTTT-CGGAC-------

Aju_MJ1_Clone7 GT-------------------CAGGAGTTTGTTTTTT-CGGAC-------

Ale_MJ1_Clone4 GT-------------------CAGGAGTTTGTTTTTT-CGGAC-------

Ale_MJ1_Clone10 GT-------------------CAGGAGTTTGTTTTTT-CGGAC-------

Aju_MJ1_Clone5 GT-------------------CAGGAGTTTGTTTTTT-CGGAC-------

Ale_MJ1_Clone5 GT-------------------CAGGAGTTTGTTTTTT-CGGAC-------

Aju_MJ1_Clone6 GT-------------------CAGCAGTTTGTTTTTT-CGGAC-------

Ale_MJ1_Clone3 GT-------------------CAGGAGTTTGTTTTTT-CGGAC-------

Ale_MJ1_Clone12 GT-------------------CAGGAGTTTGTTTTTT-CGGAC-------

Aju_MJ1_Clone4 GT-------------------CAGGAGTTTGTTTTTT-CGGAC-------

Aju_MJ1_Clone8 GT-------------------CAGGAGTTTGTTTTTT-CGGAC-------

Ale_MJ1_Clone9 GT-------------------CAGGAGTTTGTTTTTT-CGGAC-------

Ale_MJ1_Clone6 GT-------------------CAGGAGTTTGTTTTTT-CGGAC-------

Ale_MJ1_Clone13 GT-------------------CAGGAGTTTGTTTTTT-CGGAC-------

Ale_MJ1_Clone2 GT-------------------CAGGAGTTTGTTTTTT-CGGAC-------

Ape_MJ1_Clone1 GT-------------------CAGGAGTTTGTTTTTT-CGGAC-------

Ape_MJ1_Clone3 GT-------------------CAGGAGTTTGTTTTTT-CGGAC-------

CONTIG_574 AT-------------------CAGGAGTTTGTTTTTT-CGGAC-------

CONTIG_7401 GT-------------------CAGGAGTTTGTTTTTT-CGGAC-------

CONTIG_10225 --------------------------------------------------

CONTIG_29853 --------------------------------------------------

CONTIG_11920 --------------------------------------------------

CONTIG_8991 --------------------------------------------------

CONTIG_23766 --------------------------------------------------

CONTIG_13910 --------------------------------------------------

CONTIG_4960 --------------------------------------------------

Ape_MJ1_Clone6 --------------------------------------------------

Ape_MJ1_Clone7 --------------------------------------------------

Ape_MJ1_Clone2 --------------------------------------------------

Ale_MJ1_Clone1 --------------------------------------------------

Ale_MJ1_Clone11 --------------------------------------------------

Ahy_MJ1_Clone1 --------CTCCA--------------TTCCTTTTCCAT-----------

Ahy_MJ1_Clone2 --------CTCCA--------------TTCCTTTTCCAT-----------

Ale_MJ1_Clone8 TGTTCGTGCTGCAGCAGCCGCACAATGTCCCATCGCCATCGAGGGACAGC

Acr_MJ1_Clone2 --------------------------------------------------

Acr_MJ1_Clone3 --------------------------------------------------

Acr_MJ1_Clone1 --------------------------------------------------

Ape_MJ1_Clone4 --------------------------------------------------

Ape_MJ1_Clone5 --------------------------------------------------

Ahy_MJ1_Clone3 --------------------------------------------------

Ale_MJ1_Clone7 --------------------------------------------------

Aju_MJ1_Clone3 --------------------------------------------------

Aya_MJ1_Clone2 --------------------------------------------------

Asi_MJ1_Clone2 --------------------------------------------------

Asi_MJ1_Clone7 --------------------------------------------------

Asi_MJ1_Clone1 --------------------------------------------------

Asi_MJ1_Clone3 --------------------------------------------------

Asi_MJ1_Clone4 --------------------------------------------------

Asi_MJ1_Clone6 --------------------------------------------------

Akl_MJ1_Clone3 --------------------------------------------------

Aba_MJ1_Clone2 --------------------------------------------------

Akw_MJ1_Clone3 --------------------------------------------------

Aya_MJ1_Clone3 --------------------------------------------------

Aju_MJ1_Clone1 --------------------------------------------------

Asi_MJ1_Clone5 --------------------------------------------------

Asi_MJ1_Clone9 --------------------------------------------------

Akw_MJ1_Clone2 --------------------------------------------------

Asi_MJ1_Clone8 --------------------------------------------------

Akl_MJ1_Clone2 --------------------------------------------------

Aya_MJ1_Clone1 --------------------------------------------------

Akl_MJ1_Clone1 --------------------------------------------------

Aba_MJ1_Clone3 --------------------------------------------------

Aba_MJ1_Clone1 --------------------------------------------------

Akw_MJ1_Clone1 --------------------------------------------------

Aju_MJ1_Clone2 --------------------------------------------------

Aju_MJ1_Clone7 --------------------------------------------------

Ale_MJ1_Clone4 --------------------------------------------------

Ale_MJ1_Clone10 --------------------------------------------------

Aju_MJ1_Clone5 --------------------------------------------------

Ale_MJ1_Clone5 --------------------------------------------------

Aju_MJ1_Clone6 --------------------------------------------------

Ale_MJ1_Clone3 --------------------------------------------------

Ale_MJ1_Clone12 --------------------------------------------------

Aju_MJ1_Clone4 --------------------------------------------------

Aju_MJ1_Clone8 --------------------------------------------------

Ale_MJ1_Clone9 --------------------------------------------------

Ale_MJ1_Clone6 --------------------------------------------------

Ale_MJ1_Clone13 --------------------------------------------------

Ale_MJ1_Clone2 --------------------------------------------------

Ape_MJ1_Clone1 --------------------------------------------------

Ape_MJ1_Clone3 --------------------------------------------------

CONTIG_574 --------------------------------------------------

CONTIG_7401 --------------------------------------------------

CONTIG_10225 ------GAG--------AAACTGTTCGTGCTGCAGCAGCCGCACAATGTG

CONTIG_29853 ------GAG--------AAACTGTTCGTGCTGCAGCAGCCGCACAATGTG

CONTIG_11920 ------GAG--------AAACTGTTCGTGCTGCAGCAGCCGCACAATGTG

CONTIG_8991 ------GAG--------AAACTGTTCGTGCTGCAGCAGCCGCACAATGTG

CONTIG_23766 ------GAG--------AAACTGTTCGTGCTGCAGCAGCCGCACAATGTG

CONTIG_13910 ------GAG--------AAACTGTTCGTGCTGCAGCAGCCGCACAATGTG

CONTIG_4960 ------GAG--------AAACTGTTCGTGCTGCAGCAGCCGCACAATGTG

Ape_MJ1_Clone6 ------GAG--------AAACTGTTCGTGCTGCAGCAGCCGCACAATGTG

Ape_MJ1_Clone7 ------GAG--------AAACTGTTCGTGCTGCAGCAGCCGCACAATGTG

Ape_MJ1_Clone2 ------GAG--------AAACTGTTCGTGCTGCAACAGCCGCACAATGTG

Ale_MJ1_Clone1 ------GAG--------AAACTGTTCGTGCTGCAGCAGCCGCACAATGTG

Ale_MJ1_Clone11 ------GAG--------AAACTGTTCGTGCTGCAGCAGCCGCACAATGTG

Ahy_MJ1_Clone1 --TCCTGAATCCAATATAAAC-----------------------------

Ahy_MJ1_Clone2 --TCCTGAATCCAATATAAAC-----------------------------

Ale_MJ1_Clone8 ATTCCTGAATCCAATATAAAC-----------------------------

Acr_MJ1_Clone2 ------GAG--------AAACTGTTCGTGCTGCAGCAGCCGCACAATGTG

Acr_MJ1_Clone3 ------GAG--------AAACTGTTCGTGCTGCAGCAGCCGCACAATGTG

Acr_MJ1_Clone1 ------GAG--------AAACTGTTCGTGCTGCAGCAGCCGCACAATGTG

Ape_MJ1_Clone4 ------GAG--------AAA------------------------------

Ape_MJ1_Clone5 ------GAG--------AAA------------------------------

Ahy_MJ1_Clone3 ------GAG--------AAACTGTTCGTGCTGCAGCAGCCGCACAATGTG

Ale_MJ1_Clone7 ------GAG--------AAACTGTTCGTGCTGCAGCAGCCGCACAATGTG

Aju_MJ1_Clone3 ------GAG--------AAACTGTTCGTGCTGCAGCAGCCGCACAATGTG

Aya_MJ1_Clone2 ------GAG--------AAACTGTTCGTGCTGCAGCAGCCGCACAATGTG

Asi_MJ1_Clone2 ------GAG--------AAACTGTTCGTGCTGCAGCAGCCGCACAATGTG

Asi_MJ1_Clone7 ------GAG--------AAACTGTTCGTGCTGCAGCAGCCGCACAATGTG

Asi_MJ1_Clone1 ------GAG--------AAACTGTTCGTGCTGCAGCAGCCGCACAATGTG

Asi_MJ1_Clone3 ------GAG--------AAACTGTTCGTGCTGCAGCAGCCGCACAATGTG

Asi_MJ1_Clone4 ------GAG--------AAACTGTTCGTGCTGCAGCAGCCGCACAATGTG

Asi_MJ1_Clone6 ------GAG--------AAACTGTTCGTGCTGCAGCAGCCGCACAATGTG

Akl_MJ1_Clone3 ------GAG--------AAACTGTTCGTGCTGCAGCAGCCGCACAATGTG

Aba_MJ1_Clone2 ------GAG--------AAACTGTTCGTGCTGCAGCAGCCGCACAATGTG

Akw_MJ1_Clone3 ------GAG--------AAACTGTTCGTGCTGCAGCAGCCGCACAATGTG

Aya_MJ1_Clone3 ------GAG--------AAACTGTTCGTGCTGCAGCAGCCGCACAATGTG

Aju_MJ1_Clone1 ------GAG--------AAACTGTTCGTGCTGCAGCAGCCGCACAATGTG

Asi_MJ1_Clone5 ------GAG--------AAACTGTTCGTGCTGCAGCAGCCGCACAATGTG

Asi_MJ1_Clone9 ------GAG--------AAACTGTTCGTGCTGCAGCAGCCGCACAATGTG

Akw_MJ1_Clone2 ------GAG--------AAACTGTTCGTGCTGCAGCAGCCGCACAATGTG

Asi_MJ1_Clone8 ------GAG--------AAACTGTTCGTGCTGCAGCAGCCGCACAATGTG

Akl_MJ1_Clone2 ------GAG--------AAACTGTTCGTGCTGCAGCAGCCGCACAATGTG

Aya_MJ1_Clone1 ------GAG--------AAACTGTTCGTGCTGCAGCAGCCGCACAATGTG

Akl_MJ1_Clone1 ------GAG--------AAACTGTTCGTGCTGCAGCAGCCGCACAATGTG

Aba_MJ1_Clone3 ------GAG--------AAACTGTTCGTGCTACAGCAGCCGCACAATGTG

Aba_MJ1_Clone1 ------GAG--------AAACTGTTCGTGCTGCAGCAGCCGCACAATGTG

Akw_MJ1_Clone1 ------GAG--------AAACTGTTCGTGCTGCAGCAGCCGCACAATGTG

Aju_MJ1_Clone2 ------GAG--------AAACTGTTCGTGCTGCAGCAGCCGCACAATGTG

Aju_MJ1_Clone7 ------GAG--------AAACTGTTCGTGCTGCAGCAGCCGCACAATGTG

Ale_MJ1_Clone4 ------GAG--------AAACTGTTCGTGCTGCAGCAGCCGCACAATGTG

Ale_MJ1_Clone10 ------GAG--------AAACTGTTCGTGCTGCAGCAGCCGCACAATGTG

Aju_MJ1_Clone5 ------GAG--------AAACTGTTCGTGCTGCAGCAGCCGCACAATGTG

Ale_MJ1_Clone5 ------GAG--------AAACTGTTCGTGCTGCAGCAGCCGCACAATGTG

Aju_MJ1_Clone6 ------GAG--------AAACTGTTCGTGCTGCAGCAGCCGCACAATGTG

Ale_MJ1_Clone3 ------GAG--------AAACTGTTCGTGCTGCAGCAGCCGCACAATGTG

Ale_MJ1_Clone12 ------GAG--------AAACTGTTCGTGCTGCAGCAGCCGCACAATGTG

Aju_MJ1_Clone4 ------GAG--------AAACTGTTCGTGCTGCAGCAGCCGCACAATGTG

Aju_MJ1_Clone8 ------GAG--------AAACTGTTCGTGCTGCAGCAGCCGCACAATGTG

Ale_MJ1_Clone9 ------GAG--------AAACTGTTCGTGCTGCAGCAGCCGCACAATGTG

Ale_MJ1_Clone6 ------GAG--------AAACTGTTCGTGCTGCAGCAGCCGCACAATGTG

Ale_MJ1_Clone13 ------GAG--------AAACTGTTCGTGCTGCAGCAGCCGCACAATGTG

Ale_MJ1_Clone2 ------GAG--------AAACTGTTCGTGCTGCAGCAGCCGCACAATGTG

Ape_MJ1_Clone1 ------GAG--------AAACTGTTCGTGCTGCAGCAGCCGCACAATGTG

Ape_MJ1_Clone3 ------GAG--------AAACTGTTCGTGCTGCAGCAGCCGCACAATGTG

CONTIG_574 ------GAG--------AAACTGTTCGTGCTGCAGCAGCCGCACAATGTA

CONTIG_7401 ------GAG--------AAACTGTTCGTGCTGCAGCAGCCGCACAATGTG

CONTIG_10225 CAAAATGACCGGGTGTGGGCGCCATCGAGGGACAGCATTCCTGAATCCAA

CONTIG_29853 CAAAATGACCGGGTGTGGGCGCCATCGAGGGACAGCATTCCTGAATCCAA

CONTIG_11920 CAAAATGACCGGGTGTGGGCGCCATCGAGGGACAGCATTCCTGAATCCAA

CONTIG_8991 CAAAATGACCGGGTGTGGGCGCCATCGAGGGACAGCATTCCTGAATCCAA

CONTIG_23766 CAAAATGACCGGGTGTGGGCGCCATCGAGGGACAGCATTCCTGAATCCAA

CONTIG_13910 CAAAATGACCGGGTGTGGGCGCCATCGAGGGACAGCATTCCTGAATCCAA

CONTIG_4960 CAAAATGACCGGGTGTGGGCGCCATCGAGGGACAGCATTCCTGAATCCAA

Ape_MJ1_Clone6 CAAAATGACCGGGTGTGGGCGCCATCGAGGGACAGCATTCCTGAATCCAA

Ape_MJ1_Clone7 CAAAATGACCGGGTGTGGGCGCCATCGAGGGACAGCATTCCTGAATCCAA

Ape_MJ1_Clone2 CAAAATGACCGGGTGTGGGCGCCATCGAGGGACAGCATTCCTGAATCCAA

Ale_MJ1_Clone1 CAAAATGACCGGGTGTGGGCGCCATCGAGGGACAGCATTCCTGAATCCAA

Ale_MJ1_Clone11 CAAAATGACCGGGTGTGGGCGCCATCGAGGGACAGCATTCCTGAATCCAA

Ahy_MJ1_Clone1 --------------------------------------------------

Ahy_MJ1_Clone2 --------------------------------------------------

Ale_MJ1_Clone8 --------------------------------------------------

Acr_MJ1_Clone2 CAAAATGACCGGGTGTGGGCGCCATCGAGGGACAGCATTCCTGAATCCAA

Acr_MJ1_Clone3 CAAAATGACCGGGTGTGGGCGCCATCGAGGGACAGCATTCCTGAATCCAA

Acr_MJ1_Clone1 CAAAATGACCGGGTGTGGGCCCCATCGAGGGACAGCATTCCTGAATCCAA

Ape_MJ1_Clone4 --------------------------------------------------

Ape_MJ1_Clone5 --------------------------------------------------

Ahy_MJ1_Clone3 CAAAATGACCGGGTGTGGGCGCCATCGAGGGACAGCATTCCTGAATCCAA

Ale_MJ1_Clone7 CAAAATGACCGGGTGTGGGCGCCATCGAGGGACAGCATTCCTGAATCCAA

Aju_MJ1_Clone3 CAAAATGACCGGGTGTGGGCGCCATCGAGGGACAGCATTCCTGAATCCAA

Aya_MJ1_Clone2 CAAAATGACCGGGTGTGGGCGCCATCGAGGGACAGCATTCCTGAATCCAA

Asi_MJ1_Clone2 CGAAATGACCGGGTGTGGGCGCCATCGAGGGACAGCATTCCTGAATCCAA

Asi_MJ1_Clone7 CAAAATGACCGGGTGTGGGCGCCATCGAGGGACAGCATTCCTGAATCCAA

Asi_MJ1_Clone1 CAAAATGACCGGGTGTGGGCGCCATCGAGGGACAGCATTCCTGAATCCAA

Asi_MJ1_Clone3 CAAAATGACCGGGTGTGGGCGCCATCGAGGGACAGCATTCCTGAATCCAA

Asi_MJ1_Clone4 CAAAATGACCGGGTGTGGGCGCCATCGAGGGACAGCATTCCTGAATCCAA

Asi_MJ1_Clone6 CAAAATGACCGGGTGTGGGCGCCATCGAGGGACAGCATTCCTGAATCCAA

Akl_MJ1_Clone3 CAAAATGACCGGGTGTGGGCGCCATCGAGGGACAGCATTCCTGAATCCAA

Aba_MJ1_Clone2 CAAAATGACCGGGTGTGGGCGCCATCGAGGGACAGCATTCCTGAATCCAA

Akw_MJ1_Clone3 CAAAATGACCGGGTGTGGGCGCCATCGAGGGACAGCATTCCTGAATCCAA

Aya_MJ1_Clone3 CAAAATGACCGGGTGTGGGCGCCATCGAGGGACAGCATTCCTGAATCCAA

Aju_MJ1_Clone1 CAAAATGACCGGGTGTGGGCGCCATCGAGGGACAGCATTCCTGAATCCAA

Asi_MJ1_Clone5 CAAAATGACCGGGTGTGGGCGCCATCGAGGGACAGCATTCCTGAATCCAA

Asi_MJ1_Clone9 CAAAATGACCGGGTGTGGGCGCCATCGAGGGACAGCATTCCTGAATCCAA

Akw_MJ1_Clone2 CAAAATGACCGGGTGTGGGCGCCATCGAGGGACAGCATTCCTGAATCCAA

Asi_MJ1_Clone8 CAAAATGACCGGGTGTGGGCGCCATCGAGGGACAGCATTCCTGAATCCAA

Akl_MJ1_Clone2 CAAAA---CCGGGTGTGGGCGCCATCGAGGGACAGCATTCCTGAATCCAA

Aya_MJ1_Clone1 CAAAATGACCGGGTGTGGGCGCCATCGAGGGACAGCATTCCTGAATCCAA

Akl_MJ1_Clone1 CAAAATGACCGGGTGTGGGCGCCATCGAGGGACAGCATTCCTGAATCCAA

Aba_MJ1_Clone3 CAAAATGACCGGGTGTGGGCGCCATCGAGGGACAGCATTCCTAAATCCAA

Aba_MJ1_Clone1 CAAAATGACCGGGTGTGGGCGCCATCGAGGGACAGCATTCCTGAATCCAA

Akw_MJ1_Clone1 CAAAATGACCGGATGTGGGCGCCATCGAGGGACAGCATTCCTGAATCCAA

Aju_MJ1_Clone2 CAAAATG-------------------------------------------

Aju_MJ1_Clone7 CAAAATGACCGGGTGTGGGCGCCATCGAGGGACAGCATTCCTGAATCCAA

Ale_MJ1_Clone4 CAAAATGACCGGGTGTGGGCGCCATCGAGGGACAGCATTCCTGAATCCAA

Ale_MJ1_Clone10 CAAAATGACCGGGTGTGGGCGCCATCGAGGGACAGCATTCCTGAATCCAA

Aju_MJ1_Clone5 CAAAATGACCGGGTGTGGGCGCCATCGAGGGACAGCATTCCTGAATCCAA

Ale_MJ1_Clone5 CAAAATGACCGGGTGTGGGCGCCATCGAGGGACAGCATTCCTGAATCCAA

Aju_MJ1_Clone6 CAAAATG-------------------------------------------

Ale_MJ1_Clone3 CAAAATGACCGGGTGTGGGCGCCATCGAGGGACAGCATTCCTGAATCCAA

Ale_MJ1_Clone12 CAAAATGACCGGGTGTGGGCGCCATCGAGGGACAGCATTCCTGAATCCAA

Aju_MJ1_Clone4 CAAAATGACCGGGTGTGGGCGCCATCGAGGGACAGCATTCCTGAATCCAA

Aju_MJ1_Clone8 CAAAATGACCGGGTGTGGGCGCCATCGAGGGACAGCATTCCTGAATCCAA

Ale_MJ1_Clone9 CAAAATGACCGGGTGTGGGCGCCATCGAGGGACAGCATTCCTGAATCCAA

Ale_MJ1_Clone6 CAAAATGACCGGGTGTGGGCGCCATCGAGGGACAGCATTCCTGAATCCAA

Ale_MJ1_Clone13 CAAAATGACCGGGTGTGGGCGCCATCGAGGGACAGCATTCCTGAATCCAA

Ale_MJ1_Clone2 CAAAATGACCGGGTGTGGGCGCCATCGAGGGACAGCATTCCTGAATCCAA

Ape_MJ1_Clone1 CAAAATGACCGGGTGTGGGCGCCATCGAGGGACAGCATTCCTGAATCCAA

Ape_MJ1_Clone3 CAAAATGACCGGGTGTGGGCGCCATCGAGGGACAGCATTCCTGAATCCAA

CONTIG_574 CAAAATGACCGGGTGTGGGCGCCATCGAGGGACAGCATTCCTGAATCCAA

CONTIG_7401 CAAAATGACCGGGTGTGGGCGCCATCGAGGGACAGCATTCCTGAATCCAA

CONTIG_10225 TATAAACATCCCTTGGTTCCAAAGTGCCGCGTCGGTGATGGTTTGGGGGG

CONTIG_29853 TATAAACATCCCTCGGTTCCAAAGTGCCGCGTCGGTGATGGTTTGGGGGG

CONTIG_11920 TATAAACATCCCTCGGTTCCAAAGTGCCGCGTCGGTGATGGTTTGGGGGG

CONTIG_8991 TATAAACATCCCTCGGTTCCAAAGTGCCGCGTCGGTGATGGTTTGGGGGG

CONTIG_23766 TATAAACATCCCTCGGTTCCAAAGTGCCGCGTCGGTGATGGTTTGGGGGG

CONTIG_13910 TATAAACATCCCTCGGTTCCAAAGTGCCGCGTCGGTGATGGTTTGGGGGG

CONTIG_4960 TATAAACATCCCTCGGTTCCAAAGTGCCGCGTCGGTGATGGTTTGGGGGG

Ape_MJ1_Clone6 TATAAACATCCCTCGGTTCCAAAGTGCCGCGTCGGTGATGGTTTGGGGGG

Ape_MJ1_Clone7 TATAAACATCCCTCGGTTCCAAAGTGCCGCGTCGGTGATGGTTTGGGGGG

Ape_MJ1_Clone2 TATAAACATCCCTCGGTTCCAAAGTGCCGCGTCGGTGATGGTTTGGGGGG

Ale_MJ1_Clone1 TATAAACATCCCTCGGTTCCAAAGTGCCGCGTCGGTGATGGTTTGGGGGG

Ale_MJ1_Clone11 TATAAACATCCCTCGGTTCCAAAGTGCCGCGTCGGTGATGGTTTGGGGGG

Ahy_MJ1_Clone1 -------ATCCCTCGGTTCCAAAGTGCCGCGTCGGTGATGGTTTGGGGGG

Ahy_MJ1_Clone2 -------ATCCCTCGGTTCCAAAGTGCCGCGTCGGTGATGGTTTGGGGGG

Ale_MJ1_Clone8 -------ATCCCTCGGTTCCAAAGTGCCGCGTCGGTGATGGTTTGGGGGG

Acr_MJ1_Clone2 TATAAACATCCCTCGGTTCCAAAGTGCCGCGTCGGTGGTGGTTTGGGGGG

Acr_MJ1_Clone3 TATAAACATCCCTCGGTTCCAAAGTGCCGCGTCGGTGGTGGTTTGGGGGG

Acr_MJ1_Clone1 TATAAACATCCCTCGGTTCCAAAGTGCCGCGTCGGTGGTGGTTTGGGGGG

Ape_MJ1_Clone4 --------------------------------------------------

Ape_MJ1_Clone5 --------------------------------------------------

Ahy_MJ1_Clone3 TATAAACATCCCTCGGTTCCAAAGTGCCGCGTCGGTGATGGTTTGGGGGG

Ale_MJ1_Clone7 TATAAACATCCCTCGGTTCCAAAGTGCCGCGTCGGTGATGGTTTGGGGGG

Aju_MJ1_Clone3 TATAAACATCCCTCGGTTCCAAAGTGCCGCGTCGGTGATGGTTTGGGGGG

Aya_MJ1_Clone2 TATAAACATCCCTCGGTTCCAAAGTGCCGCGTCGGTGATGGTTTGGGGGG

Asi_MJ1_Clone2 TATAAACATCCCTCGGTTCCAAAGTGCCGCGTCGGTGATGGTTTGGGGGG

Asi_MJ1_Clone7 TATAAACATCCCTCGGTTCCAAAGTGCCGCGTCGGTGATGGTTTGGGGGG

Asi_MJ1_Clone1 TATAAACATCCCTCGGTTCCAAAGTGCCGCGTCGGTGATGGTTTGGGGGG

Asi_MJ1_Clone3 TATAAACATCCCTCGGTTCCAAAGTGCCACGTCGGTGATGGTTTGGGGGG

Asi_MJ1_Clone4 TATAAACATCCCTCGGTTCCAAAGTGCCACGTCGGTGATGGTTTGGGGGG

Asi_MJ1_Clone6 TATAAACATCCCTCGGTTCCAAAGTGCCACGTCGGTGATGGTTTGGGGGG

Akl_MJ1_Clone3 TATAAACATCCCTCGGTTCCAAAGTGCCGCGTCGGTGATGGTTTGGGGGG

Aba_MJ1_Clone2 TATAAACATCCCTCGGTTCCAAAGTGCCGCGTCGGTGATGGTTTGGGGGG

Akw_MJ1_Clone3 TATAAACATCCCTCGGTTCCAAAGTGCCGCGTCGGTGATGGTTTGGGGGG

Aya_MJ1_Clone3 TATAAACATCCCTCGGTTCCAAAGTGCCGCGTCGGTGATGGTTTGGGGGG

Aju_MJ1_Clone1 TATAAACATCCCTCGGTTCCAAAGTGCCGCGTCGGTGATGGTTTGGGGGG

Asi_MJ1_Clone5 TATAAACATCCCTCGGTTCCAAAGTGCCGCGTCGGTGATGGTTTGGGGGG

Asi_MJ1_Clone9 TATAAACATCCCTCGGTTCCAAAGTGCCGCGTCGGTGATGGTTTGGGGGG

Akw_MJ1_Clone2 TATAAACATCCCTCGGTTCCAAAGTGCCGCGTCGGTGATGGTTTGGGGGG

Asi_MJ1_Clone8 TATAAACATCCCTCGGTTCCAAAGTGCCGCGTCGGTGATGGTTTGGGGGG

Akl_MJ1_Clone2 TATAAACATCCCTCGGTTCCAAAGTGCCGCGTCGGTGATGGTTTGGGGGG

Aya_MJ1_Clone1 TATAAACATCCCTCGGTTCCAAAGTGCCGCGTCGGTGATGGTTTGGGGGG

Akl_MJ1_Clone1 TATAAACATCCCTCGGTTCCAAAGTGCCGCGTCGGTGATGGTTTGGGGGG

Aba_MJ1_Clone3 TATAAACATCCCTCGGTTCCAAAGTGCCGCGTCGGTGATGGTTTGGGGGG

Aba_MJ1_Clone1 TATAAACATCCCTCGGTTCCAAAGTGCCGCGTCGGTGATGGTTTGGGGGG

Akw_MJ1_Clone1 TATAAACATCCCTCGGTTCCAAAGTGCCGCGTCGGTGATGGTTTGGGGGG

Aju_MJ1_Clone2 -----------------------------CGTCGGTGATGGTTTGGGGGG

Aju_MJ1_Clone7 TATAAACATCCCTCGGTTCCAAAGTGCCGCGTCGGTGATGGTTTGGGGGG

Ale_MJ1_Clone4 TATAAACATCCCTCGGTTCCAAAGTGCCGCGTCGGTGATGGTTTGGGGGG

Ale_MJ1_Clone10 TATAAACATCCCTCGGTTCCAAAGTGCCGCGTCGGTGATGGTTTAGGGGG

Aju_MJ1_Clone5 TATAAACATCCCTCGGTTCCAAAGTGCCGCGTCGGTGATGGTTTGGGGGG

Ale_MJ1_Clone5 TATAAACATCCCTCGGTTCCAAAGTGCCGCGTCGGTGATGGTTTGGGGGG

Aju_MJ1_Clone6 -----------------------------CGTCGGTGATGGTTTGGGGGG

Ale_MJ1_Clone3 TATAAACATCCCTCGGTTCCAAAGTGCCGCGTCGGTGATGGTTTGGGGGG

Ale_MJ1_Clone12 TATAAACATCCCTCGGTTCCAAAGTGCCGCGTCGGTGATGGTTTGGGGGG

Aju_MJ1_Clone4 TATAAACATCCCTCGGTTCCAAAGTGCCGCGTCGGTGATGGTTTGGGGGG

Aju_MJ1_Clone8 TATAAACATCCCTCGGTTCCAAAGTGCCGCGTCGGTGATGGTTTGGGGGG

Ale_MJ1_Clone9 TATAAACATCCCTCGGTTCCAAAGTGCCGCGTCGGTGATGGTTTGGGGGG

Ale_MJ1_Clone6 TATAAACATCCCTCGGTTCCAAAGTGCCGCGTCGGTGATGGTTTGGGGGG

Ale_MJ1_Clone13 TATAAACATCCCTCGGTTCCAAAGTGCCGCGTCGGTGATGGTTTGGGGGG

Ale_MJ1_Clone2 TATAAACATCCCTCGGTTCCAAAGTGCCGCGTCGGTGATGGTTTGGGGGG

Ape_MJ1_Clone1 TATAAACATCCCTCGGTTCCAAAGTGCCGCGTCGGTGATGGTTTGGGGGG

Ape_MJ1_Clone3 TATAAACATCCCTCGGTTCCAAAGTGCCGCGTCGGTGATGGTTTGGGGGG

CONTIG_574 TATAAACATCCCTCGGTTCCAAAGTGCCGCGTCAGTGATGGTTTGGGGGG

CONTIG_7401 TATAAACATCCCTCGGTTCCAAAGTGCCGCGTCGGTGATGGTTTGGGGGG

CONTIG_10225 -CAGTATGCAAACGTGGTAAGCTACCCTTGGTGTTTATTGAA-AAAAAA-

CONTIG_29853 -CAGTATGCAAACGTGGTAAGCTACCCTTGGTGTTTATTGAA-AAAAA--

CONTIG_11920 -CAGTATGCAAACGTGGTAAGCTACCCTTGGTGTTTATTGAA-AAAA---

CONTIG_8991 -CAGTATGCAAACGTGGTAAGCTACCCTTGGTGTTTATTGAA-AAAAA--

CONTIG_23766 -CAGTATGCAAACGTGGTAAGCTACCCTTGGTGTTTATTGAA-AAAAAAA

CONTIG_13910 -CAGTATGCAAACGTGGTAAGCTACCCTTGGTGTTTATTGAA-AAAAA--

CONTIG_4960 -CAGTATGCAAACGTGGTAAGCTACCCTTGGTGTTTATTGAA-AAAAA--

Ape_MJ1_Clone6 -CAGTATGCAAACGTGGAAAGCTACCCTTGGTGTTTATTGAA-AAAAA--

Ape_MJ1_Clone7 -CAGTATGCAAACGTGGAAAGCTACCCTTGGTGTTTATTGAG-AAAAA--

Ape_MJ1_Clone2 -CAGTATGCAAACGTGGAAAGCTACCCTTGGTGTTTATTGAA-AAAAA--

Ale_MJ1_Clone1 -CAGTATGCAAACGTGGTAAGCTACCCTTGGTGTTTATTGAA--AAAAA-

Ale_MJ1_Clone11 -CAGTATGCAAACGTGGTAAGCTACCCTTGGTGTTTATTGGA--AAAAA-

Ahy_MJ1_Clone1 -CAGTATGCAAACGTGGTAAGCTACCCTTGGTGTTTATTGAA--AAAAA-

Ahy_MJ1_Clone2 -CAGTATGCAAACGTGGTAAGCTACCCTTGGTGTTTATTGAA--AAAAA-

Ale_MJ1_Clone8 -CAGTATGCAAACGTGGTAAGCTACCCTTGGTGTTTATTGAA--AAAAA-

Acr_MJ1_Clone2 -CAGTATGCAAACGTGGTAAGCTACCCTTGGTGTTTATTGAA--AAAAA-

Acr_MJ1_Clone3 -CAGTATGCAAACGTGGTAAGCTACCCTTGGTGTTTATTGAA--AAAAA-

Acr_MJ1_Clone1 -CAGTATGCAAACGTGGTAAGCTACCCTTGGTGTTTATTGAA--AAAAA-

Ape_MJ1_Clone4 --------------------GCTACCCTTGGTGTTTATTGAAAAAAAAA-

Ape_MJ1_Clone5 --------------------GCTACCCTTGGTGTTTATTGAAGAAAAAA-

Ahy_MJ1_Clone3 -CAGTATGCAAACGTGGTAAGCTACCCTTGGTGTTTATTGAA--AAAAA-

Ale_MJ1_Clone7 -CAGTACGCAAACGTGGTAAGCTACCCTTGGTGTTTATTGAA--AAAAA-

Aju_MJ1_Clone3 -CAGTATGCAAACGTGGTAAGCTACCCTTGGTGTTTATTGAA--AAAAA-

Aya_MJ1_Clone2 -CAGTATGCAAACGTGGTAAGCTACCCTTGGTGTTTATTGAA--AAAAA-

Asi_MJ1_Clone2 -TAGTATGCAAACGTGGTAAGCTACCCTTGGTGTTTATTGAA-AAAAA--

Asi_MJ1_Clone7 -TAGTATGCAAACGTGGTAAGCTACCCTTGGTGTTTATTGAA-AAAAA--

Asi_MJ1_Clone1 -TAGTATGCAAACGTGGTAAGCTACCCTTGGTGTTTATTGAA-AAAAA--

Asi_MJ1_Clone3 -CAGTATGCAAACGTGGTAAGCTACCCTTGGTGTTTATTGAA-AAAAA--

Asi_MJ1_Clone4 -CAGTATGCAAACGTGGTAAGCTACCCTTGGTGTTTATTGAA-AAAAA--

Asi_MJ1_Clone6 -CAGTATGCAAACGTGGTAAGCTACCCTTGGTGTTTATTGAA-AAAAA--

Akl_MJ1_Clone3 GCAGTATGCAAACGTGGTAAGCTACCCTTGGTATTTATTGAA-AAAAA--

Aba_MJ1_Clone2 -CAGTATGCAAACGTGGTAAGCTACCCTTGGTGTTTATTGAA-AAAAA--

Akw_MJ1_Clone3 -CAGTATGCAAACGTGGTAAGCTACCCTTGGTGTTTATTGAA-AAAAA--

Aya_MJ1_Clone3 -CAGTATGCAAACGTGGTAAGCTACCCTTGGTGTTTATTGAA-AAAAA--

Aju_MJ1_Clone1 -CAGTATGCAAACGTGGTAAGCTACCCTTGGTGTTTATTGAA-AAAAA--

Asi_MJ1_Clone5 -CAGTATGCAAACGTGGTAAGCTACCCTTCGTGTTTATTGAA-AAAAA--

Asi_MJ1_Clone9 -CAGTATGCAAACGTGGTAAGCTACCCTTGGTGTTTATTGAA-AAAAA--

Akw_MJ1_Clone2 -CAGTATGCAAACGTGGTAAGCTACCCTTGGTGTTTATTGAA-AAAAA--

Asi_MJ1_Clone8 -CAGTATGCAAACGTGGTAAGCTACCCTTGGTGTTTATTGAA-AAAAA--

Akl_MJ1_Clone2 -CAGTATGCAAACGTGGTAAGCTACCCTTGGTGTTTATTGAA-AAAAA--

Aya_MJ1_Clone1 -CAGTATGCAAACGTGGTAAGCTACCCTTGGTGTTTATTGAA-AAAAA--

Akl_MJ1_Clone1 GCAGTATGCAAACGTGGTAAGCTACCCTTGGTGTTTATTGAA-AAAAA--

Aba_MJ1_Clone3 GCAGTATGCAAACGTGGTAAGCTACCCTTGGTGTTTATTGAA-AAAAA--

Aba_MJ1_Clone1 -CAGTATGCATACGTGGTAAGCTACCCTTGGTGTTTATTGAA-AAAAA--

Akw_MJ1_Clone1 -CAGTATGCAAACGTGGTAAGCTACCCTTGGTGTTTATTGAA-AAAAA--

Aju_MJ1_Clone2 -CAGTATGCAAACGTGGTAAGCTACCCTTGGTGTTTATTGAA-AAAAA--

Aju_MJ1_Clone7 -CAGTATGCAAACGTGGTAAGCTACCCTTGGTGTTTATTGAA-AAAAA--

Ale_MJ1_Clone4 -CAGTATGCAAACGTGGTAAGCTACCCTTGGTGTTTATTGAA-AAAAAA-

Ale_MJ1_Clone10 -CAGTATGCAAACGTGGTAAGCTACCCTTGGTGTTTATTGAA-AAAAA--

Aju_MJ1_Clone5 -CAGTATGCAAACGTGGTAAGCTACCCTTGGTGTTTATTGAA-AAAAA--

Ale_MJ1_Clone5 -CAGTATGCAAACGTGGTAAGCTACCCTTGGTGTTTATTGAA-AAAAA--

Aju_MJ1_Clone6 -CAGTATGCAAACGTGGTAAGCTACCCTTGGTGTTTATTGAA-AAAAA--

Ale_MJ1_Clone3 -CAGTATGCAAACGTGGTAAGCTACCCTTGGTGTTTATTGAA-AAAAA--

Ale_MJ1_Clone12 -CAGTATGCAAACGTGGTAAGCTACCCTTGGTGTTTATTGAA-AAAAA--

Aju_MJ1_Clone4 -CAGTATGCAAACGTGGTAAGCTACCCTTGGTGTTTATTGAA-AAAAA--

Aju_MJ1_Clone8 -CAGTATGCAAACGTGGTAAGCTACCCTTGGTGTTTATTGAA-AAAAA--

Ale_MJ1_Clone9 -CAGTATGCAAACGTGGTAAGCTACCCTTGGTGTTTATTGAA--AAAAA-

Ale_MJ1_Clone6 -CAGTATGCAAACGTGGTAAGCTACCCTTGGTGTTTATTGAA--AAAAA-

Ale_MJ1_Clone13 -CAGTATGCAAACGTGGTAAGCTACCCTTGGTGTTTATTGAA--AAAAA-

Ale_MJ1_Clone2 -CAGTATGCAAACGTGGTAAGCTACCCTTGGTGTTTATTGAA--AAAAA-

Ape_MJ1_Clone1 -CAGTATGCAAACGTGGTAAGCTACCCTTGGTGTTTATTGAA--AAAAA-

Ape_MJ1_Clone3 -CAGTATGCAAACGTGGTAAGCTACCCTTGGTGTTTATTGAA--AAAAA-

CONTIG_574 -CAGTATGCAAACGTGGTAAGCTACCCTTGGTGTTTATTGAA-AAAAA--

CONTIG_7401 -CAGTATGCAAACGTGGTAAGCTACCCTTGGTGTTTATTGAA-AAAAA--

CONTIG_10225 CGTCAAAATCAACGCGGCGTACAACAAAA-CTGAGGTTTTGGAAAAGGTT

CONTIG_29853 CGTCAAAATCAACGTGGCGTACTACAAAA-CTGAGGTTTTGGAAAAGGTT

CONTIG_11920 CGTCAAAATCAACGCGGCGTACTACAAAA-CTGAGGTTTTGGAAAAGGTT

CONTIG_8991 CGTCAAAATCAACGCGGCGTACTACAAAA-CTCAGGTTTTGGAAAAGGTT

CONTIG_23766 CGTCAAAATCAACGCGGCGTACTACAAAA-CTGAGGTTTTGGAAAAGGTT

CONTIG_13910 CGTCAAAATCAACGCGGCGTACTACAAAA-CTGAGGTTTTGGAAAAGGTT

CONTIG_4960 CGTCAAAATCAACGCGGCGTACTACAAAA-CTGAGGTTTTGGAAAAGGTT

Ape_MJ1_Clone6 CGTCAAAATCAACGCGGCGTACTACAAAA-CTGAGGTTTTGGAAAAGGTT

Ape_MJ1_Clone7 CGTCAAAATCAACGCGGCGTACTACAAAA-CTGAGGTTTTGGAAAAGGTT

Ape_MJ1_Clone2 CGTCAAAATCAACGCGGCGTACTACAAAA-CTGAGGTTTTGGAAAAGGTT

Ale_MJ1_Clone1 CGTCAAAATCAACGCGGCGTACTACAAAA-CTGAGGTTTTGGAAAAGGTT

Ale_MJ1_Clone11 CGTCAAAATCAACGCGGCGTACTACAAAA-CTGAGGTTTTGGAAAAGGTT

Ahy_MJ1_Clone1 CGTCAAAATCAACGCGGCGTACTACAAAA-CTGAGGTTTTGGAAAAGGTT

Ahy_MJ1_Clone2 CGTCAAAATCAACGCGGCGTACTACAAAA-CTGAGGTTTTGGAAAAGGTT

Ale_MJ1_Clone8 CGTCAAAATCAACGCGGCGTACTACAAAA-CTGAGGTTTTGGAAAAGGTT

Acr_MJ1_Clone2 CGTCAAAATCAAAGCGGCGTACTACAAAA-CTGAGGTTTTGGAAAAGGTT

Acr_MJ1_Clone3 CGTCAAAATCAAAGCGGCGTACTACAAAA-CTGAGGTTTTGGAAAAGGTT

Acr_MJ1_Clone1 CGTCAAAATCAACGCGGCGTACTACAAAA-CTGAGGTTTTGGAAAAGGTT

Ape_MJ1_Clone4 CGCCAAAATCAACGCGGCGTACTACAAAAACTGAGGTT------------

Ape_MJ1_Clone5 CGCCAAAATCAACGCGGCGTACTACAAAAACTGAGGTT------------

Ahy_MJ1_Clone3 CGTCAAAATCAACGCGGCGTACTACAAAA-CTGAGGTTTTGGAAAAGGTT

Ale_MJ1_Clone7 CGTCAAAATCAACGCGGCGTACTACAAAA-CTGAGGTTTTGGAAAAGGTT

Aju_MJ1_Clone3 CGTCAAAATCAACGCGGCGTACTACAAAA-CTGAGGTTTTGGAAAAGGTT

Aya_MJ1_Clone2 CGTCAAAATCAACGCGGCGTACTACAAAA-CTGAGGTTTTGGAAAAGGTT

Asi_MJ1_Clone2 CGTCAAAATCAACGCGGCGTACTACAAAA-CTGAGGTTTTGGAAAAGGTT

Asi_MJ1_Clone7 CGTCAAAATCAACGCGGCGTACTACAAAA-CTGAGGTTTTGGAAAAGGTT

Asi_MJ1_Clone1 CGTCAAAATCAACGCGGCGTACTACAAAA-CTGAGGTTTTGGAAAAGGTT

Asi_MJ1_Clone3 CGTCAAAATCAACGCGGCGTACTACAAAA-CTGAGGTTTTGGAAAAGGTT

Asi_MJ1_Clone4 CGTCAAAATCAACGCGGCGTACTACAAAA-CTGAGGTTTTGGAAAAGGTT

Asi_MJ1_Clone6 CGTCAAAATCAACGCGGCGTACTACAAAA-CTGAGGTTTTGGAAAAGGTT

Akl_MJ1_Clone3 CGTCAAAATCAACGCGGCGTACTACGAAA-CTGAGGTTTTGGAAAAGGTT

Aba_MJ1_Clone2 CGTCAAA-------------------------------------------

Akw_MJ1_Clone3 CGTCAAAATCAACGCGGCGTACTACAAAA-CTGAGGTTTTGGAAAAGGTT

Aya_MJ1_Clone3 CGTCAAAATCAACGCGGCGTACTACAAAA-CTGAGGTTTTGGAAAAGGTT

Aju_MJ1_Clone1 CGTCAAAATCAACGCGGCGTACTACAAAA-CTGAGGTTTTGGAAAAGGTT

Asi_MJ1_Clone5 CGTCAAAATCAACGCGGCGTACTACAAAA-CTGAGGTTTTGGAAAAGGTT

Asi_MJ1_Clone9 CGTCAAAATCAACGCGGCGTACTACAAAA-CTGAGGTTTTGGAAAAGGTT

Akw_MJ1_Clone2 CGTCAAAATCAACGCGGCGTACTACAAAA-CTGAGGTTTTGGAAAAGGTT

Asi_MJ1_Clone8 CGTCAAAATCAACGCGGCGTACTACAAAA-CTGAGGTTTTGGAAAAGGTT

Akl_MJ1_Clone2 CGTCAAAATCAACGCGGCGTACTACAAAA-CTGAGGCTTTGGAAAAGGTT

Aya_MJ1_Clone1 CGTCAAAATCAACGCGGCGTACTACAAAA-CTGAGGTTTTGGAAAAGGTT

Akl_MJ1_Clone1 CGTCAAAATCAACGCGGCGTACTACAAAA-CTGAGGTTTTGGAAAAGGTT

Aba_MJ1_Clone3 CGTCAAAATCAACGCGGCGTACTACAAAA-CTGAGGTTTTGGAAAAGGTT

Aba_MJ1_Clone1 CGTCAAAATCAACGCGGCGTACTACAAAA-CTGAGGTTTTGGAAAAGGTT

Akw_MJ1_Clone1 CGTCAAAATCAACGCGGCGTACTACAAAA-CTGAGGTTTTGGAAAAGGTT

Aju_MJ1_Clone2 CGTCAAAATCAACGCGGCGTACTACAAAA-CTGAGGTTTTGGAAAAGGTT

Aju_MJ1_Clone7 CGTCAAAATCAACGCGGCGTACTACAAAA-CTGAGGTTTTGGAAAAGGTT

Ale_MJ1_Clone4 CGTCAAAATCAACGCGGCGTACTACAAAA-CTGAGGTTTTGGAAAAGGTT

Ale_MJ1_Clone10 CGTCAAAATCAACGCGGCGTACTACAAAA-CTGAGGTTTTGGAAAAGGTT

Aju_MJ1_Clone5 CGTCAAAATCAACGCGGCGTACTACAAAA-CTGAGGTTTTGGAAAAGGTT

Ale_MJ1_Clone5 CGTCAATATCAACGCGGCGTACTACAAAA-CTGAGGTTTTGGAAAAGGTT

Aju_MJ1_Clone6 CGTCAAAATCAACGCGGCGTACTACAAAA-CTGAGGTTTTGGAAAAGGTT

Ale_MJ1_Clone3 CGTCAAAATCAACGCGGCGTACTACAAAA-CTGAGGTTTTGGAAAAGGTT

Ale_MJ1_Clone12 CGTCAAAATCAACGCGGCGTACTACAAAA-CTGAGGTTTTGGAAAAGGTT

Aju_MJ1_Clone4 CGTCAAAATCAACGCGGCGTACTACAAAA-CTGAGGTTTTGGAAAAGGTT

Aju_MJ1_Clone8 CGTCAAAATCAACGCGGCGTACTACAAAA-CTGAGGTTTTGGAAAAGGTT

Ale_MJ1_Clone9 CGTCAAAATCAACGCGGCGTACTACAAAA-CTGAGGTTTTGGAAAAGGTT

Ale_MJ1_Clone6 CGTCAAAATCAACGCGGCGTACTACAAAA-CTGAGGTTTTGGAAAAGGTT

Ale_MJ1_Clone13 CGTCAAAATCAACGCGGCGTACTACAAAA-CTGAGGTTTTGGAAAAGGTT

Ale_MJ1_Clone2 CGTCAAAATCAACGCGGCGTACTACAAAA-CTGAGGTTTTGGAAAAGGTT

Ape_MJ1_Clone1 CGTCAAAATCAACGCGGCGTACTACAAAA-CTGAGGTTTTGGAAAAGGTT

Ape_MJ1_Clone3 CGTCAAAATCAACGCGGCGTACTACAAAA-CTGAGGTTTTGGAAAAGGTT

CONTIG_574 CGTCAAAATCAACGCGGCGTACTACAAAA-CTGAGGTTTTGGAAAAGGTT

CONTIG_7401 CGTCAAAATCAACGCGGCGTACTACAAAA-CTGAGGTTTTGGAAAAGGTT

CONTIG_10225 GTTGCCCCCAGTCTCCGAAGCCTCTACGGCGATGAGCACTACGTGTTCCA

CONTIG_29853 GTTGCCCCCAGTCTCCGAAGCCTCTACGGCGATGAGCACTACGTGTTCCA

CONTIG_11920 GTTGCCCCCAGTCTCCGAAGCCTCTACGGCGATGAGCACTACGTGTTCCA

CONTIG_8991 GTTGCCCCCAGTCTCCGAAGCATCTACGGCGATGAGCACTACGTGTTCCA

CONTIG_23766 GTTGCCCCCAGTCTCCGAAGCCTCTACGGCGATGAGCACTACGTGTTCCA

CONTIG_13910 GTTGCCTCCAGTCTCCGAAGCCTCTACGGCGATGAGCACTACGTGTTCCA

CONTIG_4960 GTTGCCCCCAGTCTCCG---------------------------------

Ape_MJ1_Clone6 GTTGCCCCCAGTCTCCGAAGCCTCTACGGCGATGAGCACTACGTGTTCCA

Ape_MJ1_Clone7 GTTGCCCCCAGTCTCCGAAGCCTCTACGGCGATGAGCACTACGTGTTCCA

Ape_MJ1_Clone2 GTTGCCCCCAGTCTCCGAAGCCTCTACGGCGATGAGCACTACGTGTTCCA

Ale_MJ1_Clone1 GTTGCCTCCAGTCTTCGAAGCCTCTACGGCGATGAGCACTACGTGTTCCA

Ale_MJ1_Clone11 GTTGCCTCCAGTCTTCGAAGCCTCTACGGCGATGAGCACTACGTGTTCCA

Ahy_MJ1_Clone1 GTTGCCCCCAGTCTCCGAAGCCTCTACGGCGATGAGCACTACGTGTTCCA

Ahy_MJ1_Clone2 GTTGCCCCCAGTCTCCGAAGCCTCTACGGCGATGAGCACTACGTGTTCCA

Ale_MJ1_Clone8 GTTGCCCCCAGTCTCCGAAGCCTCTACGGCGATGAGCGCTACGTGTTCCA

Acr_MJ1_Clone2 GTTGCCCCCAGTCTCCGAAGCCTCTACGGCGATGAGCACTACGTGTTCCA

Acr_MJ1_Clone3 GTTGCCCCCAGTCTCCGAAGCCTCTACGGCGATGAGCACTACGTGTTCCA

Acr_MJ1_Clone1 GTTGCCCCCAGTCTCCGAAGCCTCTACGGCGATGAGCACTACGTGTTCCA

Ape_MJ1_Clone4 GTTGCCCCCAGTCTCCGAAGCCTCTACGGCGATGAGCA------------

Ape_MJ1_Clone5 GTTGCCCCCAGTCTCCGAAGCCTCTACGGCGATGAGCA------------

Ahy_MJ1_Clone3 GTTGCCCCCAGTCTCCGAAGCCTCTACGGCGATGAACACTACGTGTTCCA

Ale_MJ1_Clone7 GTTGCCCCCAGTCTCCGAAGCCTCTACGGCGATGAGCACTACGTGTTCCA

Aju_MJ1_Clone3 GTTGCCCCCAGTCTCCGAAGCCTCTACGGCGATGAGCACTACGTGTTCCA

Aya_MJ1_Clone2 GTTGCCCCCAGTCTCCGAAGCCTCTACGGCGATGAGCACTACGTGTTCCA

Asi_MJ1_Clone2 GTTGCCCCCAGTCTCCGAAGCCTCTACGGCGATGAGCACTACGTGTTCCA

Asi_MJ1_Clone7 GTTGCCCCCAGTCTCCGAAGCCTCTACGGCGATGAGCACTACGTGTTCCA

Asi_MJ1_Clone1 GTTGCCCCCAGTCTCCGAAGCCTCTACGGCGATGAGCACTACGTGTTCCA

Asi_MJ1_Clone3 GTTGCCCCCAGTCTCCGAAGCCTCTACGGCGATGAGCACTACGTGTTCCA

Asi_MJ1_Clone4 GTTGCCCCCAGTCTCCGAAGCCTCTACGGCGATGAGCACTACGTGTTCCA

Asi_MJ1_Clone6 GTTGCCCCCAGTCTCCGAAGCCTCTACGGCGATGAGCACTACGTGTTCCA

Akl_MJ1_Clone3 GTTGCCCCCAGTCTCCGAAGCTTCTACGGCGATGAGCACTACGTGTTCCA

Aba_MJ1_Clone2 --------------------------------------------------

Akw_MJ1_Clone3 GTTGCCCCCAGTCTCCGAAGCCTCTACGGCGATGAGCACTACGTGTTCCA

Aya_MJ1_Clone3 GTTGCCCCCAGTCTCCGAAGCCTCTACGGCGATGAGCACTACGTGTTCCA

Aju_MJ1_Clone1 GTTGCCCCCAGTCTCCGAAGCCTCTACGGCGATGAGCACTACGTGTTCCA

Asi_MJ1_Clone5 GTTGCCCCCAGTCTCCGAAGCCTCTACGGTGATGAGCACTACGTGTTCCA

Asi_MJ1_Clone9 GTTGCCCCCAGTCTCCGAAGCCTCTACGGTGATGAGCACTACGTGTTCCA

Akw_MJ1_Clone2 GTTGCCCCCAGTCTCCGAAGCCTCTACGGCGATGAGCACTACGTGTTCCA

Asi_MJ1_Clone8 GTTGCCCCCAGTCTCCGAAGCCTCTACGGCGATGAGCACTACGTGTTCCA

Akl_MJ1_Clone2 GTTGCCCCCAGTCTCCGAAGCCTCTACGGCGATGAGCACTACGTGTTCCA

Aya_MJ1_Clone1 GTTGCCCCCAGTCTCCGAAGCCTCTACGGCGATGAGCACTACGTGTTCCA

Akl_MJ1_Clone1 GTTGCCCCCAGTCTCCGAAGCCTCTACGGCGATGAGCACTACGTGTTCCA

Aba_MJ1_Clone3 GTTGCCCCCAGTCTCCGAAGCCTCTACGGCGATGAGCACTACGTGTTCCA

Aba_MJ1_Clone1 GTTGCCCCCAGTCTCCGAAGCCTCTACGGCGATGAGCACTACGTGTTCCA

Akw_MJ1_Clone1 GTTGCCCCCAGTCTCCGAAGCCTCTACGGCGATGAGCACTACGTGTTCCA

Aju_MJ1_Clone2 GTTGCCCCCAGTCTCCGAAGCCTCTACGGCGATGAGCACTACGTGTTCCA

Aju_MJ1_Clone7 GTTGCCCCCAGTCTCCGAAGCCTCTACGGCGATGAGCACTACGTGTTCCA

Ale_MJ1_Clone4 GTTGCCCCCAGTCTCCGAAGCCTCTACGGCGATGAGCACTACGTGTTCCA

Ale_MJ1_Clone10 GTTGCCCCCAGTCTCCGAAGCCTCTACGGCGATGAGCACTACGTGTTCCA

Aju_MJ1_Clone5 GTTGCCCCCAGTCTCCGAAGCCTCTACGGCGATGAGCACTACGTGTTCCA

Ale_MJ1_Clone5 GTTGCCCCCAGTCTCCGAAGCCTCTACGGCGATGAGCACTACGTGTTCCA

Aju_MJ1_Clone6 GTTGCCCCCAGTCTCCGAAGCCTCTACGGCGATGAGCACTACGTGTTCCA

Ale_MJ1_Clone3 GTTGCCCCCAGTCTCCGAAGCCTCTACGGCGATGAGCACTACGTGTTCCA

Ale_MJ1_Clone12 GTTGCCCCCAGTCTCCGAAGCCTCTACGGCGATGAGCACTACGTGTTCCA

Aju_MJ1_Clone4 GTTGCCCCCAGTCTCCGAAGCCTCTACGGCGATGAGCACTACGTGTTCCA

Aju_MJ1_Clone8 GTTGCCCCCAGTCTCCGAAGCCTCTACGGCGATGAGCACTACGTGTTCCA

Ale_MJ1_Clone9 GTTGCCCCCAGTCTCCGAAGCCTCTACGGCGATGAGCACTACGTGTTCCA

Ale_MJ1_Clone6 GTTGCCCCCAGTCTCCGAAGCCTCTACGGCGATGAGCACTACGTGTTCCA

Ale_MJ1_Clone13 GTTGCCCCCAGTCTCCGAAGCCTCTACGGCGATGAGCACTACGTGTTCCA

Ale_MJ1_Clone2 GTTGCCCCCAGTCTCCGAAGCCTCTACGGCGATGAGCACTACGTGTTCCA

Ape_MJ1_Clone1 GTTGCCCCCAGTCTCCGAAGCCTCTACGGCGATGAGCACTACGTGTTCCA

Ape_MJ1_Clone3 GTTGCCCCCAGTCTCCGAAGCCTCTACGGCGATGAGCACTACGTGTTCCA

CONTIG_574 GTTGCCCCCAGTCTCCGAAGCCTCTACGGCGATGAGCACTGCGTGTTCCA

CONTIG_7401 GTTGTCCCCAGTCTCCGAAGCCTCTACGGCGATGAGCACTACGTGTTCCA

CONTIG_10225 GCAGGACGGTGCACCAGCCCATACGGCAAATGTGGTTTAAGCCTGGTGTC

CONTIG_29853 GCAGGACGGTGCACCAGCCCATACGGCAAATGTGGTTCAAGCCTGGTGTC

CONTIG_11920 GCAGGACGGTGCACCAGCCCATACGGCAAATGTGGTTCAAGCCTGGTGTC

CONTIG_8991 GCAGGACGGTGCACCAGCCCATACGGCAAATGTGGTTCAAGCCTGGTGTC

CONTIG_23766 GCAGGACGGTGCACCAGCCCATACGGCAAATGTGGTTCAAGCCTGGTGTC

CONTIG_13910 GCAGGACGGTGCACCAGCCCATACGGCAAATGTGGTTCAAGCCTGGTGTC

CONTIG_4960 -------------------------GCAAATGTAGTTCAAGCCTGGTGTC

Ape_MJ1_Clone6 GCAGGACGGTGCACCAGCCCATACGGCAAATGTGGTTCAAGCCTGGTGTC

Ape_MJ1_Clone7 GCAGGACGGTGCACCAGCCCATACGGCAAATGTGGTTCAAGCCTGGTGTC

Ape_MJ1_Clone2 GCAGGACGGTGCACCAGCCCATACGGCAAATGTGGTTCAAGCCTGGTGTC

Ale_MJ1_Clone1 GCAGGACGGTGCACCAGCCCATACGGCAAATGTGGTTCAAGCCTGGTGTC

Ale_MJ1_Clone11 GCAGGACGGTGCACCAGCCCATACGGCAAATGTGGTTCAAGCCTGGTGTC

Ahy_MJ1_Clone1 GCAGGACGGTGCACCAGCCCATACGGCAAATGTGGTTCAAGCCTGGTGTC

Ahy_MJ1_Clone2 GCAGGACGGTGCACCAGCCCATACGGCAAATGTGGTTCAAGCCTGGTGTC

Ale_MJ1_Clone8 GCAGGACGGTGCACCAGCCCATACGGCAAATGTGGTTCAAGCCTGGTGTC

Acr_MJ1_Clone2 GCAGGACGGTGCACCAGCCCATACGGCAAATGTGGCTCAAGCCTGGTGTC

Acr_MJ1_Clone3 GCAGGACGGTGCACCAGCCCATACGGCAAATGTGGCTCAAGCCTGGTGTC

Acr_MJ1_Clone1 GCAGGACGGTGCACCAGCCCATACGGCAAATGTGGTTCAAGCCTGCTGTC

Ape_MJ1_Clone4 ---GCACGGTGCACCAGCCCATACGGCAAATGTGGTTCAAGCCTGGTGTC

Ape_MJ1_Clone5 ---GCACGGTGCACCAGCCCATACGGCAAATGTGGTTCAAGCCTGGTGTC

Ahy_MJ1_Clone3 GCAGGACGGTGCACCAGCCCATACGGCAAATGTGGTTCAAGCCTGGTGTC

Ale_MJ1_Clone7 GCAGGACGGTGCACCAGCCCATACGGCAAATGTGGTTCAAGCCTGGTGTC

Aju_MJ1_Clone3 GCAGGACGGTGCACCAGCCCATACGGCAAATGTGGTTCAAGCCTGGTGTC

Aya_MJ1_Clone2 GCAGGACGGTGCACCAGCCCATACGGCAAATGTGGTTCAAGCCTGGTGTC

Asi_MJ1_Clone2 GCAGGACGGTGCACCAGCCCATACGGCAAATGTGGTTCAAGCCTGGTGTC

Asi_MJ1_Clone7 GCAGGACGGTGCACCAGCCCATACGGCAAATGTGGTTCAAGCCTGGTGTC

Asi_MJ1_Clone1 GCAGGACGGTGCACCAGCCCATACGGCAAATGTGGTTCAAGCCTGGTGTC

Asi_MJ1_Clone3 GCAGGACGGTGCACCAGCCCATACGGCAAATGTGGTTCAAGCCTGGTGTC

Asi_MJ1_Clone4 GCAGGACGGTGCACCAGCCCATACGGCAAATGTGGTTCAAGCCTGGTGTC

Asi_MJ1_Clone6 GCAGGACGGTGCACCAGCCCATACGGCAAATGTGGTTCAAGCCTGGTGTC

Akl_MJ1_Clone3 GCAGGACGGTGCACCAGCCCATACGGCAAATGTGGTTCAAGCCTGGTGTC

Aba_MJ1_Clone2 -------------------TGTGCGGCAAATGTGGTTCAAGCCTGGTGTC

Akw_MJ1_Clone3 GCAGCACGGTGCACCAGCCCATACGGCAAATGTGGTTCAAGCCTGGTGTC

Aya_MJ1_Clone3 GCAGGACGGTGCACCAGCCCATACGGCAAATGTGGTTCAAGCCTGGTGTC

Aju_MJ1_Clone1 GCAGGACGGTGCACCAGCCCATACGGCAAATGTGGTTCAAGCCTGGTGTC

Asi_MJ1_Clone5 GCAGGACGGTGCACCAGCCCATACGGCAAATGTGGTTCAAGCCTGGTGTC

Asi_MJ1_Clone9 GCAGGACGGTGCACCAGCCCATACAGCAATTGTGGTTCGAGCCTGGTGTC

Akw_MJ1_Clone2 GCAGGACGGTGCACCAGCCCATACGGCAAATGTGGTTCAAGCCTGGTGTC

Asi_MJ1_Clone8 GCAGGACGGTGCACCAGCCCATACGGCAAATGTGGTTCAAGCCTGGTGTC

Akl_MJ1_Clone2 GCAGGACGGTGCACCAGCCCATACGGCAAATGTGGTTCAAGCCTGGTGTC

Aya_MJ1_Clone1 GCAGGACGGTGCACCAGCCCATACGGCAAATGTGGTTCAAGCCTGGTGTC

Akl_MJ1_Clone1 GCAGGACGGTGCACCAGCCCATACGGCAAATGTGGTTCAAGCCTGGTGTC

Aba_MJ1_Clone3 GCAGGACGGTGCACCAGCCCATACGGCAAATGTGGTTCAAGCCTGGTGTC

Aba_MJ1_Clone1 GCAGGACGGTGCACCAGCCCATACGGCAAATGTGGTTCAAGCCTGGTGTC

Akw_MJ1_Clone1 GCAGGACGGTGCACCAGCCCATACGGCAAATGTGGTTCAAGCCTGGTGTC

Aju_MJ1_Clone2 GCAGGACGGTGCACCAGCCCATACGGCAAATGTGGTTCAAGCCTGGTGTC

Aju_MJ1_Clone7 GCAGGACGGTGCACCAGCCCATACGGCAAATGTGGTTCAAGCCTGGTGTC

Ale_MJ1_Clone4 GCAGGACGGTGCACCAGCCCATACGGCAAATGTGGTTCAAGCCTGGTGTC

Ale_MJ1_Clone10 GCAGGACGGTGCACCAGCCCATACGGCAAATGTGGTTCAAGCCTGGTGTC

Aju_MJ1_Clone5 GCAGGACGGTGCACCAGCCCATACGGCAAATGTGGTTCAAGCCTGGTGTC

Ale_MJ1_Clone5 GCAGGACGGTGCACCAGCCCATACGGCAAATGTGGTTCAAGCCTGGTGTC

Aju_MJ1_Clone6 GCAGGACGGTGCACCAGCCCATACGGCAAATGTGGCTCAAGCCTGGTGTC

Ale_MJ1_Clone3 GCAGGACGGTGCACCAGCCCATACGGCAAATGTGGTTCAAGCCTGGTGTC

Ale_MJ1_Clone12 GCAGGACGGTGCACCAGCCCATACGGCAAATGTGGTTCAAGCCTGGTGTC

Aju_MJ1_Clone4 GCAGGACGGTGCACCAGCCCATACGGCAAATGTGGTTCAAGCCTGGTGTC

Aju_MJ1_Clone8 GCAGGACGGTGCACCAGCCCATACGGCAAATGTGGTTCAAGCCTGGTGTC

Ale_MJ1_Clone9 GCAGGACGGTGCACCAGCCCATACGGCAAATGTGGTTCAAGCCTGGTGTC

Ale_MJ1_Clone6 GCAGGACGGTGCACCAGCCCATACGGCAAATGTGGTTCAAGCCTGGTGTC

Ale_MJ1_Clone13 GCAGGACGGTGCACCAGCCCATATGGCAAATGTGGTTCAAGCCTGGTGTC

Ale_MJ1_Clone2 GCAGGACGGTGCACCAGCCCATACGGCAAATGTGGTTCAAGCCTGGTGTC

Ape_MJ1_Clone1 GCAGGACGGTGCACCAGCCCATACGGCAAATGTGGTTCAAGCCTGGTGTC

Ape_MJ1_Clone3 GCAGGACGGTGCACCAGCCCATACGGCAAATGTGGTTCAAGCCTGGTGTC

CONTIG_574 GCAGGACGGTGCACCAGCCCATACGGCAAATGTGGTTCAAGCCTGGTGTC

CONTIG_7401 GCAGGACGGTGCACCAGCCCATACGGCAAATGTGGTTCAAGCCTGGTGTC

CONTIG_10225 GGGACAATTTAACCGACTTTCTGGACAAAACTTTGTGGCCTCCCAGCTCC

CONTIG_29853 GGGACAATTTAACCGACTTTCTGGACAAAACTTTGTGGCCTCCCAGCTCC

CONTIG_11920 GGGACAATTTAACCGATTTTCTGGACAAAACTTTGTGGCCTCCCAGCTCC

CONTIG_8991 GGGACAATTTAACCGACTTTCTGGACAAAACTTTGTGGCCTCCCAGCTCC

CONTIG_23766 GGGACAATTTAACCAACTTTCTGGACAAAACTTTGTGGCCTCCCAGCTCC

CONTIG_13910 GGGACAATTTAACCGACTTTCTGGACAAAACTTTGTGGCCTCCCAGCTCC

CONTIG_4960 GGGACAATTTAACCGACTTTCTGGACAAAACTTTGTGGCCTCCCAGCTCC

Ape_MJ1_Clone6 GGGACAATTTAACCGACTTTCTGGACAAAACTTTGTGGCCTCCCAGCTCC

Ape_MJ1_Clone7 GGGACAATTTAACCGACTTTCTGGACAAAACTTTGTGGCCTCCCAGCTCC

Ape_MJ1_Clone2 GGGACAATTTAACCGACTTTCTGGACAAAACTTTGTGGCCTCCCAGCTCC

Ale_MJ1_Clone1 GGGACAATTTAACCGACTTTCTGGACAAAACTTTGTGGCCTCCCAGCTCC

Ale_MJ1_Clone11 GGGACAATTTAACCGACTTTCTGGACAAAACTTTGTGGCCTCCCAGCTCC

Ahy_MJ1_Clone1 GGGACAATTTAACCGACTTTCTGGACAAAACTTTGTGGCCTCCCAGCTCC

Ahy_MJ1_Clone2 GGGACAATTTAACCGACTTTCTGGACAAAACTTTGTGGCCTCCCAGCTCC

Ale_MJ1_Clone8 CGGACAATTTAACCGACTTTCTGGACAAAACTTTGTGGCCTCCCAGCTCC

Acr_MJ1_Clone2 GGGACAATTTAACCGACTTTCTGGACAAAACTTTGTGGCCTCCTAGCTCC

Acr_MJ1_Clone3 GGGACAATTTAACCGACTTTCTGGACAAAACTTTGTGGCCTCCTAGCTCC

Acr_MJ1_Clone1 GGGACAATTTAACCGACTTTCTGGACAAAACTTTGTGGCCTCCCAGCTCC

Ape_MJ1_Clone4 GGGACAATTTAACCGACTTTCTGGACAAAACTTTGTGGCCTCCCAGCTCC

Ape_MJ1_Clone5 GGGACAATTTAACCGACTTTCTGGACAAAACTTTGTGGCCTCCCAGCTCC

Ahy_MJ1_Clone3 GGGACAATTTAACCGACTTTCTGGACAAAACTTTGTGGCCTCCCAGCTCC

Ale_MJ1_Clone7 GGGACAATTTAACCGACTTTCTGGACAAAACTTTGTGGCCTCCCAGCTCC

Aju_MJ1_Clone3 GGGACAATTTAACCGACTTTCTGGACAAAACTTTGTGGCCTCCCAGCTCC

Aya_MJ1_Clone2 GGGACAATTTAACCGACTTTCTGGACAAAACTTTGTGGCCTCCCAGCTCC

Asi_MJ1_Clone2 GGGACAATTTAACCGACTTTCTGGACAAAACTTTGTGGCCTCCCAGCTCC

Asi_MJ1_Clone7 GGGACAATTTAACCGACTTTCTGGACAAAACTTTGTGGCCTCCCAGCTCC

Asi_MJ1_Clone1 GGGACAATTTAACCGACTTTCTGGACAAAACTTTGTGGCCTCCCAGCTCC

Asi_MJ1_Clone3 GGGACAATTTAACCGACTTTCTGGACAAAACTTTGTGGCCTCCCAGCTCC

Asi_MJ1_Clone4 GGGACAATTTAACCGACTTTCTGGACAAAACTTTGTGGCCTCCCAGCTCC

Asi_MJ1_Clone6 GGGACAATTTAACCGACTTTCTGGACAAAACTTTGTGGCCTCCCAGCTCC

Akl_MJ1_Clone3 GGGACAATTTAACCGACTTTCTGGACAAAACTTTGTGGCCTCCCAGCTCC

Aba_MJ1_Clone2 GGGACAATTTAACCGACTTTCTGGACAAAACTTTGTGGCCTCCCAGCTCC

Akw_MJ1_Clone3 GGGACAATTTAACCGACTTTCTGGACAAAACTTTGTGGCCTCCCAGCTCC

Aya_MJ1_Clone3 GGGACAATTTAACCGACTTTCTGGACAAAACTTTGTGGCCTCCCAGCTCC

Aju_MJ1_Clone1 GGGACAATTTAACCGACTTTCTGGACAAAACTTTGTGGCCTCCCAGCTCC

Asi_MJ1_Clone5 GGGACAATTTAACCGACTTTCTGGACAAAACTTTGTGGCCTCCCAGCTCC

Asi_MJ1_Clone9 GGGACAATTTAACCGACTTTCTGGACAAAACTTTGTGGCCTCCCAGCTCC

Akw_MJ1_Clone2 GGGACAATTTAACCGACTTTCTGGACAAAACTTTGTGGCCTCCCAGCTCC

Asi_MJ1_Clone8 GGGACAATTTAACCGACTTTCTGGACAAAACTTTGTGGCCTCCCAGCTCC

Akl_MJ1_Clone2 GGGACAATTTAACCGACTTTCTGGACAAAACTTTGTGGCCTCCCAGCTCC

Aya_MJ1_Clone1 GGGACAATTTAACCGACTTTCTGGACAAAACTTTGTGGCCTCCCAGCTCC

Akl_MJ1_Clone1 GGGACAATTTAACCGACTTTCTGGACAAAACTTTGTGGCCTCCCAGCTCC

Aba_MJ1_Clone3 GGGACAATTTAACCGACTTTCTGGACAAAACTTTGTGGCCTCCCAGCTCC

Aba_MJ1_Clone1 GGGACAATTTAACCGACTTTCTGGACAAAACTTTGTGGCCTCCCAGCTCC

Akw_MJ1_Clone1 GGGACAATTTAACCGACTTTCTGGACAAAACTTTGGGACCTCCCAGCTTC

Aju_MJ1_Clone2 GGGACAATTTAACCGACTTTCTGGACAAAACTTTGTGGCCTCCCAGCTCC

Aju_MJ1_Clone7 GGGACAATTTAACCGACTTTCTGGACAAAACTCTGTGGCCTCCCAGCTCC

Ale_MJ1_Clone4 GGGACAATTTAACCGACTTTCTGGACAAAACTTTGTGGCCTCCCAGCTCC

Ale_MJ1_Clone10 GGGACAATTTAACCGACTTTCTGGACAAAACTTTGTGGCCTCCCAGCTCC

Aju_MJ1_Clone5 GGGACAATTTAACCGACTTTCTGGACAAAACTTTGTGGCCTCCCAGCTCC

Ale_MJ1_Clone5 GGGACAATTTAACCGACTTTCTGGACAAAACTTTGTGGCCTCCCAGCTCC

Aju_MJ1_Clone6 GGGACAATTTAACCGACTTTCTGGACAAAACTTTGTGGCCTCCCAGCTCC

Ale_MJ1_Clone3 GGGACAATTTAACCGACTTTCTGGACAAAACTTTGTGGCCTCCCAGCTCC

Ale_MJ1_Clone12 GGGACAATTTAACCGACTTTCTGGACAAAACTTTGTGGCCTCCCAGCTCC

Aju_MJ1_Clone4 GGGACAATTTAACCGACTTTCTGGACAAAACTTTGTGGCCTCCCAGCTCC

Aju_MJ1_Clone8 GGGACAATTTAACCGACTTTCTGGACAAAACTTTGTGGCCTCCCAGCTCC

Ale_MJ1_Clone9 GGGACAATTTAACCGACTTTCTGGACAAAACTTTGTGGCCTCCCAGCTCT

Ale_MJ1_Clone6 GGGACAATTTAACCGACTTTCTGGACAAAACTTTGTGGCCTCCCAGCTCC

Ale_MJ1_Clone13 GGGACAATTTAACCGACTTTCTGGACAAAACTTTGTGGCCTCCCAGCTCC

Ale_MJ1_Clone2 GGGACAATTTAACCGACTTTCTGGACAAAACTTTGTGGCCTCCCAGCTCC

Ape_MJ1_Clone1 GGGACAATTTAACCGACTTTCTGGACAAAACTTTGTGGCCTCCCAGCTCC

Ape_MJ1_Clone3 GGGACAATTTAACCGACTTTCTGGACAAAACTTTGTGGCCTCCCAGCTCC

CONTIG_574 GGGACAATTTAACCGACTTTCTGGACAAAACTTTGTGGCCTCCCAGCTCC

CONTIG_7401 GGGAAAATTTAACCGACTTTCTGGACAAAACTTTGTGGCCTCCCAGCTCC

CONTIG_10225 CCGGACTTGAATCCTCTCGACTTTTTTGTTTGGTCCTATATGATGGCGAA

CONTIG_29853 CCGGACTTGAATCCTCTCGACTTTTTTGTTTGGTCCTATATGATGACGAA

CONTIG_11920 CCGGACTTGAATCCTCTCGACTTTTTTGTTTGGTCCTATATGATGGCGAA

CONTIG_8991 CCGGACTTGAATCCTCTCGAATTTTTTGTTTGGTCCTATATGATGGCGAA

CONTIG_23766 CCGGACTTGAATCCTCTCGACTTTTTTGTTTGGTCCTATATGATGGCGAA

CONTIG_13910 CCGGACTTGAATCCTCTCGACTTTTTTGTTTGGTCCTATATGATGGCGAA

CONTIG_4960 CCGGACTTGAATCCTCTCGACTTTTTTGTTTGGTCCTATATGATGGCGAA

Ape_MJ1_Clone6 CCGGACTTGAATCCTCTCGACTTTTTTGTTTGGTCCTATATGATGGCGAA

Ape_MJ1_Clone7 CCGGACTTGAATCCTCTCGACTTTTTTGTTTGGTCCTATATGATGGCGAA

Ape_MJ1_Clone2 CCGGACTTGAATCCTCTCGACTTTTTTGTTTGGTCCTATATGATGGCGAA

Ale_MJ1_Clone1 CCGGACTTGAATCCTCTCGACTTTTTTGTTTGGTCCTATATGATGGCGAA

Ale_MJ1_Clone11 CCGGACTTGAATCCTCTCGACTTTTTTGTTTGGTCCTATATGATGGCGAA

Ahy_MJ1_Clone1 CCGGACTTGAATCCTCTCGACTTTTTTGTTTGGTCCTATATGATGGCGAA

Ahy_MJ1_Clone2 CCGGACTTGAATCCTCTCGACTTTTTTGTTTGGTCCTATATGATGGCGAA

Ale_MJ1_Clone8 CCGGACTTGAATCCTCTCGACTTTTTTGTTTGGTCCTATATGATGGCGAA

Acr_MJ1_Clone2 CCGGACTTGAATCCTCTCGACTTTTTTGTTTGGTCCTATATGATGGCGAA

Acr_MJ1_Clone3 CCGGACTTGAATCCTCTCGACTTTTTTGTTTGGTCCTATATGATGGCGAA

Acr_MJ1_Clone1 CCGGACTTGAATCCTCTCGACTTTTTTGTTTGGTCCTATATGATGGCGAA

Ape_MJ1_Clone4 CCGGACTTGAATCCTCTCGACTTTTTTGTTTGGTCCTATATGATGGCGAA

Ape_MJ1_Clone5 CCGGACTTGAATCCTCTCGACTTTTTTGTTTGGTCCTATATGATGGCGAA

Ahy_MJ1_Clone3 CCGGACTTGAATCCTCTCGACTTTTTTGTTTGGTCCTATATGATGGCGAA

Ale_MJ1_Clone7 CCGGACTTGAATCCTCTCGACTTTTTTGTTTGGTCCTATATGATGGCGAA

Aju_MJ1_Clone3 CCGGACTTGAATCCTCTCGACTTTTTTGTTTGGTCCTATATGATGGCGAA

Aya_MJ1_Clone2 CCGGACTTGAATCCTCTCGACTTTTTTGTTTGGTCCTATATGATGGCGAA

Asi_MJ1_Clone2 CCGGACTTGAATCCTCTCGACTTTTTTGTTTGGTCCTATATGATGGCGAA

Asi_MJ1_Clone7 CCGGACTTGAATCCTCTCGACTTTTTTGTTTGGTCCTATATGATGGCGAA

Asi_MJ1_Clone1 CCGGACTTGAATCCTCTCGACTTTTTTGTTTGGTCCTATATGATGGCGAA

Asi_MJ1_Clone3 CCGGACTTGAATCCTCTCGACTTTTTTGTTTGGTCCTATATGATGGCGAA

Asi_MJ1_Clone4 CCGGACTTGAATCCTCTCGACTTTTTTGTTTGGTCCTATATGATGGCGAA

Asi_MJ1_Clone6 CCGGACTTGAATCCTCTCGACTTTTTTGTTTGGTCCTATATGATGGCGAA

Akl_MJ1_Clone3 CCGGACTTGAATCCTCTCGACTTTTTTGTTTGGTCCTATATGATGGCGAA

Aba_MJ1_Clone2 CCGGACTTGAATCCTCTCGACTTTTTTGTTTGGTCCTATATGATGGCGAA

Akw_MJ1_Clone3 CCGGACTTGAATCCTCTCGACTTTTTTGTTTGGTCCTATATGATGGCGAA

Aya_MJ1_Clone3 CCGGACTTGAATCCTCTCGACTTTTTTGTTTGGTCCTATATGATGGCGAA

Aju_MJ1_Clone1 CCGGACTTGAATCCTCTCGACTTTTTTGTTTGGTCCTATATGATGGCGAA

Asi_MJ1_Clone5 CCGGACTTGAATCCTCTCGACTTTTTTGTTTGGTCCTATATGATGGCGAA

Asi_MJ1_Clone9 CCGGACTTGAATCCTCTCGACTTTTTTGTTTGGTCCTATATGATGGCGAA

Akw_MJ1_Clone2 CCGGACTTGAATCCTCTCGACTTTTTTGTTTGGTCCTATATGATGGCGAA

Asi_MJ1_Clone8 CCGGACTTGAATCCTCTCGACTTTTTTGTTTGGTCCTATATGATGGCGAA

Akl_MJ1_Clone2 CCGGACTTGAATCCTCTCGACTTTTTTGTTTGGTCCTATATGATGGCGAA

Aya_MJ1_Clone1 CCGGACTTGAATCCTCTCGACTTTTTTGTTTGGTCCTATATGATGGCGAA

Akl_MJ1_Clone1 CCGGACTTGAATCCTCTCGACTTTTTTGTTTGGTCCTATATGATGGCGAA

Aba_MJ1_Clone3 CCGGACTTGAATCCTCTCGACTTTTTTGTTTGGTCCTATATGATGGCGAA

Aba_MJ1_Clone1 CCGGACTTGAATCCTCTCGACTTTTTTGTTTGGTCCTATATGATGGCGAA

Akw_MJ1_Clone1 CCGGACTTGAATCCTCTCGACTTTTTTGTTTGGTCCTATATGATGGCGAA

Aju_MJ1_Clone2 CCGGACTTGAATCCTCTCGACTTTTTTGTTTGGTCCTATATGATGGCGAA

Aju_MJ1_Clone7 CCGGACTTGAATCCTCTCGACTTTTTTGTTTGGTCCTATATGATGGCGAA

Ale_MJ1_Clone4 CCGGACTTGAATCCTCTCGACTTTTTTGTTTGGTCCTATATGATGGCGAA

Ale_MJ1_Clone10 CCGGACTTGAATCCTCTCGACTTTTTTGTTTGGTCCTATATCATGGCGAA

Aju_MJ1_Clone5 CCGGACTTGAATCCTCTCGACTTTTTTGTTTGGTCCTATATGATGGCGAA

Ale_MJ1_Clone5 CCGGACTTGAATCCTCTCGACTTTTTTGTTTGGTCCTATATGATGGCGAA

Aju_MJ1_Clone6 CCGGACTTGAATCCTCTCGACTTTTTTGTTTGGTCCTATATGATGGCGAC

Ale_MJ1_Clone3 CCGGACTTGAATCCTCTCGACTTTTTTGTTTGGTCCTATATGATGGCGAA

Ale_MJ1_Clone12 CCGGACTTGAATCCTCTCGACTTTTTTGTTTGGTCCTATATGATGGCGAA

Aju_MJ1_Clone4 CCGGACTTGAATCCTCTCGACTTTTTTGTTTGGTCCTATATGATGGCGAA

Aju_MJ1_Clone8 CCGGACTTGAATCCTCTCGACTTTTTTGTTTGGTCCTATATGATGGCGAA

Ale_MJ1_Clone9 CCGGACTTGAATCCTCTCGACTTTTTTGTTTGGTCCTATATGATGGCGAA

Ale_MJ1_Clone6 CCGGACTTGAATCCTCTCGACTTTTTTGTTTGGTCCTATATGATAGCGAA

Ale_MJ1_Clone13 CCAGACTTGAATCCTCTCGACTTTTTTGTTTGGTCCTATATGATGGCGAA

Ale_MJ1_Clone2 CCGGACTTGAATCCTCTCGACTTTTTTGTTTGGTCCTATATGATGGCGAA

Ape_MJ1_Clone1 CCGGACTTGAATCCTCTCGACTTTTTTGTTTGGTCCTATATGATGGCGAA

Ape_MJ1_Clone3 CCGGACTTGAATCCTCTCGACTTTTTTGTTTGGTCCTATATGATGGCGAA

CONTIG_574 CCGGACTTGAATCCTCTCGACTTTTTTGTTTGGTCCTATATGATGGCGAA

CONTIG_7401 CCGGACTTGAATCCTCTCGACTTTTTTGTTTGGTCCTATATGATGGCGAA

CONTIG_10225 GCTGAACGAATACAAGGTCAGCACTTTGGACCATTTCAAGACGGTAATTC

CONTIG_29853 GCTGAACGAATACAAGGTCAGCACTTTGGACCATTTCAAGACGGTAATTC

CONTIG_11920 GCTGAACGAATACAAGGTCAGCACTTTGGACCATTTCAAGACGGTAATTC

CONTIG_8991 GCTGAACGAATACAAGGTCAGCACTTTGGACCATTTCAAGACGGTAATTC

CONTIG_23766 GCTGAACGAATACAAGGTCAGCACTTTGGACCATTTCAAGACGGTAATTC

CONTIG_13910 GCTGAACGAATACAATGTCAGCACTTTGGACCATTTCAAGACAGTAATTC

CONTIG_4960 GCTGAACGAATACAAGGTCAGCACTTTGGACCATTTCAAGACGGTAATTC

Ape_MJ1_Clone6 GCTGAACGAATACAAGGTCAGCACTTTGGATCATTTCAAGACGGTAATTC

Ape_MJ1_Clone7 GCTGAACGAATACAAGGTCAGCACTTTGGATCATTTCAAGACGGTAATTC

Ape_MJ1_Clone2 GCTGAACGAATACAAGATCAGCACTTTGGATCATTTCAAGACGGTAATTC

Ale_MJ1_Clone1 GCTGAACGAATACAAGGTCAGCACTTTGGATCATTTCAAGACGGTAATTC

Ale_MJ1_Clone11 GCTGAACGAATACAAGGTCAGCACTTTGGATCATTTCAAGACGGTAATTC

Ahy_MJ1_Clone1 GCTGAACGAATACAAGGTCAGCACTTTGGATCATTTCAAGACGGTAATTC

Ahy_MJ1_Clone2 GCTGAACGAATACAAGGTCAGCACTTTGGATCATTTCAAGACGGTAATTC

Ale_MJ1_Clone8 GCTGAACGAATACAAGGTCAGCACTTTGGATCATTTCAAGACGGTAATTC

Acr_MJ1_Clone2 GCTGAACGAATACAAGGTCAGCACTTTGGATCATTTCAAGACGGTAATTC

Acr_MJ1_Clone3 GCTGAACGAATACAAGGTCAGCACTTTGGATCATTTCAAGACGGTAATTC

Acr_MJ1_Clone1 GCTGAACGAATACAAGGTCAGCACTTTGGATCATTTCAAGACAGTAATTC

Ape_MJ1_Clone4 GCTGAACGAATACAAGGTCAGCACTTTGGATCATTTCAAGACGGTAATTC

Ape_MJ1_Clone5 GCTGAACGAATACAAGGTCAGCACTTTGGATCATTTCAAGACGGTAATTC

Ahy_MJ1_Clone3 GCTGAACGAATACAAGGTCAGCACTTTGGATCATTTCAAGACGGTAATTC

Ale_MJ1_Clone7 GCTGAACGAATACAAGGTCAGCACTTTGGATCATTTCAAGACGGTAATTC

Aju_MJ1_Clone3 GCTGAACGAATACAAGGTCAGCACTTTGGATCATTTCAAGACGGTAATTC

Aya_MJ1_Clone2 GCTGAACGAATACAAGGTCAGCACTTTGGATCATTTCAAGACGGTAATTC

Asi_MJ1_Clone2 GCTGAACGAATACAAGGTCAGCACTTTGGATCATTTCAAGACGGTAATTC

Asi_MJ1_Clone7 GCTGAACGAATACAAGGTCAGCACTTTGGATCATTTCAAGACGGTAATTC

Asi_MJ1_Clone1 GCTGAACGAATACAAGGTCAGCACTTTGGATCATTTCAAGACGGTAATTC

Asi_MJ1_Clone3 GCTGAACGAATACAAGGTCAGCACTTTGGATCATTTCAAGACGGTAATTC

Asi_MJ1_Clone4 GCTGAACGAATACAAGGTCAGCACTTTGGATCATTTCAAGACGGTAATTC

Asi_MJ1_Clone6 GCTGAACGAATACAAGGTCAGCACTTTGGATCATTTCAAGACGGTAATTC

Akl_MJ1_Clone3 GCTGAACGAATACAAGGTCAGCACTTTGGATCATTTCAAGACGGTAATTC

Aba_MJ1_Clone2 GCTGAACGAATACAAGGTCAGCACTTTGGATCATTTCAAGACGGTAATTC

Akw_MJ1_Clone3 GCTGAACGAATACAAGGTCAGCACTTTGGATCATTTCAAGACGGTAATTC

Aya_MJ1_Clone3 GCTGAACGAATACAAGGTCAGCACTTTGGATCATTTCAAGACGGTAATTC

Aju_MJ1_Clone1 GCTGAACGAATACAAGGTCAGCACTTTGGATCATTTCATGACGGTAATGC

Asi_MJ1_Clone5 GCTGAACGAATACAAGGTCAGCACTTTGGATCATTTCAAGACGGTAATTC

Asi_MJ1_Clone9 GCTGAACGAATACAAGGTCAGCACTTTGGATCATTTCAAGCCGGTAATTC

Akw_MJ1_Clone2 GCTGAACGAATACAAGGTCAGCACTTTGGATCATTTCAAGACGGTAATTC

Asi_MJ1_Clone8 GCTGAACGAATACAAGGTCAGCACTTTGGATCATTTCAAGACGGTAATTC

Akl_MJ1_Clone2 GCTGAACGAATACAAGGTCAGCACTTTGGATCATTTCAAGACGGTAATTC

Aya_MJ1_Clone1 GCTGAACGAATACAAGGTCAGCACTTTGGATCATTTCAAGACGGTAATTC

Akl_MJ1_Clone1 GCTGAACGAATACAAGGTCAGCACTTTGGATCATTTCAAGACGGTAATTC

Aba_MJ1_Clone3 GCTGAACGAATACAAGGTCAGCACTTTGGATCATTTCAAGACGGTAATTC

Aba_MJ1_Clone1 GCTGAACGAATACAAGGTCAGCACTTTGGATCATTTCAAGACGGTAATTC

Akw_MJ1_Clone1 GCTGAACGAATACAAGGTCAGCACTTTGGATCATTTCAAGACGGTAATTC

Aju_MJ1_Clone2 GCTGAACGAATACAAGGTCAGCACTTTGGATCATTTCAAGACGGTAATTC

Aju_MJ1_Clone7 GCTGAACGAATACAAGGTCAGCACTTTGGATCATTTCAAGACGGTAATTC

Ale_MJ1_Clone4 GCTGAACGAATACAAGGTCAGCACTTTGGATCATTTCAAGACGGTAATTC

Ale_MJ1_Clone10 GCTGAACGAATACAAGGTCAGCACTTTGGATCATTTCAAGACGGTAATTC

Aju_MJ1_Clone5 GCTGAACGAATACAAGGTCAGCACTTTGGATCATTTCAAGACGGTAATTC

Ale_MJ1_Clone5 GCTGAACGAATACAAGGTCAGCACTTTGGATCATTTCAAGACGGTAATTC

Aju_MJ1_Clone6 GCTGAACGAATACAAGGTCAGCACTTTGGATCATTTCAAGACGGTAATTC

Ale_MJ1_Clone3 GCTGAACGAATACAAGGTCAGCACTTTGGATCATTTCAAGACGGTAATTC

Ale_MJ1_Clone12 GCTGAACGAATACAAGGTCAGCACTTTGGATCATTTCAAGACGGTAATTC

Aju_MJ1_Clone4 GCTGAACGAATACAAGGTCAGCACTTTGGATCATTTCAAGACGGTAATTC

Aju_MJ1_Clone8 GCTGAACGAATACAAGGTCAGCACTTTGGATCATTTCAAGACGGTAATTC

Ale_MJ1_Clone9 GCTGAACGAATACAAGGTCAGCACTTTGGATCATTTCAAGACGGTAATTC

Ale_MJ1_Clone6 GCTGAACGAATACAAGGTCAGCACTTTGGATCATTTCAAGACGGTAATTC

Ale_MJ1_Clone13 GCTGAACGAATACAAGGTCAGCACTTTGGATCATTTCAAGACGGTAATTC

Ale_MJ1_Clone2 GCTGAACGAATACAAGGTCAGCACTTTGGATCATTTCAAGACGGTAATTC

Ape_MJ1_Clone1 GCTGAACGAATACAAGGTCAGCACTTTGGATCATTTCAAGACGGTAATTC

Ape_MJ1_Clone3 GCTGAACGAATACAAGGTCAGCACTTTGGATCATTTCAAGACGGTAATTC

CONTIG_574 GCTGAACGAATACAAGGTCAGCACTTTGGACCATTTCAAGACGGTAATTC

CONTIG_7401 GCTGAACGAATACAAGGTCAGCACTTTGGACCATTTCAAGACGGTAATTC

CONTIG_10225 TCAAAATCTGGGACGAAATGCCCATGCAGT-CCGTGCG-TGCCGCTTGCG

CONTIG_29853 TCAAAATCTGGGACGAAATGCCCATGCAGT-CCGTGCG-TGCCGCTTGCG

CONTIG_11920 TCAAAATCTGGGACGAAATGCCCATGCAGT-CCGTGCG-TGCCGCTTGCG

CONTIG_8991 TCAAAATCTAGGACGAAATGCCCATGCAGT-CCGTGCG-TGCCGCTTGCG

CONTIG_23766 TCAAAATCTGGGACGAAATGCCCATGCAGT-CCGTGCG-TGCCGCTTGCG

CONTIG_13910 TCAAAATCTGGGACGAAATGCCCATGCAGT-CCGTGCG-TGCCGCTTGCG

CONTIG_4960 TCAAAATCTGGGACGAAATGCCCATGCAGT-CCGTGCG-TGCCGCTTGCG

Ape_MJ1_Clone6 TTAAAATCTGGGACGACATGCCCATGCAGT-CCGTGCG-TGCCGCCTGCG

Ape_MJ1_Clone7 TTAAAATCTGGGACGACATGCCCATGCAGT-CCGTGCG-TGCCGCCTGCG

Ape_MJ1_Clone2 TCAAAATCTGGGACGAAATGCCCATGCAGT-CCGTGCG-TGCCGCCTGCG

Ale_MJ1_Clone1 TCAAAATCTGGGACGAAATGCCCATGCAGT-CCGTGCG-TGCCGCCTGCG

Ale_MJ1_Clone11 TCAAAATCTGGGACGAAATGCCCATGCAGT-CCGTGCG-TGCCGCCTGCG

Ahy_MJ1_Clone1 TCAAAATCTGGGACGAAATGCCCATGCAGT-CCGTGCG-TGCCGCCTGCG

Ahy_MJ1_Clone2 TCAAAATCTGGGACGAAATGCCCATGCAGT-CCGTGCG-TGCCGCCTGCG

Ale_MJ1_Clone8 TCAAAATCTGGGACGAAATGCCCATGCAGT-CCGTGCG-TGCCGCCTGCG

Acr_MJ1_Clone2 TCGAAATCTGGGACGAAATGCCCATGCAGT-CCGTGCG-TGCCGCCTGCG

Acr_MJ1_Clone3 TCGAAATCTGGGACGAAATGCCCATGCAGT-CCGTGCG-TGCCGCCTGCG

Acr_MJ1_Clone1 TCGAAATCTGGGACGAAATGCCCATGCAGT-CCGTGCG-TGCCGCCTGCG

Ape_MJ1_Clone4 TCAAAATCTGGGACGAAATGCCCATGCAGT-CCGTGCG-TGCCGCCTGCG

Ape_MJ1_Clone5 TCAAAATCTGGGACGAAATGCCCATGCAGT-CCGTGCG-TGCCGCCTGCG

Ahy_MJ1_Clone3 TCAAAATCTGGGACGAAATGCCCATGCAGT-CCGTGCG-TGCCGCCTGCG

Ale_MJ1_Clone7 TCAAAATCTGGGACGAAATGCCCATGCAGT-CCGTGCG-TGCCGCCTGCG

Aju_MJ1_Clone3 TCAAAATCTGGGACGAAATGCCCATGCAGT-CCGTGCG-TGCCGCCTGCG

Aya_MJ1_Clone2 TCAAAATCTGGGACGAAATGCCCATGCAGT-CCGTGCG-TGCCGCCTGCG

Asi_MJ1_Clone2 TCAAAATCTGGGACGAAATGCCCATGCAGT-CCGTGCG-TGCCGCCTGCG

Asi_MJ1_Clone7 TCAAAATCTGGGACGAAATGCCCATGCAGT-CCGTGCGGTGCCGCCTGCG

Asi_MJ1_Clone1 TCAAAATCTGGGACGAAATGCCCATGCAGT-CCGTGCG-TGCCGCCTGCG

Asi_MJ1_Clone3 TCAAAATCTGGGACGAAATGCCCATGCAGT-CCGTGCG-TGCCGCCTGCG

Asi_MJ1_Clone4 TCAAAATCTGGGACGAAATGCCCATGCAGT-CCGTGCG-TGCCGCCTGCG

Asi_MJ1_Clone6 TCAAAATCTGGGACGAAATGCCCATGCAGT-CCGTGCG-TGCCGCCTGCG

Akl_MJ1_Clone3 TCAAAATCTGGGACGAAATGCCCATGCAGT-CCGTGCG-TGCCGCCTGCG

Aba_MJ1_Clone2 TCAAAATCTGGGACGAAATGCCCATGCAGT-CCGTGCG-TGCCGCCTGCG

Akw_MJ1_Clone3 TCAAAATCTGGGACGAAATGCCCATGCAGT-CCGTGCG-TGCCGCCTGCG

Aya_MJ1_Clone3 TCAAAATCTGGGACGAAATGCCCATGCAGT-CCGTGCG-TGCCGCCTGCG

Aju_MJ1_Clone1 TCAAAATCTGGGACGAAATGCCCATGCAGT-CCGTGCG-TGCCGCCTGCG

Asi_MJ1_Clone5 TCAAAATCTGGGACGAAATGCCCATGCAGT-CCGTGCG-TGCCGCCTGCG

Asi_MJ1_Clone9 TCAAAATCTGGGACGAAATGCCCATGCAGT-CCGTGCG-TGCCGCCTGCG

Akw_MJ1_Clone2 TCAAAATCTGGGACGAAATGCCCATGCAGT-CCGTGCG-TGCCGCCTGCG

Asi_MJ1_Clone8 TCAAAATCTGGGACGAAATGCCCATGCAGT-TCGTGCG-TGCCGCCTGCG

Akl_MJ1_Clone2 TCAAAATCTGGGACGAAATGCCCATGCAGT-CCGTGCG-TGCCGCCTGCG

Aya_MJ1_Clone1 TCAAAATCTGGGACGAAATGCCCATGCGGT-CCGTGCG-TGCCGCCTGCG

Akl_MJ1_Clone1 TCAAAATCTGGGACGAAATGCCCATGCATTTCCGTGCG-TGCCGCCTGCG

Aba_MJ1_Clone3 TCAAAATCTGGGACGAAATGCCCATGCAGT-CCGTGCG-TGCCGCCTGCG

Aba_MJ1_Clone1 TCAAAATCTGGGACGAAATGCCCATGCAGT-CCGTGCG-TGCCGCCTGCG

Akw_MJ1_Clone1 TCAAAATCTGGGACGAAATGCCCATGCAGT-CCGTGCG-TGCCGCCTGCG

Aju_MJ1_Clone2 TCAAAATCTGGGACGAAATGCCCATGCAGT-CCGTGCG-TGCCGCCTGCG

Aju_MJ1_Clone7 TCAAAATCTGGGACGAAATGCCCATGCAGT-CCGTGCG-TGCCGCCTGCG

Ale_MJ1_Clone4 TCAAAATCTGGGACGAAATGCCCATGCAGT-CCGTGCG-TGCCGCCTGCG

Ale_MJ1_Clone10 TCAAAATCTGGGACGAAATGCCCATACAGT-CCGTGCG-TGCCGCCTGCG

Aju_MJ1_Clone5 TCAAAATCTGGGACGAAATGCCCATGCAGT-CCGTGCG-TGCCGCCTGCG

Ale_MJ1_Clone5 TCAAAATCTGGGACGAAATGCCCATGCAGT-CCGTGCG-TGCCGCCTGCG

Aju_MJ1_Clone6 TCAAAATCTGGGACGAAATGCTCATGCAGT-CCGTGCG-TGCCGCCTGCG

Ale_MJ1_Clone3 TCAAAATCTGGGACGAAATGCCCATGCAGT-CCGTGCG-TGCCGCCTGCG

Ale_MJ1_Clone12 TCAAAATCTGGGACGAAATGCCCATGCAGT-CCGTGCG-TGCCGCCTGCG

Aju_MJ1_Clone4 TCAAAATCTGGGACGAAATGCCCATGCAGT-CCGTGCG-TGCCGCCTGCG

Aju_MJ1_Clone8 TCAAAATCTGGGACGAAATGCCCATGCAGT-CCGTGCG-TGCCGCCTGCG

Ale_MJ1_Clone9 TCAAAATCTGGGACGAAATGCCCATGCAGT-CCGTGCG-TGCCGCCTGCG

Ale_MJ1_Clone6 TCAAAATCTGGGACGAAATGCCCATGCAGT-CCGTGCG-TGCCGCCTGCG

Ale_MJ1_Clone13 TCAAAATCTGGGACGAAATGCCCATGCAGT-CCGTGCG-TGCCGCCTGCG

Ale_MJ1_Clone2 TCAAAATCTGGGACGAAATGCCCATGCAGT-CCGTGCG-TGCCGCCTGCG

Ape_MJ1_Clone1 TCAAAATCTGGGACGAAATGCCCATGCAGT-CCGTGCG-TGCCGCCTGCG

Ape_MJ1_Clone3 TCAAAATCTGGGACGAAATGCCCATGCAGT-CCGTGCG-TGCCGCCTGCG

CONTIG_574 TCAAAATCT----CAAA---------------------------------

CONTIG_7401 TCAAAATCTGGGACGAAATGCCCATGCAGT-CCGTGCG-TGCCGCTTGCG

CONTIG_10225 ACGCGTTCGAGAAACGTTTGAAGCTCGTTAAGG-----------------

CONTIG_29853 ACGCGTTCGAGAAACGTTTGAAGCTCGTTAAGG-----------------

CONTIG_11920 ACGCGTTCGAGAAACGTTTGAAGCTCGTTAAGG-----------------

CONTIG_8991 ACGCGTTCGAGAAACGTTTGAAGCTCGTTAAGGTGATTATAGAACGAAGC

CONTIG_23766 ACGCGTTCGAGAAACGTTTGAAGCTCGTTAAGG-----------------

CONTIG_13910 ACGCGTTCGAGAAACGTTTGAAGCTCGTTAAGG-----------------

CONTIG_4960 ACGCGTTCGAGAAACGTTTGAAGCTCGTTAAGG-----------------

Ape_MJ1_Clone6 ACGCGTTCGAGAAACGTTTGAAGCTCGTTAAGG-----------------

Ape_MJ1_Clone7 ACGCGTTCGAGAAACGTTTGAAGCTCGTTAAGG-----------------

Ape_MJ1_Clone2 ACGCGTTCGAGAAACGTTTGAAGCTCGTTAAGG-----------------

Ale_MJ1_Clone1 ACGCGTTCGAGAAACGTTTGAAGCTCGTTAAGG-----------------

Ale_MJ1_Clone11 ACGCGTTCGAGAAACGTTTGAAGCTCGTTAAGG-----------------

Ahy_MJ1_Clone1 ACGCGTTCGAGAAACGTTTGAAGCTCGTTAAGG-----------------

Ahy_MJ1_Clone2 ACGCGTTCGAGAAACGTTTGAAGCTCGTTAAGG-----------------

Ale_MJ1_Clone8 ACGCGTTCGAGAAACGTTTGAAGCTCGTTAAGG-----------------

Acr_MJ1_Clone2 ACGCGTTCGAGAAACATTTGAAGCTCGTTAAGG-----------------

Acr_MJ1_Clone3 ACGCGTTCGAGAAACATTTGAAGCTCGTTAAGG-----------------

Acr_MJ1_Clone1 ACGCGTTCGAGAAACGTTTGAAGCTCGTTAAGG-----------------

Ape_MJ1_Clone4 ACGCGTTCGAGAAACGTTTGAAGCTCGTTAAGG-----------------

Ape_MJ1_Clone5 ACGCGTTCGAGAAACGTTTGAAGCTCGTTAAGG-----------------

Ahy_MJ1_Clone3 ACGCGTTCGAGAAACGTTTGAAGCTCGTTAAGG-----------------

Ale_MJ1_Clone7 ACGCGTTCGAGAAACGTTTGAAGCTCGTTAAGG-----------------

Aju_MJ1_Clone3 ACGCGTTCGAGAAACGTTTGAAGCTCGTTAAGG-----------------

Aya_MJ1_Clone2 ACGCGTTCGAGAAACGTTTGAAGCTCGTTAAGG-----------------

Asi_MJ1_Clone2 ACGCGTTCGAGAAACGTTTGAAGCTCGTTAAGG-----------------

Asi_MJ1_Clone7 ACGCGTTCGAGAAACGTTTGAAGCTCGTTAAGG-----------------

Asi_MJ1_Clone1 ACGCGTTCGAGAAACGTTTGAAGCTCGTTAAGG-----------------

Asi_MJ1_Clone3 ACGCGTTCGAGAAACGTTTGAAGCTCGTTAAGG-----------------

Asi_MJ1_Clone4 ACGCGTTCGAGAAACGTTTGAAGCTCGTTAAGG-----------------

Asi_MJ1_Clone6 ACGCGTTCGAGAAACGTTTGAAGCTCGTTAAGG-----------------

Akl_MJ1_Clone3 ACGCGTTCGAGAAACGTTTGAAGCTCGTTAAGG-----------------

Aba_MJ1_Clone2 ACGCGTTCGAGAAACGTTTGAAGCTCGTTAAGG-----------------

Akw_MJ1_Clone3 ACGCGTTCGAGAAACGTTTGAAGCTCGTTAAGG-----------------

Aya_MJ1_Clone3 ACGCGTTCGAGAAACGTTTGAAGCTCGTTAAGG-----------------

Aju_MJ1_Clone1 ACGCGTTCGAGAAACGTTTGAAGCTCGTTAAGG-----------------

Asi_MJ1_Clone5 ACGCGTTCGAGAAACGTTTGAAGCTCGTTAAGG-----------------

Asi_MJ1_Clone9 ACGCGTTCGAGAAACGTTTGAAGCTCGTTAAGG-----------------

Akw_MJ1_Clone2 ACGCGTTCGAGAAACGTTTGAAGCTCGTTAAGG-----------------

Asi_MJ1_Clone8 ACGCGTTCGAGAAACGTTTGAAGCTCGTTAAGG-----------------

Akl_MJ1_Clone2 ACGCGTTCGAGAAACGTTTGAAGCTCGTTAAGG-----------------

Aya_MJ1_Clone1 ACGCGTTCGAGAAACGTTTGAAGCTCGTTAAGG-----------------

Akl_MJ1_Clone1 ACGCGTTCGAGAAACGTTTGAAGCTCGTTAAGG-----------------

Aba_MJ1_Clone3 ACGCGTTCGAGAAACGTTTGAAGCTCGTTAAGG-----------------

Aba_MJ1_Clone1 ACGCGTTCGAGAAACGTTTGAAGCTCGTTAAGG-----------------

Akw_MJ1_Clone1 ACGCGTTCGAGAAACGTTTGAAGCTCGTTAAGG-----------------

Aju_MJ1_Clone2 ACGCGTTCGAGAAACGTTTGAAGCTCGTTAAGG-----------------

Aju_MJ1_Clone7 ACGCGTTCGAGAAACGTTTGAAGCTCGTTAAGG-----------------

Ale_MJ1_Clone4 ACGCGTTCGAGAAACGTTTGAAGCTCGTTAAGG-----------------

Ale_MJ1_Clone10 ACGCGTTCGAGAAACGTTTGAAGCTCGTTAAGG-----------------

Aju_MJ1_Clone5 ACGCGTTCGAGAAACGTTTGAAGCTCGTTAAGG-----------------

Ale_MJ1_Clone5 ACGCGTTCGAGAAACGTTTGAAGCTCGTTAAGG-----------------

Aju_MJ1_Clone6 ACGCGTTCGAGAAACGTTTGAAGCTCGTTAAGG-----------------

Ale_MJ1_Clone3 ACGCGTTCGAGAAACGTTTGAAGCTCGTTAAGG-----------------

Ale_MJ1_Clone12 ACGCGTTCGAGAAACGTTTGAAGCTCGTTAAGG-----------------

Aju_MJ1_Clone4 ACGCGTTCGAGAAACGTTTGAAGCTCGTTAAGG-----------------

Aju_MJ1_Clone8 ACGCGTTCGAGAAACGTTTGAAGCTCGTTAAGG-----------------

Ale_MJ1_Clone9 ACGCGTTCGAGAAACGTTTGAAGCTCGTTAAGG-----------------

Ale_MJ1_Clone6 ACGCGTTCGAGAAACGTTTGAAGCTCGTTAAGG-----------------

Ale_MJ1_Clone13 ACGCGTTCGAGAAACGTTTGAAGCTCGTTAAGG-----------------

Ale_MJ1_Clone2 ACGCGTTCGAGAAACGTTTGAAGCTCGTTAAGG-----------------

Ape_MJ1_Clone1 ACGCGTTCGAGAAACGTTTGAAGCTCGTTAAGG-----------------

Ape_MJ1_Clone3 ACGCGTTTGAGAAACGTTTGAAGCTCGTTAAGG-----------------

CONTIG_574 -----TTCGAGAAACGTTTGAAGCTCGTTAAGG-----------------

CONTIG_7401 ACGCGTTCGAGAAACGTTTGAAGCTCGTTAAGG-----------------

CONTIG_10225 --------------------------------------------------

CONTIG_29853 --------------------------------------------------

CONTIG_11920 --------------------------------------------------

CONTIG_8991 CATACCTCAAATTTTGAAGAGCACAAGACTTGAGAACCAAACAGCGCTTC

CONTIG_23766 --------------------------------------------------

CONTIG_13910 --------------------------------------------------

CONTIG_4960 --------------------------------------------------

Ape_MJ1_Clone6 --------------------------------------------------

Ape_MJ1_Clone7 --------------------------------------------------

Ape_MJ1_Clone2 --------------------------------------------------

Ale_MJ1_Clone1 --------------------------------------------------

Ale_MJ1_Clone11 --------------------------------------------------

Ahy_MJ1_Clone1 --------------------------------------------------

Ahy_MJ1_Clone2 --------------------------------------------------

Ale_MJ1_Clone8 --------------------------------------------------

Acr_MJ1_Clone2 --------------------------------------------------

Acr_MJ1_Clone3 --------------------------------------------------

Acr_MJ1_Clone1 --------------------------------------------------

Ape_MJ1_Clone4 --------------------------------------------------

Ape_MJ1_Clone5 --------------------------------------------------

Ahy_MJ1_Clone3 --------------------------------------------------

Ale_MJ1_Clone7 --------------------------------------------------

Aju_MJ1_Clone3 --------------------------------------------------

Aya_MJ1_Clone2 --------------------------------------------------

Asi_MJ1_Clone2 --------------------------------------------------

Asi_MJ1_Clone7 --------------------------------------------------

Asi_MJ1_Clone1 --------------------------------------------------

Asi_MJ1_Clone3 --------------------------------------------------

Asi_MJ1_Clone4 --------------------------------------------------

Asi_MJ1_Clone6 --------------------------------------------------

Akl_MJ1_Clone3 --------------------------------------------------

Aba_MJ1_Clone2 --------------------------------------------------

Akw_MJ1_Clone3 --------------------------------------------------

Aya_MJ1_Clone3 --------------------------------------------------

Aju_MJ1_Clone1 --------------------------------------------------

Asi_MJ1_Clone5 --------------------------------------------------

Asi_MJ1_Clone9 --------------------------------------------------

Akw_MJ1_Clone2 --------------------------------------------------

Asi_MJ1_Clone8 --------------------------------------------------

Akl_MJ1_Clone2 --------------------------------------------------

Aya_MJ1_Clone1 --------------------------------------------------

Akl_MJ1_Clone1 --------------------------------------------------

Aba_MJ1_Clone3 --------------------------------------------------

Aba_MJ1_Clone1 --------------------------------------------------

Akw_MJ1_Clone1 --------------------------------------------------

Aju_MJ1_Clone2 --------------------------------------------------

Aju_MJ1_Clone7 --------------------------------------------------

Ale_MJ1_Clone4 --------------------------------------------------

Ale_MJ1_Clone10 --------------------------------------------------

Aju_MJ1_Clone5 --------------------------------------------------

Ale_MJ1_Clone5 --------------------------------------------------

Aju_MJ1_Clone6 --------------------------------------------------

Ale_MJ1_Clone3 --------------------------------------------------

Ale_MJ1_Clone12 --------------------------------------------------

Aju_MJ1_Clone4 --------------------------------------------------

Aju_MJ1_Clone8 --------------------------------------------------

Ale_MJ1_Clone9 --------------------------------------------------

Ale_MJ1_Clone6 --------------------------------------------------

Ale_MJ1_Clone13 --------------------------------------------------

Ale_MJ1_Clone2 --------------------------------------------------

Ape_MJ1_Clone1 --------------------------------------------------

Ape_MJ1_Clone3 --------------------------------------------------

CONTIG_574 --------------------------------------------------

CONTIG_7401 --------------------------------------------------

CONTIG_10225 --------------------------------------------------

CONTIG_29853 --------------------------------------------------

CONTIG_11920 --------------------------------------------------

CONTIG_8991 GTGTTGAAAATCTATCCCATTGGTCACCACCAGCAAGCAAGCAATTTGAT

CONTIG_23766 --------------------------------------------------

CONTIG_13910 --------------------------------------------------

CONTIG_4960 --------------------------------------------------

Ape_MJ1_Clone6 --------------------------------------------------

Ape_MJ1_Clone7 --------------------------------------------------

Ape_MJ1_Clone2 --------------------------------------------------

Ale_MJ1_Clone1 --------------------------------------------------

Ale_MJ1_Clone11 --------------------------------------------------

Ahy_MJ1_Clone1 --------------------------------------------------

Ahy_MJ1_Clone2 --------------------------------------------------

Ale_MJ1_Clone8 --------------------------------------------------

Acr_MJ1_Clone2 --------------------------------------------------

Acr_MJ1_Clone3 --------------------------------------------------

Acr_MJ1_Clone1 --------------------------------------------------

Ape_MJ1_Clone4 --------------------------------------------------

Ape_MJ1_Clone5 --------------------------------------------------

Ahy_MJ1_Clone3 --------------------------------------------------

Ale_MJ1_Clone7 --------------------------------------------------

Aju_MJ1_Clone3 --------------------------------------------------

Aya_MJ1_Clone2 --------------------------------------------------

Asi_MJ1_Clone2 --------------------------------------------------

Asi_MJ1_Clone7 --------------------------------------------------

Asi_MJ1_Clone1 --------------------------------------------------

Asi_MJ1_Clone3 --------------------------------------------------

Asi_MJ1_Clone4 --------------------------------------------------

Asi_MJ1_Clone6 --------------------------------------------------

Akl_MJ1_Clone3 --------------------------------------------------

Aba_MJ1_Clone2 --------------------------------------------------

Akw_MJ1_Clone3 --------------------------------------------------

Aya_MJ1_Clone3 --------------------------------------------------

Aju_MJ1_Clone1 --------------------------------------------------

Asi_MJ1_Clone5 --------------------------------------------------

Asi_MJ1_Clone9 --------------------------------------------------

Akw_MJ1_Clone2 --------------------------------------------------

Asi_MJ1_Clone8 --------------------------------------------------

Akl_MJ1_Clone2 --------------------------------------------------

Aya_MJ1_Clone1 --------------------------------------------------

Akl_MJ1_Clone1 --------------------------------------------------

Aba_MJ1_Clone3 --------------------------------------------------

Aba_MJ1_Clone1 --------------------------------------------------

Akw_MJ1_Clone1 --------------------------------------------------

Aju_MJ1_Clone2 --------------------------------------------------

Aju_MJ1_Clone7 --------------------------------------------------

Ale_MJ1_Clone4 --------------------------------------------------

Ale_MJ1_Clone10 --------------------------------------------------

Aju_MJ1_Clone5 --------------------------------------------------

Ale_MJ1_Clone5 --------------------------------------------------

Aju_MJ1_Clone6 --------------------------------------------------

Ale_MJ1_Clone3 --------------------------------------------------

Ale_MJ1_Clone12 --------------------------------------------------

Aju_MJ1_Clone4 --------------------------------------------------

Aju_MJ1_Clone8 --------------------------------------------------

Ale_MJ1_Clone9 --------------------------------------------------

Ale_MJ1_Clone6 --------------------------------------------------

Ale_MJ1_Clone13 --------------------------------------------------

Ale_MJ1_Clone2 --------------------------------------------------

Ape_MJ1_Clone1 --------------------------------------------------

Ape_MJ1_Clone3 --------------------------------------------------

CONTIG_574 --------------------------------------------------

CONTIG_7401 --------------------------------------------------

CONTIG_10225 --------------------------------------------------

CONTIG_29853 --------------------------------------------------

CONTIG_11920 --------------------------------------------------

CONTIG_8991 TGGTTTTCAACGCGAACTGTTGTCAGATTCTCCAGTCTTGTGCACTTGAA

CONTIG_23766 --------------------------------------------------

CONTIG_13910 --------------------------------------------------

CONTIG_4960 --------------------------------------------------

Ape_MJ1_Clone6 --------------------------------------------------

Ape_MJ1_Clone7 --------------------------------------------------

Ape_MJ1_Clone2 --------------------------------------------------

Ale_MJ1_Clone1 --------------------------------------------------

Ale_MJ1_Clone11 --------------------------------------------------

Ahy_MJ1_Clone1 --------------------------------------------------

Ahy_MJ1_Clone2 --------------------------------------------------

Ale_MJ1_Clone8 --------------------------------------------------

Acr_MJ1_Clone2 --------------------------------------------------

Acr_MJ1_Clone3 --------------------------------------------------

Acr_MJ1_Clone1 --------------------------------------------------

Ape_MJ1_Clone4 --------------------------------------------------

Ape_MJ1_Clone5 --------------------------------------------------

Ahy_MJ1_Clone3 --------------------------------------------------

Ale_MJ1_Clone7 --------------------------------------------------

Aju_MJ1_Clone3 --------------------------------------------------

Aya_MJ1_Clone2 --------------------------------------------------

Asi_MJ1_Clone2 --------------------------------------------------

Asi_MJ1_Clone7 --------------------------------------------------

Asi_MJ1_Clone1 --------------------------------------------------

Asi_MJ1_Clone3 --------------------------------------------------

Asi_MJ1_Clone4 --------------------------------------------------

Asi_MJ1_Clone6 --------------------------------------------------

Akl_MJ1_Clone3 --------------------------------------------------

Aba_MJ1_Clone2 --------------------------------------------------

Akw_MJ1_Clone3 --------------------------------------------------

Aya_MJ1_Clone3 --------------------------------------------------

Aju_MJ1_Clone1 --------------------------------------------------

Asi_MJ1_Clone5 --------------------------------------------------

Asi_MJ1_Clone9 --------------------------------------------------

Akw_MJ1_Clone2 --------------------------------------------------

Asi_MJ1_Clone8 --------------------------------------------------

Akl_MJ1_Clone2 --------------------------------------------------

Aya_MJ1_Clone1 --------------------------------------------------

Akl_MJ1_Clone1 --------------------------------------------------

Aba_MJ1_Clone3 --------------------------------------------------

Aba_MJ1_Clone1 --------------------------------------------------

Akw_MJ1_Clone1 --------------------------------------------------

Aju_MJ1_Clone2 --------------------------------------------------

Aju_MJ1_Clone7 --------------------------------------------------

Ale_MJ1_Clone4 --------------------------------------------------

Ale_MJ1_Clone10 --------------------------------------------------

Aju_MJ1_Clone5 --------------------------------------------------

Ale_MJ1_Clone5 --------------------------------------------------

Aju_MJ1_Clone6 --------------------------------------------------

Ale_MJ1_Clone3 --------------------------------------------------

Ale_MJ1_Clone12 --------------------------------------------------

Aju_MJ1_Clone4 --------------------------------------------------

Aju_MJ1_Clone8 --------------------------------------------------

Ale_MJ1_Clone9 --------------------------------------------------

Ale_MJ1_Clone6 --------------------------------------------------

Ale_MJ1_Clone13 --------------------------------------------------

Ale_MJ1_Clone2 --------------------------------------------------

Ape_MJ1_Clone1 --------------------------------------------------

Ape_MJ1_Clone3 --------------------------------------------------

CONTIG_574 --------------------------------------------------

CONTIG_7401 --------------------------------------------------

CONTIG_10225 --------------------------------------AGTACAA-----

CONTIG_29853 --------------------------------------AGTACAA-----

CONTIG_11920 --------------------------------------AGTACAA-----

CONTIG_8991 AATTCCAAGTTTGGCTTCGTTTTATAATCACCTTAAGGAGTACAA-----

CONTIG_23766 --------------------------------------AGTACAA-----

CONTIG_13910 --------------------------------------AGTACAA-----

CONTIG_4960 --------------------------------------AGTACAA-----

Ape_MJ1_Clone6 --------------------------------------AGTACAA-----

Ape_MJ1_Clone7 --------------------------------------AGTACAA-----

Ape_MJ1_Clone2 --------------------------------------AGTACAA-----

Ale_MJ1_Clone1 --------------------------------------AGTACAA-----

Ale_MJ1_Clone11 --------------------------------------AGTACAA-----

Ahy_MJ1_Clone1 --------------------------------------AGTACAA-----

Ahy_MJ1_Clone2 --------------------------------------AGTACAA-----

Ale_MJ1_Clone8 --------------------------------------AGTACAA-----

Acr_MJ1_Clone2 --------------------------------------AGTACAA-----

Acr_MJ1_Clone3 --------------------------------------AGTACAA-----

Acr_MJ1_Clone1 --------------------------------------AGTACAA-----

Ape_MJ1_Clone4 --------------------------------------AGTACAA-----

Ape_MJ1_Clone5 --------------------------------------AGTACAA-----

Ahy_MJ1_Clone3 --------------------------------------AGTACAA-----

Ale_MJ1_Clone7 --------------------------------------AGTACAA-----

Aju_MJ1_Clone3 --------------------------------------AGTACAA-----

Aya_MJ1_Clone2 --------------------------------------AGTACAA-----

Asi_MJ1_Clone2 --------------------------------------AGTACAA-----

Asi_MJ1_Clone7 --------------------------------------AGTACAA-----

Asi_MJ1_Clone1 --------------------------------------AGTACAA-----

Asi_MJ1_Clone3 --------------------------------------AGTACAA-----

Asi_MJ1_Clone4 --------------------------------------AGTACAA-----

Asi_MJ1_Clone6 --------------------------------------AGTACAA-----

Akl_MJ1_Clone3 --------------------------------------AGTACAA-----

Aba_MJ1_Clone2 --------------------------------------AGTACAA-----

Akw_MJ1_Clone3 --------------------------------------AGTACAA-----

Aya_MJ1_Clone3 --------------------------------------AGTACAA-----

Aju_MJ1_Clone1 --------------------------------------AGTACAA-----

Asi_MJ1_Clone5 --------------------------------------AGTACAA-----

Asi_MJ1_Clone9 --------------------------------------AGTACAA-----

Akw_MJ1_Clone2 --------------------------------------AGTACAA-----

Asi_MJ1_Clone8 --------------------------------------AGTACAA-----

Akl_MJ1_Clone2 --------------------------------------AGTACAA-----

Aya_MJ1_Clone1 --------------------------------------AGTACAA-----

Akl_MJ1_Clone1 --------------------------------------AGTACAA-----

Aba_MJ1_Clone3 --------------------------------------AGTACAA-----

Aba_MJ1_Clone1 --------------------------------------AGTACAA-----

Akw_MJ1_Clone1 --------------------------------------AGTACAA-----

Aju_MJ1_Clone2 --------------------------------------AGTACAA-----

Aju_MJ1_Clone7 --------------------------------------AGTACAA-----

Ale_MJ1_Clone4 --------------------------------------AGTACAA-----

Ale_MJ1_Clone10 --------------------------------------AGTACAA-----

Aju_MJ1_Clone5 --------------------------------------AGTACAA-----

Ale_MJ1_Clone5 --------------------------------------AGTACAAAGGAG

Aju_MJ1_Clone6 --------------------------------------AGTACAA-----

Ale_MJ1_Clone3 --------------------------------------AGTACAA-----

Ale_MJ1_Clone12 --------------------------------------AGTACAA-----

Aju_MJ1_Clone4 --------------------------------------AGTACAA-----

Aju_MJ1_Clone8 --------------------------------------AGTACAA-----

Ale_MJ1_Clone9 --------------------------------------AGTACAA-----

Ale_MJ1_Clone6 --------------------------------------AGTACAA-----

Ale_MJ1_Clone13 --------------------------------------AGTACAA-----

Ale_MJ1_Clone2 --------------------------------------AGTACAA-----

Ape_MJ1_Clone1 --------------------------------------AGTACAA-----

Ape_MJ1_Clone3 --------------------------------------AGTACAA-----

CONTIG_574 --------------------------------------AGTACAA-----

CONTIG_7401 --------------------------------------AGTACAA-----

CONTIG_10225 -------AGGGGGGG----TCATTCCAAGAGAAATGTTGTAAACGTTCCT

CONTIG_29853 -------AGGGGGGG----TCATTCCAAGAGAAATGTTGTAAACGTTCCT

CONTIG_11920 -------AGGGGGGG----TCATTCCAAGAGAAATGTTGTAAACGTTCCT

CONTIG_8991 -------AGGGGGGGG---TCATACCAAGATAAATGTTGTAAACGTTCCT

CONTIG_23766 -------AGGGGGGGG---TCATTCCAAGAGAAATGTTGTAAACGTTCCT

CONTIG_13910 -------AGGAGGGG----TCATTCCAAGAGAAATGTTGTAAACGTTCCT

CONTIG_4960 -------AGGGGGGGGGGGTCATTCCAAGAGAAATGTTGTGAACGTTCCT

Ape_MJ1_Clone6 -------AGGGGGGG----TCATTCCAAGAGAAATGTTGTAAACGTTCCT

Ape_MJ1_Clone7 -------AGGGGGGG----TCATTCCAAGAGAAATGTTGTAAACGTTCCT

Ape_MJ1_Clone2 -------AGGGGGGGG---TCATTCCAAGAGAAATGTTGTAAACGTTCCT

Ale_MJ1_Clone1 -------AGGGGGGG----TCATCCCAAGAGAAATGTTGTAAACGTTCCT

Ale_MJ1_Clone11 -------AGGGGGGG----TCATTCCAAGAGAAATGTTGTAAACGTTCCT

Ahy_MJ1_Clone1 -------AGGGGGGG----TCATTCCAAGAGAAATGTTGTAAACGTTCCT

Ahy_MJ1_Clone2 -------AGGGGGGG----TCATTCCAAGAGAAATGTTGTAAACGTTCCT

Ale_MJ1_Clone8 -------AGGGGGGG----TCATTCCAAGAGAAATGTTGTAAACGTTCCT

Acr_MJ1_Clone2 -------AGGGGGGG----TCATTCCAAGAGAAATGTTGTAAACGTTCCT

Acr_MJ1_Clone3 -------AGGGGGGG----TCATTCCAAGAGAAATGTTGTAAACGTTCCT

Acr_MJ1_Clone1 -------AGGGGGGGGGG-TCATTCCAAGAGAAATGTTGTAAACGTTCCT

Ape_MJ1_Clone4 -------AGGGGGG-----TCATTCCAAGAGAAATGTTGTAAACGTTCCT

Ape_MJ1_Clone5 -------AGGGGGG-----TCATTCCAAGAGAAATGTTGTAAACGTTCCT

Ahy_MJ1_Clone3 -------AGGGGGGG----TCATTCCAAGAGAAATGTTGTAAACGTTCCT

Ale_MJ1_Clone7 -------AGGGGGGG----TCATTCCAAGAGAAATGTTGTAAACGTTCCT

Aju_MJ1_Clone3 -------AGGGGGGG----TCATTCCAAGAGAAATGTTGTAAACGTTCCT

Aya_MJ1_Clone2 -------AGGGGGGG----TCATTCCAAGAGAAATGTTGTAAACGTTCCT

Asi_MJ1_Clone2 -------AGGGGGGG----TCATTCCAAGAGAAATGTTGTAAACGTTCCT

Asi_MJ1_Clone7 -------AGGGGGGG----TCATTCCAAGAGAAATGTTGTAAACGTTCCT

Asi_MJ1_Clone1 -------AGGGGGGG----TCATTCCAAGAGAAATGTTGTAAACGTTCCT

Asi_MJ1_Clone3 -------AGGGGGGG----TCATTCCAAGAGAAATGTTGTAAACGTTCCT

Asi_MJ1_Clone4 -------AGGGGGGG----TCATTCCAAGAGAAATGTTGTAAACGTTCCT

Asi_MJ1_Clone6 -------AGGGGGGG----TCATTCCAAGAGAAATGTTGTAAACGTTCCT

Akl_MJ1_Clone3 -------AGGGGGGG----TCATTCCAAGAGAAATGTTGTAAACGTTCCT

Aba_MJ1_Clone2 -------AGGGGGGG------AATCCAAGAGAAATGTTGTAAACGTTCCT

Akw_MJ1_Clone3 -------AGGGGGGG----TCATTCCAAGAGAAATGTTGTAAACGTTCCT

Aya_MJ1_Clone3 -------AGGGGGGG----TCATTCCAAGAGAAATGTTGTAAACGTTCCT

Aju_MJ1_Clone1 -------AGGGGGGG----TCATTCCAAGAGAAATGTTGTAAACGTTCCT

Asi_MJ1_Clone5 -------AGGGGGG-----TCATTCCAAGAGAAATGTTGTAAACGTTCCT

Asi_MJ1_Clone9 -------AGGGGGGG----TCATTCCAAGAGAAATGTTGTAAACGTTCCT

Akw_MJ1_Clone2 -------AGGGGGGG----TCATTCCAAGAGAAATGTTGTAAACGTTCCT

Asi_MJ1_Clone8 -------AGGGGGGG----TCATTCCAAGAGAAATGTTGTAAACGTTCCT

Akl_MJ1_Clone2 -------AGGGGGGG----TCATTCCAAGAGAAATGTTGTAAACGTTCCT

Aya_MJ1_Clone1 -------AGGGGGGG----TCATTCCAAGAGAAATGTTGTAAACGTTCCT

Akl_MJ1_Clone1 -------AGGGGGGG----TCATTCCAAGAGAAATGTTGTAAACGTTCCT

Aba_MJ1_Clone3 -------AGGGGGGG----TCATTCCAAGAGAAATGTTGTAAACGTTCCT

Aba_MJ1_Clone1 -------AGGGGGGG----TCATTCCAAGAGAAATGTTGTAAACGTTCCT

Akw_MJ1_Clone1 -------AGGGGGGG----TCATTCCAAGAGAAATGTTGTAAACGTTCCT

Aju_MJ1_Clone2 -------AGGGGGGG----TCATTCCAAGAGAAATGTTGTAAACGTTCCT

Aju_MJ1_Clone7 -------AGGGGGGG----TCATTCCAAGAGAAATGTTGTAAACGTTCCT

Ale_MJ1_Clone4 -------AGGGGGGG----TCATTCCAAGAGAAATGTTGTAAACGTTCCT

Ale_MJ1_Clone10 -------AGGGGGGG----TCATTCCAAGAGAAATGTTGTAAACGTTCCT

Aju_MJ1_Clone5 -------AGGGGGGG----TCATTCCAAGAGAAATGTTGTAAACGTTCCT

Ale_MJ1_Clone5 GGGGGGGGGGGGGGG----TCATTCCAAGAGAAATGTTGTAAACGTTCCT

Aju_MJ1_Clone6 -------GGGGGGGG----TCATTCCAAGAGAAATGTTGTAAACGTTCCT

Ale_MJ1_Clone3 -------AGGGGGGG----TCATTCCAAGAGAAATGTTGTAAACGTTCCT

Ale_MJ1_Clone12 -------AGGGGGGG----TCATTCCAAGAGAAATGTTGTAAACGTTCCT

Aju_MJ1_Clone4 -------AGGGGGGG----TCATTCCAAGAGAAATGTTGTAAACGTTCCT

Aju_MJ1_Clone8 -------AGGGGGGG----TCATTCCAAGAGAAATGTTGTAAACGTTCCT

Ale_MJ1_Clone9 -------AGGGGGGG----TCATTCCAAGAGAAATGTTGTAAACGTTCCT

Ale_MJ1_Clone6 -------AGGGGGGG----TCATTCCAAGAGAAATGTTGTAAACGTTCCT

Ale_MJ1_Clone13 -------AGGGGGGG----TCATTCCAAGAGAAATGTTGTAAACGTTCCT

Ale_MJ1_Clone2 -------AGGGGGGG----TCATTCCAAGAGAAATGTTGTAAACGTTCCT

Ape_MJ1_Clone1 -------AGGGGGG-----TCATTCCAAGAGAAATGTTGTAAACGTTCCT

Ape_MJ1_Clone3 -------AGGGGGGG----TCATTCCAAGAGAAATGTTGTAAACGTTCCT

CONTIG_574 -------AGGGGGGGG---TCATTCCAAGAGAAATGTTGTAAACGTTCCT

CONTIG_7401 -------AGGGGGGG----TCATTCCAAGAGAAATGTTGTAAACGTTCCT

CONTIG_10225 TGTAAACATAGCTTTCAATAACATAAATCCAAAAAATAAAAAAA--CATG

CONTIG_29853 TGTAAACATAGCTTTCAATAACATAAATCCAAAAAATAAAAAAA--CATG

CONTIG_11920 TG------------------------------------------------

CONTIG_8991 TGTAAACATAGCTTTCAATAACATAAATCCAAAAAATAAAAAAA--CATG

CONTIG_23766 TGTAAACATAGCTTTCAATAACATAATTCCAAAAAATAAAAAAAA-CATG

CONTIG_13910 TGTAAACATAGCTTTCAATAACATAAATCCAAAAAATAAAAAAA--CATG

CONTIG_4960 TGTAAACATAGCTTTCAATAACATAAATCCAAAAAATAAAAAAA--CATG

Ape_MJ1_Clone6 TATAAACATAGCTTTCAACAACATAAATCCAAAAAATAAAAAAAA-CATG

Ape_MJ1_Clone7 TATAAACATAGCTTTCAACAACATAAATCCAAAAAATAAAAAAAA-CATG

Ape_MJ1_Clone2 TGTAAACATAGCTTTCAACAACATAAATCCAAAAAATAAAAAAAA-CATG

Ale_MJ1_Clone1 TGTAAACATAGCTTTCAATAACTTAAATCCAAAAAATAAAAAAAAACATG

Ale_MJ1_Clone11 TGTAAACATAGCTTTCAATAACTTAAATCCAAAAAATAAAAAAAAACATG

Ahy_MJ1_Clone1 TGTAAACATAGCTTTCAATAACTTAAATCCAAAAAATAAAAAAAA-CATG

Ahy_MJ1_Clone2 TGTAAACATAGCTTTCAATAACTTAAATCCAAAAAATAAAAAAAA-CATG

Ale_MJ1_Clone8 TGTAAACATAGATTTCAATAACTTAAATCCAAAAAATAAAAAAAA-CATG

Acr_MJ1_Clone2 TGTAAACATAGCTTTCAATAACATAAATCCAAAAAATAAAAAAAA-CATG

Acr_MJ1_Clone3 TGTAAACATAGCTTTCAATAACATAAATCCAAAAAATAAAAAAAA-CATG

Acr_MJ1_Clone1 TGTAAACATAGCTTTCAATAACATAAATCCAAAAAATAAAAAAA--CATG

Ape_MJ1_Clone4 TGTAAACATAGCTTTCAATACCATAAATCCAAAAAATAAAAAAA--CATG

Ape_MJ1_Clone5 TGTAAACATAGCTTTCAATACCATAAATCCAAAAAATAAAAAAA--CATG

Ahy_MJ1_Clone3 TGTAAACATAGCTTTCAATAACTTAAATCCAAAAAATAAAAAAAA-CATG

Ale_MJ1_Clone7 TGTAAACATAGCTTTCAATAACTTAAATCCAAAAAATAAAAAAAA-CATG

Aju_MJ1_Clone3 TGTAAACATAGCTTTCAATAACTTAAATCCAAAAAATAAAAAAAA-CATG

Aya_MJ1_Clone2 TGTAAACATAGCTTTCAATAACTTAAATCCAAAAAATAAAAAAAA-CATG

Asi_MJ1_Clone2 TGTAAACATAGCTTTCAATAACTTAAATCCAAAAAATAAAAAAAA-CATG

Asi_MJ1_Clone7 TGTAAACATGGCTTTCAATAACTTAAATCCAAAAAATAAAAAAAA-CATG

Asi_MJ1_Clone1 TGTAAACATAGCTTTCAATAACTTAAATCCAAAAAATAAAAAAAA-CATG

Asi_MJ1_Clone3 TGTAAACATAGCTTTCAATAACTTAAATCCAAAAAATAAAAAAAA-CATG

Asi_MJ1_Clone4 TGTAAACATAGCTTTCAATAACTTAAATCCAAAAAATAAAAAAAA-CATG

Asi_MJ1_Clone6 TGTAAACATAGCTTTCAATAACTTAAATCCAAAAAATAAAAAAAA-CATG

Akl_MJ1_Clone3 TGTAAACATAGCTTTCAAAAACTTAAATCCAAAAAATAAAAAAAA-CATG

Aba_MJ1_Clone2 TGTAAACATAGCTTTCAATAACTTAAATCCAAAAAATAAAAAAAA-CATG

Akw_MJ1_Clone3 TGTAAACATAGCTTTCAATAACTTAAATCCAAAAAATAAAAAAAA-CATG

Aya_MJ1_Clone3 TGTAAACATAGCTTTCAATAACTTAAATCCAAAAAATAAAAAAAA-CATG

Aju_MJ1_Clone1 TGTAAACATAGCTTTCAATAACTTAAATCCAAAAAATAAAAAAAA-CATG

Asi_MJ1_Clone5 TGTAAACATAGCTTTCAATAACTTAAATCCAAAAAATAAAAAAAA-CATG

Asi_MJ1_Clone9 TGTAAACATAGCTTTCAATAACTTAAATCCAAAAAATAAAAAAAAACATG

Akw_MJ1_Clone2 TGTAAACATAGCTTTCAATAACTTAAATCCAAAAAATAAAAAAAA-CATG

Asi_MJ1_Clone8 TGTAAACATAGCTTTCAATAACTTAAATCCAAAAAATAAAAAAAA-CATG

Akl_MJ1_Clone2 TGTAAACATAGCTTTCAATAACTTAAATCCAAAAAATAAAAAAAA-CATG

Aya_MJ1_Clone1 TGTAAACATAGCTTTCAATAACTTAAATCCAAAAAATAAAAAAAA-CATG

Akl_MJ1_Clone1 TGTAAACATAGCTTTCAATAACTTAAATCCAAAAAATAAAAAAAA-CATG

Aba_MJ1_Clone3 TGTAAACATAGCTTTCAATAACTTAAATCCAAAAAATAAAAAAAA-CATG

Aba_MJ1_Clone1 TGTAAACATAGCTTTCAATAACTTAAATCCAAAAAATAAAAAAAA-CATG

Akw_MJ1_Clone1 TGTAAACATAGCTTTCAATAACTTAAATCCAAAAAATAAAAAAAA-CATG

Aju_MJ1_Clone2 TGTAAACATAGCTTTCAATAACT-------------TAAAAA-------G

Aju_MJ1_Clone7 TGTAAACATAGCTTTCAATAACTAAAATCCAAAAAATAAAAAAAC-CATG

Ale_MJ1_Clone4 TGTAAACATAGCTTTCAATAACTTAAATCCAAAAAATAAAAAAAA-CATG

Ale_MJ1_Clone10 TGTAAACATAGCTTTCAATAACTTAAATTCAAAAAATAAAAAAAA-CATG

Aju_MJ1_Clone5 TGTAAACATAGCTTTCAATAACTTAAATCCAAAAAATAAAAAAAA-CATG

Ale_MJ1_Clone5 TGTAAACATAGCTTTCAATAACTTAAATCCAAAAAATAAAAAAAA-CATG

Aju_MJ1_Clone6 TGTAAACATAGCTTTCAATAACTTAAATCCAAAAAATAAAAAAAA-CATG

Ale_MJ1_Clone3 TGTAAACATAGCTTTCAATAACTTAAATCCAAAAAATAAAAAAAA-CATG

Ale_MJ1_Clone12 TGTAAACATAGCTTTCAATAACTTAAATCCAAAAAATAAAAAAAA-CATG

Aju_MJ1_Clone4 TGTAAACATAGCTTTCAATAACTTAAATCCAAAAAATAAAAAAAA-CATG

Aju_MJ1_Clone8 TGTAAACATAGCTTTCAATAACTTAAATCCAAAAAATAAAAAAAA-CATG

Ale_MJ1_Clone9 TGTAAACATAGCTTTCAATAACTTAAATCCAAAAAATAAAAAAAA-CATG

Ale_MJ1_Clone6 TGTAAACATAGCTTTCAATAACTTAAATCCAAAAAATAAAAAAAA-CATG

Ale_MJ1_Clone13 TGTAAACATAGCTTTCAATAACTTAAATCCAAAAAATAAAAAAAA-CATG

Ale_MJ1_Clone2 TGTAAACATAGCTTTCAATAACTTAAATCCAAAAAATAAAAAAAA-CATG

Ape_MJ1_Clone1 TGTAAACATAGCTTTCAATAACTTAAATCCAAAAAATAAAAAAAA-CATG

Ape_MJ1_Clone3 TGTAAACATAGCTTTCAATAACTTAAATCCAAAAAATAAAAAAAA-CATG

CONTIG_574 TGTAAACATAGCTTTTAATAACATAAATCCAAAAAATAAAAAAAA-CATG

CONTIG_7401 TGTAAACATAGCTTTCAATAACATAAATCCAAAAAATAAAAAAA--CATG

CONTIG_10225 TTTTCATTTTTTT-AACAAATTTTGAAAGTGTATCCGAACTTATTGAACA

CONTIG_29853 TTTTCATTTTTTT-AACAAATTTTGAAAGTGTATCCGAACTTATTGAACA

CONTIG_11920 --------------------------------------------------

CONTIG_8991 TTTTCATTTTTTT-AACAAATTTTGAAAGTGTATCCGAACTAATTGAACA

CONTIG_23766 TTTTCATTTTTTTTAACAAATTTTGAAAGTGTATCCGAACTTACTGAACA

CONTIG_13910 TTTTCATTTTTTT-AACAAATTTTGAAAGTGTATCCGAACTTATTGAACA

CONTIG_4960 TTTTCATTTTTTT-AACAAATTTTGAAAGTGTATCCGAACTTATTGAACA

Ape_MJ1_Clone6 TTTTCATTTTTTT-AACAAATTTTGAAAGTGTATCCGAACTTATTGAACA

Ape_MJ1_Clone7 TTTTCATTTTTTT-AACAAATTTTGAAAGTGTATCCGAACTTATTGAACA

Ape_MJ1_Clone2 TTTTCATTTTTTT-AACAAATTTTGAAAGTGTATCCGAACTTATTGAACA

Ale_MJ1_Clone1 TTTTCATTTTTTT-AACAAATTTTGAAAGTGTATCCGAACTTATTGAACA

Ale_MJ1_Clone11 TTTTCATTTTTTT-AACAAATTTTGAAAGTGTACCCGAACTTATTGAACA

Ahy_MJ1_Clone1 TTTTCATTTTTTT-AACAAATTTTGAAAGTGTATCCGAACTTATTGAACA

Ahy_MJ1_Clone2 TTTTCATTTTTTT-AACAAATTTTGAAAGTGTATCCGAACTTATTGAACA

Ale_MJ1_Clone8 TTTTCATTTTTTT-AACAAATTTTGAAAGTGTATCCGAACTTATTGAACA

Acr_MJ1_Clone2 TTTTCATTTTTTT-AACAAATTTTGAAAGTGTATCCGAACTTATTGAACA

Acr_MJ1_Clone3 TTTTCATTTTTTT-AACAAATTTTGAAAGTGTATCCGAACTTATTGAACA

Acr_MJ1_Clone1 TTTTCATTTTTTT-AACAAATTTTGAAAGTGTATCCGAACTTATTGAACA

Ape_MJ1_Clone4 TTTTCATTTTTTT-AACAAATTTTGAAAGTGTATCCGAACTTATTGAACA

Ape_MJ1_Clone5 TTTTCATTTTTTT-AACAAATTTTGAAAGTGTATCCGAACTTATTGAACA

Ahy_MJ1_Clone3 TTTTCATTTTTTT-AACAAATTTTGAAAGTGTATCCGAACTTATTGAACA

Ale_MJ1_Clone7 TTTTCATTTTTTT-AACAAATTTTGAAAGTGTATCCGAACTTATTGAACA

Aju_MJ1_Clone3 TTTTCATTTTTTT-AACAAATTTTGAAAGTGTATCCGAACTTATTGAACA

Aya_MJ1_Clone2 TTTTCATTTTTTT-AACAAATTTTGAAAGTGTATCCGAACTTATTGAACA

Asi_MJ1_Clone2 TTTTCATTCTTTT-AACAAATTTTGAAAGTGTATCCGAACTTATTGAACA

Asi_MJ1_Clone7 TTTTCATTTTTTT-AACAAATTTTGAAAGTGTATCCGAACTTATTGAACA

Asi_MJ1_Clone1 TTTTCATTTTTTT-AACAAATTTTGAAAGTGTATCCGAACTTATTGAACA

Asi_MJ1_Clone3 TTTTCATTTTTTT-AACAAATTTTGAAAGTGTATCCGAACTTATTGAACA

Asi_MJ1_Clone4 TTTTCATTTTTTT-AACAAATTTTGAAAGTGTATCCGAACTTATTGAACA

Asi_MJ1_Clone6 TTTTCATTTTTTT-AACAAATTTTGAAAGTGTATCCGAACTTATTGAACA

Akl_MJ1_Clone3 TTTTCATTTTTTT-AACAAATTTTGAAAGTGTATCCGAACTTATTGAACA

Aba_MJ1_Clone2 TTTTCATTTTTTT-AACAAATTTTGAAAGTGTATCCGAACTTATTGAACA

Akw_MJ1_Clone3 TTTTCATTTTTTT-AACAAATTTTGAAAGTGTATCCGAACTTATTGAACA

Aya_MJ1_Clone3 TTTTCATTTTTTT-AACAAATTTTGAAAGTGTATCCGAACTTATTGAACA

Aju_MJ1_Clone1 TTTTCATTTTTTT-AACAAATTTTGAAAGTGTATCCGAACTTATTGAACA

Asi_MJ1_Clone5 TTTTCATTTTTTT-TACAAATTTTGAAAGTGTATCCGAACTTATTGAACA

Asi_MJ1_Clone9 TTTTCATTTTTTT-AACAAATTTTGAAAGTGTATCCGAACTTATTGAACA

Akw_MJ1_Clone2 TTTTCATTTTTTT-AACAAATTTTGAAAGTGTATCCGAACTTATTGAACA

Asi_MJ1_Clone8 TTTTCATTTTTTT-AACAAATTTTGAAAGTGTATCCGAACTTATTGAACA

Akl_MJ1_Clone2 TTTTCATTTTTTT-AACAAATTTTGAAAGTGTATCCGAACTTATTGAACA

Aya_MJ1_Clone1 TTTTCATTTTTTT-AACAAATTTTGAAAGTGTATCCGAACTTATTGAACA

Akl_MJ1_Clone1 TTTTCATTTTTTT-AACAAATTTTGAAAGTGTATCCGAACTTATTGAACA

Aba_MJ1_Clone3 TTTTCATTTTTTT-AACAAATTTTGAAAGTGTATCCGAACTTATTGAACA

Aba_MJ1_Clone1 TTTTCATTTTTTT-AACAAATTTTGAAAGTGTATCCGAACTTATTGAACA

Akw_MJ1_Clone1 TTTTCATTTTTTT-AACAAATTTTGAAAGTGTATCCGAACTTATTGAACA

Aju_MJ1_Clone2 TTATTGTGT---------------AAAAGTGTATCCGAACTTATTGAACA

Aju_MJ1_Clone7 TTTTCATTTTTTT-AACAAATTTTGAAAGTGTATCCGAACTTATTGAACA

Ale_MJ1_Clone4 TTTTCATTTTTTT-AACAAATTTTGAAAGTGTATCCGAACTTATTGAACA

Ale_MJ1_Clone10 TTTTCATTTTTTT-AACAAATTTTGAAAGTGTATCCGAACTTATTGAACA

Aju_MJ1_Clone5 TTTTCATTTTTTT-AACAAATTTTGAAAGTGTATCCGAACTTATTGAACA

Ale_MJ1_Clone5 TTTTCATTTTTTT-AACAAATTTTGAAAGTGTATCCGAACTTATTGAACA

Aju_MJ1_Clone6 TTTTCATTTTTTT-AACAAATTTTGAAAGTGTATCCGAACTTATTGAACA

Ale_MJ1_Clone3 TTTTCATTTTTTT-AACAAATTTTGAAAGTGTATCCGAACTTATTGAACA

Ale_MJ1_Clone12 TTTTCATTTTTTT-AACAAATTTTGAAAGTGTATCCGAACTTATTGAACA

Aju_MJ1_Clone4 TTTTCATTTTTTT-AACAAATTTTGAAAGTGTATCCGAACTTATTGAACA

Aju_MJ1_Clone8 TTTTCATTTTTTT-AACAAATTTTGAAAGTGTATCCGAACTTATTGAACA

Ale_MJ1_Clone9 TTTTCATTTTTTT-AACAAATTTTGAAAGTGTATCCGAACTTATTGAACA

Ale_MJ1_Clone6 TTTTCATTTTTTT-AACAAATTTTGAAAGTGTATCCGAACTTATTGAACA

Ale_MJ1_Clone13 TTTTCATTTTTTT-AACAAATTTTGAAAGTGTATCCGAACTTATTGAACA

Ale_MJ1_Clone2 TTTTCATTTTTTT-AACAAATTTTGAAAGTGTATCCGAACTTATTGAACA

Ape_MJ1_Clone1 TTTTCATTTTTTT-AACAAATTTTGAAAGTGTATCCGAACTTATTGAACA

Ape_MJ1_Clone3 TTTTCATTTTTTT-AACAAATTTTGAAAGTGTATCCGAACTTATTGAACA

CONTIG_574 TTTTCATTTTTTT-AACAAATTTTGAAAGTGTATCCGAACTTATTGAACA

CONTIG_7401 TTTTCATTTTTT--AACAAATTTTGAAAGTGTATCCGAACTTATTGAACA

CONTIG_10225 CCGTG-

CONTIG_29853 CCGTG-

CONTIG_11920 ------

CONTIG_8991 CCGTG-

CONTIG_23766 CCGTG-

CONTIG_13910 CCGTG-

CONTIG_4960 CCGTG-

Ape_MJ1_Clone6 CCGTG-

Ape_MJ1_Clone7 CCGTG-

Ape_MJ1_Clone2 CCGTG-

Ale_MJ1_Clone1 CCGTG-

Ale_MJ1_Clone11 CCGTG-

Ahy_MJ1_Clone1 CCGTG-

Ahy_MJ1_Clone2 CCGTG-

Ale_MJ1_Clone8 CCGTG-

Acr_MJ1_Clone2 CCGTG-

Acr_MJ1_Clone3 CCGTG-

Acr_MJ1_Clone1 CCGTG-

Ape_MJ1_Clone4 CCGTG-

Ape_MJ1_Clone5 CCGTG-

Ahy_MJ1_Clone3 CCGTG-

Ale_MJ1_Clone7 CCGTG-

Aju_MJ1_Clone3 CCGTG-

Aya_MJ1_Clone2 CCGTG-

Asi_MJ1_Clone2 CCGTG-

Asi_MJ1_Clone7 CCCGGG

Asi_MJ1_Clone1 CCGTG-

Asi_MJ1_Clone3 CCGTG-

Asi_MJ1_Clone4 CCGTG-

Asi_MJ1_Clone6 CCGTG-

Akl_MJ1_Clone3 CCGTG-

Aba_MJ1_Clone2 CCGTG-

Akw_MJ1_Clone3 CCGTG-

Aya_MJ1_Clone3 CCGTG-

Aju_MJ1_Clone1 CCGTG-

Asi_MJ1_Clone5 CCGTG-

Asi_MJ1_Clone9 CCGTG-

Akw_MJ1_Clone2 CCGTG-

Asi_MJ1_Clone8 CCGTG-

Akl_MJ1_Clone2 CCGTG-

Aya_MJ1_Clone1 CCGTG-

Akl_MJ1_Clone1 CCGTG-

Aba_MJ1_Clone3 CCGTG-

Aba_MJ1_Clone1 CCGTG-

Akw_MJ1_Clone1 CCGTG-

Aju_MJ1_Clone2 CCGTG-

Aju_MJ1_Clone7 CCGTG-

Ale_MJ1_Clone4 CCGTG-

Ale_MJ1_Clone10 CCGTG-

Aju_MJ1_Clone5 CCGTG-

Ale_MJ1_Clone5 CCGTG-

Aju_MJ1_Clone6 CCGTG-

Ale_MJ1_Clone3 CCGTG-

Ale_MJ1_Clone12 CCGTG-

Aju_MJ1_Clone4 CCGTG-

Aju_MJ1_Clone8 CCGTG-

Ale_MJ1_Clone9 CCGTG-

Ale_MJ1_Clone6 CCGTG-

Ale_MJ1_Clone13 CCGTG-

Ale_MJ1_Clone2 CCGTG-

Ape_MJ1_Clone1 CCGTG-

Ape_MJ1_Clone3 CCGTG-

CONTIG_574 CCGTG-

CONTIG_7401 CCGTG-

;

lset nst=6 rates=gamma ngammacat=4;

prset statefreqpr=fixed(.2814,.2231,.2298,.2657) revmatpr=fixed(1.0,4.7939,1.4641,1.4641,4.7939,1.0000) shapepr=fixed(2.9370) pinvarpr=fixed(0);

mcmc ngen=2500000 file=fasta2.out;
sump burnin=6250;
sumt burnin=6250;

end;
